# Electrochemical Synthesis of the *In Human S*-oxide Metabolites of Phenothiazine-Containing Antipsychotic Medications

**DOI:** 10.3390/molecules29133038

**Published:** 2024-06-26

**Authors:** Ridho Asra, Aigul Erbosynovna Malmakova, Alan M. Jones

**Affiliations:** 1 School of Pharmacy, Institute of Clinical Sciences, College of Medical and Dental Sciences, University of Birmingham, Birmingham B15 2TT, UK; 2 Bekturov Institute of Chemical Sciences, Almaty 050010, Kazakhstan

**Keywords:** electrochemistry, metabolite, phenothiazine, chlorpromazine

## Abstract

The tractable preparation of Phase I drug metabolites is a critical step to understand the first-pass behaviour of novel chemical entities (NCEs) in drug discovery. In this study, we have developed a structure–electroactivity relationship (SeAR)-informed electrochemical reaction of the parent 2-chlorophenothiazine and the antipsychotic medication, chlorpromazine. With the ability to dial-in under current controlled conditions, the formation of *S*-oxide and novel *S*,*S*-dioxide metabolites has been achieved for the first time on a multi-milligram scale using a direct batch electrode platform. A potential rationale for the electrochemical formation of these metabolites in situ is proposed using molecular docking to a cytochrome P_450_ enzyme.

## 1. Introduction

Phenothiazine (PTZ) is a heterocyclic pharmaceutical lead structure in medicinal chemistry [1], representing a major parent scaffold of antipsychotic drugs that have an inhibitory effect against dopaminergic receptors, especially D_2_ receptor [2]. Derivatives of PTZ have been extensively used to treat psychosis, including schizophrenia and bipolar disorder [3]. While these medications are effective, side effects of PTZs include (1) the autonomic and cardiovascular systems, endocrinologic, metabolic, hematologic, hepatic, allergic/dermatologic, ophthalmologic, and anticholinergic effects; (2) the central nervous system, including extrapyramidal symptoms, tardive dyskinesia, neuroleptic malignant syndrome, seizure, and sedation; (3) the sexual and reproductive side effects, including sexual dysfunction and teratogenicity [4]. These side effects are primarily caused by the PTZs’ non-selective blocking of receptors in the central nervous system, including dopamine (D_1_ and D_2_), muscarinic, histamine H_1_, and serotonergic 5-hydroxytryptamine (HT) 2 receptors [5]. Clinical studies have shown that these side effects occur due to bioactive metabolites, which may significantly contribute to the toxicity in humans [6]. Sulfoxide metabolites have been annotated to be responsible for cardiotoxic activity [7], and the 7-hydroxylated metabolites of the PTZs form an electrophilic quinone imine intermediate, which is responsible for mechanisms of drug-induced idiosyncratic hepatotoxicity [8]. PTZ analogues are classified into three groups, as shown in Table 1 [1,9].

**Scheme 1:**
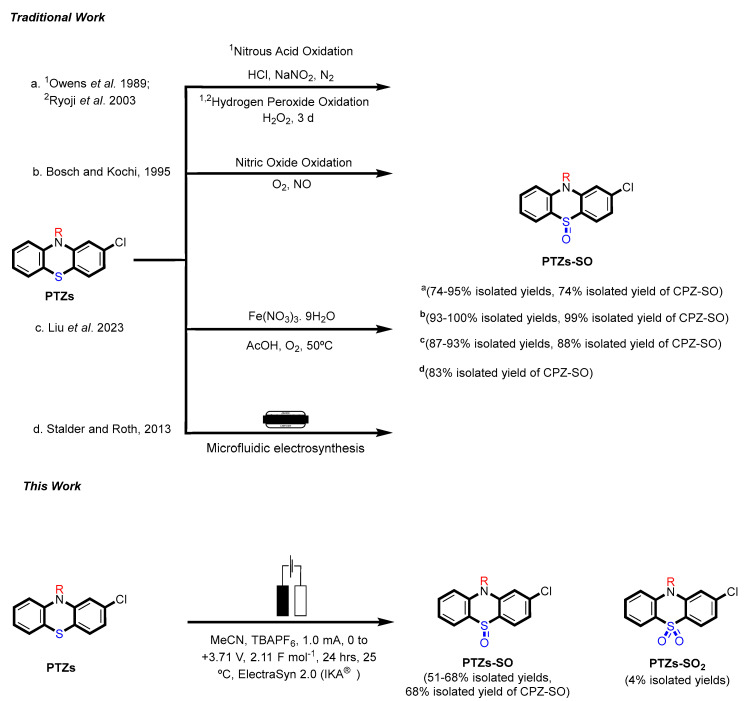
Previous approaches (a–d) [7,14,15,16,17] towards synthesising PTZs sulfoxide using chemical oxidants and microfluidic electrosynthesis and *this work*: a green electrosynthesis approach.

The 2-chlorophenothiazine (2CPTZ) is an important intermediate in the synthesis of neuroleptic drugs, such as chlorpromazine (CPZ), perphenazine, and prochlorperazine [10]. CPZ is subject to a significant amount of first-pass metabolism, including hydroxylation, *N*-dealkylation, *N*-oxidation, and *S*-oxidation [11,12,13]. Chlorpromazine (CPZ) is primarily metabolised as chlorpromazine-*S*-oxide (CPZ-SO) and was found to have the same effect as CPZ in the vasomotor and central nervous systems. Chemical methods to oxidise PTZ derivatives to sulfoxide are shown in Scheme 1. Owens and co-workers prepared phenothiazine sulfoxide (PTZ-SO) using aqueous nitrous acid and hydrogen peroxide at room temperature (PTZs-SO, 95% yield, with CPZ-SO, yield 74%) [14]. Ryoji and co-workers investigated the preparation of sulfoxide compounds using aqueous hydrogen peroxide, a tungstate complex, and a quaternary ammonium hydrogen-sulfate as a phase-transfer catalyst in 95% yield [15]. Bosch and colleagues also reported sulfoxidation using nitric oxide oxidation (CPZ-SO, 99% yield) [7]. Liu and colleagues used ferric nitrate nonahydrate (Fe(NO_3_)_3_·9H_2_O) as a catalyst and oxygen as a green oxidant for the selective oxidation of thioethers to sulfoxides, including CPZ with an 88% yield [16]. Stadler and Roth reported a microfluidic electrochemical preparation of CPZ-sulfoxide [17]. In total, four drugs were subjected to continuous-flow electrolysis, including the conversion of CPZ to CPZ-SO with an 83% isolated yield. However, this technique has some limitations, including (1) microfluidic-electrosynthesis can be complex to design, setup, and operate, and (2) high-cost equipment. The recent application of homogeneous catalysis in electrosynthesis has proven to be a strategy for controlling the chemo-, regio-, and enantio-selectivity of reactions [18], but as of yet is not reported for sulfoxidation.

In this work, electrochemistry was explored as a green technique [19] to determine the voltammetric behaviour [20] of PTZ analogues and synthesise the oxidation metabolites of 2CPTZ and CPZ [21,22]. The aim was to gain a deeper understanding of the oxidation outcomes of 2CPTZ and CPZ through electrosynthesis approaches and to examine how these metabolites relate to both drug metabolites (*in human*) and *in silico* metabolism predictions. Electrosynthesis advantages include: (1) a greener approach to synthesis, as the electron is the reagent, (2) a mild and safe oxidation condition (no heat is needed and the electric supply can be stopped instantaneously), (3) avoids the use of protective groups and additional chemical derivatisation steps, (4) phase I metabolism mimicry, (5) the capability to generate diverse drug metabolites, and (6) single-step metabolite synthesis. Additionally, *in silico* predictions to explore the oxidative sites of 2CPTZ and CPZ were examined to correlate to both the electrochemical and *in human* metabolite outcomes. The results of this study suggest that electrosynthesis can be a promising technique for the green synthesis of drug metabolites in drug discovery and development.

## 2. Result and Discussion

### 2.1. Structure–Electroactivity Relationship (SeAR) and Cyclic Voltammetry Studies

**Scheme 2:**
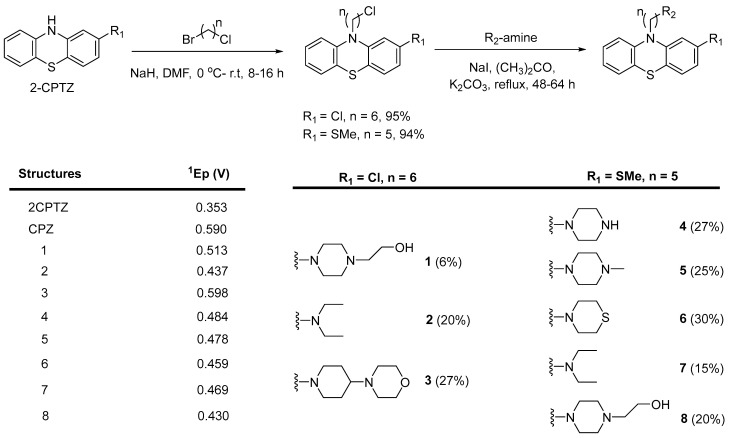
General synthesis of novel PTZ-containing structures to explore linker length and amine effect. Summary of initial oxidation potentials observed in PTZ analogues derived from cyclic voltammetry measurements.

To explore the structure–electroactive relationships (SeAR) [23] between the PTZ analogues represented by 2CPTZ and CPZ, we sought to develop a modular synthesis of R_1_ and R_2_ point variables during a related medchem campaign (Scheme 2).

**Figure 1:**
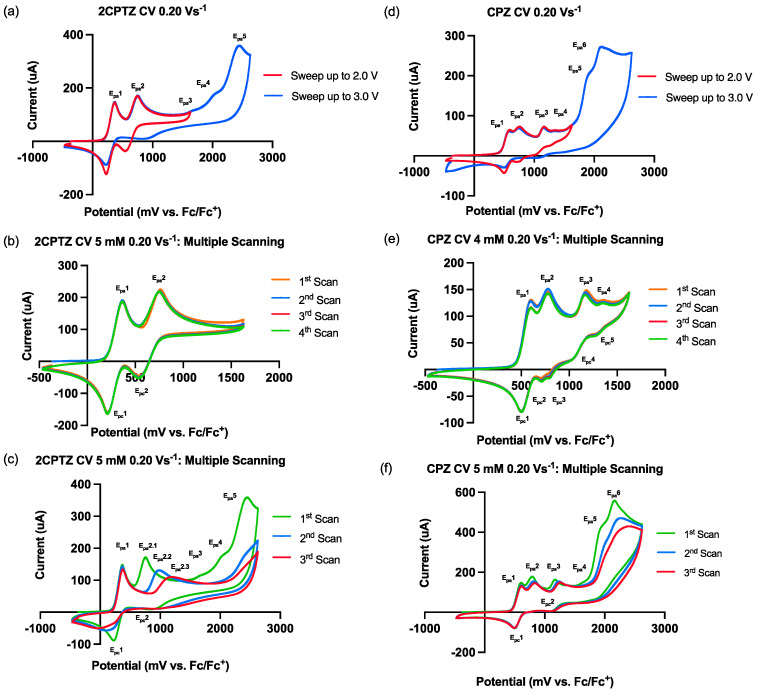
Cyclic voltammetry behaviour of 2CPTZ and CPZ, GCE (WE), Pt (CE), and Ag/AgCl (*pseudo* RE), referenced to Fc/Fc^+^. Electrolyte: TBAPF_6_; solvent: MeCN. (**a**) CV studies of 2CPTZ with multiple sweeps up to 2.0 V and 3.0 V, with a scan rate of 0.20 Vs^−1^. (**b**) Multiple-scan analysis of 2CPTZ (5 mM) sweeping up to 2.0 V. (**c**) Multiple-scan analysis of 2CPTZ (5 mM) sweeping up to 3.0 V. (**d**) CV studies of CPZ with multiple sweeps up to 2.0 V and 3.0 V, with a scan rate of 0.20 Vs^−1^. (**e**) Multiple-scan analysis of CPZ (4 mM) sweeping up to 2.0 V. (**f**) Multiple-scan analysis of CPZ (5 mM) sweeping up to 3.0 V.

With eight novel analogues, alongside 2CPTZ and CPZ, the cyclic voltammetric behaviour was determined (see the Supplementary Materials), and a summary of the effect of the functional group on oxidisability is shown in Scheme 2. Two clear structure–electroactivity relationship (SeAR) trends emerged. The presence of a chlorine group generally increased the difficulty with which the PTZ scaffold undergoes oxidation, compared to a thiomethyl group. The ease of oxidisability of the free *N*H analogue (2CPTZ) versus all alkylated analogues was explored (e.g., 0.353 V vs. 0.590 V for 2CPTZ vs. CPZ, respectively). With this tentative SeAR in hand, initial optimisation studies for the electrosynthesis of metabolites commenced with the parent scaffold, 2CPTZ, via enhanced CV studies [24]. To the best of our knowledge, no other CV study of 2CPTZ has been reported. The CV studies are shown in Figure 1.

Here, 2CPTZ had five oxidation events (Figure 1a), with initial oxidation peaks E_pa_1 376 mV/E_pc_1 218 mV and E_pa_2 756 mV/E_pc_2 542 mV identified as redox couples (most likely reversible *S*-radical cation formation in redox couple 1 and *S*-oxide radical cation formation in redox couple 2). Multiple-scan analysis (Figure 1b) revealed the reversibility of the electrochemical system being studied, with peak-to-peak separation of ΔE_p_1_quasi_ = 158 mV (moderate electron transfer) and ΔE_p_2_irrev_ = 214 mV (slow electron transfer). A scan up to +3.0 V showed three additional irreversible peaks, including E_pa_3 1676, E_pa_4 2027, and E_pa_5 2453 mV, respectively (Figure 1c). Ep_2_ shifted positively from 756 to 988 to 1176 mV during successive scans, indicating that the electrochemical processes occurring at the electrode surface may involve complex redox reactions and new chemical species. Thus, applying a potential greater than 2.453 V in the electrosynthesis reaction might ensure efficient oxidation of 2CPTZ. 

In contrast to 2CPTZ, CPZ had six oxidation events (Figure 1d). Specifically, the first oxidation peak corresponded to the moderate electron-transfer-reversible redox species at an anodic potential of 595 mV (E_pa_1) and a cathodic potential of 492 mV (E_pc_1), ΔE_p_1_quasi_: 103 mV. The CV profile of CPZ is in accordance with other reports [25]. This reversible redox reaction corresponds to the oxidation of CPZ to form radical cation CPZ^•+^ involving a one-electron process [26]. E_pa_2–4 were found to demonstrate slow electron transfer/be irreversible at 765, 1172, 1355, 1909, and 2113 mV vs. Fc/Fc^+^, respectively, as shown in Figure 1e. The irreversible process was confirmed in multiple-scan analysis, indicating that after the first scan, the reaction generated new chemical species/intermediate species in E_pa_2 (Figure 1f). E_pa_2 shifted to more positive potential from the first swept 1182 mV to the second swept 1240 mV and to the third swept 1268 mV.

### 2.2. Influence of Applied Current Variations on the PTZ Metabolites’ Electrosynthesis

**Figure 2:**
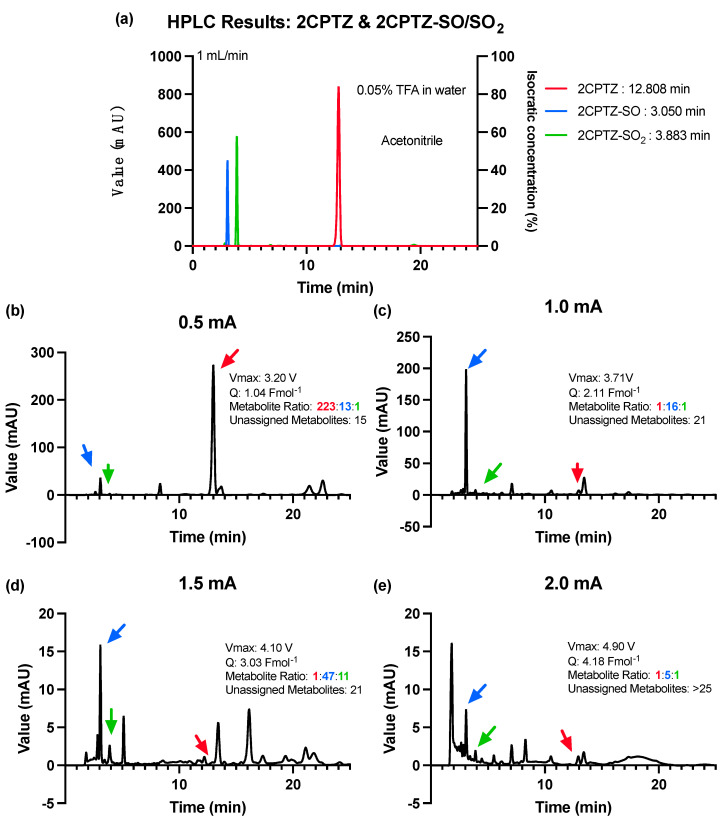
HPLC analysis of 2CPTZ metabolites’ electrosynthesis under different constant current conditions using an ElectraSyn 2.0 at 25 °C for 24 h. (**a**) HPLC of the 2CPTZ and 2CPTZ-SO/SO_2_, (**b**) constant current of 0.5 mA, (**c**) constant current of 1.0 mA, (**d**) constant current of 1.5 mA, and (**e**) constant current of 2.0 mA.

Informed by the CV studies, the electrosynthesis reaction was optimised to oxidise the PTZ by adjusting the desired current and avoiding the over-oxidation process [27]. A constant current of initially 0.5 mA, with 0.5 mA increments to 2 mA, was used for optimising the 2CPTZ oxidation reaction. The optimisation was concluded when the current used reached the maximum applied voltage of >+ 5.0 V for 24 h. All reactions were monitored by using HPLC to reference standards (Figure 2a). The starting current used for the electrosynthesis of 2CPTZ metabolites was 0.5 mA, with a maximum applied voltage of 3.2 V for 24 h. However, this was insufficient to oxidise 2CPTZ to generate the desired metabolite with a low metabolite conversion ratio, as shown in Figure 2b. The optimum condition was found to be a current of 1.0 mA (3.71 V), resulting in increased production of metabolite 2CPTZ-SO with a ratio of 1:16:1 for 2CPTZ, 2CPTZ-SO, and 2CPTZ-SO_2_, respectively (Figure 2c). Unidentifiable metabolites were formed at a constant current higher than 1.0 mA (Figure 2d,e). A constant current of 2 mA (4.90 V) resulted in over-oxidised products.

**Figure 3:**
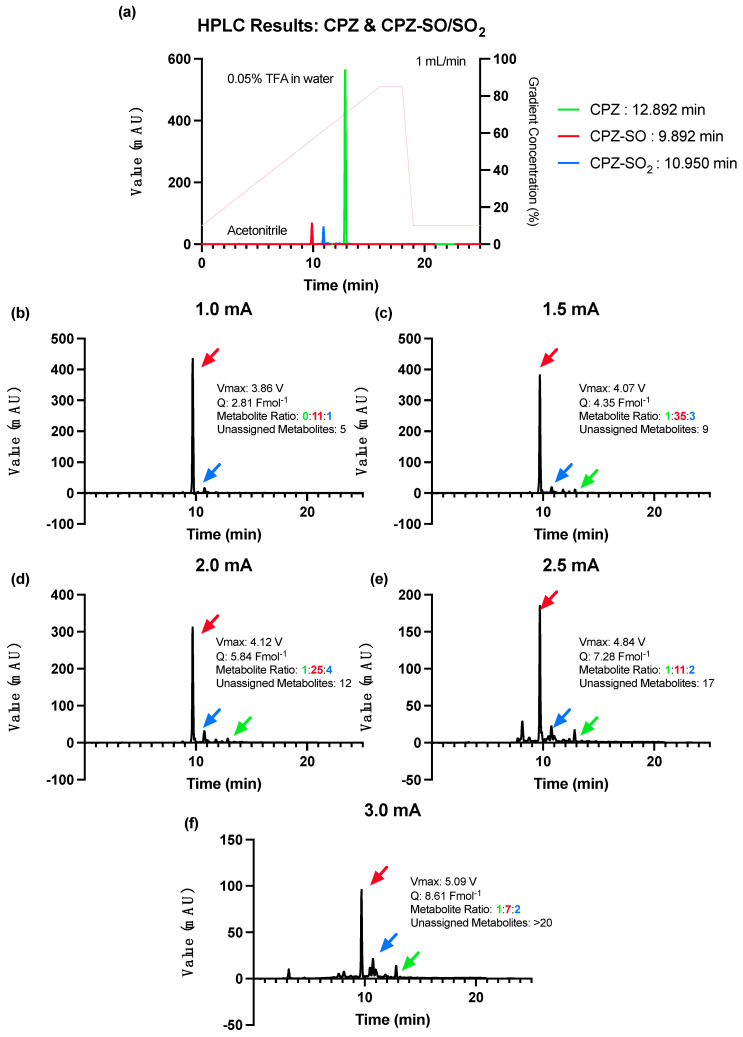
HPLC analysis of the CPZ metabolites’ electrosynthesis under different constant current conditions using ElectraSyn 2.0 at 25 °C for 24 h. (**a**) HPLC of the CPZ and CPZ-SO/SO_2_ with solvent gradient indicated in pink (acetonitrile and 0.05% TFA in water), (**b**) constant current of 1.0 mA, (**c**) constant current of 1.5 mA, (**d**) constant current of 2.0 mA, (**e**) constant current of 2.5 mA, and (**f**) constant current of 3.0 mA.

Informed by the optimisation step of the 2CPTZ metabolite reaction, a constant current of 1.0 mA was selected as the starting current for the CPZ reaction. Based on the CV profile, the first oxidation occurred at 595 mV, 0.2 Vs^−1^, higher than 2CPTZ (376 mV, 0.2 Vs^−1^). The optimal condition for CPZ metabolite electrosynthesis was achieved by using a constant current of 1.0 mA (3.86 V) with the highest percentage conversion of CPZ and selectivity for the CPZ-SO metabolite, CPZ-SO vs. CPZ-SO_2_, with a ratio of 11:1, respectively (Figure 3b). Higher currents of 1.5–3 mA (Figure 3c–f) led to more unassigned CPZ metabolites, with no improvement in the ratio of the desired metabolites, CPZ-SO and CPZ-SO_2_. 

### 2.3. Metabolite Profiling and Sulfoxidation Mechanism of PTZ Metabolites

It was observed during the 2CPTZ oxidation reaction that the reaction mixture changed from a clear solution to pink at 0.95 V and dark green at >1.25 V. This was most likely due to the one-electron oxidation [28]. The one-electron oxidation process led to the formation of radical cations of PTZs that exhibited a pink colour related to a broad visible spectral band at a peak of 520 nm [29]. The reaction colour gradually shifted to a dark green colour as the voltage increased above 1.25 V. At the end of the reaction, an unidentified dark green precipitate formed, likely polymeric products from *m*/*z* evidence (Table 2). It should be noted that increasing the current above 1 mA led to more unwanted products and increased precipitation after the electrosynthesis reaction. Conversely, no precipitation was observed in electrosynthesis of CPZ metabolites, even when using a higher constant current of 3 mA.

**Figure 4:**
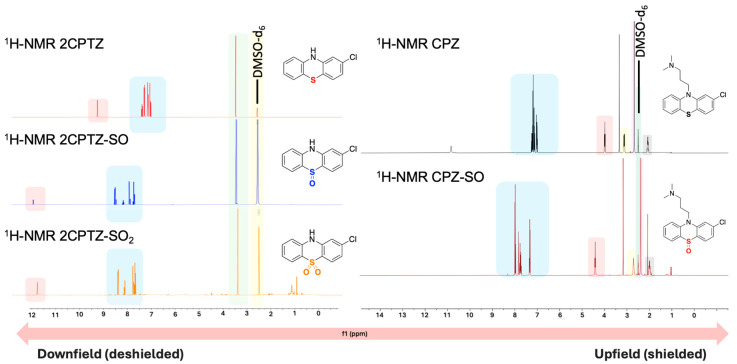
The stacked ^1^H-NMR spectra (400 MHz, DMSO-d_6_) of the PTZs studied and their respective metabolites synthesised via electrochemical oxidation.

The first step in purifying the metabolites formed in both reactions involved removing TBAPF_6_ from the crude reaction mixture through methanol recrystallisation, as outlined in Procedure B (Supplementary Materials), yielding a 72% recovery of the supporting electrolyte. The crystals were confirmed as unchanged and reusable TBAPF_6_ using ^1^H-NMR spectroscopic analysis (Supplementary Materials). Two major metabolites of each compound (2CPTZ and CPZ, respectively) were successfully isolated in their pure form after column chromatography, including the sulfoxide metabolite (2CPTZ-SO: 51% isolated yield, and CPZ-SO: 68% isolated yield) and sulfone metabolite (2CPTZ-SO_2_: 4% isolated yield, and CPZ-SO_2_: n.d.). The ^1^H NMR spectra of the metabolites are shown in Figure 4. 

The presence of both sulfoxide and sulfone groups significantly impacted the chemical shift of the aromatic protons, resulting in the de-shielding of the protons, as observed in ^1^H NMR spectra and the *N*H proton in 2CPTZ (Figure 4). The thioether in 2CPTZ underwent oxidation due to significant electron density localised in the sulfur unit [30,31,32,33,34,35]. As a result, the *S*-oxidation of 2CPTZ led to the formation of 2CPTZ-SO (2CPTZ-5-oxide) and 2CPTZ-SO_2_ (2CPTZ-5,5-dioxide), respectively. The formation of these two metabolites significantly altered the electronic properties of PTZs [36,37]. FTIR analysis of 2CPTZ-SO showed a stretching vibration of the S=O bond in the sulfoxide functional group at 987 cm^−1^. This is in accordance with the structural label via dual-frequency, two-dimensional infrared (2DIR) spectroscopy, where the S=O stretching mode in sulfoxide occurred in the frequency range of 950–1150 cm^−1^ [38]. Moreover, the stretching frequency band of the sulfone group of 2CPTZ existed at 1128 cm^−1^ in a symmetric form (label ~1118 cm^−1^) [39]. 

**Scheme 3:**
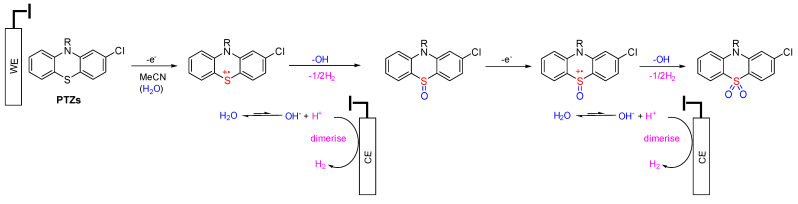
A potential mechanism for sulfoxide and sulfone formation from PTZs under electrochemical conditions in non-anhydrous acetonitrile solvent.

The mechanisms of sulfoxide and sulfone metabolites of PTZs are similar. According to the CPZ metabolites’ electrosynthesis, the existence of the sulfur atom (sulfide state, +2 oxidation) was more vulnerable to oxidation rather than the tertiary amine on the alkyl chain. It can be concluded that all PTZ derivates would undergo oxidation at the sulfide position. Liu and colleagues demonstrated that PTZ derivatives undergo oxidation at the sulfur position over the *N*-alkyl position using conventional chemical oxidants [16]. The proposed oxidation mechanisms of PTZ derivatives to form sulfoxide and sulfone are shown in Scheme 3.

**Scheme 4:**
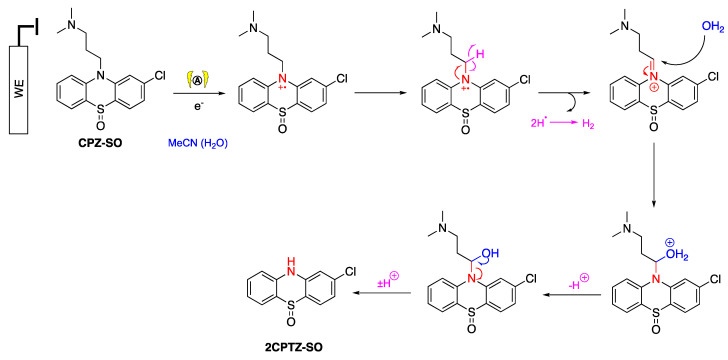
*N*-dealkylation mechanism of CPZ to 2CPTZ.

The oxidation process of PTZ occurred at the anode (working electrode), while the reduction process occurred at the cathode (counter-electrode) to maintain the balance of the oxidation reaction. One-electron oxidation of PTZ afforded a radical cation in non-anhydrous acetonitrile. Lee and coworkers presented a labelled control experiment in which water was the oxygen source in sulfoxide and sulfone formation [40], and water acted as a hydroxide source with hydrogen evolved at the cathode [41]. The hydroxide ions intercepted the *S*-centred radical cation, and the generated proton was reduced to produce hydrogen gas at the cathode. The presence of hydrogen gas can be inferred from pressure release upon opening the electrochemical vial cap. Further oxidation of the sulfoxide under analogous steps afforded the sulfone (Scheme 3). LCMS analysis was employed to identify other unpurified metabolites (Table 3). It was identified that one of the metabolites of CPZ is 2CPTZ-SO via an *N*-dealkylation reaction and sulfoxidation. A proposed mechanism of *N*-dealkylation is shown in Scheme 4. 

### 2.4. Computational Predictive and Electrochemical Detection of Metabolites (CP-EDM) of PTZs

**Figure 5:**
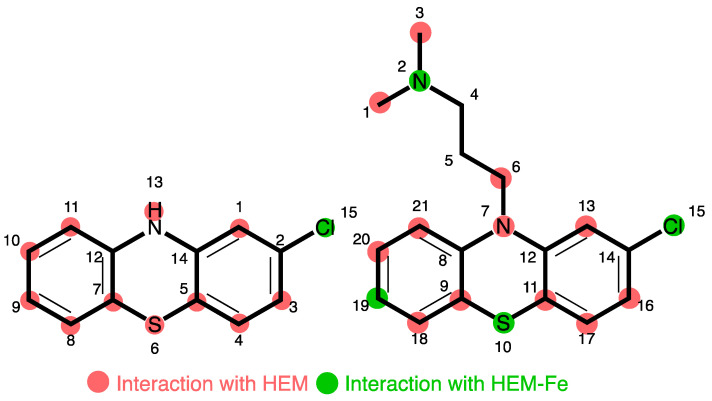
Summary of PTZ site interaction with heme-Fe species inferred from docking studies. Numbers relate to non-IUPAC atomic positions in the docking model.

Phase I metabolism of drugs usually involves chemical processes, such as hydroxylation, dealkylation, and oxidation to the corresponding *N*-oxide or sulfoxides [42]. Cytochromes (CYP1A2, CYP2B6, CYP2D6, CYP2C9, CYP2C19, and CYP3A4/5) are the pivotal isoenzymes involved in this metabolic event of most psychotropic drugs [43]. CYP1A2 and CYP3A4 are the primary isoforms responsible for catalysing 5-sulfoxidation (32% and 30%, respectively) [44]. Molecular docking was performed with Flare^TM^ V 8.0.0 from Cresset using multiple CYP isoenzymes, including 1A2, 3A4, 2B6, 2C9, and 2D6 (Figure 5). 

**Figure 6:**
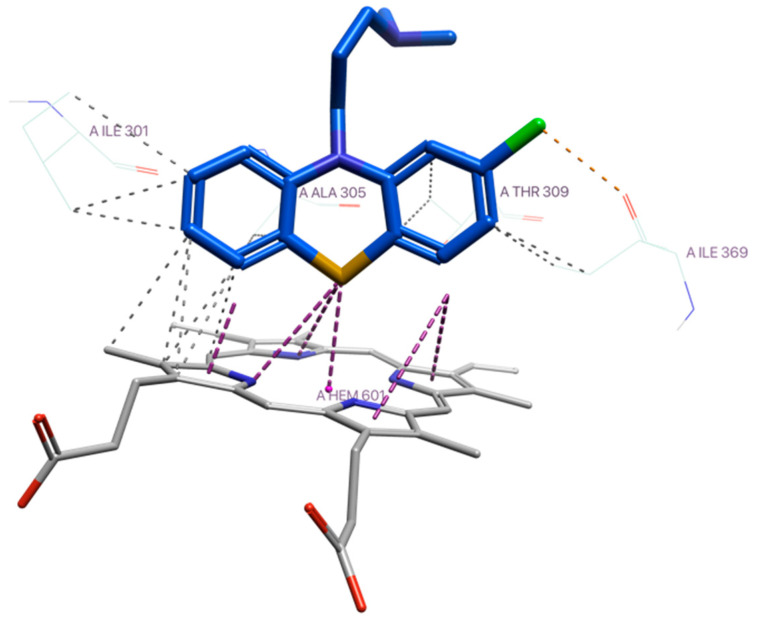
Thioether–heme-Fe interaction of CPZ with CYP3A4. Colour coding: protein in grey scale with nitrogen (blue) and oxygen (red) highlighted; and CPZ in blue carbon chain, with sulfur (yellow), chlorine (green) highlighted.

According to the docking results with CYP3A4 (Supplementary Materials), the sulfur atom of CPZ was observed to engage in a sulfur–ion pair interaction with heme-Fe at 3.0 Å (affinity energy: −7.45 kcal/mol), which would suggest that *S*-oxidation is a likely metabolic pathway with PTZs (Figure 6) [23].

## 3. Materials and Methods

### 3.1. Electroanalysis Studies of PTZs 

All drugs and chemical reagents were of analytical grade and used as-received, unless stated otherwise. They were purchased from Sigma-Aldrich^®^, St. Louis, MO, USA. Electroanalysis studies were performed using an Autolab potentiostat–galvanostat (PGSTAT 100 N, Barendrecht, The Netherlands), and CV staircase settings were controlled by the Autolab Nova 2.0 software. The CV experiments referenced ferrocene (Fc/Fc^+^) as an internal standard. An undivided glass cell (electrochemical cell) was equipped with a glassy carbon electrode (GCE BASI^®^ MF-2012, geometric area 0.071 cm^2^, 3.0 mm-diameter electrode disk of GCE material) as the working electrode, and a platinum wire (Sigma Aldrich^®^ 0.5 mm diameter) was used as a counter-electrode (CE). Ag/AgCl pseudo-reference wire was used as the reference electrode (RE). To this electrochemical setup, the corresponding samples were added to be analysed. Scan rates were varied, and the electroanalysis profile selected in the procedure and configuration was fixed with a 0.00244 V step potential and a start and stop potential of 0.0 V using the Autolab Nova 2.0 software. Before each experiment, the GCE was manually polished with 1.0 micron liquid diamond type K (Kemet, Maidstone, UK) on a smooth velvet polishing pad. The electrodes were rinsed with double-distilled, deionised water, followed by suitable solvents used in this study, and allowed to dry prior to the experiment. All CV data were exported to an Excel file and processed using Microsoft Excel^®^ version 16.69.1 and Prism 10.

### 3.2. Electrosynthesis of PTZ-Based Metabolites

All electrosynthesis of drug metabolites was performed using ElectraSyn 2.0 (IKA^®^) under a constant current control. An undivided glass cell (electrochemical vial) equipped with a magnetic stirrer was added to the analyte solution under study. Two glassy carbon electrodes (GCE; IKA^®^, dimensions (W × H × D = 8 × 52.5 × 2 mm)), as the working electrode (WE) and the counter-electrode (CE), were inserted into the conductive solution at an opposing distance of ~5 mm. Prior to the experiment, the electrodes were rinsed with double-distilled, deionised water, followed by MeCN, and allowed to air-dry. Solutions of 2-chlorophenothiazine (100 mg, 0.43 mmol) and chlorpromazine (100 mg, 0.31 mmol) were prepared in an electrochemical vial containing tetrabutylammonium hexafluorophosphate (TBAPF_6_; analyte:electrolyte 1:5) as the supporting electrolyte in 12 mL of MeCN. A fixed current of 1.0 mA was passed through the solution for 24 h until the desired charge (Q) was transferred (2CPTZ: 2.11 F/mol and CPZ: 2.81 F/mol), with a stirring speed of 500 rpm. The electrolysis products were analysed and monitored using TLC. TBAPF_6_ was removed by recrystallisation (procedure B). The crude mixture was then purified through the flash chromatography Biotage^®^ Isolera™ system and Biotage^®^ Sfär silica high-capacity duo columns of 20 µm (5, 10, 25, and 50 g) with Samplet^®^ and the indicated solvent systems, which afforded the corresponding isolated sulfoxide and sulfone metabolites. All metabolites were characterized using ^1^H- and ^13^C-NMR spectra and were recorded on a Bruker Ascend^TM^ 400 spectrometer operating at 400 and 101 MHz, fitted with a 5 mm “smart” BBFO probe, respectively. Mass spectra were recorded on a Waters Xevo G2-XS Tof or Synap G2-S mass spectrometer using Zspray and a Bruker microTOF^®^ LCMS using electrospray ionisation in positive (ESI^+^) and negative (ESI^−^) modes. Infrared spectra were recorded on a Thermo Scientific™ Nicolet™ iS™ 5 FTIR Spectrometer with ZnSe ATR crystal, reported in % transmittance vs. wavenumber in cm^−1^. 

### 3.3. Chromatographic Separation and Analysis

Chromatographic separation was carried out using a Waters Acquity SQD2 LC-MS with UPLC consisting of a quaternary pump, autosampler, column compartment, online degasser, and diode-array detector. The chromatographical separation was conducted on an Acquity UPLC BEH C_18_ column (Waters, Milford, MA, USA; 2.1 × 50 mm, i.d., 1.7 μm). The mass detection was carried out on a Waters SQD2 electrospray ionisation, single quadrupole mass spectrometer equipped with positive and negative electrospray ionisation (ESI) sources. All the operations and the post-data-processing were controlled by MassLynx 4.1 SCN855 software. High-performance liquid chromatography (HPLC) analysis was performed using the Thermo Scientific™ Vanquish™ Flex System with UV/VIS 4 wavelength setting refractive index detectors, equipped with Ascentis^®^ C18 HPLC Column RP-Amide, 25 cm × 4.6 mm I.D., 5 μm particles (581325-U). The post-data analysis was processed by Chromeleon 7.3.2. At each workday’s end, the column was first double-washed with acetonitrile (100 *v*/*v*) for 30 min and then acetonitrile–water (50:50 *v*/*v*) for 60 min.

### 3.4. Docking Procedures

Molecular docking was performed with Flare^TM^ V 8.0.0 from Cresset. The protein structures, including CYP1A2 (2HI4), CYP2B6 (4RQL), CYP3A4 (1TQN), CYP2C9 (4NZ2), and CYP2D6 (5TFT), were obtained from a protein data bank (PDB) and prepared using the following parameters: calculation method—normal, cap chains—intelligent capping, remove waters outside active side—yes, active site size—6.00 Å, and copy protein and auto-extract ligand (reference)—yes. The ligand was prepared using the following parameters: pop to 3D and minimize—yes. The docking calculation method was performed using the following parameters: method—very accurate but slow, number of runs—3, and maximum poses to output each ligand—100. The protein binding sites were investigated using the Lead Finder version 2212 build 1, 10 December 2022, in a total of 100 different conformations. The final poses were selected among the most negative energies and interactions with heme-Fe.

## 4. Conclusions

In this study, we comprehensively studied the electrochemical behaviour of a series of PTZ derivatives. Analysis of the cyclic voltammetry behaviour of PTZ derivatives revealed SeAR based on molecular characteristics and their respective influence on oxidation potential. Molecular modelling enabled the prediction of the metabolites most likely to form. Ultimately, an optimised electrochemical reaction platform for the parent scaffold and exemplar drug molecule enabled a tractable synthesis of the *S*-oxide and, for the first time, the *S*,*S*-dioxide metabolites. 

## Figures and Tables

**Table 1:** PTZs’ structure and their respective classifications (type 1–3) and bioactivities.

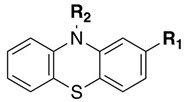				
Type of Compound	Example	Activity	R_1_	R_2_
Lead structure	phenothiazine	anthelmintic	H	H
Intermediate structure	2-chlorophenothiazine	-	-Cl	H
Type 1) Amino alkyl compound	chlorpromazine	sedatives and antipsychotic	-Cl	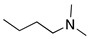
	promethazine	antihistamine	H	
Type 2) Piperidine	thioridazine	antimicrobial	-SCH_3_	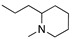
Type 3) Piperazine	trifluoperazine	anticonvulsant	-CF_3_	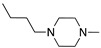
	fluphenazine	antipsychotic		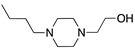

**Table 2:** MS analysis of the precipitation products from the 2CPTZ reaction (n.d. = not determined).

No.	HRMS (EI^+^)	HRMS (EI^−^)	Product
1	-	247.99	2CPTZ-SO
2	-	263.98	2CPTZ-SO_2_
3	271.17	-	n.d.
4	282.28	-	n.d.
5	465.01	462.99	polymeric products
6	506.53	-	
7	697.99	695.98	
8	-	731.96	
9	928.99	926.97	

**Table 3:** LCMS data of PTZ metabolites.

No.	Rt (min)	Name	MW ES+	MW ES−	MW	Structure
*2CPTZ*						
1	2.54	Metabolite 1 Unknown	-	186.2429	187	n.d.
2	2.69	Metabolite 2 2CPTZ-SO	250.0100	247.9937	249	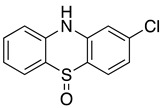 Chemical Formula: C_12_H_8_ClNOS
3	2.98	Metabolite 3 2CPTZ-SO_2_	266.0070	263.9906	265	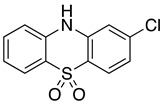 Chemical Formula: C_12_H_8_ClNO_2_S
4	3.45	Metabolite 4 Unknown	-	317.9141	319	n.d.
5	4.16	Metabolite 5 Unknown	-	276.9808	278	n.d.
*CPZ*						
1	1.89	Metabolite 1 CPZ-SO	335.1009	-	334.0933	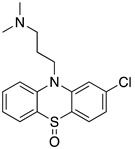 Chemical Formula: C_17_H_19_ClN_2_OS
2	2.10	Metabolite 2 CPZ-SO_2_	351.0937	-	350.0886	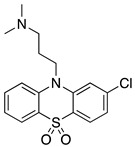 Chemical Formula: C_17_H_19_ClN_2_O_2_S
3	2.16	Metabolite 3 Unknown	369.0600	-	368.052	n.d.
4	2.28	Metabolite 4 Unknown	186.2199	-	185.2126	n.d.
5	2.51	Metabolite 5 Unknown	250.0128	247.9941	249.0015	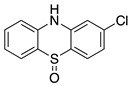 Chemical Formula: C_12_H_8_ClNOS
6	2.74	Metabolite 6 Unknown	349.0808	-	348.0735	n.d.

Key: LCMS ES^+^: actual molecular weight (amu) = MW from LCMS ES^+^ (amu)—Mass of proton (1.0073 amu), n.d. = not determined. Structures were generated from ChemDraw 20.0.0.41.

## Data Availability

The original contributions presented in the study are included in the article/Supplementary Material, further inquiries can be directed to the corresponding author.

## Supplementary Materials

The following supporting information can be downloaded at: https://www.mdpi.com/article/10.3390/molecules29133038/s1, General Experimental Methods, General Procedures, Compound Characterization, NMR and MS data, HPLC data, LCMS data, IR data, HPLC-LCMS data 2-Chlorophenothiazine Fractions, Cyclic voltammetry data on analogues, Molecular docking studies, and Biotransformer results.
