# Supplementary material for: Electrochemical Synthesis of the In Human S-oxide Metabolites of Phenothiazine-Containing Antipsychotic Medications
Source: Molecules. 2024 Jun 26;29(13):3038. doi: 10.3390/molecules29133038 (PMC11243251; doi:10.3390/molecules29133038)
Supplement: Supplementary file 1 [file molecules-29-03038-s001.zip › molecules-3072980-supplementary.pdf]

Electrochemical Synthesis of the *in human S*-oxide metabolites of phenothiazine-containing Antipsychotic Medications

Ridho Asra[1], Aigul Erbosynovna Malmakova[1,2], and Alan M. Jones[1*]

[1]School of Pharmacy, Institute of Clinical Sciences, College of Medical and Dental Sciences, University of Birmingham

[2] Bekturov Institute of Chemical Sciences, Almaty, 050010, Kazakhstan

* Corresponding author: Dr Alan M. Jones; +44(0)121-414-7288; a.m.jones.2@bham.ac.uk

CONTENTS

General Experimental Methods	Pages 2
General Procedures	Pages 4
Compound Characterization	Pages 7
NMR and MS data	Pages 16
HPLC data	Pages 28
LCMS data	Pages 49
IR data	Pages 58
HPLC-LCMS data and 2-Chlorophenothiazine Fractions	Pages 61
Cyclic voltammetry data on analogues	Pages 67
Molecular docking studies	Pages 85
Biotransformer results	Pages 108

General Experimental Methods

All electroanalysis studies were performed using an Autolab potentiostat galvanostat (PGSTAT 100 N, The Netherlands), and the CV staircase settings were controlled by the Autolab Nova 2.0 software. The CV experiments were performed with reference ferrocene (Fc/Fc^+) as an internal standard. An undivided glass cell (electrochemical cell) equipped with a glassy carbon electrode (GCE BASI® MF-2012, geometric area 0.071 cm^2 3.0 mm diameter electrode disk of GCE material) was used as the working electrode, and a platinum wire (Sigma Aldrich® 0.5 mm diameter) was used as the counter electrode (CE). Ag/AgCl pseudo reference wire was used as the reference electrode (RE). The corresponding samples were added to this electrochemical set up to be analysed. Scan rates were varied using the Autolab Nova 2.0 software. Before each experiment, the GCE was manually polished with 1.0-micron liquid diamond type K (Kemet, Maidstone, UK) on a smooth velvet polishing pad. The electrodes were rinsed with double-distilled, deionised water, followed by the suitable solvents that were used in this study, and were allowed to dry prior to the experiment. All CV data were exported to an Excel file and processed using Microsoft Excel® version 16.69.1. The linear regression equations were calculated using the least square method using Microsoft Excel® version 16.69.1.

All electrosyntheses of drug metabolites were performed using ElectraSyn 2.0 (IKA®) under a constant controlled current. An undivided glass cell (electrochemical vial) equipped with a magnetic stirrer was added to the analyte solution under study. Two glassy carbon electrodes (GCE) (IKA®, Dimensions (W x H x D = 8 x 52.5 x 2 mm) as the working electrode (WE) and the counter electrode (CE) were inserted into the conductive solution at distance of ~ 5 mm from one another. Prior to the experiment, the electrodes were rinsed with double-distilled, deionised water followed by MeCN, before being allowed to air dry.

All drugs and chemical reagents were of analytical grade and used as received unless stated otherwise. They were purchased from commercial suppliers, including Merk®/Sigma Aldrich®, Thermo Scientific®, MedChemExpress®, Toronto Research Chemicals®, and Cayman Chem Company®.

Solvents used for work-up procedures and column chromatography were of HPLC grade and were obtained from Merk®, Sigma-Aldrich®, VWR®, and Fischer Scientific®. Unless stated otherwise, solvents were removed by rotary evaporation under a reduced pressure between 30 and 50 °C, and all chemical reagents were utilised without any modifications or alterations unless otherwise stated.

The progress of reactions was monitored by thin layer chromatography (TLC) using Merck® or VWR® aluminium silica gel 60 F_{254} plates, which were visualised with UV light (254 nm) and iodine (I_2) vapour in an iodine chamber/with potassium permanganate. The preparative TLC for purification was purchased from Sigma Aldrich®.

Flash column chromatography was carried out using the Biotage® Isolera™ system and Biotage® Sfär silica high-capacity duo columns 20 µm (5, 10, 25, and 50 g) with Samplet® and the indicated solvent systems.

The melting point was measured using the melting point apparatus Gallenkamp®.

^{1}H- and ^{13}C-NMR spectra were recorded on a Bruker Ascend™ 400 spectrometer operating at 400 and 101 MHz, fitted with a 5 mm "smart" BBFO probe, respectively. Chemical shift data were reported in parts per million (ppm, δ scale) downfield from tetramethylsilane (TMS: δ 0.0), and referenced internally to the residual proton in the solvent. Wilmad® NMR tubes with a 5 mm frequency of 400 MHz and Merk® deuterated solvents were used for analysis including chloroform ($CDCl_3$: δ_H 7.26, δ_C 77.16), dimethyl sulfoxide ($(CD_3)_2SO$: δ_H 2.50, δ_C 39.52),

acetone ($(CD_3)_2CO$: δ_H 2.05, δ_C 29.84), methanol (CD_3OD: δ_H 3.31, δ_C 49.00), and deuterium oxide (D_2O: δ_H 4.78). Coupling constants (*J*) are given in hertz (Hz). The NMR data are presented as follows: chemical shift, multiplicity (s = singlet, d = doublet, t = triplet, q = quartet, p = pentet (quintet), m = multiplet, br = broad, app. = apparent and combinations thereof), coupling constant, integration, and structural assignment.

Mass spectra were recorded on a Waters Xevo G2-XS Tof or Synap G2-S mass spectrometer using Zspray and a Bruker microTOF® LCMS using electrospray ionisation in positive (ESI^+) and negative ($ESI^–$) modes.

Chromatographic separation was carried out using a Waters Acquity SQD2 LC-MS with UPLC consisting of a quaternary pump, autosampler, column compartment, online degasser, and diode array detector. The chromatographical separation was conducted on an Acquity UPLC BEH C_{18} column (Waters, Milford, MA, USA; 2.1 × 50 mm, i.d., 1.7 μm). The mass detection was carried out on a Waters SQD2 electrospray ionisation, single quadrupole mass spectrometer equipped with positive and negative electrospray ionisation (ESI) sources. All of the operations and the post-data processing were controlled by MassLynx 4.1 SCN855 software. High-performance liquid chromatography (HPLC) analysis was performed using Thermo Scientific™ Vanquish™ Flex System with UV/VIS 4 wavelength setting refractive index detectors, equipped with Ascentis® C18 HPLC Column RP-Amide, 25 cm x 4.6 mm I.D., 5 μm particles (581325-U). The post-data analysis was processed by Chromeleon 7.3.2. At the end of each workday, the column was first double washed with acetonitrile (100 v/v) for 30 minutes and then acetonitrile–water (50:50 v/v) for 60 minutes.

Infrared spectra were recorded on a Thermo Scientific™ Nicolet™ iS™ 5 FTIR Spectrometer with a ZnSe ATR crystal and reported in % Transmittance vs. wavenumber in $cm^{–1}$.

Molecular docking was performed with Flare™ V 8.0.0 from Cresset, and metabolism prediction was conducted by using BioTransformer 3.0 (http://biotransformer.ca)

General Procedures

Procedure A: Electrosynthesis of 2-Chlorophenothiazine Metabolites

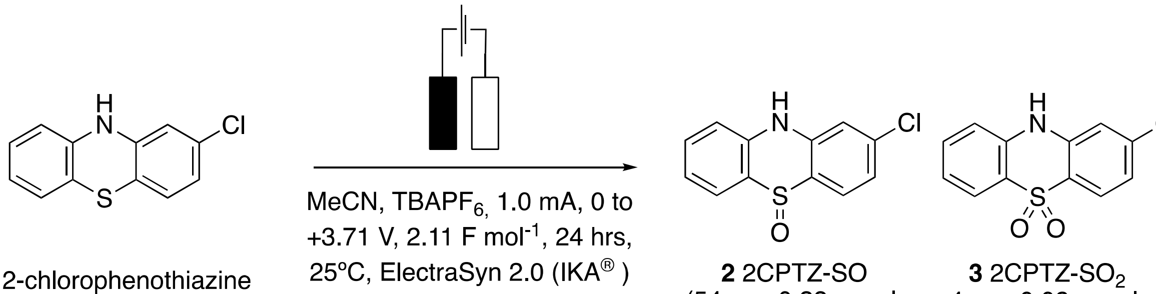

1 2-chlorophenothiazine (2CPTZ)

2 2CPTZ-SO (54 mg, 0.22 mmol, 51% isolated yield)

3 2CPTZ-SO_2 4 mg, 0.02 mmol, 4% isolated yield)

A solution of 2-chlorophenothiazine (0.43 mmol) was prepared in an electrochemical vial containing tetrabutylammonium hexafluorophosphate ($TBAPF_6$) (2.14 mmol) (analyte–electrolyte 1:5) as the supporting electrolyte in MeCN (12 mL). A fixed current of 1.0 mA was passed through the solution for 24 hours until the desired charge (Q) was transferred (2.11 F/mol) with a stirring speed of 500 rpm. The electrolysis product was analysed and monitored using TLC (SiO_2, eluent – cyclohexane: ethyl acetate – 3:2). $TBAPF_6$ was removed by recrystallisation (procedure B). The crude mixture was then purified through flash chromatography (Procedure C), which afforded the corresponding isolated metabolites **2** and **3** as a white solid and a pink solid, respectively (two primary isolated metabolites). Metabolite **2** was found to precipitate in ethyl acetate using procedure C.

Procedure B: Recrystallisation of $TBAPF_6$ (4)

The reaction mixture in MeCN was decanted into the flask, and MeCN was evaporated under reduced pressure using a rotary evaporator. Methanol or ethanol (15 mL) was added to dissolve the crude material and was then evaporated until a crystal of $TBAPF_6$ formed. The crystal of $TBAPF_6$ was separated from the filtrate, and the filtrate was cooled overnight in the fridge (0 °C) or cooled at -20 °C for 15 minutes, and the remaining crystal formed. The crystal of $TBAPF_6$ was collected either by filtration or using a chemical dropper to collect and separate the filtrate containing a mixture of drug metabolites. Recrystallisation from this process affords **4** as a bright white solid crystal (average yield 72.15%).

Procedure C: Flash Chromatography Conditions

The purification of 2-chlorophenothiazine metabolites using the Biotage® Isolera™ system is carried out as follows: Column: Biotage® Sfär silica high-capacity duo columns 20 µm (25 g) with Samplet®. Mobile phase: [A] cyclohexane; [B] ethyl acetate. Gradient: 5% B for 3 CV length; to 20% B for 5 CV length; held for 10 CV; to 100% B for 10 CV; held for 5 CV. Flow rate: 20 mL/min. UV detectors: 254 and 280 nm.

Procedure D: HPLC Analysis of 2-Chlorophenothiazine Metabolites

Preparation of Standard Solutions

A standard solution of 2-chlorophenothiazine (0.23 mg/mL) was accurately prepared using acetonitrile. The solution was then transferred to a 20 ml volumetric flask, and the remaining volume was made up. The solution was dissolved well following one minute of stirring.

Preparation of Samples

3 mg of the reaction mixture was dissolved in 20 mL MeCN, and then 1.5 mL of the solution was transferred to a 2 mL autosampler vial.

Chromatographic Conditions

The HPLC analysis conditions for 2-chlorophenothiazine metabolites are as follows: Column-Ascentis® C_{18} HPLC Column RP-Amide, 25 cm x 4.6 mm I.D., 5 µm particles (581325-U). Mobile phase: [A] 0.05% TFA in water; [B] acetonitrile (3:7). Flow rate: 1. mL/min. Injection: 5 µL. Chromatograms were recorded at 254 nm and 275 nm with a run time of 25 min.

Procedure E: Electrosynthesis of Chlorpromazine (CPZ) Metabolites

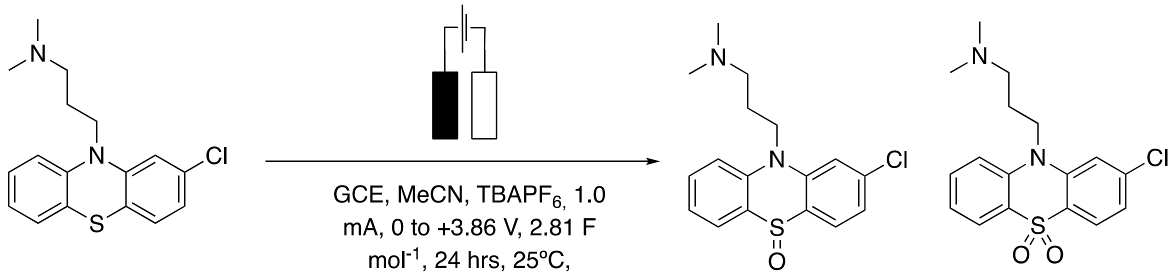

GCE, MeCN, $TBAPF_6$, 1.0 mA, 0 to +3.86 V, 2.81 F mol^{-1}, 24 hrs, 25°C, ElectraSyn 2.0 (IKA®)

5 Chlorpromazine (CPZ)

6 CPZ-SO (71 mg, 0.21 mmol, 68% isolated yield)

7 CPZ-SO_2 (n.d)

A solution of CPZ (100 mg, 0.31 mmol) was prepared in an electrochemical vial containing tetrabutylammonium hexafluorophosphate ($TBAPF_6$) (608 mg, 1.57 mmol) (analyte: electrolyte 1:5) as the supporting electrolyte in MeCN (12.0 mL). A fixed current of 1.0 mA was passed through the solution for 24 hours until the desired charge (Q) was transferred (2.81 F/mol) with a stirring speed of 500 rpm. The electrolysis product was analysed and monitored using TLC (SiO_2, eluent – DCM: methanol– 9:1). $TBAPF_6$ was removed by recrystallisation (procedure B). The crude mixture was then purified through flash chromatography (Procedure F), which afforded the corresponding isolated metabolites **6** and **7** as brown solids (two primary isolated metabolites).

Procedure F: Flash Chromatography Condition

The purification of chlorpromazine metabolites was performed using the Biotage® Isolera™ system as follows: Column: Biotage® Sfär silica high-capacity duo columns 20 µm (25 g) with Samplet®. Mobile phase: [A] DCM; [B] Methanol. Gradient: 0% B for 5 CV length to 10% B for 10 CV length which is held for 15 CV. Flow rate: 35 mL/min. UV detectors: 254 and 280 nm.

Procedure G: HPLC Analysis of CPZ Metabolites

Preparation of Standard Solutions

The standard solution of CPZ (0.15 mg/mL) was accurately prepared using MeCN. The solution was then transferred to a 20 ml volumetric flask, and the remaining volume was made up. The solution was dissolved well following one minute of stirring.

Preparation of Samples

3 mg of the reaction mixture was dissolved in 20 mL MeCN, and then 1.5 mL of the solution was transferred to a 2 mL autosampler vial.

Chromatographic Conditions

The HPLC analysis conditions for CPZ metabolites are as follows: Column-Ascentis® C_{18} HPLC Column RP-Amide, 25 cm x 4.6 mm I.D., 5 µm particles (581325-U). Mobile phase: [A]

0.05% TFA in water; [B] acetonitrile. Gradient: 10 to 85% B in 16 min, held for 2 min, to 10% B in 1 min, which is held for 2.5 min. Flow rate: 1.0 mL/min. Injection: 5.0 µL. Chromatograms were recorded at 254 with a run time of 25 min.

Compound Characterisation

Alkylation of 2-chloro-10*H*-phenothiazine with 1-bromo-6-chlorohexane

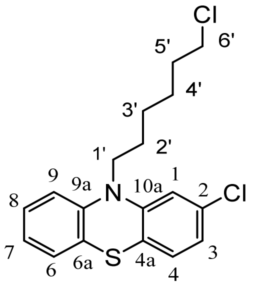

To 2-chloro-10*H*-phenothiazine (1.0 g, 4.29 mmol, 1 eq.) in DMF (10 mL) at 0 ^{o}C was added NaH (60% w/w in mineral oil, 0.205 g, 5.136 mmol, 1.2 eq.) and the mixture was stirred for 30 min and allowed to warm up to room temperature. This solution was then added dropwise to a stirred solution of 1-bromo-6-chlorohexane (0.766 mL, 5.136 mmol, 1.2 eq.) in DMF (2 mL) and stirred overnight until completion (as measured by TLC (R_f=0.46, SiO_2, eluent - hexane:EtOAc – 99:1)). Deionized water (50 mL) was added and the mixture was extracted with EtOAc (3x50 mL). The combined organic fractions were washed with deionized water (2x100 mL) and brine (100 mL), dried with $MgSO_4$, filtered, and then concentrated under reduced pressure. The crude product (1.42 g, 95%) without purification was used in the next stage.

^{1}H NMR (400 MHz, Chloroform-*d*) δ 7.20 – 7.10 (m, 2H, Ar-H), 7.03 (d, *J* = 8.2 Hz, 1H, Ar-H), 6.97 – 6.79 (m, 4H, Ar-H), 3.83 (t, *J* = 7.0 Hz, 2H, C1'-H), 3.55 – 3.46 (m, 2H, C6'-H), 1.85 – 1.69 (m, 4H, C2' & C5'-H), 1.46 (dq, *J* = 7.3, 3.8 Hz, 4H, C3' & C4'-H).

^{13}C NMR (101 MHz, $CDCl_3$) δ 146.7 (C_q -Ar), 144.7 (C_q -Ar), 133.3 (C_q -Ar), 128.1 (C_q -Ar), 127.7 (C_q -Ar), 127.5 (C_q -Ar), 125.1 (C_q -Ar), 123.8 (C_q -Ar), 123.0 (C_q -Ar), 122.4 (C_q -Ar), 115.9 (C_q -Ar), 47.4 (C1'), 45.1 (C6'), 32.6 (C2'), 26.7 (C5'), 26.6 (C3'), 26.3 (C4').

2-(4-(6-(2-Chloro-10*H*-phenothiazin-10-yl)hexyl)piperazin-1-yl)ethan-1-ol (1)

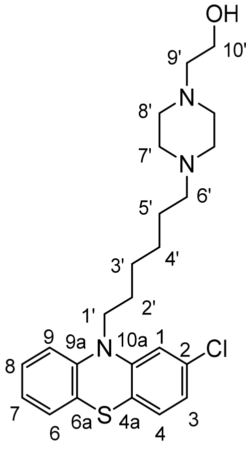

To 2-chloro-10-(6-chlorohexyl)-10*H*-phenothiazine (0.3 g, 0.854 mmol) in anhydrous acetone (15 mL) was added sodium iodide (0.154 g, 1.026 mmol), potassium carbonate (0.141 g, 1.020 mmol), and 1-(2-hydroxyethyl)piperazine (0.13 mL, 1.026 mmol). The reaction mixture was refluxed for 48 h upon completion (as measured by TLC (R_f=0.52, SiO_2, eluent - DCM:methanol – 9:1)). Then, the reaction mixture was quenched with water (30 mL) and

extracted with EtOAc (3x30 mL). The organic layer was washed with brine (100 mL), dried ($MgSO_4$), filtered, and then concentrated in vacuo. The product was separated via a flash column (SiO_2, eluent; DCM–methanol—9:1) into a purple oily compound (0.024 g, 6%).

^{1}H NMR (400 MHz, Chloroform-*d*) δ 7.13 (ddd, *J* = 17.0, 7.8, 1.6 Hz, 2H, Ar-H), 7.01 (d, *J* = 8.2 Hz, 1H, Ar-H), 6.95 – 6.78 (m, 4H, Ar-H), 3.80 (t, *J* = 7.0 Hz, 2H, C1'-H), 3.64 – 3.57 (m, 2H C6'-H), 2.81 – 2.36 (m, 11H, C7'-H & C8'-H &C 9-H & OH), 2.34 – 2.27 (m, 2H, C10'-H), 1.84 – 1.73 (m, 2H, C2'-H), 1.53 – 1.38 (m, 4H, C3'-H&C5'-H), 1.36 – 1.26 (m, 2H, C4'-H).

^{13}C NMR (101 MHz, $CDCl_3$) δ 146.7 (C10a), 144.7 (C9a), 133.3 (C2), 128.0 (C8), 127.6 (C4), 127.5 (C6), 124.9 (C5a), 123.7 (C4a), 122.9 (C3), 122.3 (C7), 115.9 (C1,9), 59.3 (C9', 10'), 58.6 (C7'), 57.8 (C8'), 53.3 (C6'), 52.9 (C1'), 47.4 (C5'), 27.2 (C2'), 26.8 (C3', 4').

LRMS: m/z (rel. abundance) 446.20 (M+H, 100%), 447.20 (30%), 448.20 (40%), 449.20 (15%), 450.20 (5%).

HRMS. *m/z* (ESI+) calculated for $C_{24}H_{33}ClN_3OS$, 446.2027 $[M^+H]^+$, found: 446.2033 .

6-(2-Chloro-10*H*-phenothiazin-10-yl)-*N,N*-diethylhexan-1-amine (2)

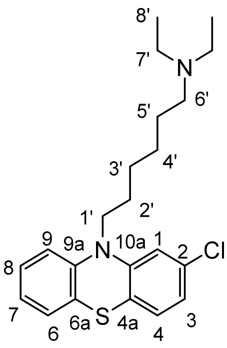

To 2-chloro-10-(6-chlorohexyl)-10*H*-phenothiazine (0.3 g, 0.854 mmol) in anhydrous acetone (15 mL) was added sodium iodide (0.154 g, 1.026 mmol), potassium carbonate (0.141 g, 1.020 mmol), and diethylamine (0.105 mL, 1.026 mmol). The reaction mixture was refluxed for 48 h upon completion (as measured by TLC (R_f=0.66; SiO_2, eluent; DCM–methanol— 9:1)). Then, the reaction mixture was quenched with water (30 mL) and extracted with EtOAc (3x30 mL). The organic layer was washed with brine (100 mL), dried ($MgSO_4$), filtered, and then concentrated in vacuo. The product was separated via a flash column (SiO_2, eluent; DCM–methanol—9:1) into a purple oil (0.060 g, 20%).

^{1}H NMR (400 MHz, Chloroform-d) δ 7.18 – 7.09 (m, 2H, Ar-H), 7.01 (d, J = 8.2 Hz, 1H, Ar-H), 6.95 – 6.78 (m, 4H, Ar-H), 3.82 (t, J = 6.9 Hz, 2H, C1'-H), 2.77 (q, J = 7.2 Hz, 4H, C7'-H), 2.67 – 2.58 (m, 2H, C6'-H), 1.78 (p, J = 7.1 Hz, 2H, C2'-H), 1.60 (ddd, J = 11.5, 9.8, 6.4 Hz, 2H, C5'-H), 1.45 (ddd, J = 15.0, 9.3, 6.9 Hz, 2H, C3'-H), 1.37 – 1.28 (m, 2H, C4'-H), 1.18 (t, J = 7.2 Hz, 6H, C8'-H).

^{13}C NMR (101 MHz, $CDCl_3$) δ 146.7 (C10a), 144.7 (C9a), 133.3 (C2), 128.1 (C8), 127.7 (C4), 127.6 (C6), 124.9 (C5a), 123.7 (C4a), 123.0 (C3), 122.4 (C7), 115.9 (C1, 9), 51.9 (C6'), 47.3 (C7'), 46.8 (C1'), 26.9 (C2'), 26.6 (C3'), 26.5 (C4'), 25.0 (C5'), 10.2 (C8').

LRMS: m/z (rel. abundance) 389.18 (M+H, 100%), 390.18 (30%), 391.18 (40%), 392.18 (10%), 393.18 (3%).

HRMS. *m/z* (ESI+) calculated for $C_{22}H_{30}ClN_2S$, 389.1813 $[M+H]^+$, found: 389.1818.

4-(1-(6-(2-Chloro-10H-phenothiazin-10-yl)hexyl)piperidin-4-yl)morpholine (3)

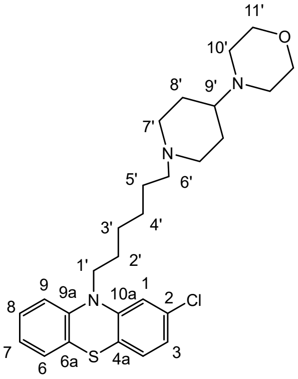

To 2-chloro-10-(6-chlorohexyl)-10*H*-phenothiazine (0.300 g, 0.854 mmol) in anhydrous acetone (15 mL) was added sodium iodide (0.154 g, 1.026 mmol), potassium carbonate (0.142 g, 1.026 mmol), and 4-morpholinopiperidine (0.175 g, 1.026 mmol). The reaction mixture was refluxed for 62 h upon completion (as measured by TLC (R_f=0.17; SiO_2, eluent; DCM–methanol—9:1)). Then, the reaction mixture was quenched with water (50 mL) and extracted with EtOAc (3 x 30 mL). The organic layer was washed with brine (100 mL), dried ($MgSO_4$), filtered and concentrated in vacuo. The product was separated via a flash column (SiO_2, eluent; DCM–methanol—9:1) into a yellow oil (0.11 g, 27%).

^{1}H NMR (400 MHz, Chloroform-*d*) δ 7.18 – 7.08 (m, 2H, Ar-H), 7.01 (d, *J* = 8.1 Hz, 1H, Ar-H), 6.96 – 6.77 (m, 4H, Ar-H), 3.80 (t, *J* = 7.0 Hz, 2H, C1'-H), 3.75 – 3.66 (m, 4H, C10'-H), 2.93 (dt, *J* = 12.1, 3.4 Hz, 2H, C6'-H), 2.60 – 2.49 (m, 4H, C11'-H), 2.31 – 2.11 (m, 3H, C8'-H (1/2) & C9'-H), 1.93 – 1.71 (m, 6H, C8'-H (1/2) & C7'-H), 1.60 – 1.23 (m, 8H, C2'-H, C3'-H, C4'-H & C5'-H).

^{13}C NMR (101 MHz, CDCl3) δ 146.7 (C10a), 144.7 (C9a), 133.3 (C2), 128.0 (C8), 127.6 (C4), 127.5 (C6), 124.9 (C5a), 123.6 (C4a), 122.9 (C3), 122.3 (C7), 115.9 (C1, 9), 67.5 (C11'), 62.4 (C10'), 58.6 (C6'), 53.3 (C7'), 49.8 (C1'), 47.5 (C9'), 28.1 (C5'), 27.3 (C2'), 27.1 (C8'), 26.8 (C3'), 26.8 (C4').

LRMS: m/z (rel. abundance) 486.2346 (M+H, 100%), 487.2377 (30%), 488.2326 (40%), 489.2350 (15%), 490.2333 (5%).

HRMS. *m/z* (ESI+) calculated for $C_{27}H_{37}ClN_3OS$, 486.2340 $[M+H]^+$, found: 486.2346.

Alkylation of 2-methylthio-10*H*-phenothiazine with 1-bromo-5-chloropentane

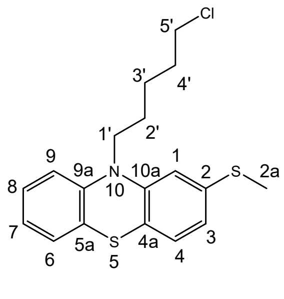

To NaH (60% w/w in mineral oil, 0.392 g, 16.3 mmol, 2 eq.) in DMF (5 mL), 2-methylthio-10*H*-phenothiazine (2.0 g, 8.15 mmol, 1 eq.) in DMF (4 mL) was added slowly at 0 ^{o}C under an argon atmosphere. After 1 h, 1-bromo-5-chloropentane (2.14 mL, 16.3 mmol, 2 eq.) was added and stirred for 10 h upon completion (as measured by TLC (R_f=0.44; SiO_2, eluent; hexane–EtOAc—99:1)). Deionized water (50 mL) was added and the mixture was extracted with DCM (3x50 mL). The combined organic fractions were washed with deionized water (2x100 mL) and brine (100 mL), dried with $MgSO_4$, filtered, and then concentrated under reduced pressure. The crude product (2.84 g, >99%) without purification was used in the next stage.

^{1}H NMR (400 MHz, Chloroform-*d*) δ 7.20 – 6.76 (m, 7H, Ar - H), 3.53 (dt, *J* = 16.8, 6.6 Hz, 2H, C1' - H), 2.47 (s, 3H, C2a - H), 1.95 – 1.73 (m, 6H, C2'&C4'&C5' - H), 1.65 – 1.50 (m, 2H, C3' - H).

^{13}C NMR (101 MHz, $CDCl_3$) δ 148.6 (C10a), 145.0 (C9a), 137.7 (C2), 127.7 (C8), 127.4 (C6), 125.6 (C5a), 122.9 (C4a), 121.1 (C7), 116.0 (C1), 114.8 (C9), 47.4 (C5'), 44.9 (C1'), 32.0 (C4'), 25.7 (C2'), 24.7 (C3'), 16.1 (C2a).

2-(Methylthio)-10-(5-(piperazin-1-yl)pentyl)-10H-phenothiazine (4)

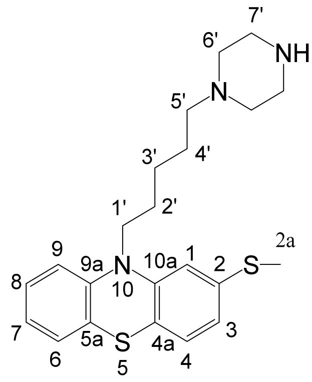

To 2-methylthio-10-(5-chloropenyl)-10H-phenothiazine (0.2 g, 0.572 mmol) in anhydrous acetone (12 mL) was added sodium iodide (0.103 g, 0.686 mmol), potassium carbonate (0.095 g, 0.686 mmol), and piperazine (0.059 mL, 0.686 mmol). The reaction mixture was refluxed for 64 h upon completion (as measured by TLC (R_f=0.60, SiO_2, eluent - DCM:methanol – 9:1)). Then, the reaction mixture was quenched with water (30 mL), and extracted with EtOAc (3x30 mL). The organic layer was washed with brine (100 mL), dried ($MgSO_4$), filtered and concentrated in vacuo. The product was separated via a flash column (SiO_2, eluent;– DCM–methanol—9:1) into an oily compound (0.062 g, 27 %).

^{1}H NMR (400 MHz, Chloroform-*d*) δ 7.16 – 7.09 (m, 2H, Ar-H), 7.03 (dd, *J* = 8.0, 1.2 Hz, 1H, Ar-H), 6.94 – 6.74 (m, 4H, Ar-H), 3.83 (t, *J* = 7.0 Hz, 2H, C1'-H), 2.66 – 2.23 (m, 11H, C5'-H & C6'-H & C7'-H & NH), 2.15 (d, *J* = 14.1 Hz, 2H. C2'-H), 1.96 – 1.73 (m, 3H, C2a-H), 1.56 – 1.33 (m, 4H, C3'-H & C4'-H).

^{13}C NMR (101 MHz, $CDCl_3$) δ 145.9 (C10a), 145.1 (C9a), 137.6 (C2), 127.7 (C8), 127.3 (C6), 125.3 (C5a), 122.7 (C4a), 120.9 (C7), 115.8 (C1), 114.7 (C9), 58.6 (C6'), 53.5 (C5'), 53.2 (C1'), 53.1 (C7'), 47.3 (C4'), 26.9 (C2'), 24.10 (C3'), 16.7 (C2a).

LRMS: m/z (rel. abundance) 400.19 (M+H, 100%), 401.19 (30%), 402.19 (15%), 403.19 (5%).

HRMS. *m/z* (ESI+) calculated for $C_{22}H_{30}N_3S_2$, 400.1881 $[M+H]^+$, found: 400.1881.

10-(5-(4-Methylpiperazin-1-yl)pentyl)-2-(methylthio)-10H-phenothiazine (5)

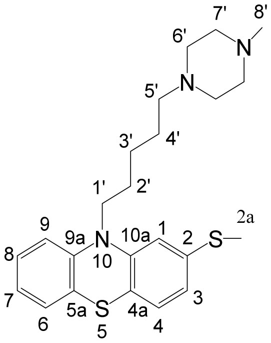

To 2-methylthio-10-(5-chloropenyl)-10H-phenothiazine (0.2 g, 0.572 mmol) in anhydrous acetone (12 mL) was added sodium iodide (0.103 g, 0.686 mmol), potassium carbonate (0.095 g, 0.686 mmol), and methyl piperazine (0.076 mL, 0.686 mmol). The reaction mixture was refluxed for 64 h upon completion (as measured by TLC (R_f=0.44; SiO_2, eluent; DCM–methanol— 9:1)). Then, the reaction mixture was quenched with water (30 mL) and extracted with EtOAc (3x30 mL). The organic layer was washed with brine (100 mL), dried ($MgSO_4$), filtered, and then concentrated in vacuo. The product was separated via a flash column (SiO_2, eluent;– DCM—methanol— 9:1) into an oily compound (0.059 g, 25%).

^{1}H NMR (400 MHz, Chloroform-*d*) δ 7.15 – 7.08 (m, 2H, Ar-H), 7.02 (d, *J* = 8.0 Hz, 1H, Ar-H), 6.91 – 6.73 (m, 4H, Ar-H), 3.82 (t, *J* = 7.0 Hz, 2H, C1'-H), 2.51 – 2.24 (m, 16H, C5'-H, C6'-H & C7'-H & C2a-H & C8'-H), 1.79 (p, *J* = 7.2 Hz, 2H, C2'-H), 1.46 (dddd, *J* = 17.4, 15.1, 9.8, 4.8 Hz, 4H, C3'-H & C4'-H).

^{13}C NMR (101 MHz, $CDCl_3$) δ 145.9 (C10a), 145.1 (C9a), 137.5 (C2), 127.6 (C8), 127.3 (C6), 125.3 (C5a), 122.6 (C4a), 122.3 (C3), 120.9 (C7), 115.8 (C1), 114.7 (C9), 58.6 (C7'), 55.2 (C5'), 53.3 (C6'), 46.9 (C1'), 46.1 (C8'), 26.8 (C4'), 26.6 (C2'), 24.9 (C3'), 16.6 (C2a).

LRMS: m/z (rel. abundance) 414.20 (M^+H, 100%), 415.20 (30%), 416.20 (15%), 417.20 (5%).

HRMS. *m/z* (ESI+) calculated for $C_{23}H_{32}N_3S_2$, 413.2032 $[M+H]^+$, found: 414.2038

2-(Methylthio)-10-(5-thiomorpholinopentyl)-10*H*-phenothiazine (6)

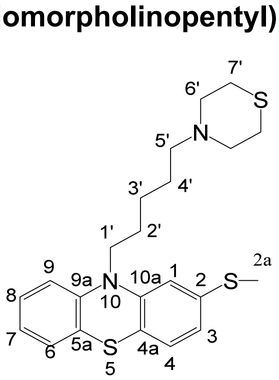

To 2-methylthio-10-(5-chloropenyl)-10*H*-phenothiazine (0.2 g, 0.572 mmol) in anhydrous acetone (12 mL) was added sodium iodide (0.103 g, 0.686 mmol), potassium carbonate (0.095 g, 0.686 mmol), and thiomorpholine (0.070 mL, 0.686 mmol). The reaction

mixture was refluxed for 52 h upon completion (as measured by TLC (R_f=0.86; SiO_2, eluent; DCM–methanol—9:1)). Then, the reaction mixture was quenched with water (30 mL), and extracted with EtOAc (3x30 mL). The organic layer was washed with brine (100 mL), dried ($MgSO_4$), filtered and concentrated in vacuo. The product was separated via a flash column (SiO_2, eluent;– DCM–methanol—9:1) into an oily compound (0.072 g, 30%).

^{1}H NMR (400 MHz, Chloroform-*d*) δ 7.13 (t, *J* = 7.9 Hz, 2H, Ar-H), 7.03 (d, *J* = 8.0 Hz, 1H, Ar-H), 6.93 – 6.74 (m, 4H, Ar-H), 3.83 (t, *J* = 6.9 Hz, 2H, C1'-H), 2.63 (s, 8H, C6'-H & C7'-H), 2.45 (s, 3H, C2a-H), 2.34 – 2.27 (m, 2H, C5'-H), 1.79 (p, *J* = 7.1 Hz, 2H, C2'-H), 1.53 – 1.35 (m, 4H, C3'-H & C4'-H).

^{13}C NMR (101 MHz, $CDCl_3$) δ 145.8 (C10a), 145.0 (C9a), 137.5 (C2), 127.6 (C8), 127.5 (C4), 127.3 (C6), 125.3 (C5a), 122.6 (C4a), 122.2 (C3), 120.8 (C7), 115.74 (C1), 114.7 (C9), 59.2 (C6'), 55.1 (C5'), 47.2 (C1') , 28.0 (C7'), 26.8 (C4'), 26.2 (C2'), 24.8 (C3'), 16.6 (C2a).

LRMS: m/z (rel. abundance) 417.15 (M+H, 100%), 418.15 (30%), 419.15 (20%), 420.15 (5%).

HRMS. *m/z* (ESI+) calculated for $C_{22}H_{29}N_2S_3$, 417.1487 $[M+H]^+$, found: 417.1493

***N,N*-Diethyl-5-(2-(methylthio)-10*H*-phenothiazin-10-yl)pentan-1-amine (7)**

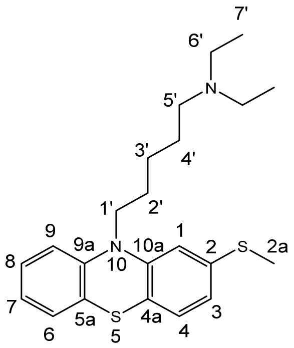

To 2-methylthio-10-(5-chloropenyl)-10*H*-phenothiazine (0.2 g, 0.572 mmol) in anhydrous acetone (12 mL) was added sodium iodide (0.103 g, 0.686 mmol), potassium carbonate (0.095 g, 0.686 mmol), and diethylamine (0.070 mL, 0.686 mmol). The reaction mixture was refluxed for 52 h upon completion (as measured by TLC (R_f=0.44; SiO_2, eluent; DCM–methanol— 9:1). Then, the reaction mixture was quenched with water (30 mL), and extracted with EtOAc (3x30 mL). The organic layer was washed with brine (100 mL), dried ($MgSO_4$), filtered, and then concentrated in vacuo. The product was separated via a flash column (SiO_2, eluent; DCM–methanol—9:1) into a yellow oily compound (0.032 g, 14%).

^{1}H NMR (400 MHz, Chloroform-*d*) δ 7.16 – 7.10 (m, 2H, Ar-H), 7.03 (d, *J* = 8.0 Hz, 1H, Ar-H), 6.93 – 6.75 (m, 4H, Ar-H), 3.84 (t, *J* = 7.0 Hz, 2H, C1'-H), 2.57 – 2.38 (m, 9H, C5'-H & C6'-H &C2a-H), 1.81 (p, *J* = 7.2 Hz, 2H, C2'-H), 1.53 – 1.37 (m, 4H, C3'-H & C4'-H), 1.01 (t, *J* = 7.2 Hz, 6H, C7'-H).

^{13}C NMR (101 MHz, $CDCl_3$) δ 145.9 (C10a), 145.1 (C9a), 137.6 (C2), 127.7 (C8), 127.6 (C4), 127.3 (C6), 125.2 (C5a), 122.7 (C4a), 122.2 (C3), 120.9 (C7), 115.8 (C1), 114.7 (C9), 52.8 (C5'), 47.4 (C6'), 46.9 (C1'), 26.9 (C2'), 26.5 (C3'), 25.1 (C4'), 16.64 (C2a), 11.5 (C7').

LRMS: m/z (rel. abundance) 387.19 (M^+H, 100%), 388.19 (30%), 389.19 (15%), 390.19 (5%).

HRMS. *m/z* (ESI+) calculated for $C_{22}H_{31}N_2S_2$, 387.1923 $[M+H]^+$, found: 387.1929.

2-(4-(5-(2-(Methylthio)-10*H*-phenothiazin-10-yl)pentyl)piperazin-1-yl)ethan-1-ol (8)

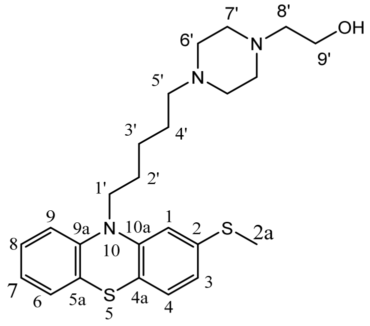

To 2-methylthio-10-(5-chloropenyl)-10*H*-phenothiazine (0.2 g, 0.572 mmol) in anhydrous acetone (12 mL) was added sodium iodide (0.103 g, 0.686 mmol), potassium carbonate (0.095 g, 0.686 mmol), and diethylamine (0.070 mL, 0.686 mmol). The reaction mixture was refluxed for 52 h upon completion (as measured by TLC (R_f=0.50, SiO_2, eluent - DCM:methanol – 9:1)). Then, the reaction mixture was quenched with water (30 mL), and extracted with EtOAc (3x30 mL). The organic layer was washed with brine (100 mL), dried ($MgSO_4$), filtered, and then concentrated in vacuo. The product was separated via a flash column (SiO_2, eluent; DCM–methanol— 9:1) into a yellow oily compound (0.052 g, 20%).

^{1}H NMR (400 MHz, Chloroform-*d*) δ 7.16 – 7.09 (m, 2H, Ar - H), 7.03 (d, *J* = 8.0 Hz, 1H, Ar - H), 6.90 (td, *J* = 7.5, 1.2 Hz, 1H, Ar - H), 6.85 – 6.78 (m, 2H, Ar - H), 6.76 (d, *J* = 1.8 Hz, 1H, Ar-H), 3.84 (t, *J* = 7.0 Hz, 2H, C1'-H), 3.60 (t, *J* = 5.4 Hz, 2H, C5'- H), 2.64 – 2.36 (m, 14H, C6'&C7'&C9'&C2a - H, OH), 2.33 – 2.27 (m, 2H, C8'-H), 1.80 (p, *J* = 7.1 Hz, 2H, C2'-H), 1.55 – 1.38 (m, 4H, C3'&C4' - H).

^{13}C NMR (101 MHz, $CDCl_3$) δ 145.9 (C10a), 145.1 (C9a), 137.6 (C2), 127.7 (C8), 127.6 (C4), 127.3 (C6), 125.3 (C5a), 122.7 (C4a), 122.3 (C3), 120.9 (C7), 115.8 (C1), 114.7 (C9), 59.3 (C8'), 58.5 (C9'), 57.8 (C7'), 53.3 (C6'), 52.9 (C5'), 47.3 (C1'), 26.8 (C4'), 26.6 (C3'), 24.9 (C2'), 16.63 (C2a).

LRMS: m/z (rel. abundance) 444.21 (M+H, 100%), 445.21 (30%), 446.21 (15%), 447.21 (5%).

HRMS. *m/z* (ESI+) calculated for $C_{24}H_{34}N_3OS_2$, 444.2138 $[M+H]^+$, found: 444.2143

Electrosynthesis of Metabolites

2CPTZ

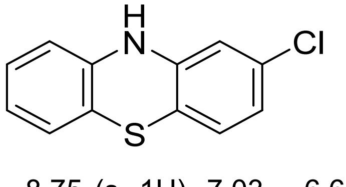

^{1}H NMR (400 MHz, DMSO-*d*$_6$) δ_H 8.75 (s, 1H), 7.03 – 6.65 (m, 7H). **^{13}C NMR** (101 MHz, DMSO-*d*$_6$) δ_C 143.49, 141.07, 131.77, 127.77, 127.42, 126.32, 122.33, 121.19, 116.02, 115.49, 114.66, 113.71.

2CPTZ-SO

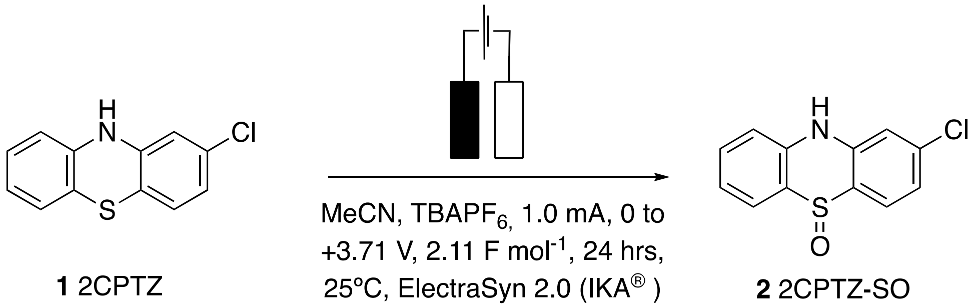

Following general procedure D, using **13** (100 mg, 0.43 mmol), $TBAPF_6$ (829 mg, 2.14 mmol), and workup procedure E yielded the title compound (54 mg, 0.22 mmol, 51%) as a white solid. **M.P** 215.2-216.3 °C. **^{1}H NMR** (400 MHz, DMSO-*d*$_6$) δ_H 11.09 (s, 1H), 8.12 – 7.14 (m, 7H). **^{13}C NMR** (101 MHz, DMSO-*d*$_6$) δ_C 137.90, 136.95, 136.14, 133.23, 133.00, 131.17, 121.87, 121.16, 119.85, 116.98, 115.96. **LRMS** (ES^+) *m/z* 250.01 ($[M+H]^+$, 100%). **HRMS** (EI^+) *m/z* $C_{12}H_8ClNOS$ requires 249.0010, found 249.0012 ($[M]^+$).

2CPTZ-SO_2

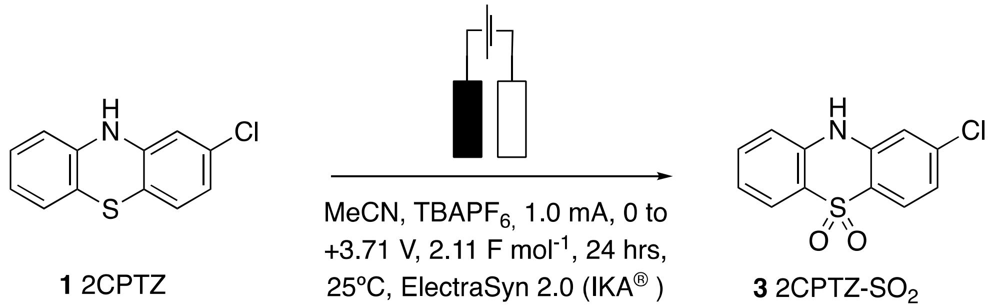

Following general procedure D, using **13** (100 mg, 0.43 mmol), $TBAPF_6$ (829 mg, 2.14 mmol), and workup procedure E yielded the title compound (4 mg, 0.02 mmol, 4%) as a pink solid. **^{1}H NMR** (400 MHz, DMSO-*d*$_6$) δ_H 11.06 (s, 1H), 8.01 – 7.22 (m, 7H). **^{13}C NMR** (101 MHz, DMSO-*d*$_6$) δ_C139.76, 138.23, 138.09, 134.06, 125.30, 122.96, 122.57, 122.04, 121.48, 120.02, 117.73, 116.68. **LRMS** (ES^+) *m/z* 265.01 ($[M+H]^+$, 100%). **HRMS** (EI^-) *m/z* $C_{12}H_8ClNO_2S$ requires 263.9886, found 263.9869 ($[M]^-$)

Tetrabutylammonium hexafluorophosphate ($TBAPF_6$)

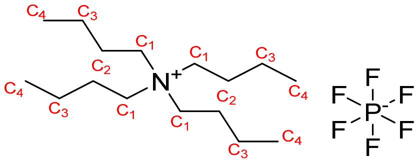

^{1}H NMR (400 MHz, Chloroform-d) δ_H 3.18 – 3.11 (m, 8H, C_1-H), 1.59 (tt, *J* = 8.1, 6.2 Hz, 8H, C_2-H), 1.41 (h, *J* = 7.3, 8.1 Hz, 8H, C_3-H), 0.98 (t, *J* = 7.3, 6.2 Hz, 12H, C_4-H). **^{13}C NMR** (101 MHz, Chloroform-d) δ_C 58.6 C_1, 23.8 C_2, 19.7 C_3, 13.6 C_4.

Chlorpromazine (CPZ)

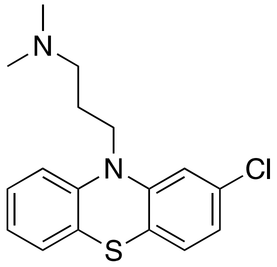

^{1}H NMR (400 MHz, DMSO-d_6) δ_H 7.34 – 6.96 (m, 7H), 3.99 (t, *J* = 7.1 Hz, 2H), 3.17 – 3.08 (m, 2H), 2.68 (s, 6H), 2.13 – 2.01 (m, 2H). **^{13}C NMR** (101 MHz, DMSO) δ_C 146.17, 143.71, 132.61, 128.22, 127.89, 127.36, 123.69, 123.27, 122.93, 122.44, 116.46, 115.91, 54.16, 43.94, 41.95, 21.44.

CPZ-SO

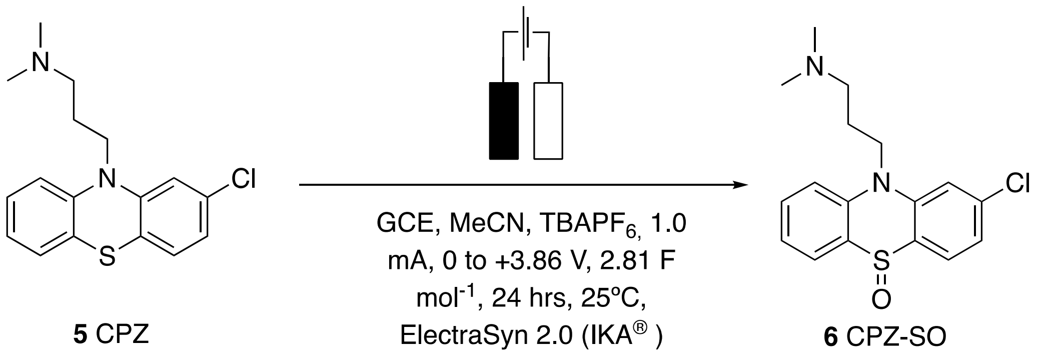

5 CPZ **6** CPZ-SO

Following the general electrosynthesis procedure G, using **16** (100 mg, 0.31 mmol), $TBAPF_6$ (608 mg, 1.57 mmol), and the work-up procedures (B and H) afforded the title compound (71 mg, 0.21 mmol, 68%) as a brown solid. **M.P** 157-161°C. **^{1}H NMR** (400 MHz, DMSO-d_6) δ_H 8.13 – 7.16 (m, 7H), 4.41 (t, J = 7.4 Hz, 2H), 2.70 (td, J = 7.1, 3.0 Hz, 2H), 2.38 (s, 6H), 2.09 – 1.92 (m, 2H).**^{13}C NMR** (101 MHz, DMSO) δ_C 139.1, 137.7, 137.4, 133.2, 132.8, 131.1, 124.6, 123.2, 122.4, 121.7, 116.87, 116.3, 54.7, 44.5, 43.8, 22.7. **LRMS** (ES$^+$) *m/z* 335.10 ([M+H]$^+$, 100%). **HRMS** (EI$^+$) *m/z* $C_{17}H_{19}ClN_2OS$ requires 334.0912, a found 334.0904 ([M]$^+$).

NMR and MS Data

^{1}H NMR of 2-chloro-10-(6-chlorohexyl)-10H-phenothiazine

(400 MHz, Chloroform-*d*).

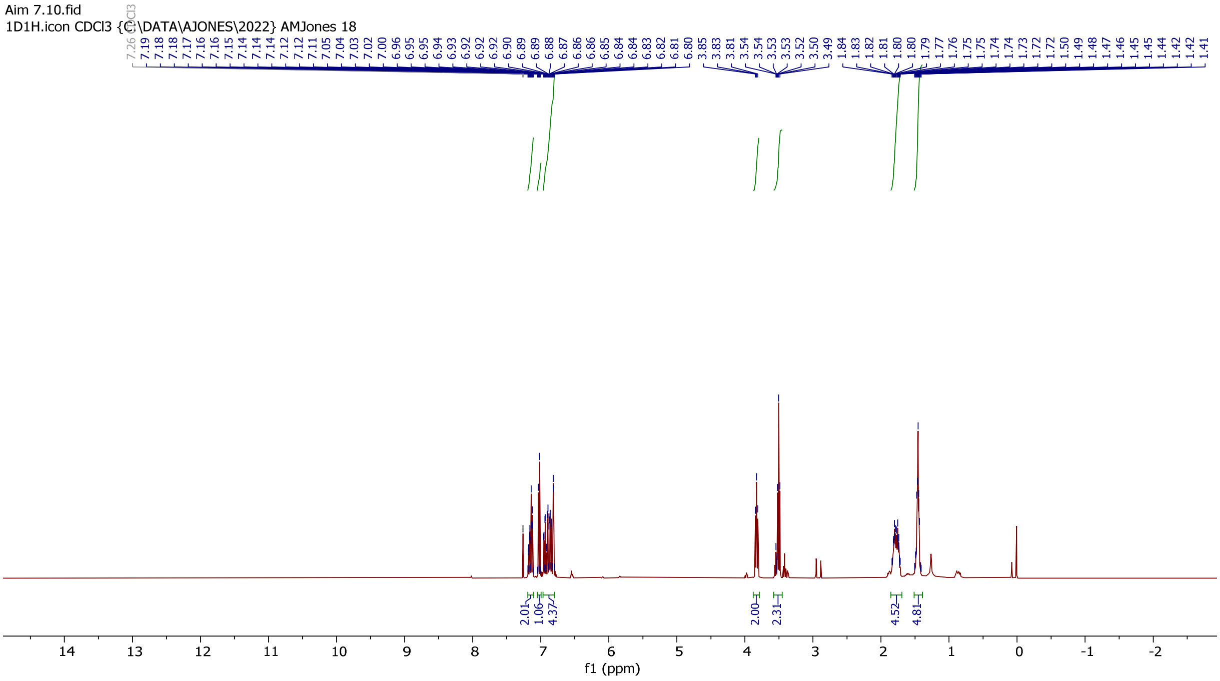

^{13}C NMR of 2-chloro-10-(6-chlorohexyl)-10H-phenothiazine

(101 MHz, $CDCl_3$)

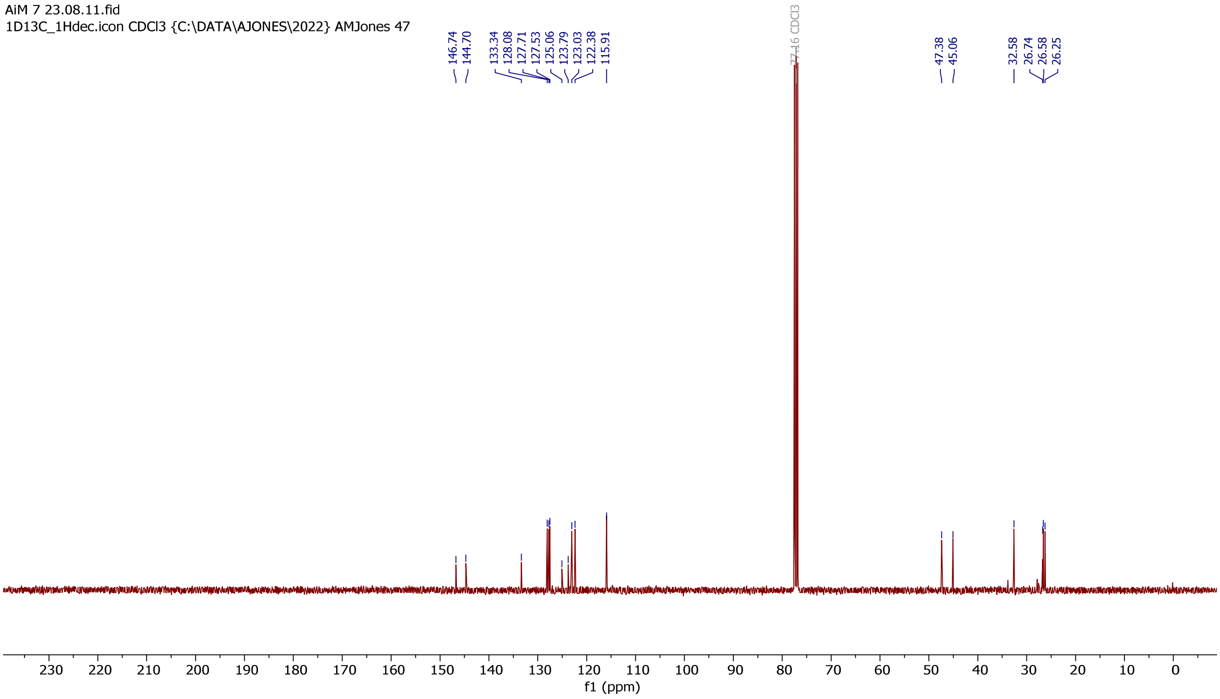

^{1}H NMR spectrum of 1 (400 MHz, Chloroform-*d*)

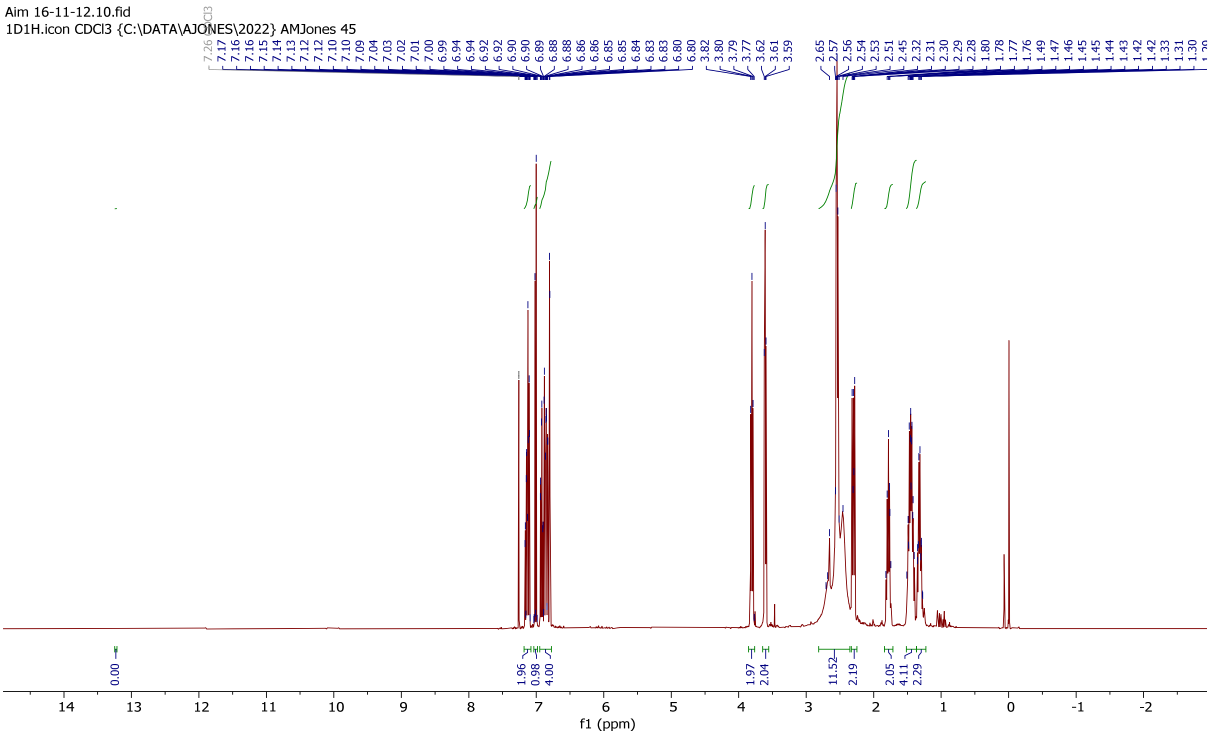

^{13}C NMR spectrum of 1 (101 MHz, $CDCl_3$)

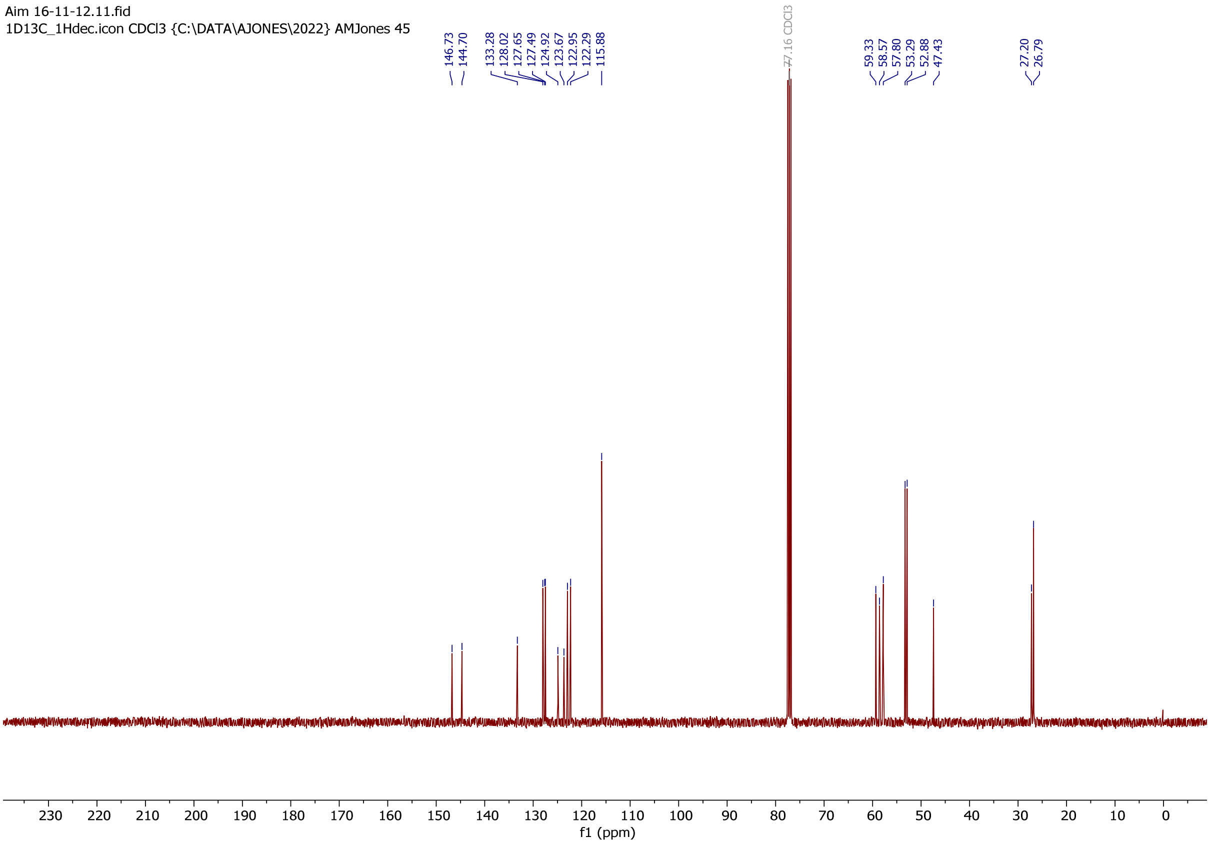

^{1}H NMR spectrum of **2** (400 MHz, Chloroform-d)

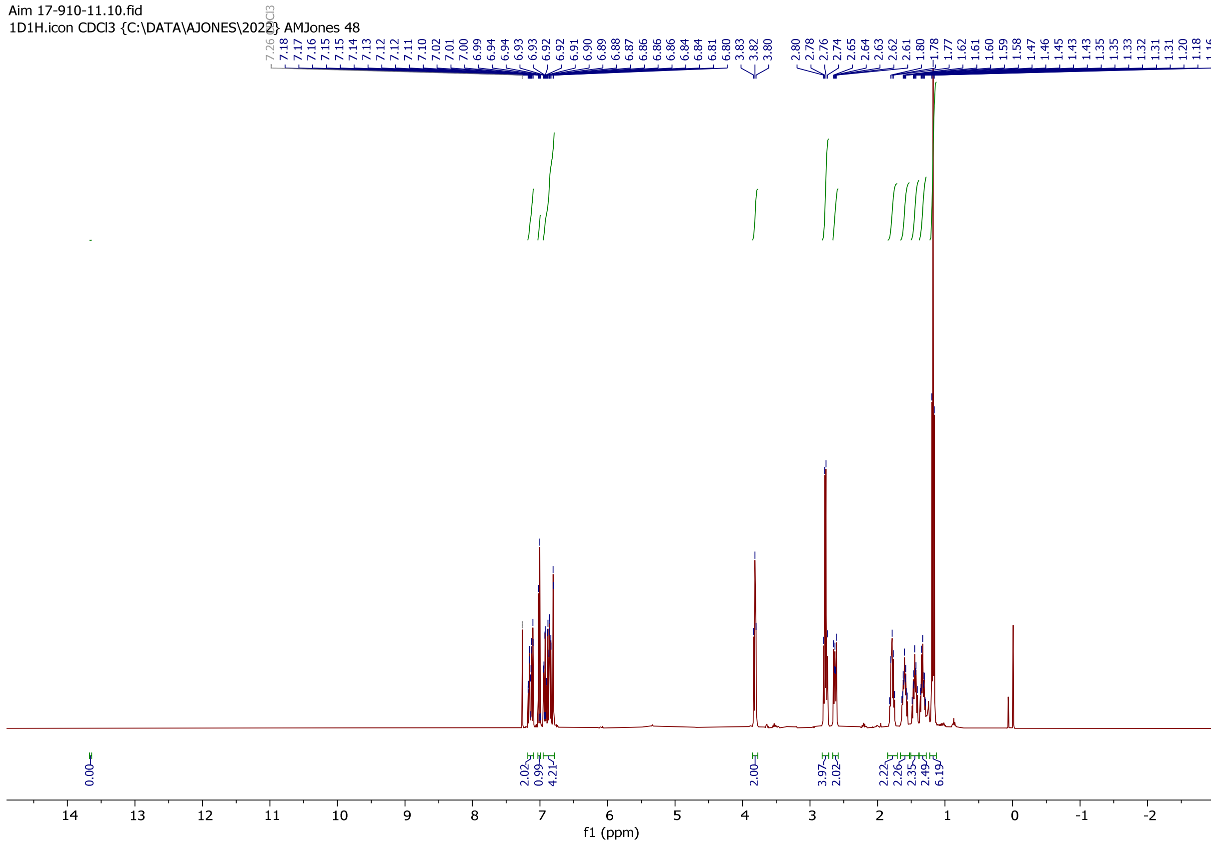

^{13}C NMR of 2 (101 MHz, $CDCl_3$)

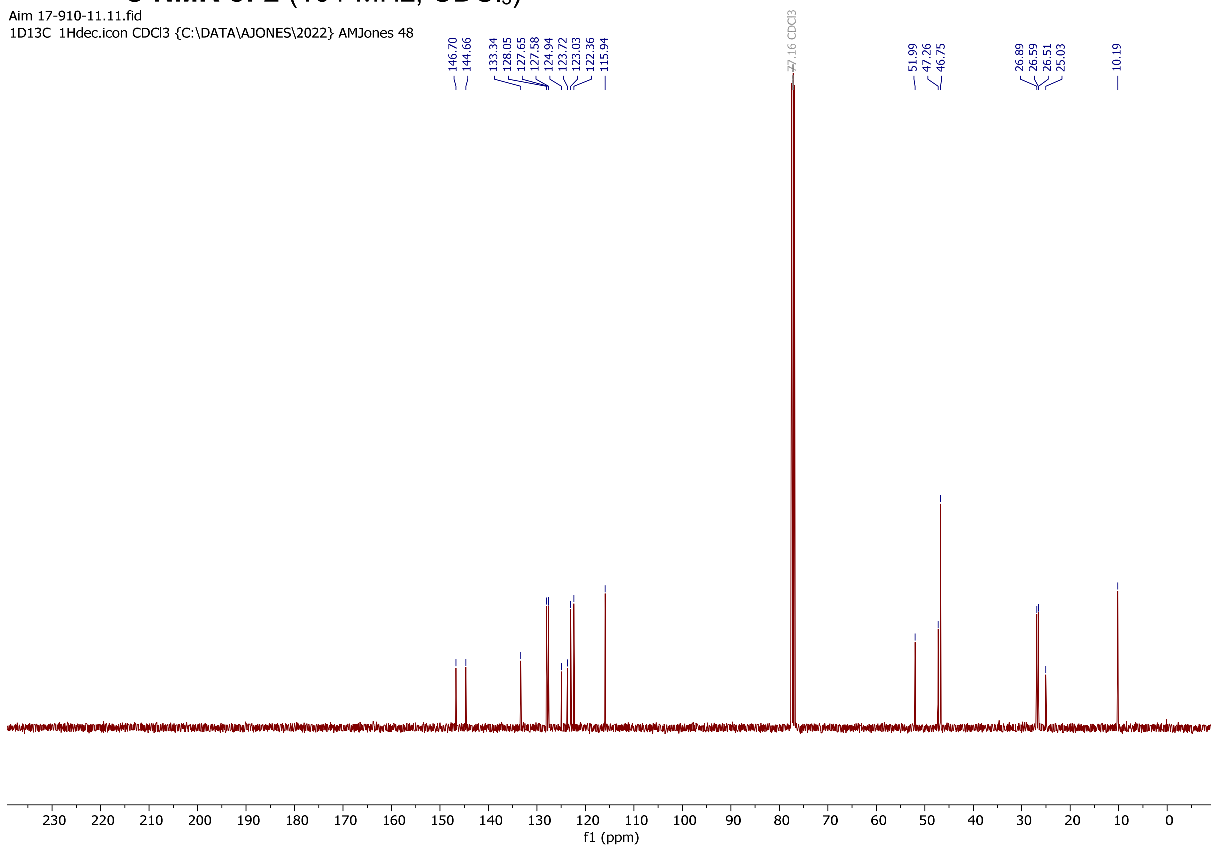

^{1}H NMR spectrum of 3 (400 MHz, Chloroform-*d*)

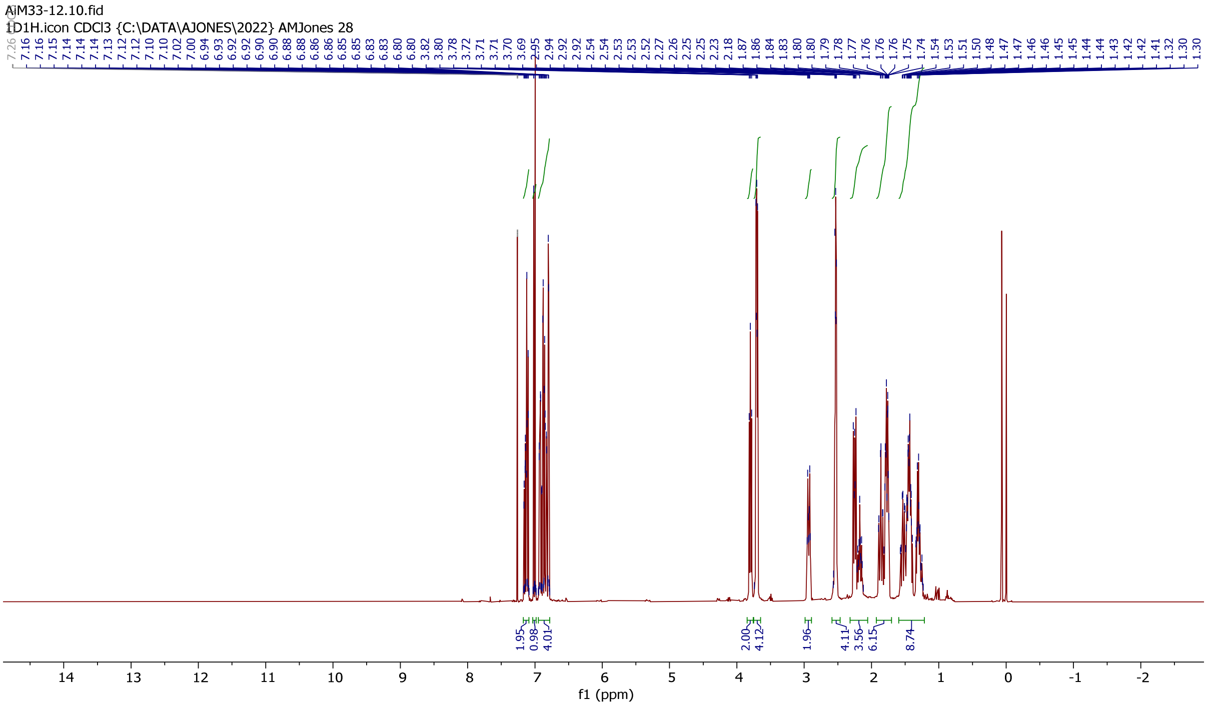

^{13}C NMR spectrum of 3 (101 MHz, $CDCl_3$)

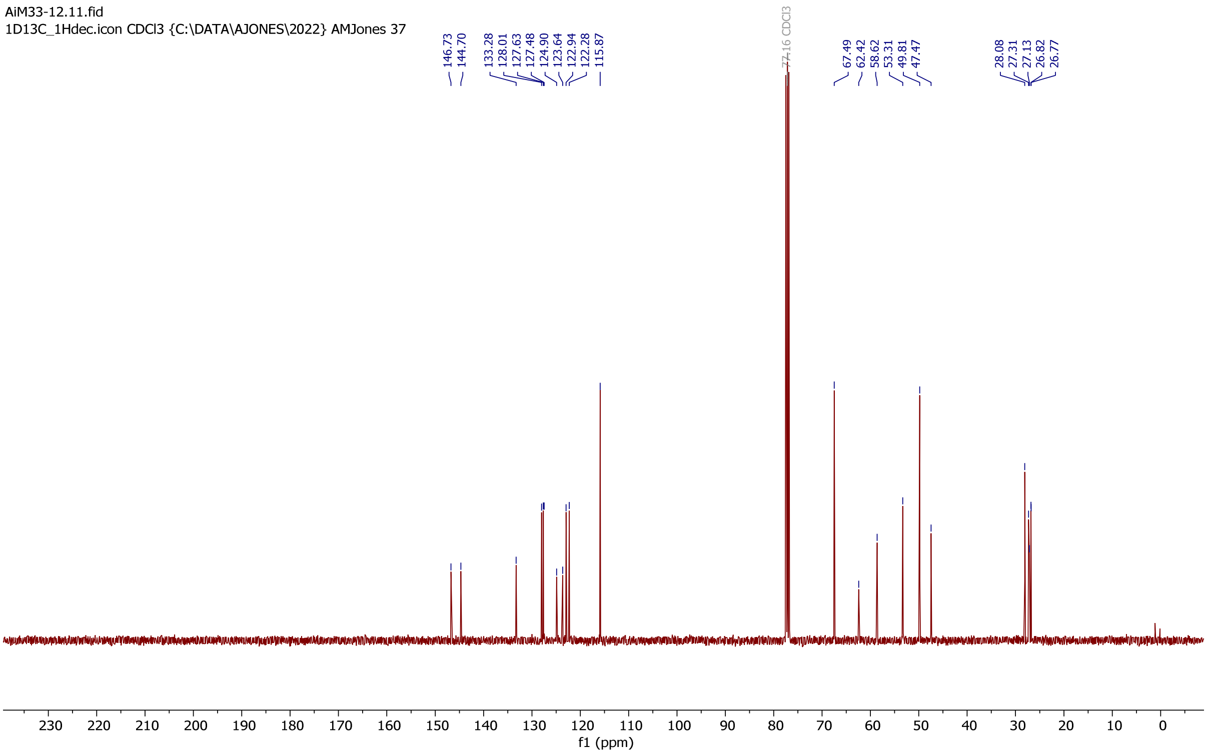

^{1}H NMR spectrum of 2-methylthio-10-(6-chloropentyl)-10H-phenothiazine (400 MHz, Chloroform-*d*) δ

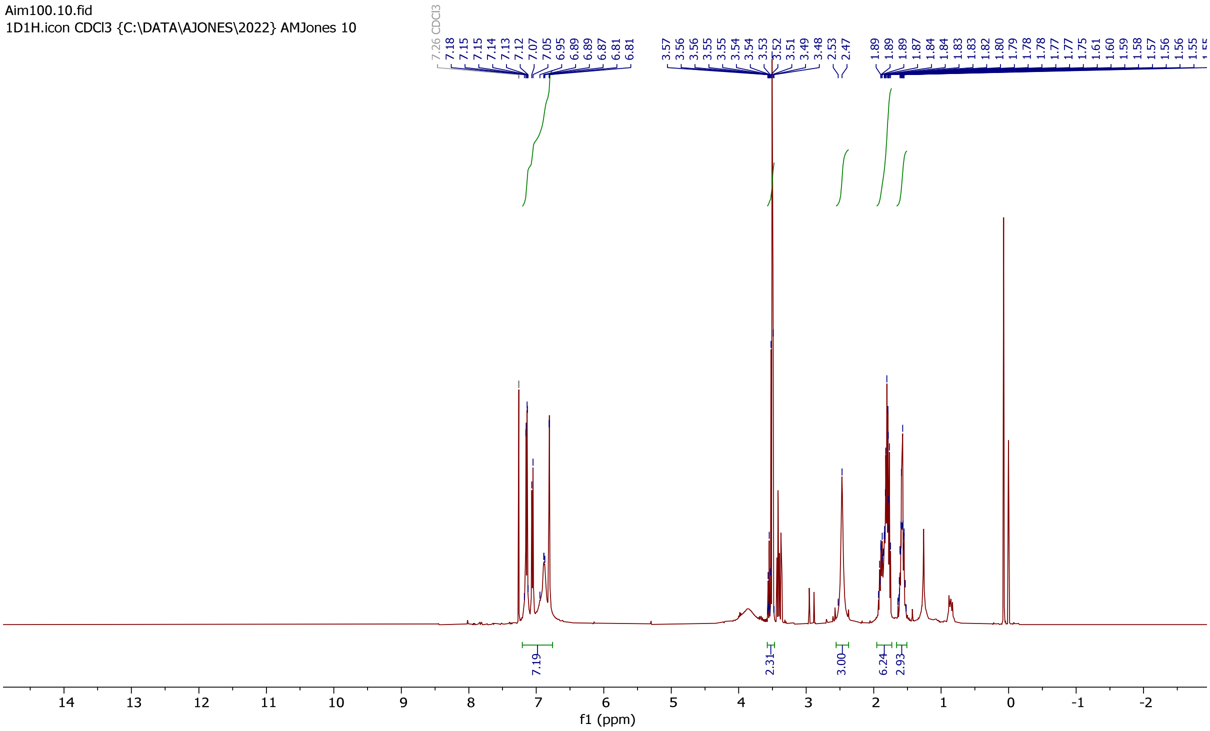

^{13}C NMR spectrum of 2-methylthio-10-(6-chloropentyl)-10H-phenothiazine (101 MHz, $CDCl_3$) δ

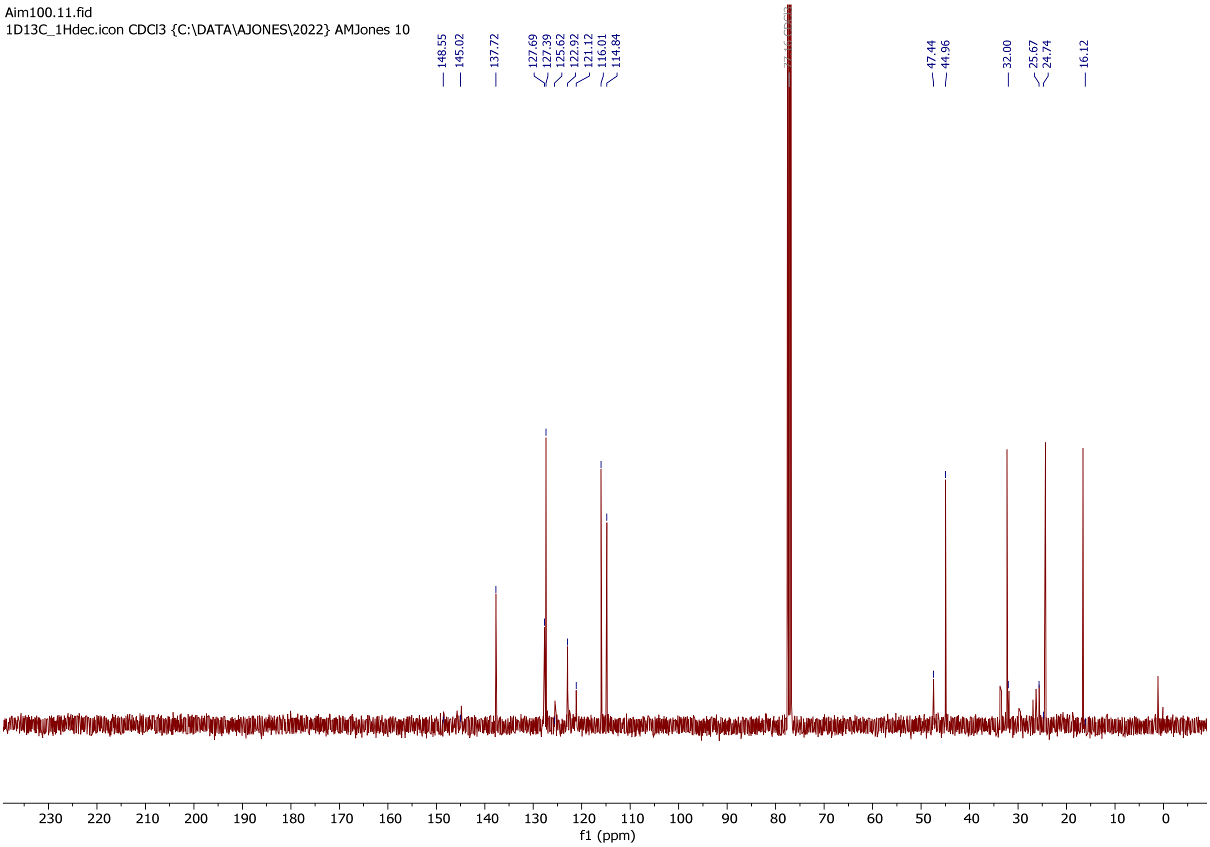

^{1}H NMR spectrum of **4** (400 MHz, Chloroform-*d*)

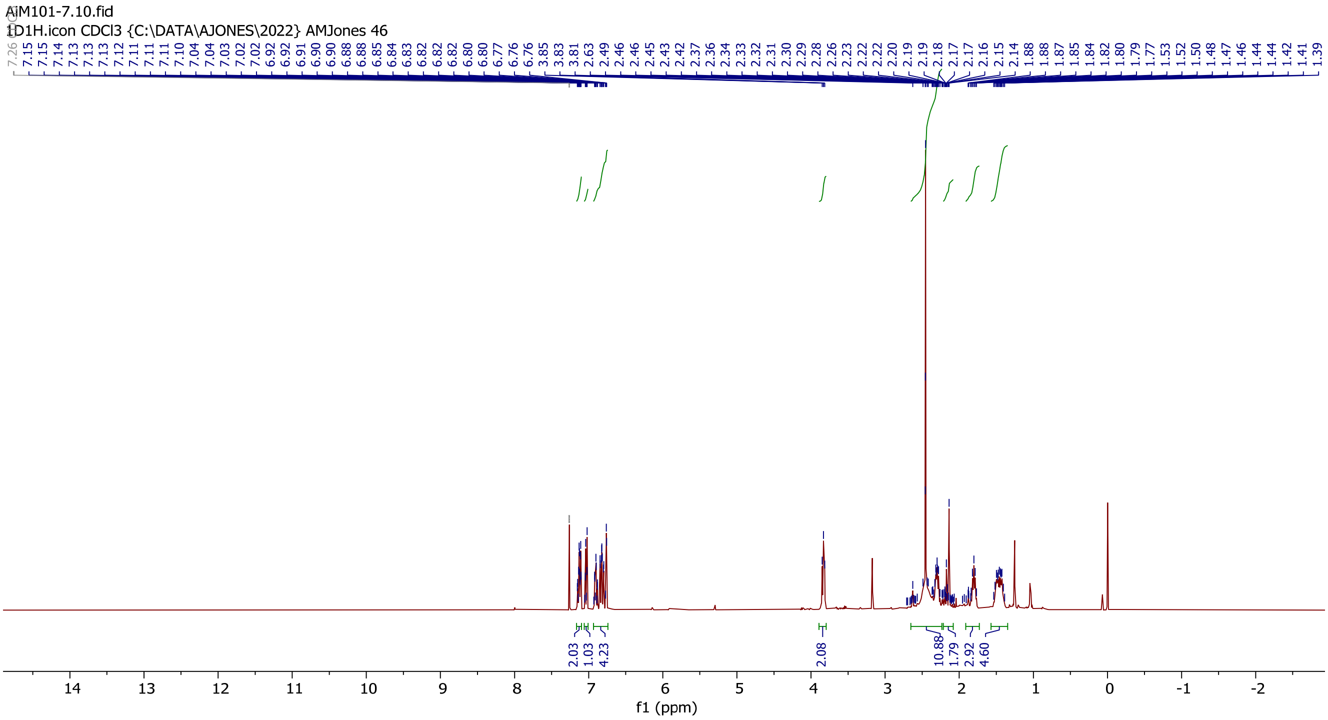

^{13}C NMR spectrum of **4** (101 MHz, $CDCl_3$)

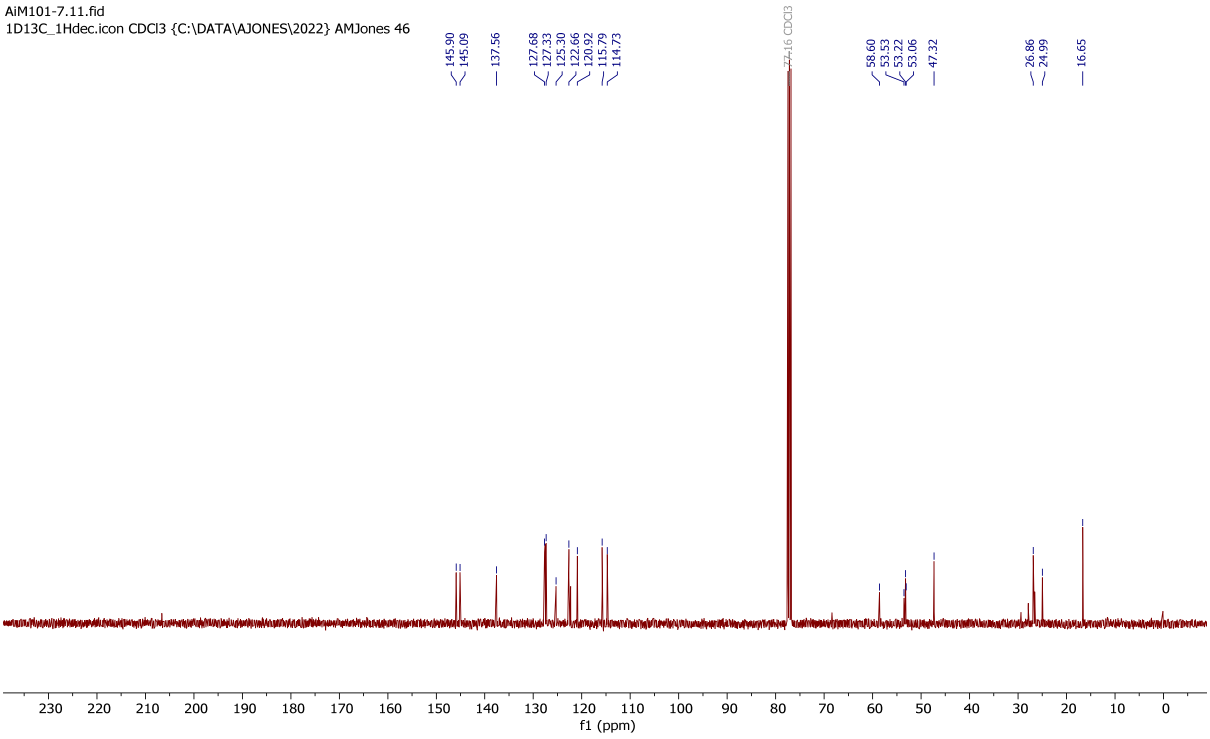

^{1}H NMR spectrum of 5 (400 MHz, Chloroform-d)

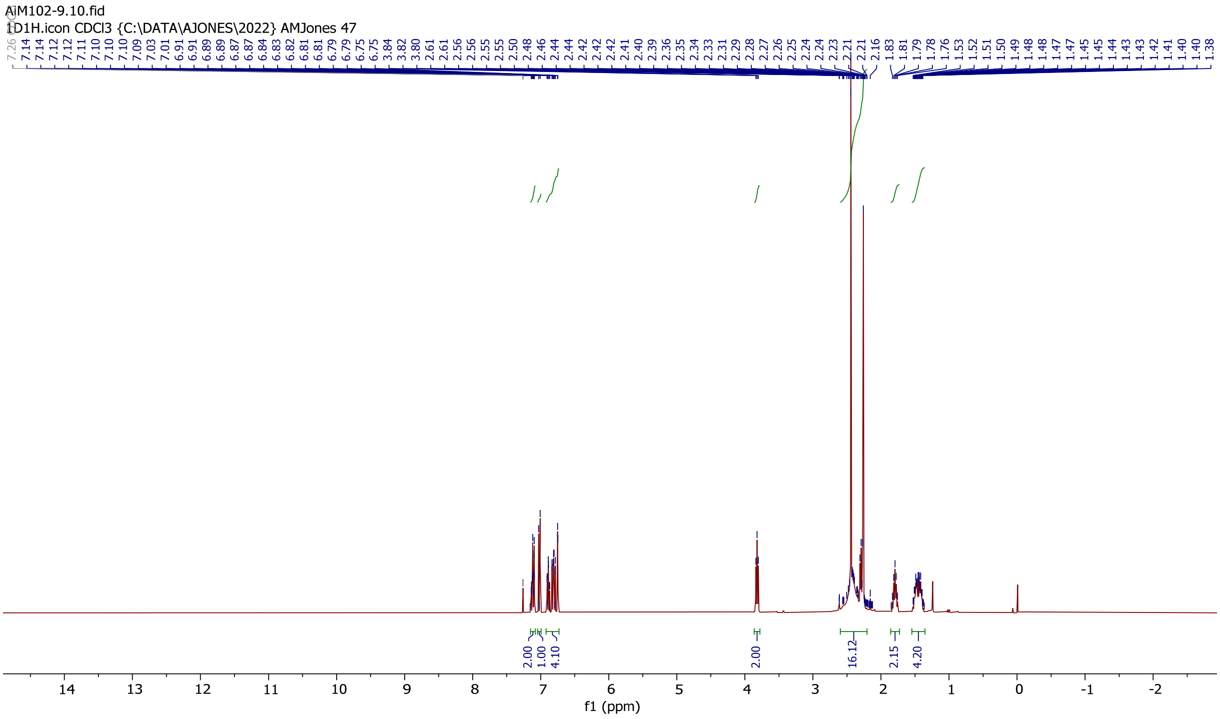

^{13}C NMR spectrum of 5 (101 MHz, $CDCl_3$)

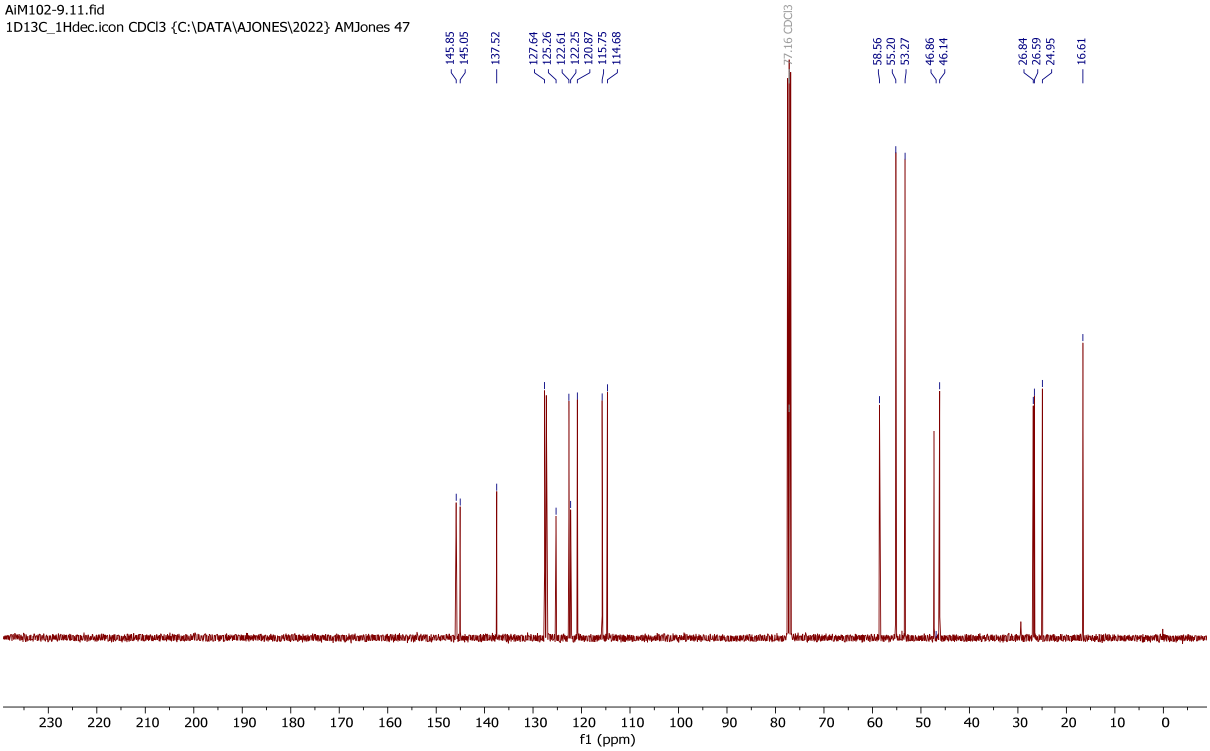

^{1}H NMR spectrum of 6 (400 MHz, Chloroform-*d*)

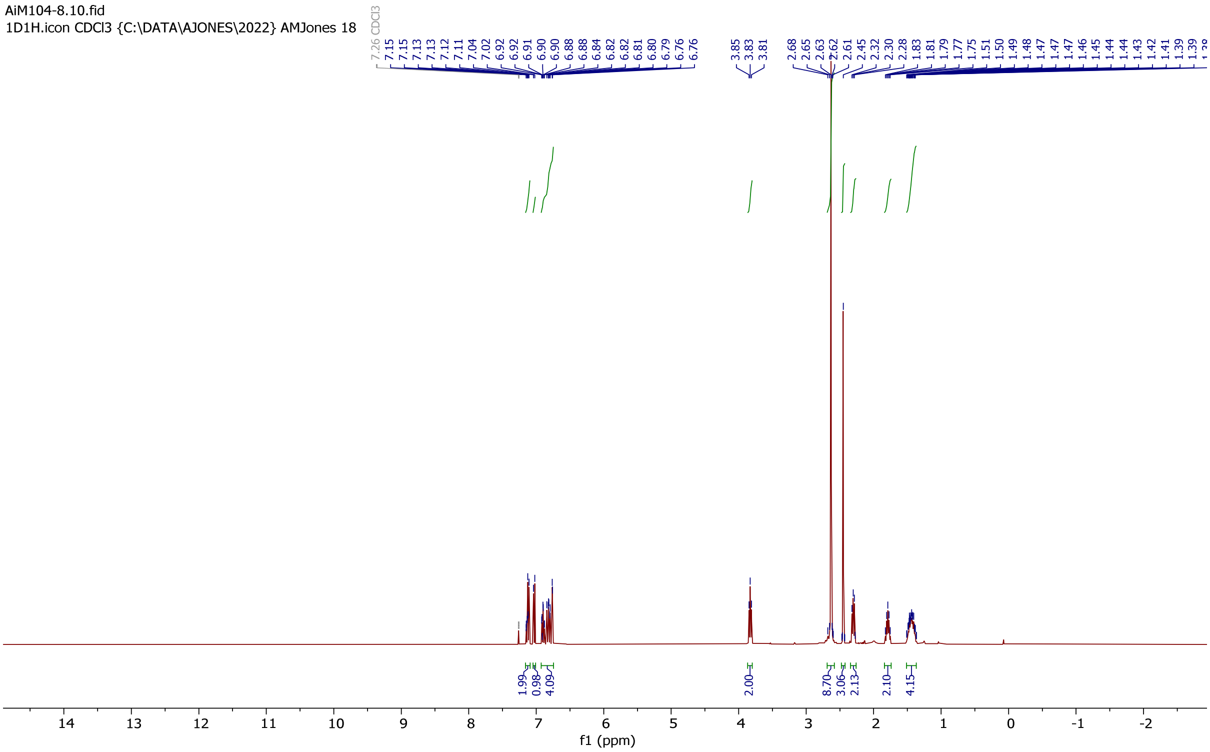

^{13}C NMR spectrum of 6 (101 MHz, $CDCl_3$) δ

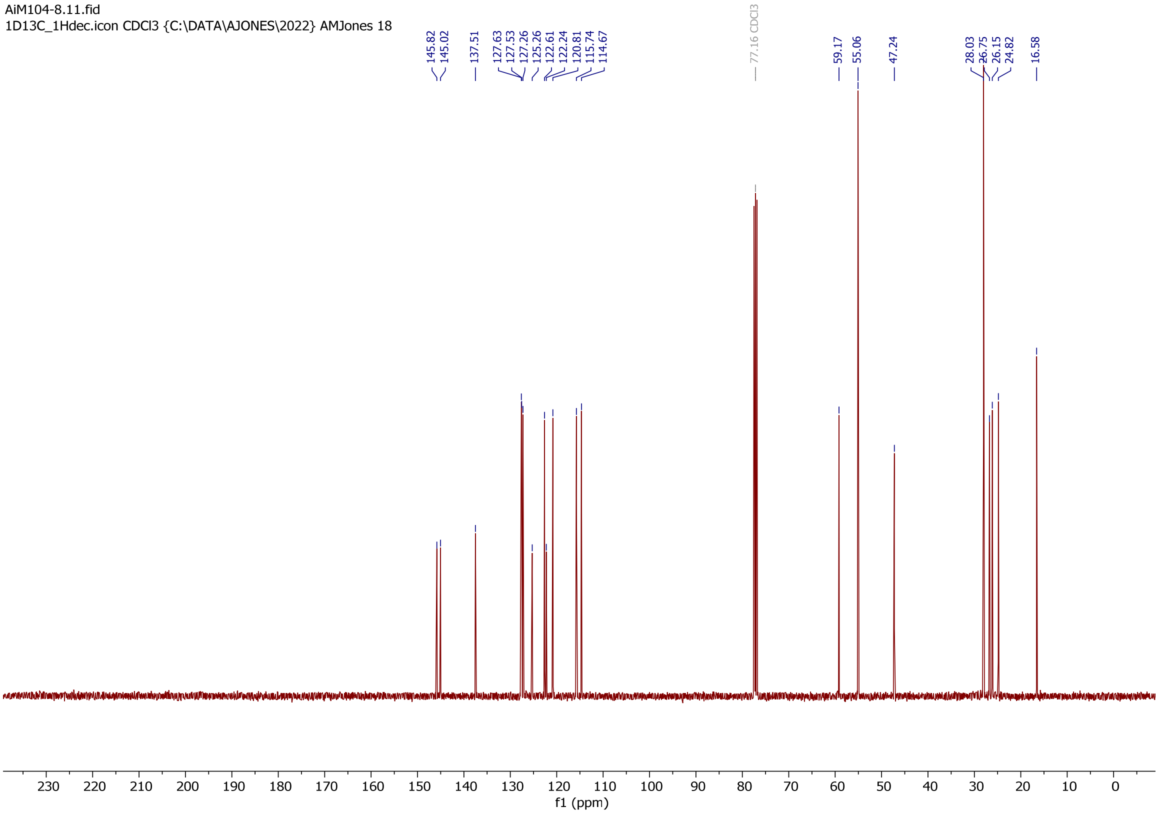

^{1}H NMR spectrum of 7 (400 MHz, Chloroform-*d*)

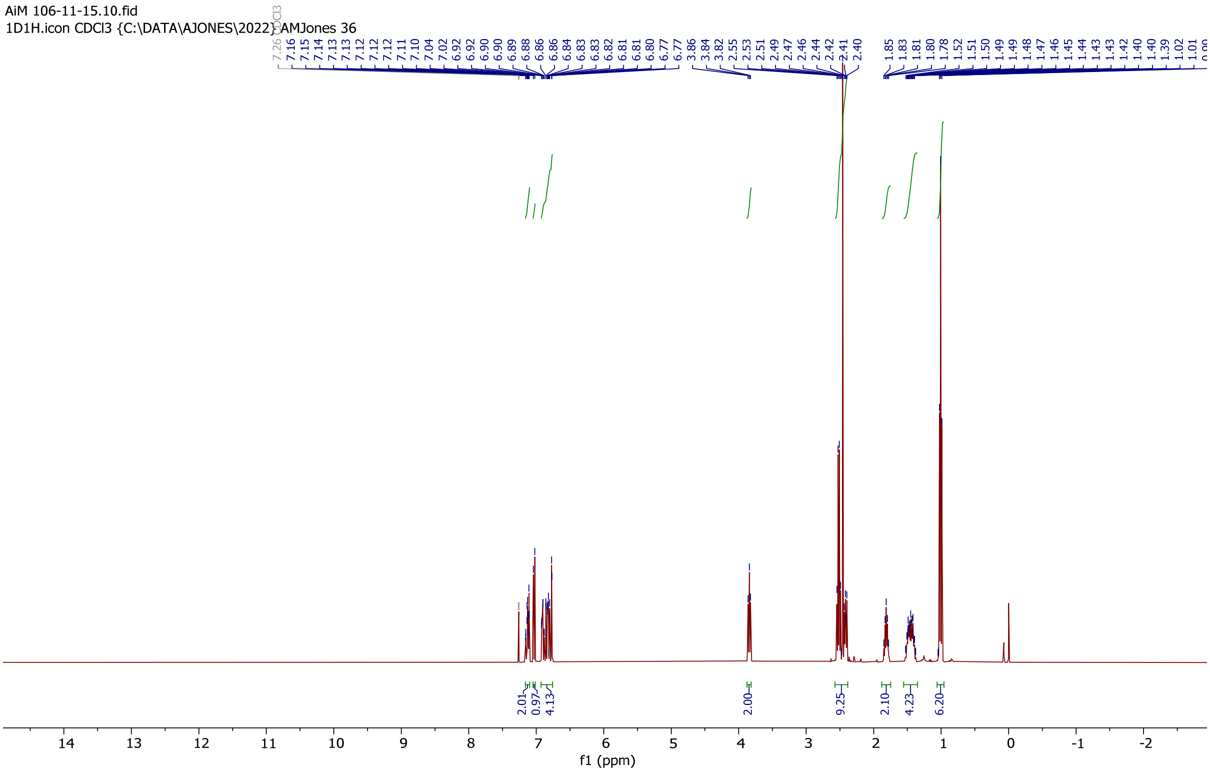

^{13}C NMR spectrum of 7 (101 MHz, $CDCl_3$) δ

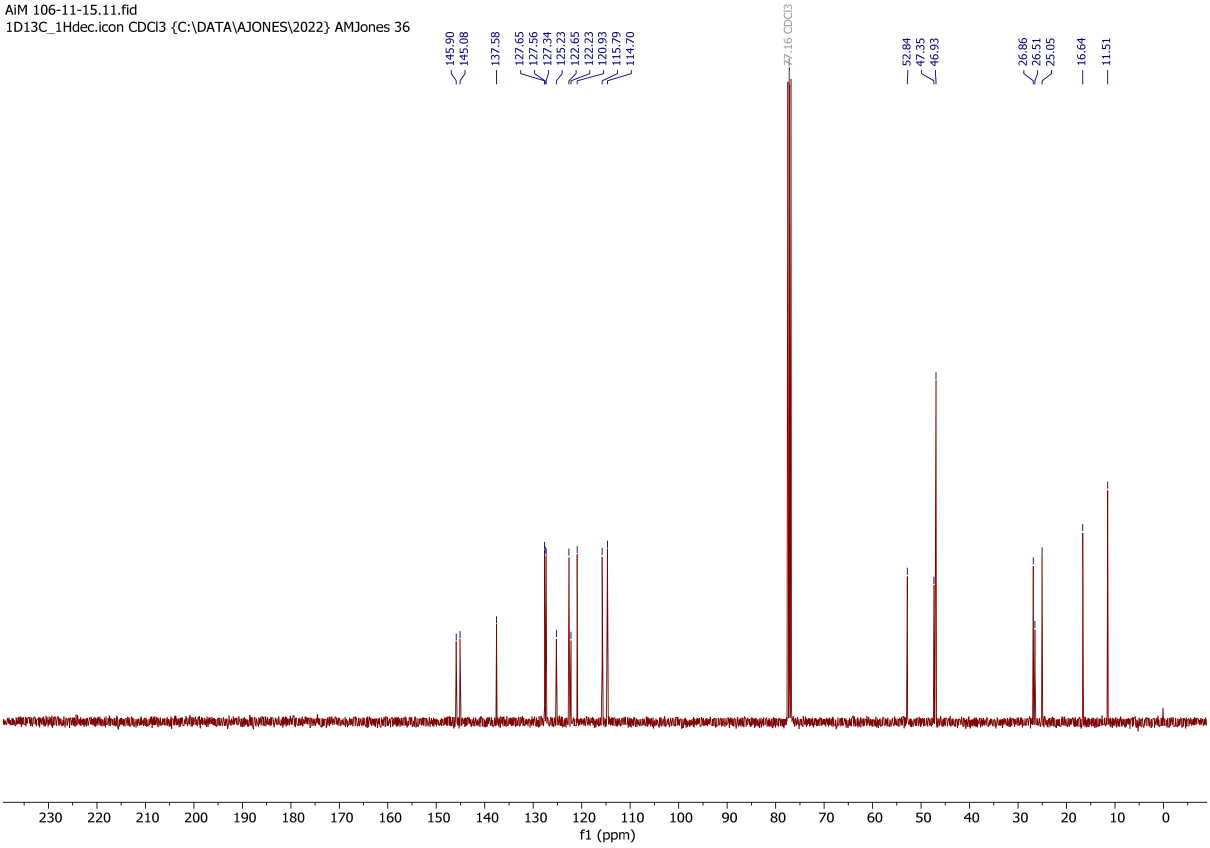

^{1}H NMR spectrum of **8** (400 MHz, Chloroform-*d*)

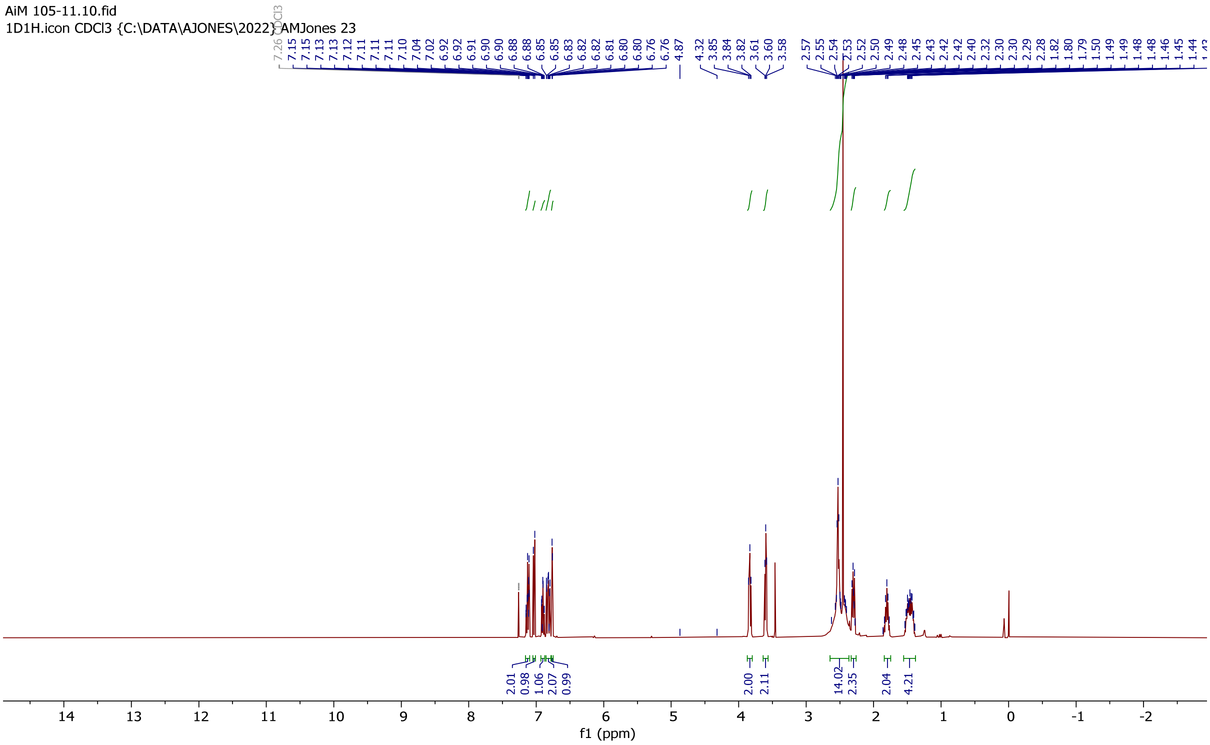

^{13}C NMR spectrum of **8** (101 MHz, $CDCl_3$)

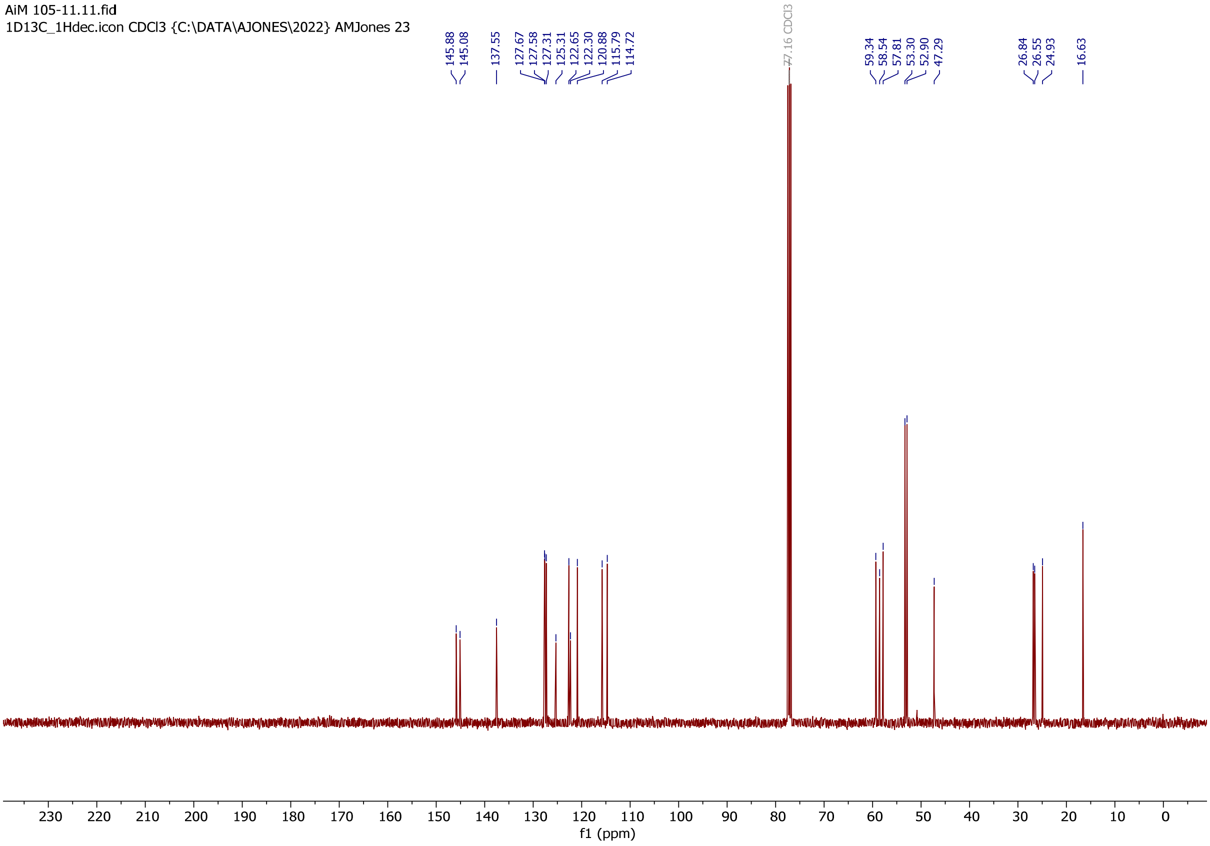

^{1}H-NMR spectrum **2-CPTZ** (DMSO-d_6, 400 MHz)

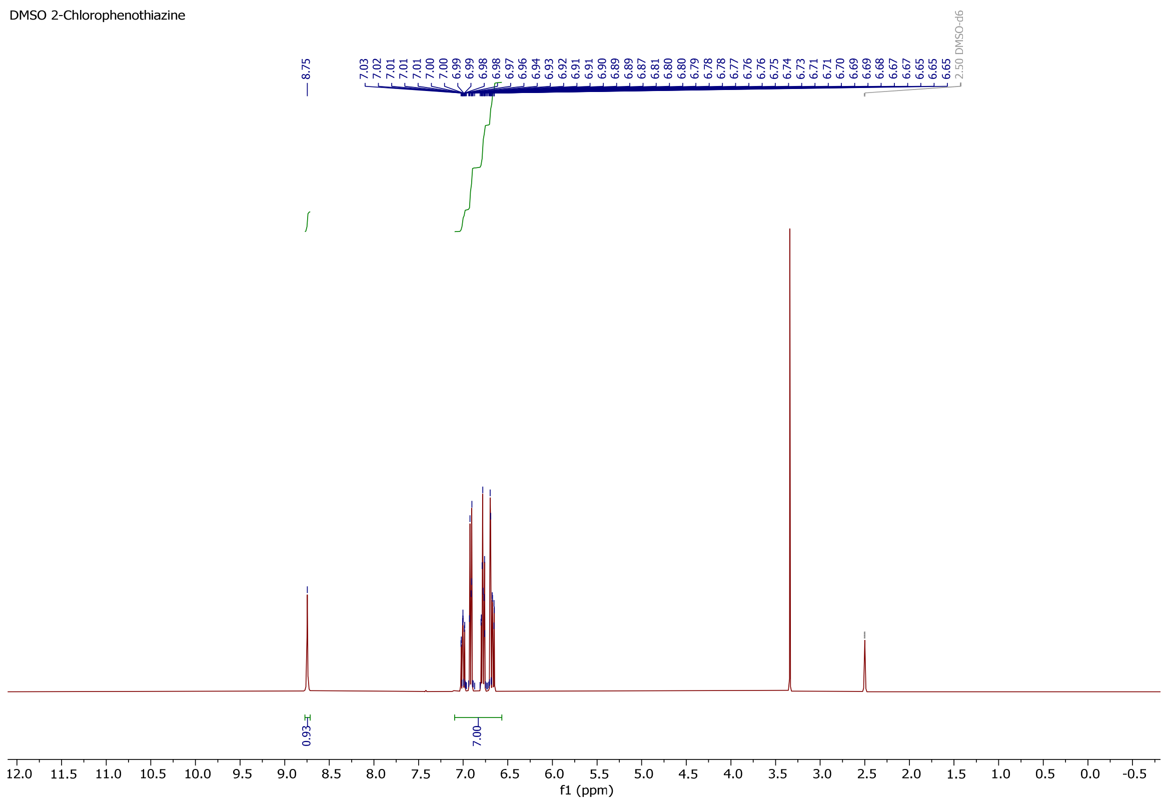

^{13}C-NMR spectrum **2-CPTZ** (DMSO-d_6, 101 MHz)

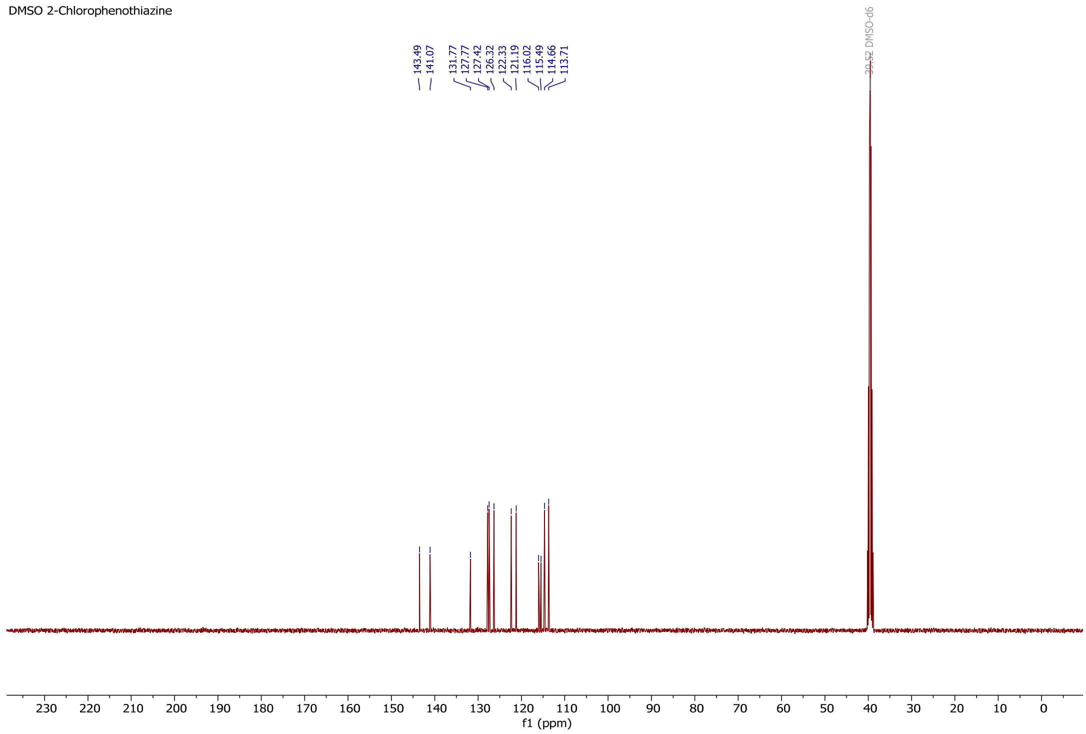

^{1}H-NMR spectrum **2CPTZ-SO** (DMSO-d_6, 400 MHz)

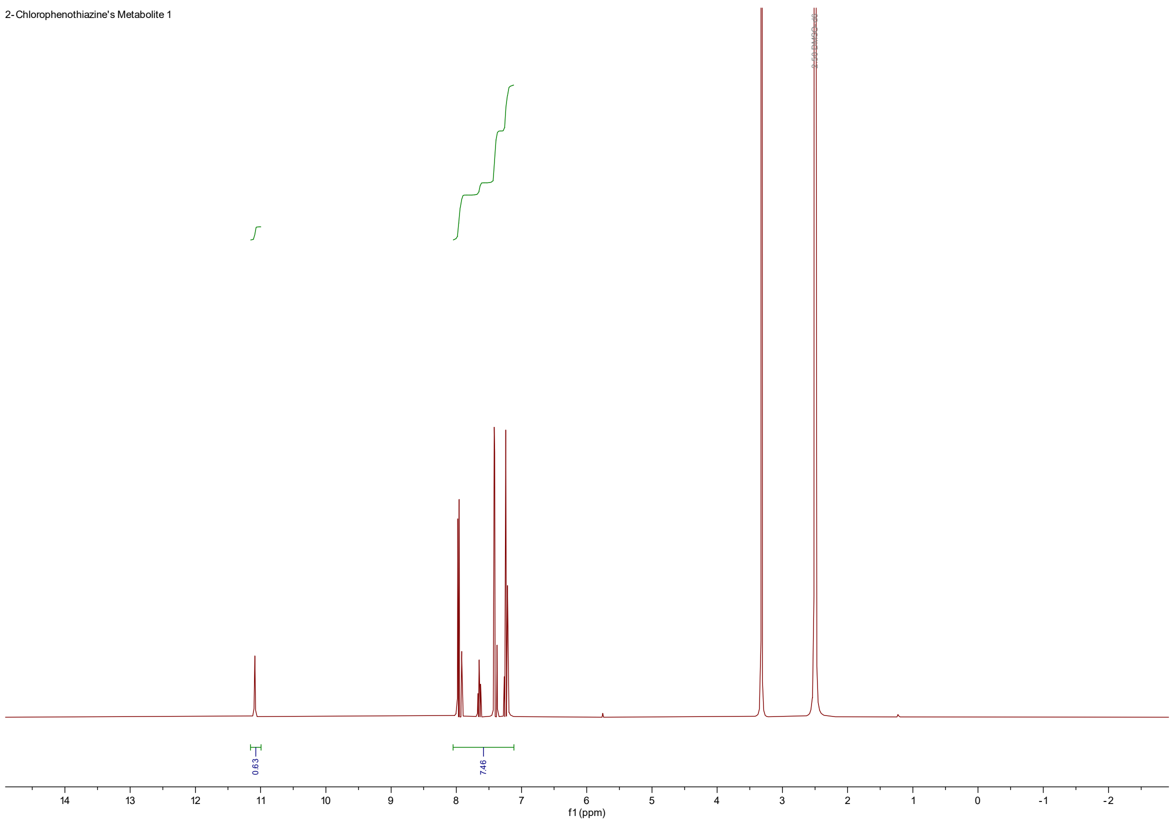

^{13}C-NMR spectrum **2CPTZ-SO** (DMSO-d_6, 101 MHz)

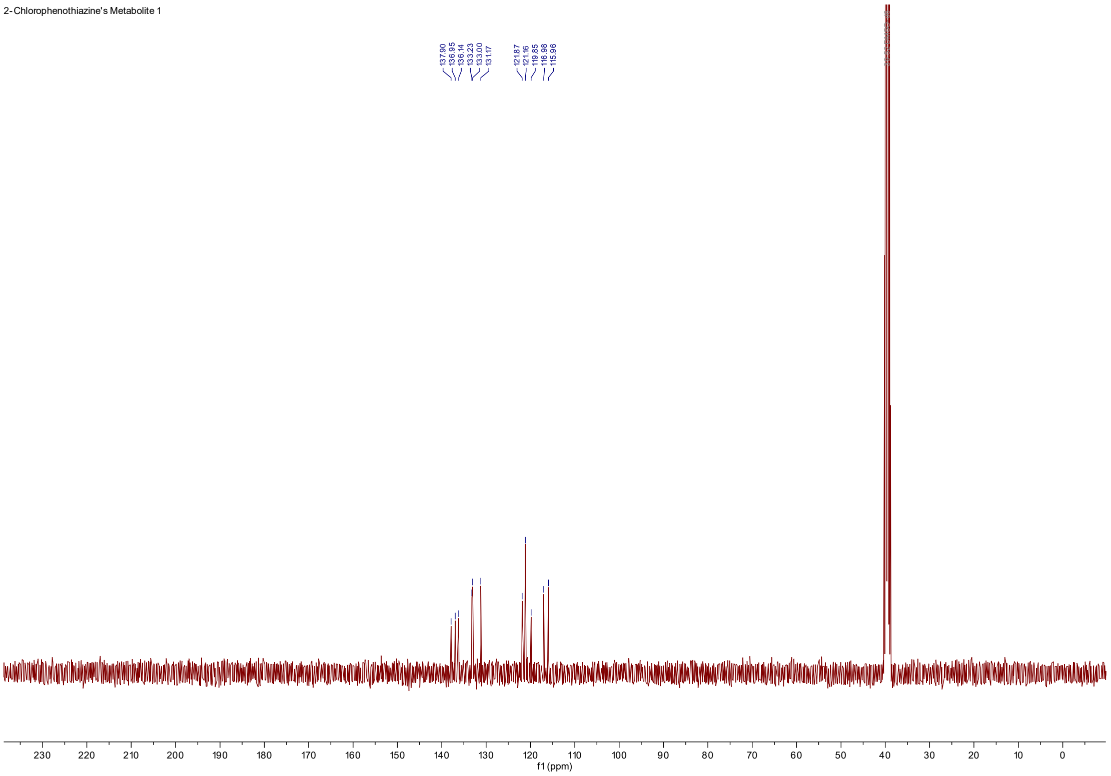

HRMS spectrum **2CPTZ-SO**

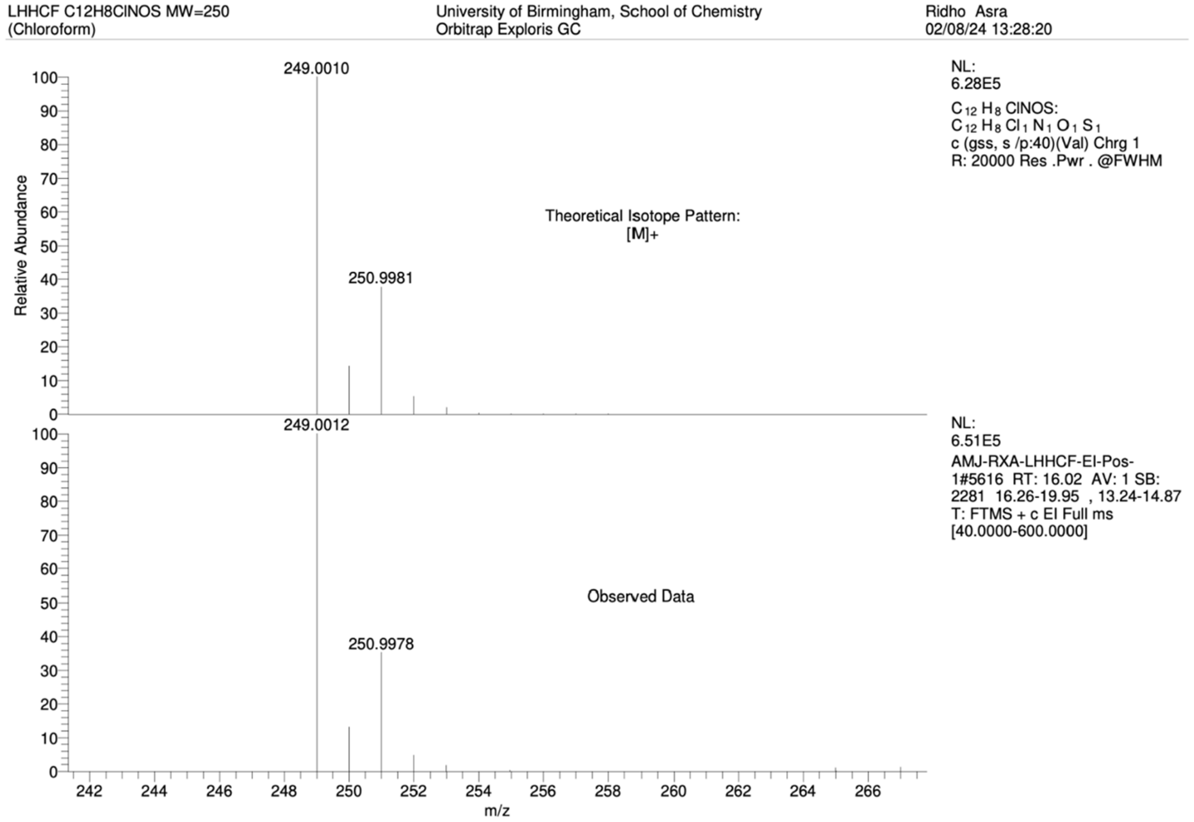

^{1}H-NMR spectrum **2CPTZ-SO$_2$** (DMSO-d_6, 400 MHz)

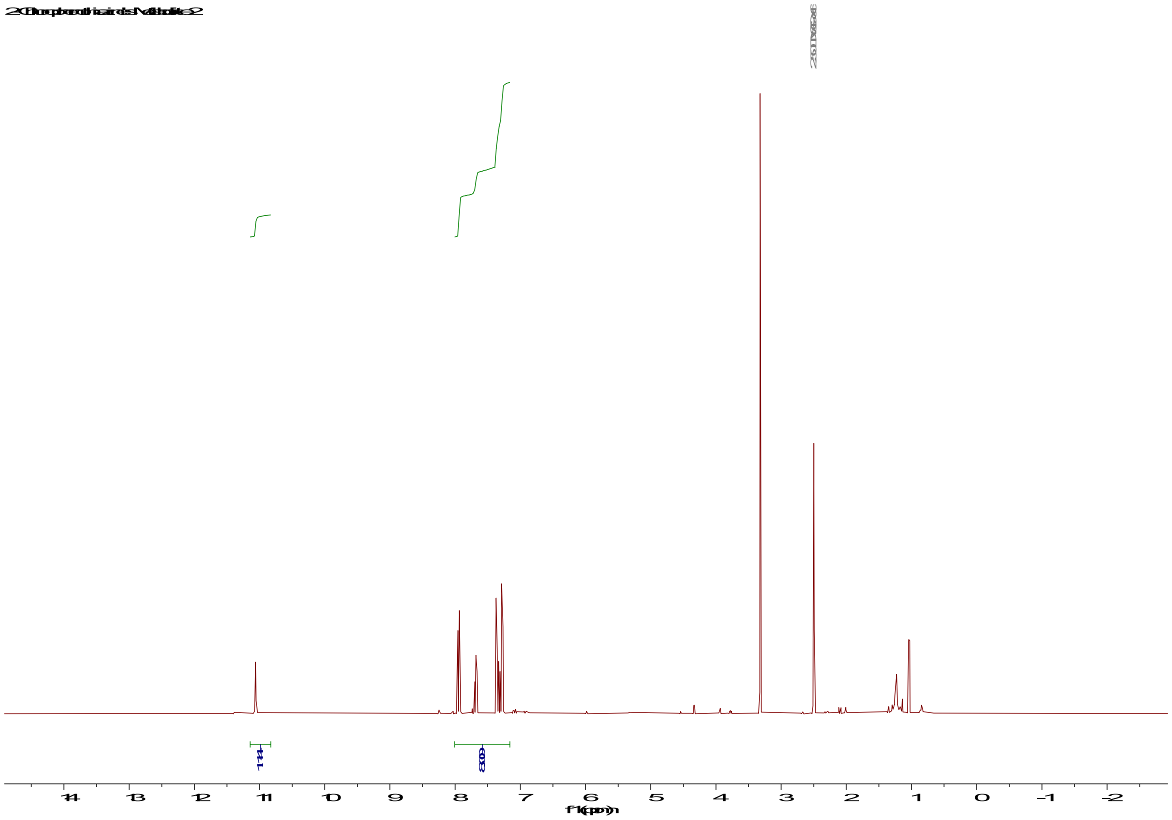

^{13}C-NMR spectrum **2CPTZ-SO$_2$** (DMSO-d_6, 101 MHz)

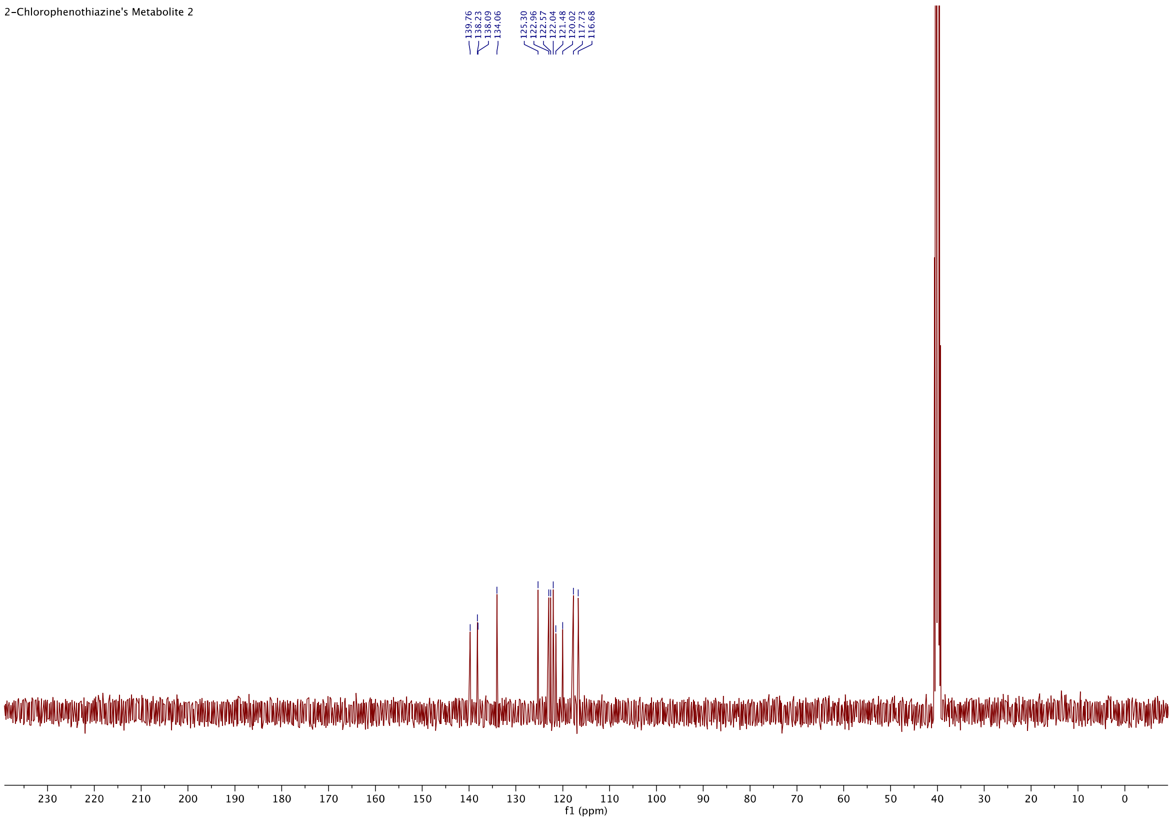

^{1}H-NMR spectrum **CPZ** (DMSO-d_6, 400 MHz)

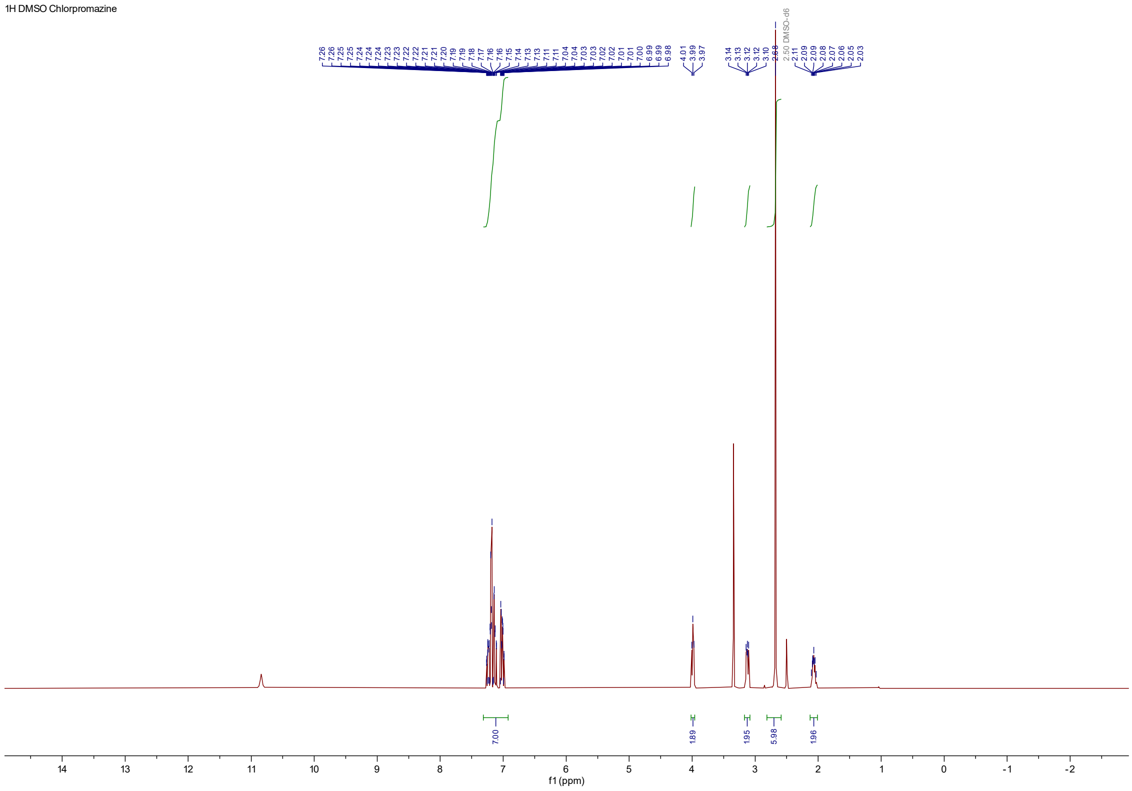

^{13}C-NMR spectrum **CPZ** (DMSO-d_6, 101 MHz)

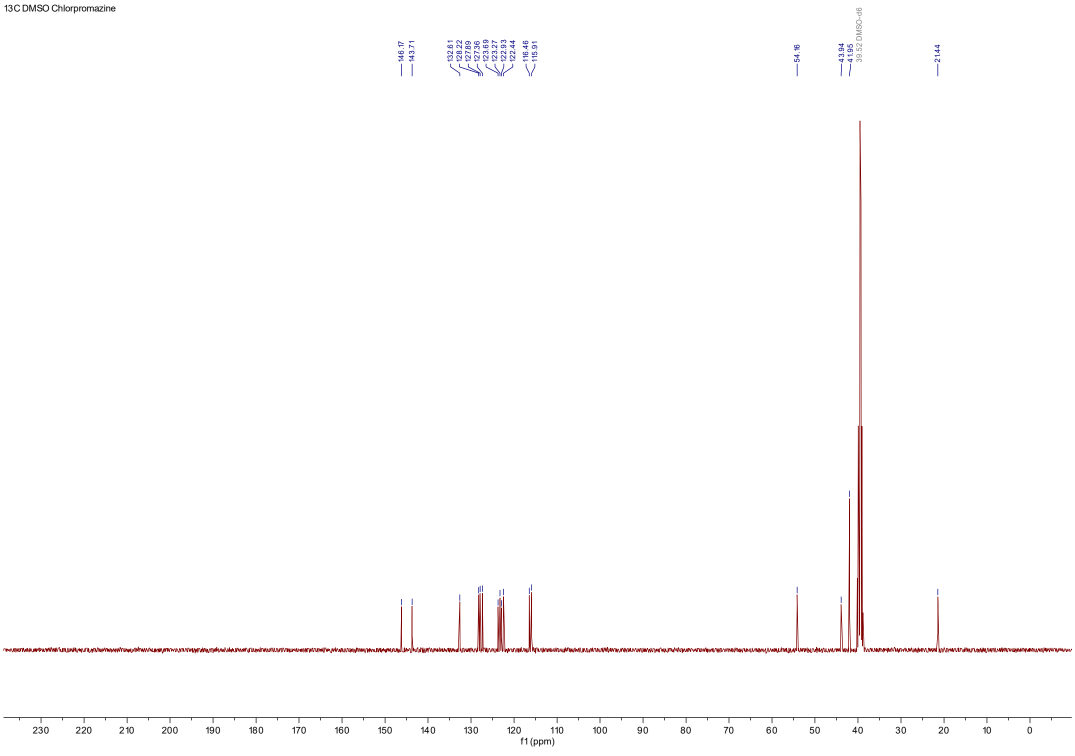

^{1}H-NMR spectrum **CPZ-SO** (DMSO-d_6, 400 MHz)

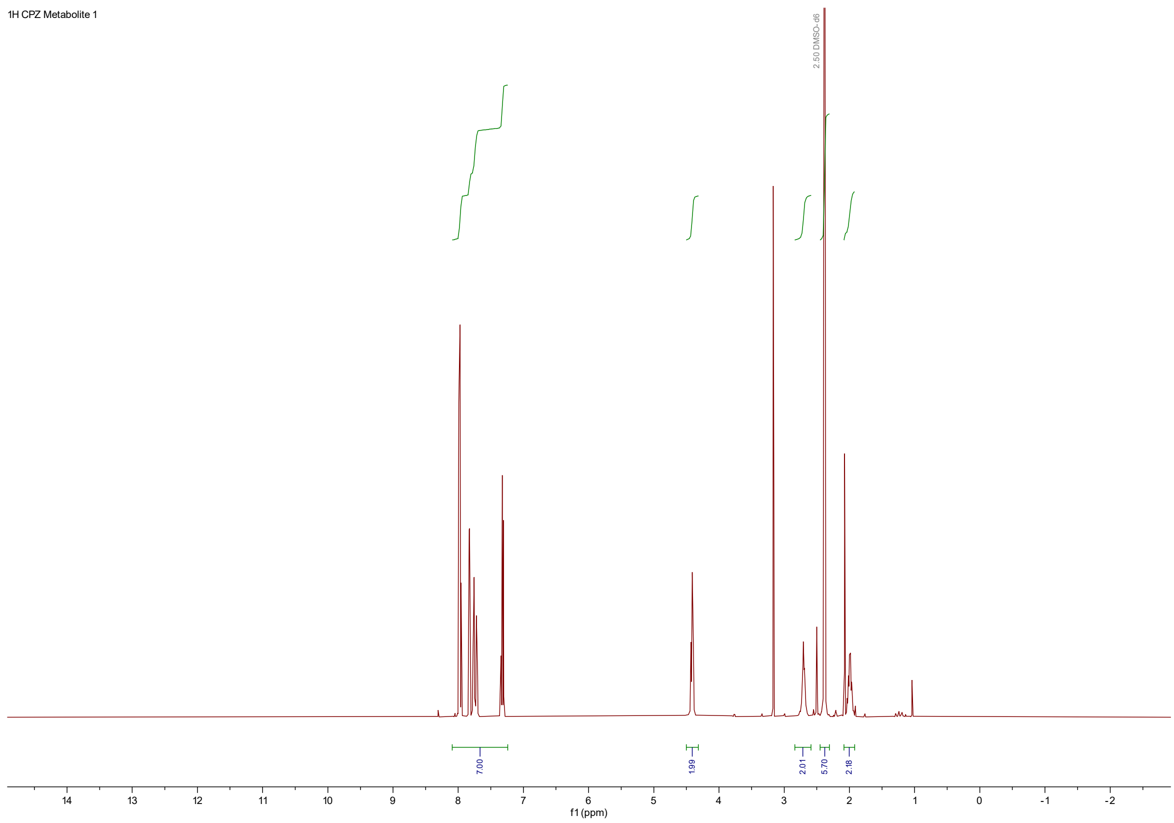

^{13}C-NMR spectrum **CPZ-SO** (DMSO-d_6, 101 MHz)

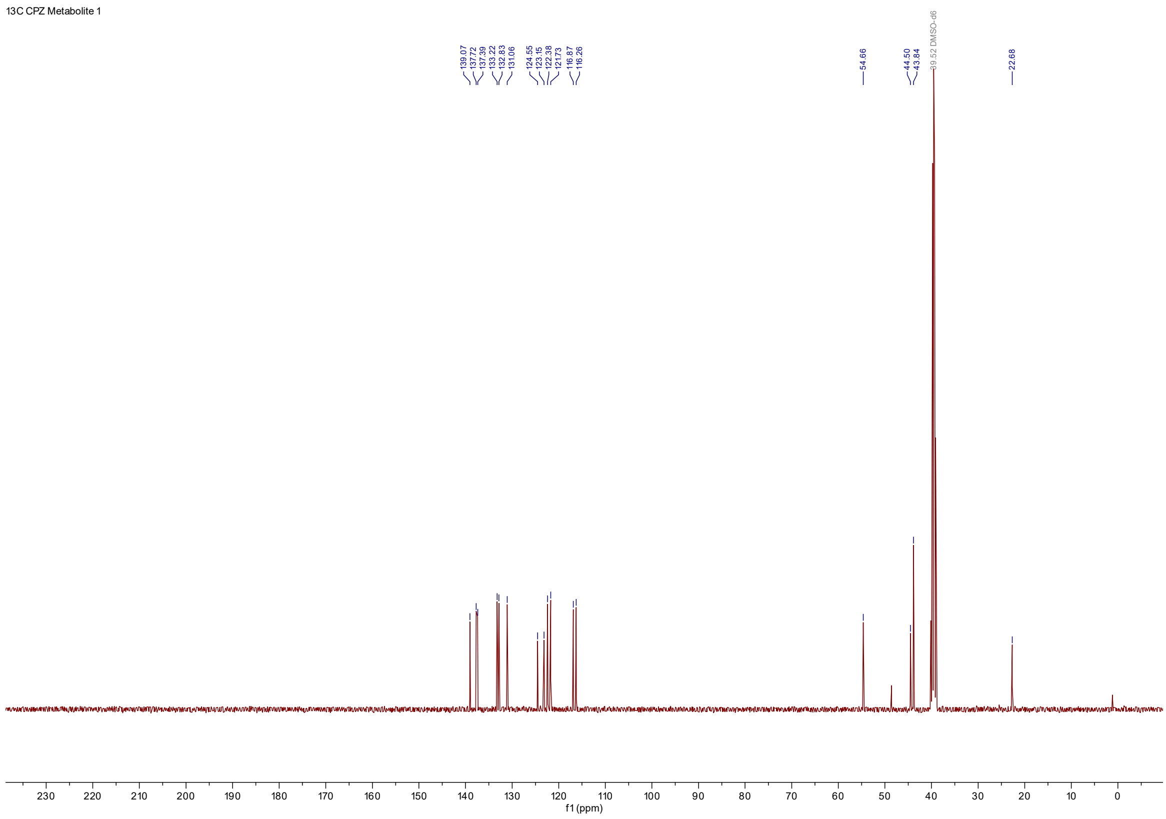

HRMS spectrum **CPZ-SO**

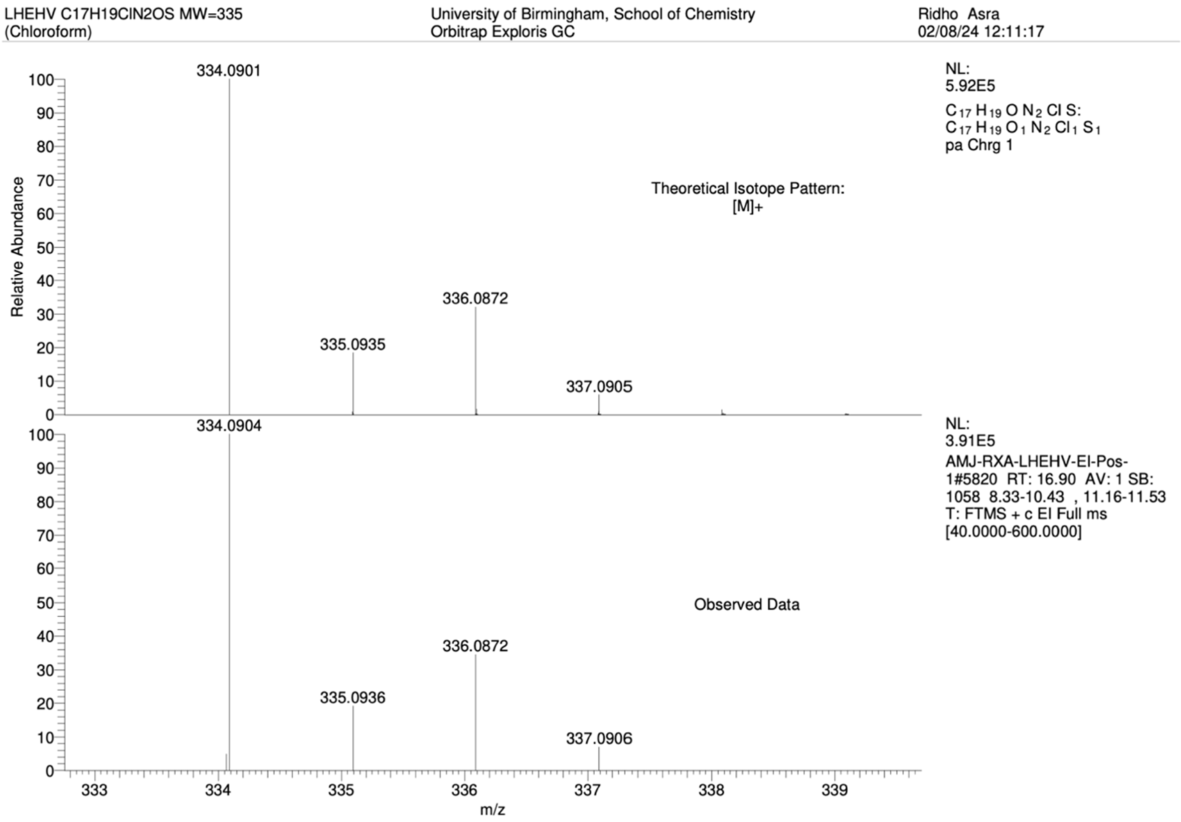

^{1}H-NMR spectrum Tetrabutylammonium hexafluorophosphate ($TBAPF_6$) (DMSO-d_6, 400 MHz)

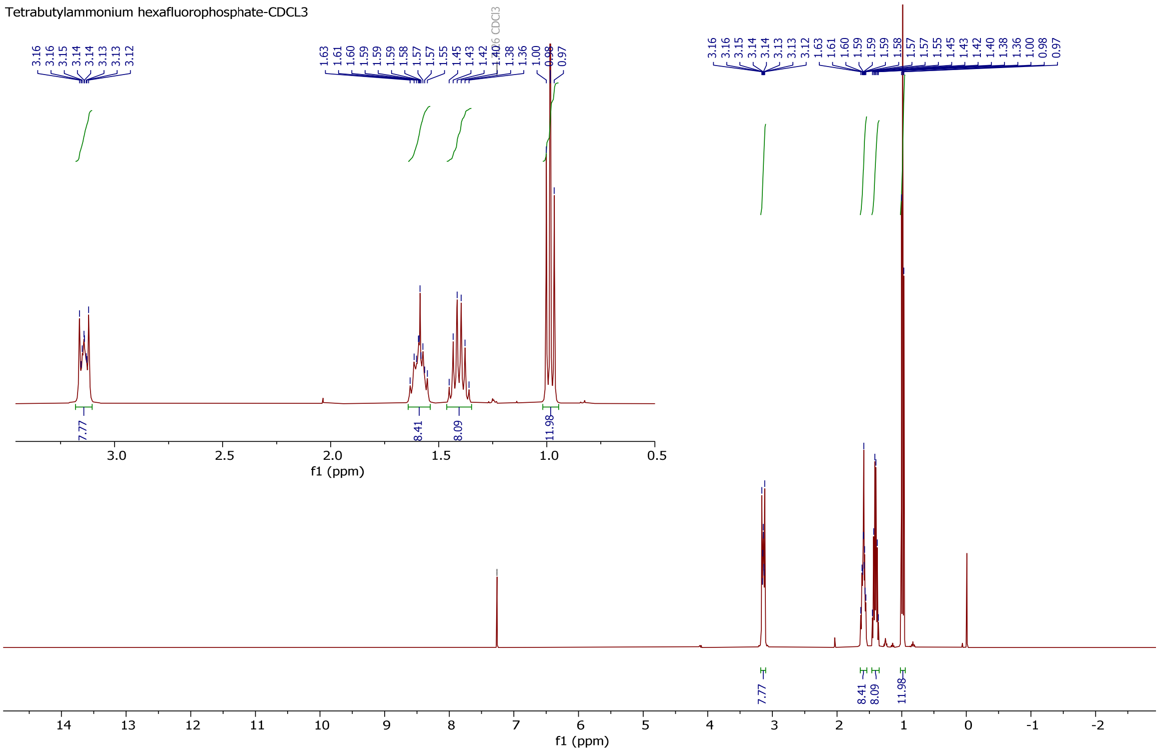

^{13}C-NMR spectrum Tetrabutylammonium hexafluorophosphate ($TBAPF_6$) (DMSO-d_6, 101 MHz)

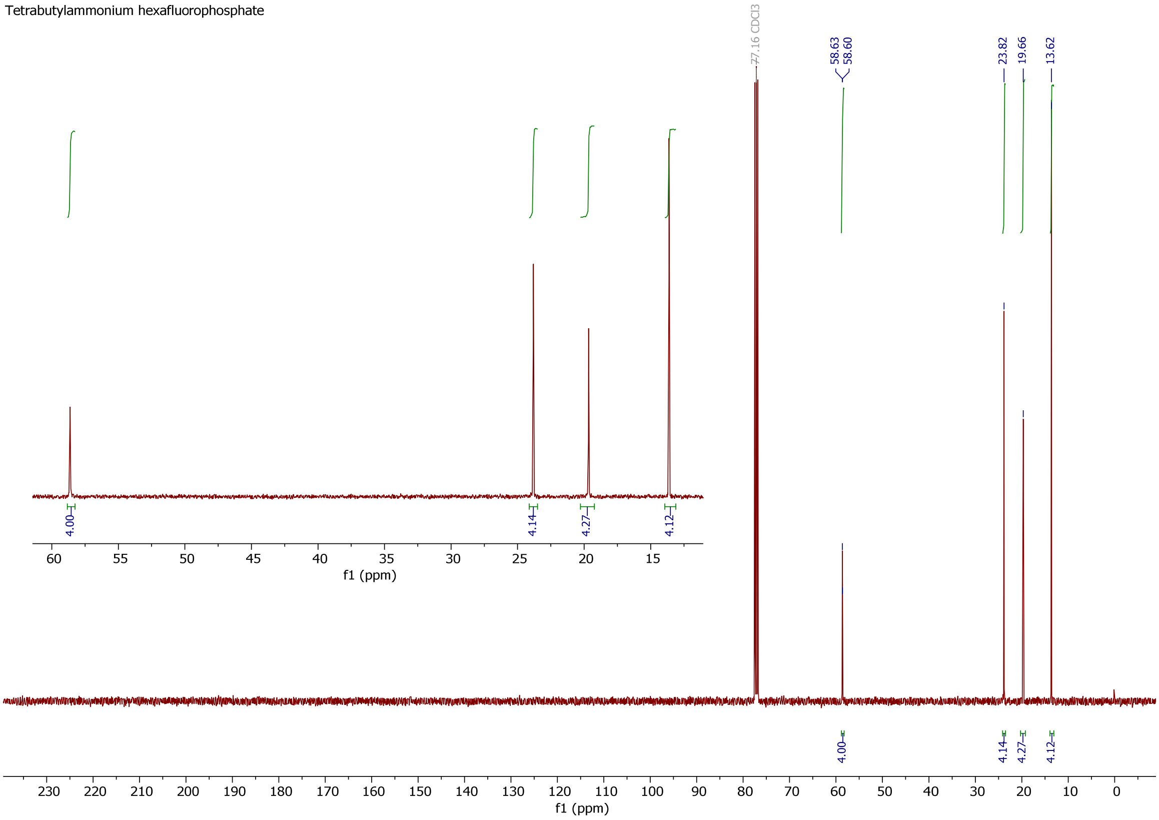

HPLC Data

a. 2-Chlorophenothiazine Metabolites

1 2CPTZ (1 mL min^{-1}, 0.05% TFA in water: acetonitrile)

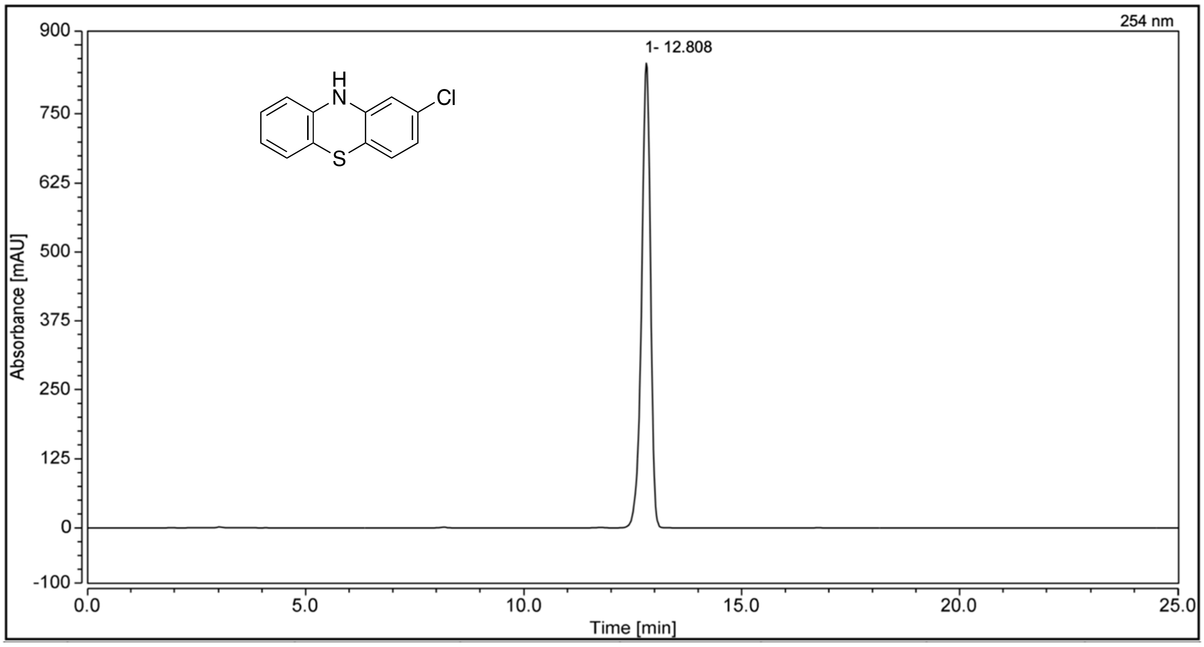

No.	Peak Name	Retention Time	Area mAU*min	Height mAU	Relative Area %	Relative Height %
1	2-Chlorophenothiazine	12.808	198.819	841.717	99.04	99.09

2 2CPTZ-SO (1 mL min^{-1}, 0.05% TFA in water: acetonitrile)

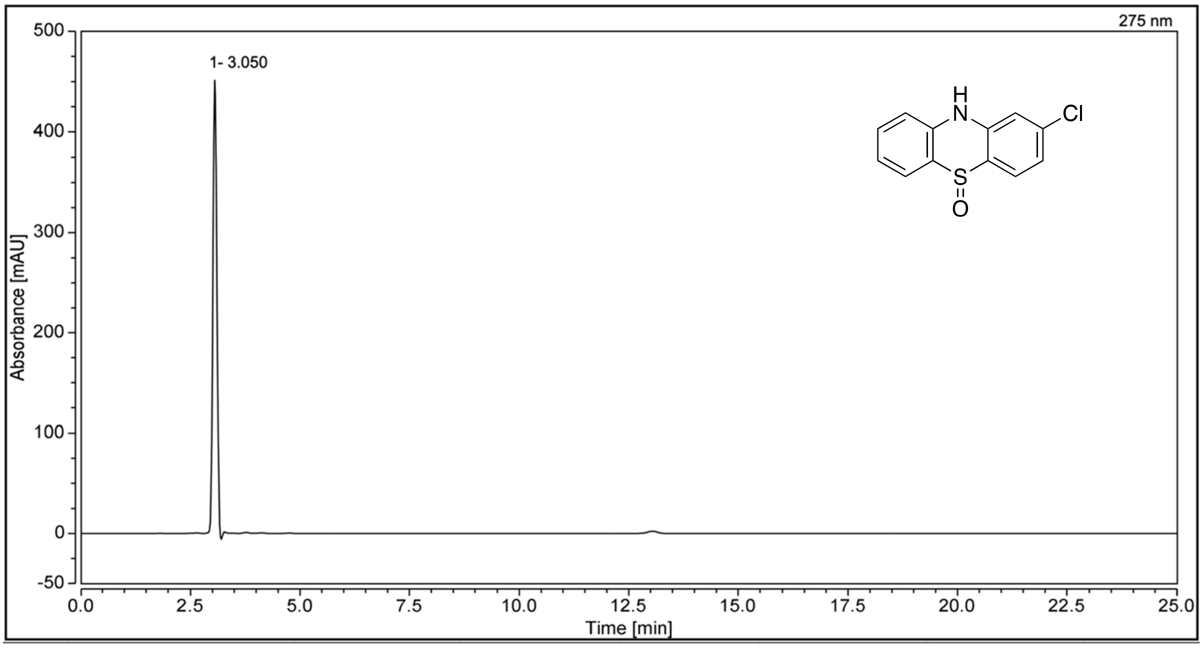

No.	Peak Name	Retention Time	Area mAU*min	Height mAU	Relative Area %	Relative Height %
1	**2CPTZ-SO**	3.050	49.560	456.748	41.56	80.77

3 2CPTZ-SO_2 (1 mL min^{-1}, 0.05% TFA in water: acetonitrile)

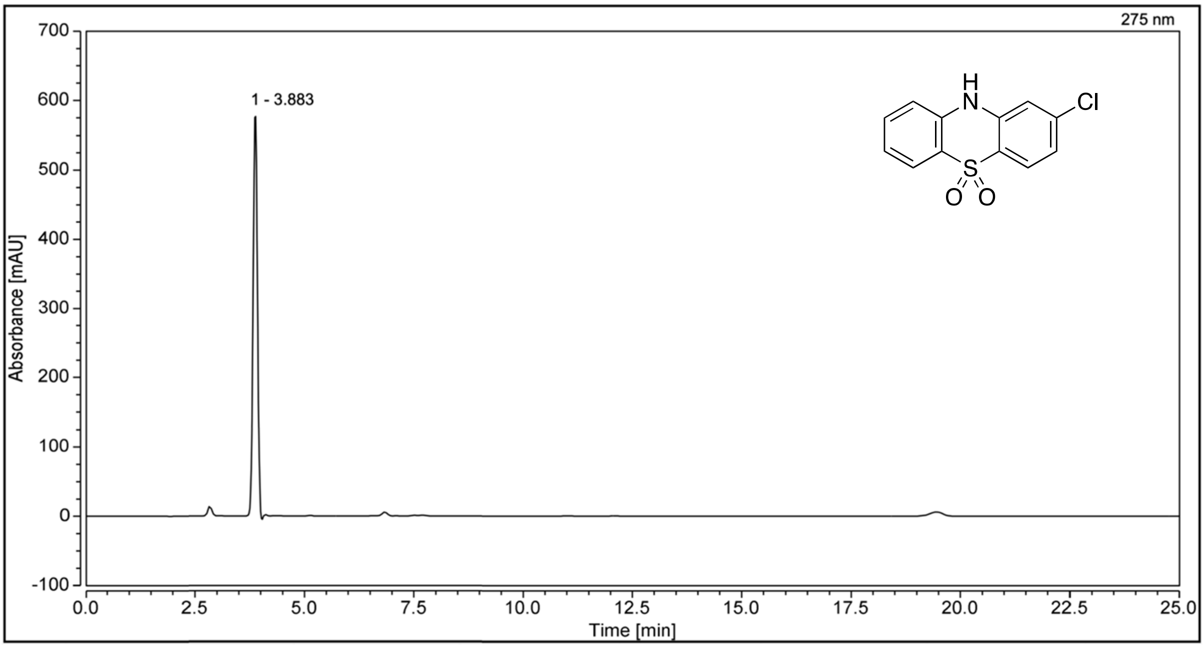

No.	Peak Name	Retention Time	Area mAU*min	Height mAU	Relative Area %	Relative Height %
1	**2CPTZ-SO_2**	3.883	67.373	579.703	87.55	95.55

Procedure A1: 1.0 mA, 6 hrs, 0.52 F/mol, Maximum applied voltage 1.28 V

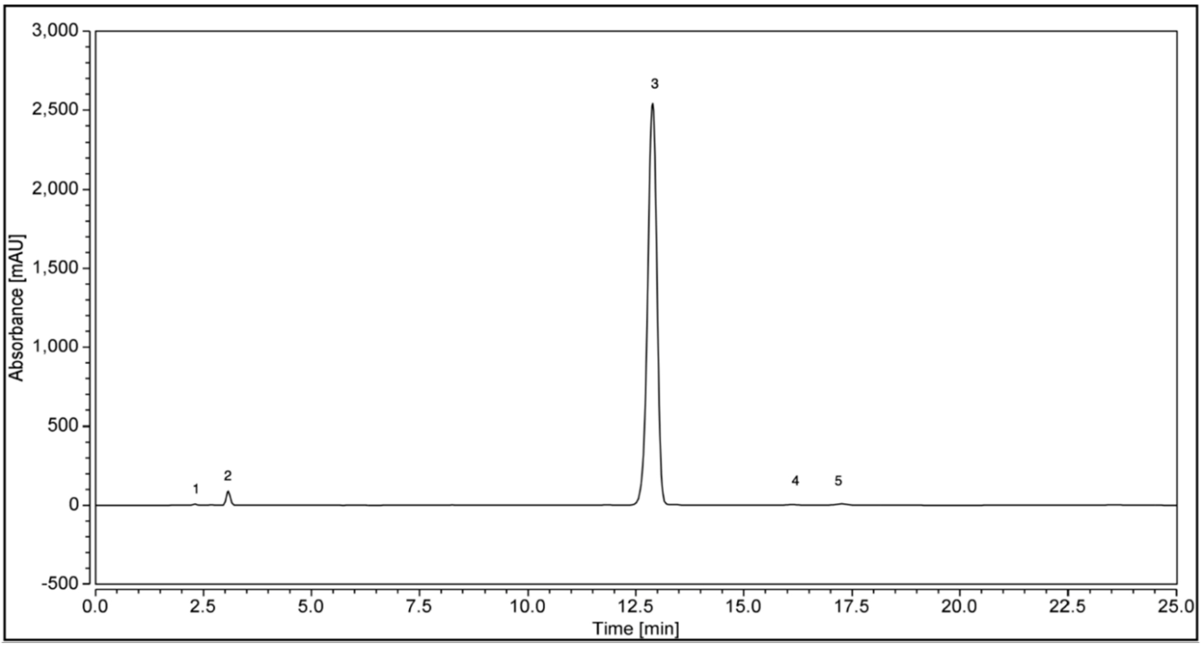

No.	Rt	Name	AUC	Ratio
1	2.308	Unknown	1.018	
2	3.075	**2CPTZ-SO**	9.296	
3	12.892	2-chlorophenothiazine	633.62	
4	16.125	Unknown	1.521	
5	17.275	Unknown	3.059	

Procedure A2: 0.5 mA, 24 hrs, 1.04 F/mol, Maximum applied voltage 3.20 V

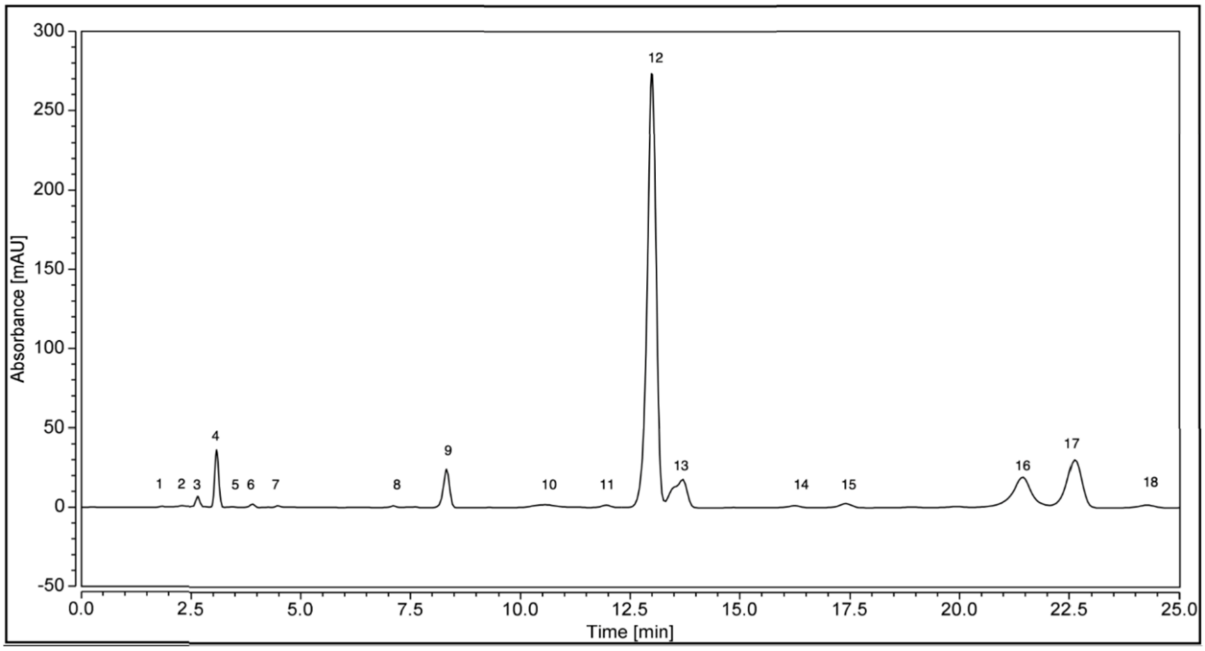

No.	Rt	Name	AUC	Ratio
1	1.842	Unknown	0.123	
2	2.308	Unknown	0.196	
3	2.642	Unknown	0.887	
4	3.075	**2CPTZ-SO**	3.869	13
5	3.442	Unknown	0.042	
6	3.908	**2CPTZ-SO$_2$**	0.291	1
7	4.475	Unknown	0.137	
8	7.125	Unknown	0.158	
9	8.325	Unknown	3.982	
10	10.558	Metabolite (MW: 318.9214)	1.236	
11	12.301	Metabolite (MW: 277.9881)	0.343	
12	12.992	2CPTZ	64.812	223
13	13.692	Unknown	6.848	
14	16.242	Unknown	0.450	
15	17.408	Unknown	0.953	
16	21.442	Unknown	10.475	
17	22.625	Unknown	12.122	
18	24.258	Unknown	0.758	

Procedure A3: 1.0 mA, 24 hrs, 2.11 F/mol, Maximum applied voltage 3.71 V

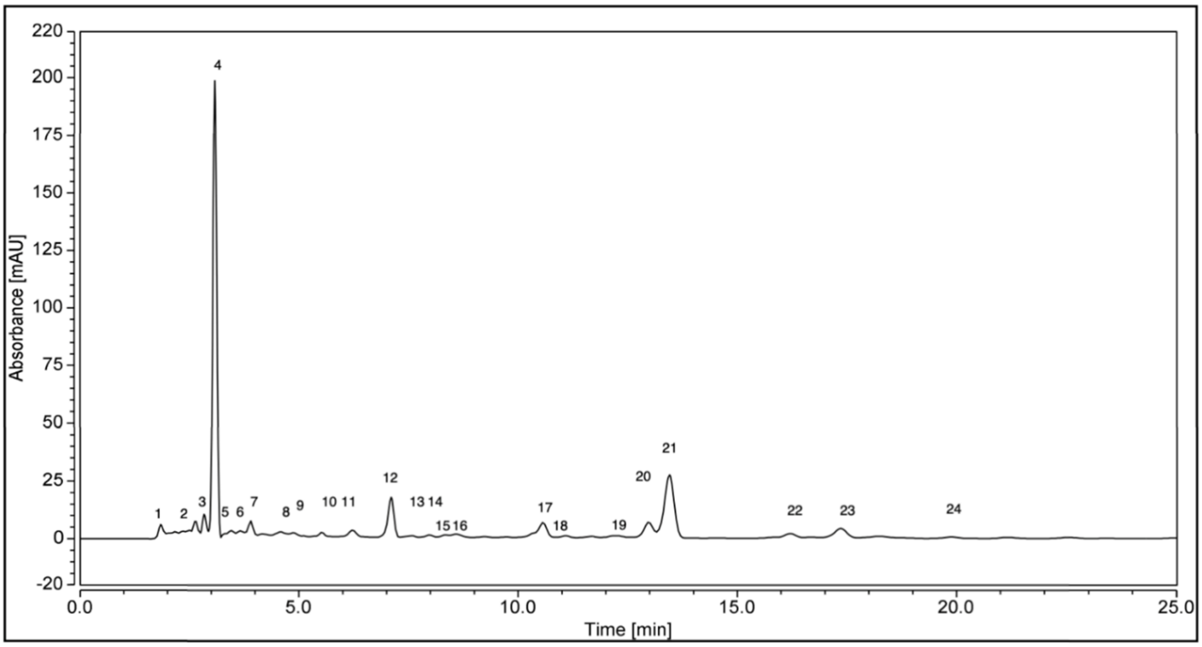

No.	Rt	Name	AUC	Ratio
1	1.842	Unknown	1.520	
2	2.642	Unknown	0.992	
3	2.842	Unknown	1.199	
4	3.075	**2CPTZ-SO**	21.153	16
5	3.458	Unknown	0.514	
6	3.658	Unknown	0.536	
7	3.908	**2CPTZ-SO_2**	1.345	1
8	4.575	Unknown	0.624	
9	4.875	Unknown	0.410	
10	5.525	Metabolite (MW: 187.2502)	0.199	
11	6.225	Unknown	0.588	
12	7.108	Unknown	2.737	
13	7.575	Unknown	0.160	
14	7.975	Unknown	0.199	
15	8.342	Unknown	0.215	
16	8.592	Unknown	0.374	
17	10.575	Metabolite (MW: 318.9214)	1.468	
18	11.075	Unknown	0.139	
19	12.208	Metabolite (MW: 277.9881)	0.286	
20	12.975	2CPTZ	1.632	1.2
21	13.458	Unknown	7.274	
22	16.208	Unknown	0.580	
23	17.358	Unknown	1.268	
24	19.875	Unknown	0.280	

Procedure A4: 1.5 mA, 24 hrs, 3.03 F/mol, Maximum applied voltage 4.10 V

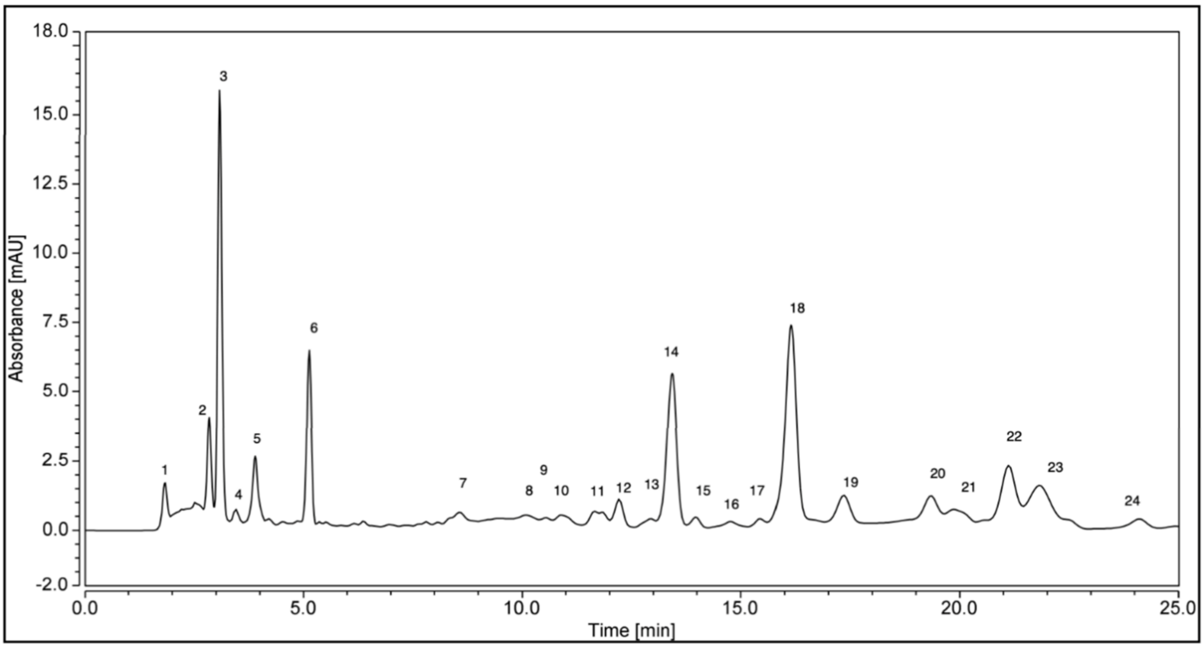

No.	Rt	Name	AUC	Ratio
1	1.842	Unknown	0.186	
2	2.842	Unknown	0.365	
3	3.075	**2CPTZ-SO**	1.689	47
4	N/A	Unknown	N/A	
5	3.892	**2CPTZ-SO_2**	0.384	11
6	5.125	Metabolite (MW: 187.2502)	0.775	
7	N/A	Unknown	N/A	
8	10.608	Metabolite (MW: 318.9214)	N/A	
9	N/A	Unknown	N/A	
10	N/A	Unknown	N/A	
11	N/A	Unknown	N/A	
12	12.308	Metabolite (MW: 277.9881)	N/A	
13	12.980	2CPTZ	0.036	1
14	13.425	Unknown	1.366	
15	N/A	Unknown	N/A	
16	N/A	Unknown	N/A	
17	N/A	Unknown	N/A	
18	16.142	Unknown	2.148	
19	N/A	Unknown	N/A	
20	N/A	Unknown	N/A	
21	N/A	Unknown	N/A	
22	21.108	Unknown	0.563	
23	N/A	Unknown	N/A	
24	N/A	Unknown	N/A	

Procedure A5: 2.0 mA, 24 hrs, 4.18 F/mol, Maximum applied voltage 4.90 V

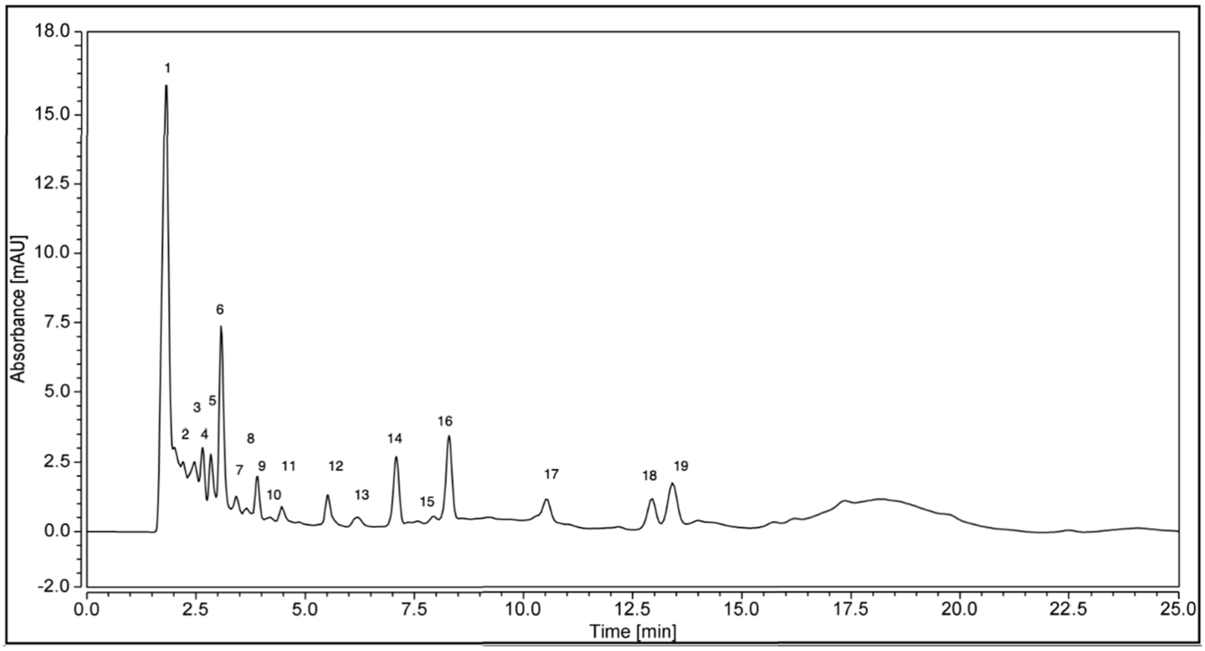

No.	Rt	Name	AUC	Ratio
1	1.808	Unknown	4.463	
2	2.208	Unknown	0.026	
3	2.475	Unknown	0.123	
4	2.658	Unknown	0.172	
5	2.842	Unknown	0.149	
6	3.075	**2CPTZ-SO**	1.253	5
7	3.425	Unknown	0.213	
8	3.642	Unknown	0.142	
9	3.908	**2CPTZ-SO_2**	0.252	1
10	4.192	Unknown	0.086	
11	4.458	Unknown	0.178	
12	5.525	Metabolite (MW: 187.2502)	0.168	
13	6.192	Unknown	0.084	
14	7.092	Unknown	0.383	
15	7.942	Unknown	0.036	
16	8.292	Unknown	0.488	
17	10.525	Metabolite (MW: 318.9214)	0.225	
18	12.942	2CPTZ	0.269	1
19	13.408	Unknown	0.485	

Procedure A6: 1.5 mA, 24 hrs, 3.11 F/mol, Maximum applied voltage 5.39 V

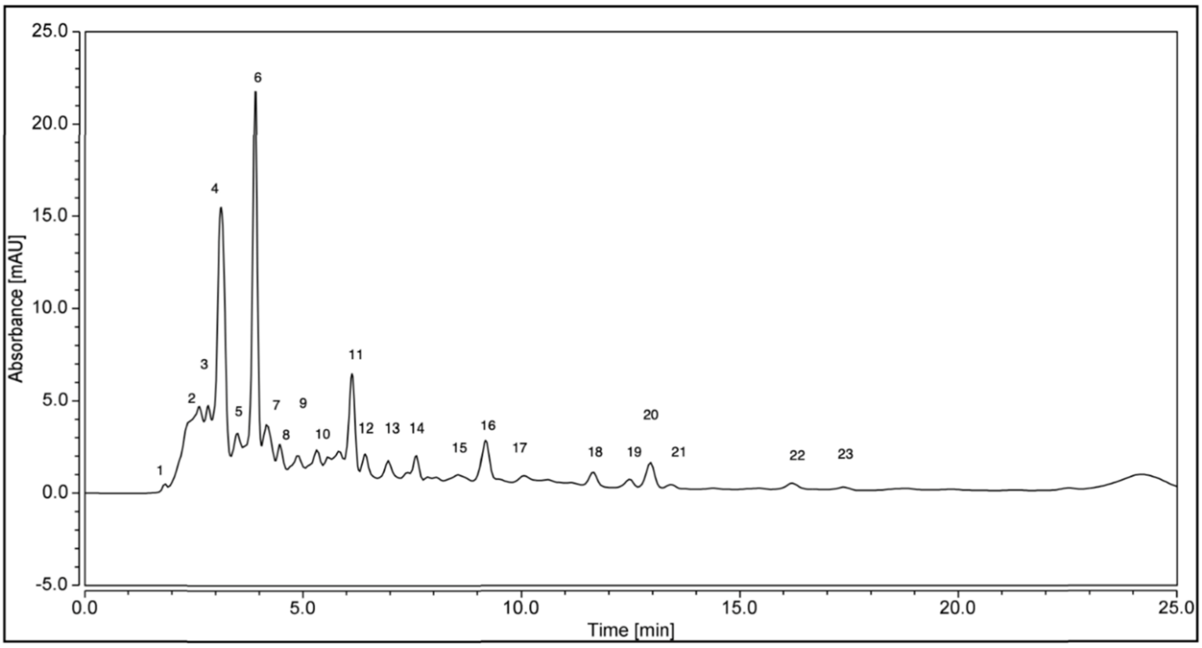

No.	Rt	Name	AUC	Ratio
1	1.842	Unknown	0.084	
2	2.625	Unknown	1.892	
3	2.825	Unknown	0.720	
4	3.125	Unknown	3.553	
5	3.492	Unknown	0.136	
6	3.892	**2CPTZ-SO_2**	5.186	
7	4.175	Unknown	0.247	
8	4.708	Unknown	0.161	
9	4.892	Unknown	0.518	
10	5.325	Unknown	0.572	
11	6.125	Unknown	1.260	
12	6.425	Unknown	0.558	
13	6.958	Unknown	0.586	
14	7.592	Unknown	0.416	
15	8.558	Unknown	0.254	
16	9.175	Unknown	0.807	
17	10.058	Metabolite (MW: 318.9214)	0.461	
18	11.642	Unknown	0.355	
19	12.958	2CPTZ	0.249	
20	13.425	Unknown	0.420	
21	13.425	Unknown	0.129	
22	16.192	Unknown	0.230	
23	17.375	Unknown	0.104	

C. Chlorpromazine Metabolites

5 Chlorpromazine (1 mL min^{-1}, 0.05% TFA in water: acetonitrile)

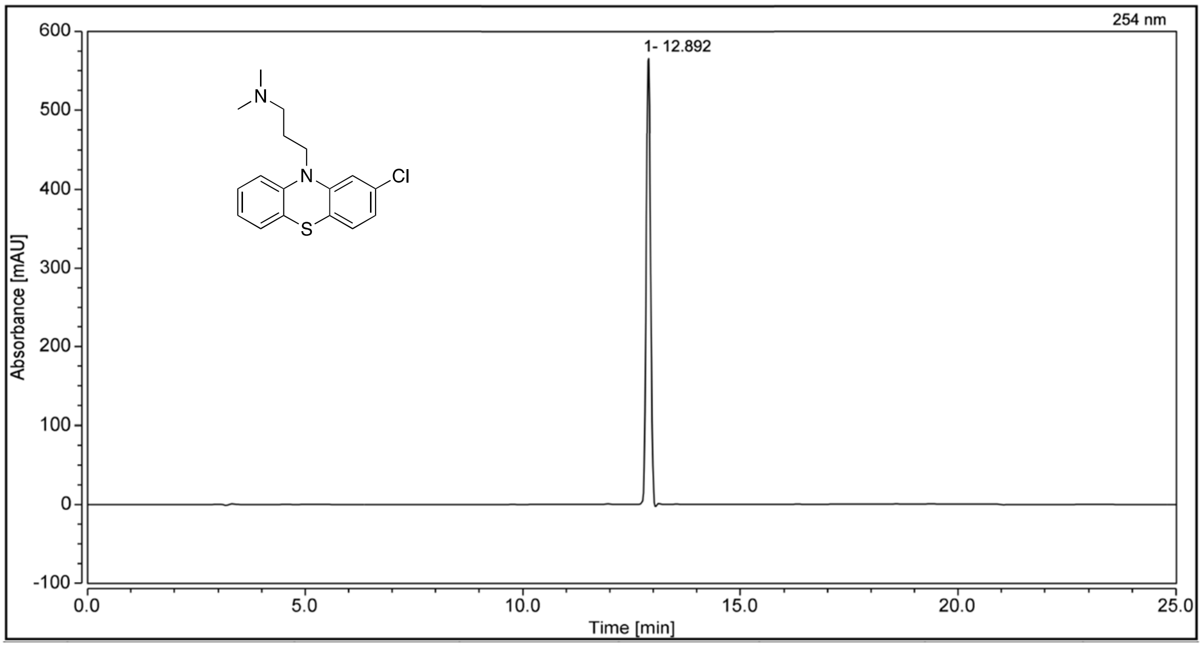

No.	Peak Name	Retention Time	Area mAU*min	Height mAU	Relative Area %	Relative Height %
1	Chlorpromazine	12.892	66.126	566.069	73.12	96.72

5 Chlorpromazine (1 mL min^{-1}, 0.05% TFA in water: acetonitrile, analysis for procedure J)

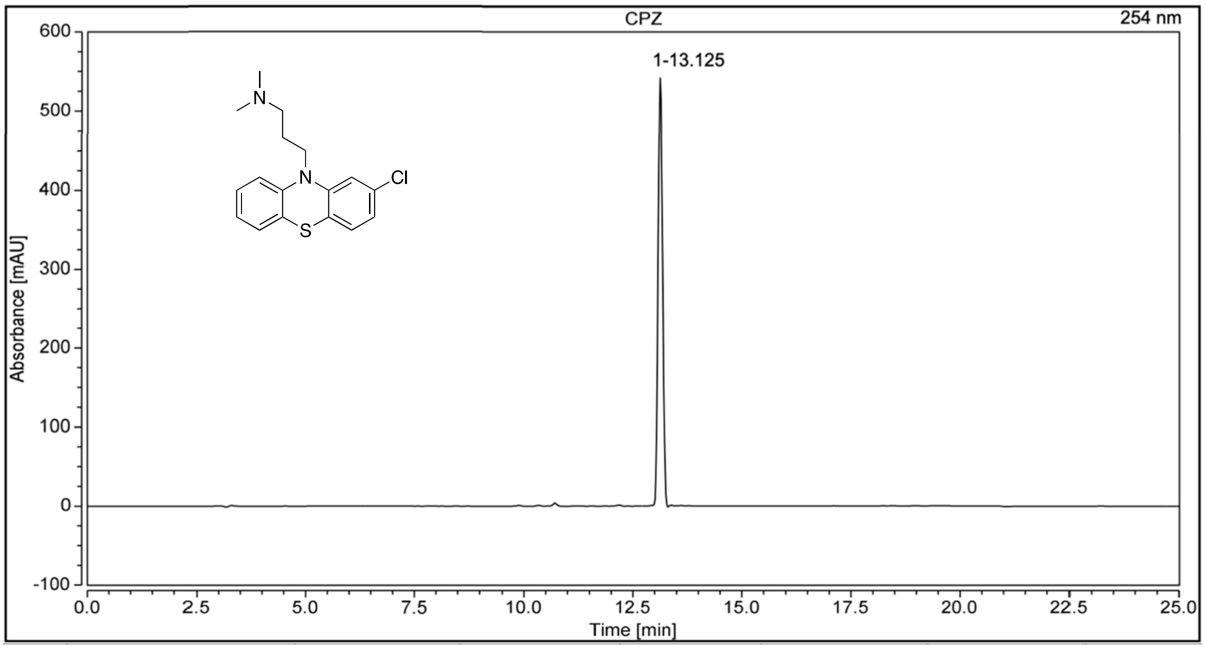

No.	Peak Name	Retention Time	Area mAU*min	Height mAU	Relative Area %	Relative Height %
1	Chlorpromazine	13.125	65.694	542.568	76.15	94.34

Procedure E1: 1.0 mA, 24 hrs, 2.81 F/mol, Maximum applied voltage 3.86 V

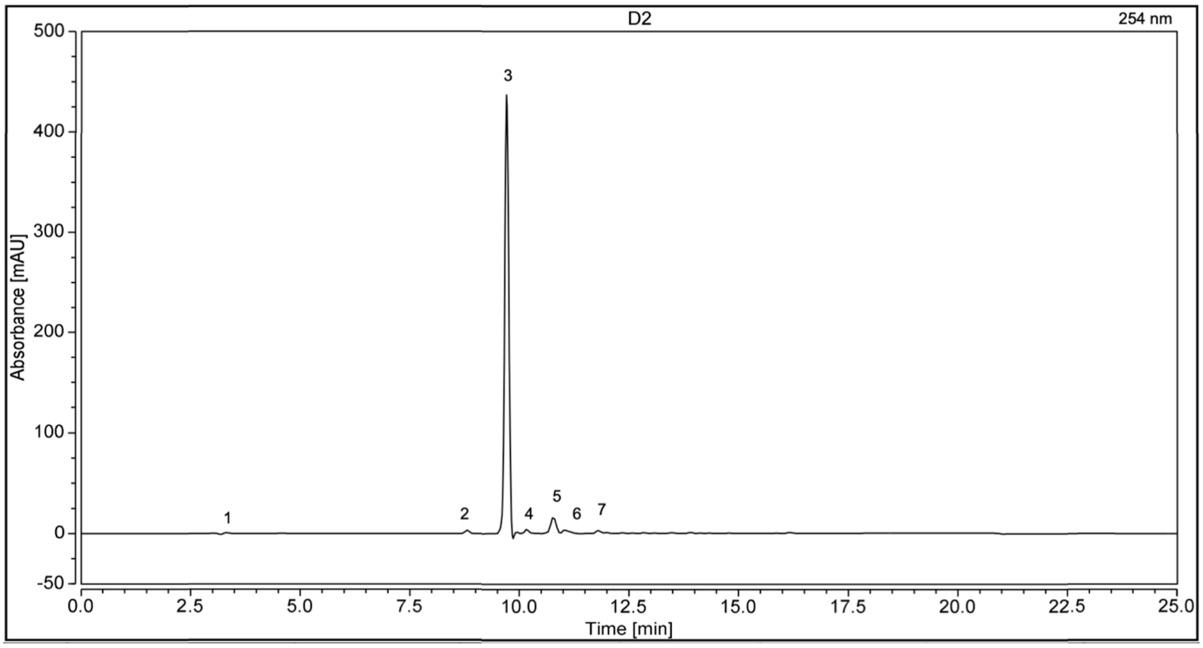

No.	Rt	Name	AUC	Ratio
1	3.308	Unknown	1.818	-
2	8.808	Unknown	4.430	-
3	9.708	**CPZ-sulfoxide**	50.30	11
4	10.175	Unknown	2.698	-
5	10.775	**CPZ-sulfone**	4.506	1
6	11.142	Unknown	1.762	-
7	11.792	Unknown	1.813	-

Procedure E2: 1.5 mA, 24 hrs, 4.35 F/mol, Maximum applied voltage 4.07 V

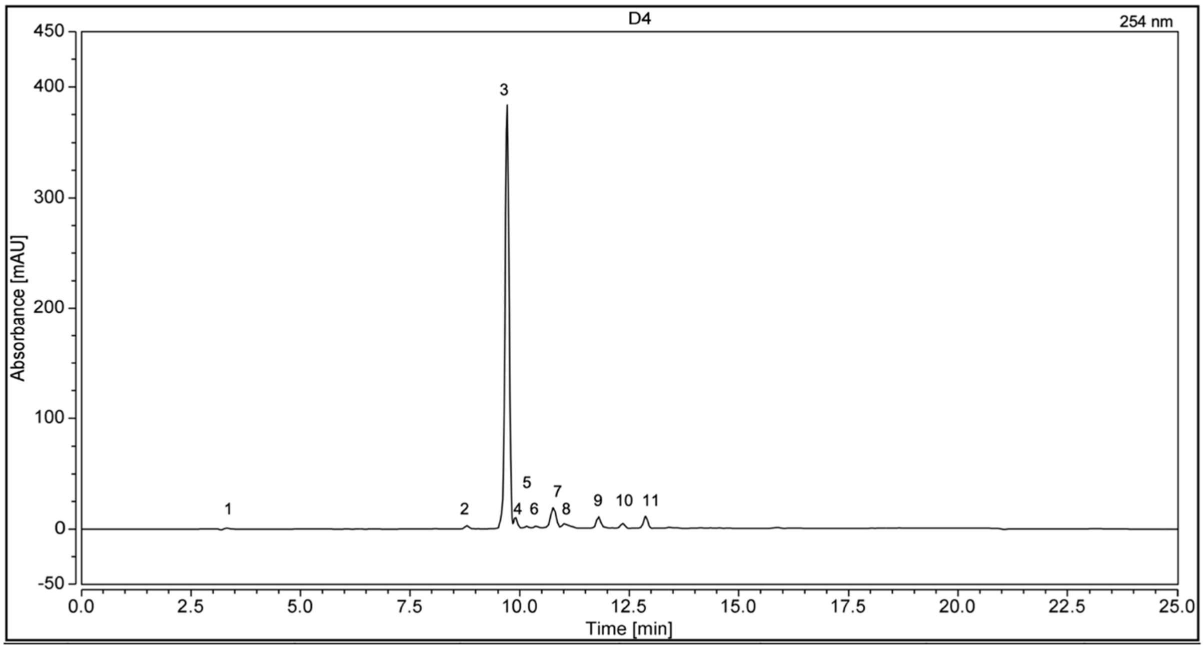

No.	Rt	Name	AUC	Ratio
1	3.308	Unknown	2.201	-
2	8.808	Unknown	0.350	-
3	9.708	**CPZ-sulfoxide**	41.66	35
4	9.892	Unknown	0.995	-
5	10.158	Unknown	0.308	-
6	10.375	Unknown	0.306	-
7	10.758	**CPZ-sulfone**	2.954	3
8	11.008	Unknown	0.929	-
9	11.792	Unknown	1.420	-
10	12.342	Unknown	0.533	-
11	12.875	**CPZ**	1.203	1

Procedure E3: 2.0 mA, 24 hrs, 5.84 F/mol, Maximum applied voltage 4.12 V

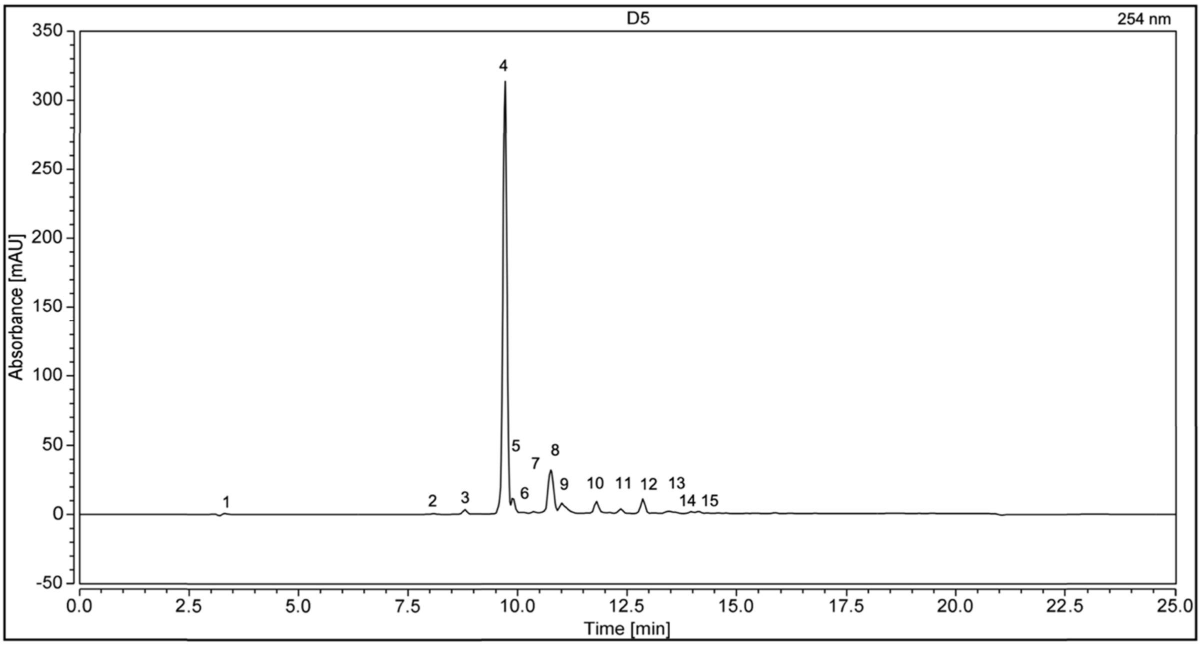

No.	Rt	Name	AUC	Ratio
1	3.308	Unknown	1.819	-
2	8.092	Unknown	0.064	-
3	8.808	Unknown	0.413	-
4	9.708	**CPZ-sulfoxide**	33.81	25
5	9.892	Unknown	1.158	-
6	10.142	Unknown	0.181	-
7	10.375	Unknown	0.313	-
8	10.758	**CPZ-sulfone**	4.932	4
9	11.008	Unknown	1.073	-
10	11.808	Unknown	1.340	-
11	12.358	Unknown	0.588	-
12	12.858	**CPZ**	1.352	1
13	13.458	Unknown	0.382	-
14	13.975	Unknown	0.231	-
15	14.142	Unknown	0.251	-

Procedure E4: 2.5 mA, 24 hrs, 7.28 F/mol, Maximum applied voltage 4.84 V

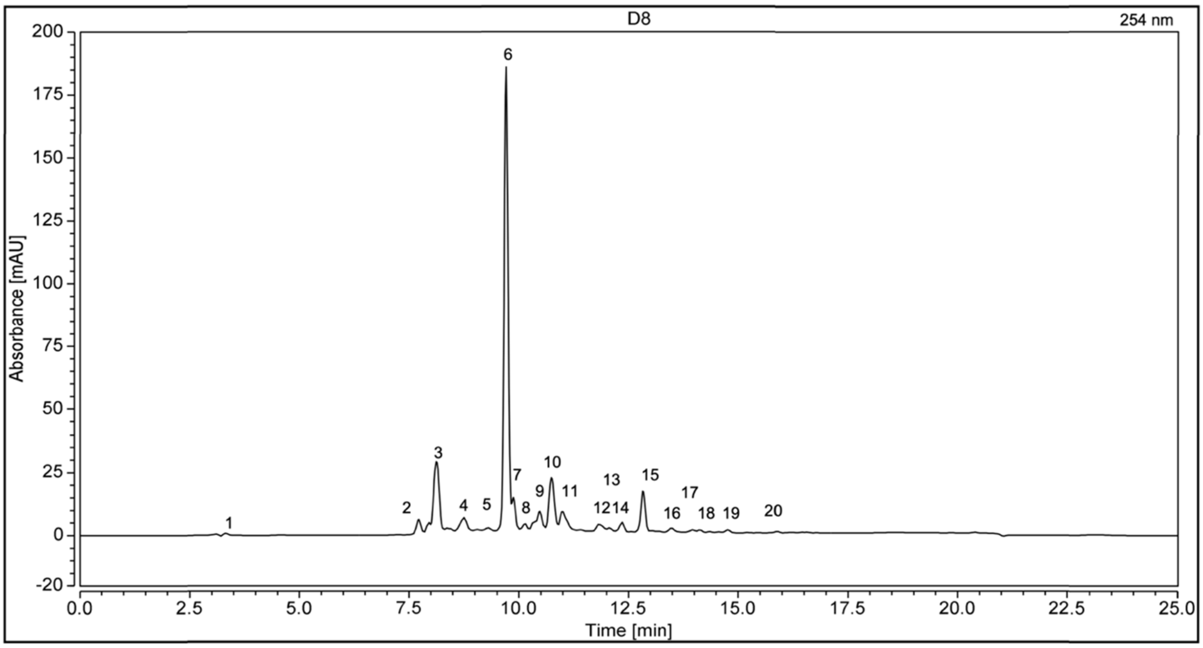

No.	Rt	Name	AUC	Ratio
1	3.308	Unknown	0.134	-
2	7.725	Unknown	0.722	-
3	8.125	Unknown	4.350	-
4	8.758	Unknown	1.299	-
5	9.308	Unknown	0.385	-
6	9.708	**CPZ-sulfoxide**	19.76	11
7	9.875	Unknown	1.355	-
8	10.142	Unknown	0.489	-
9	10.475	Unknown	1.013	-
10	10.742	**CPZ-sulfone**	3.111	2
11	10.992	Unknown	1.368	-
12	11.825	Unknown	0.524	-
13	12.075	Unknown	0.190	-
14	12.358	Unknown	0.472	-
15	12.825	**CPZ**	1.867	1
16	13.475	Unknown	0.229	-
17	13.958	Unknown	0.153	-
18	14.125	Unknown	0.120	-
19	14.758	Unknown	0.120	-
20	15.875	Unknown	0.071	-

Procedure E5: 3.0 mA, 24 hrs, 8.61 F/mol, Maximum applied voltage 5.09 V

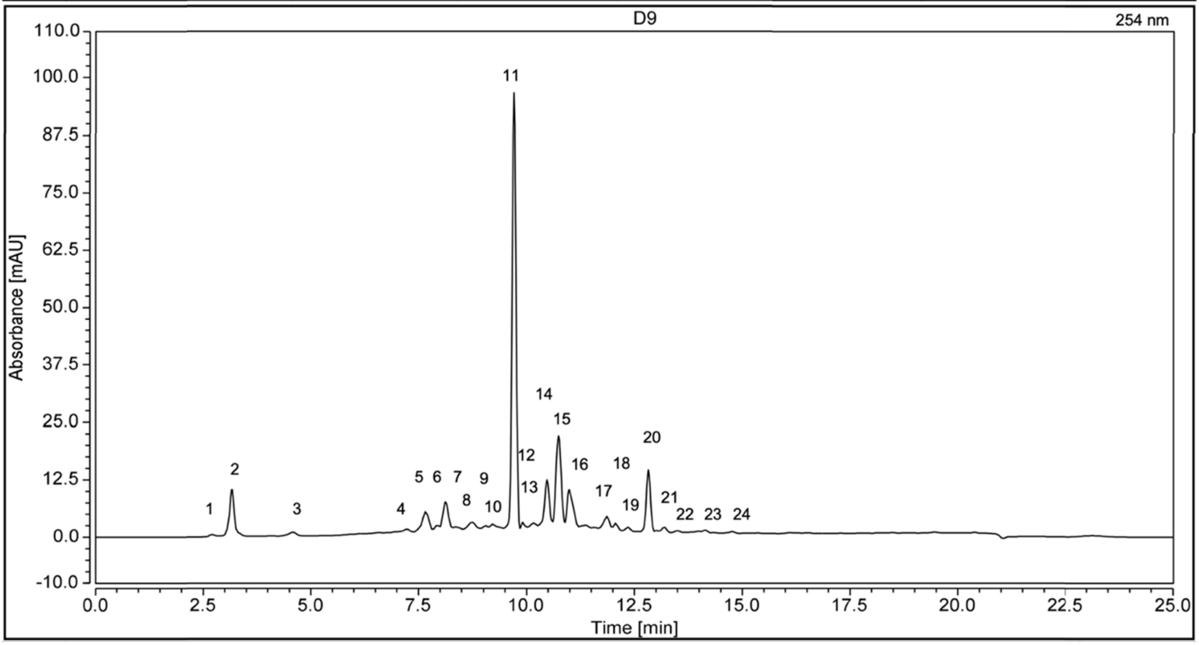

No.	Rt	Name	AUC	Ratio
1	2.692	Unknown	0.080	-
2	3.158	Unknown	1.359	-
3	4.575	Unknown	0.183	-
4	7.242	Unknown	0.134	-
5	7.658	Unknown	0.800	-
6	7.925	Unknown	0.052	-
7	8.125	Unknown	1.124	-
8	8.742	Unknown	0.423	-
9	-	Unknown	-	-
10	9.208	Unknown	0.415	-
11	9.708	**CPZ-sulfoxide**	10.063	7
12	9.908	Unknown	0.155	-
13	10.158	Unknown	0.238	-
14	10.475	Unknown	1.473	-
15	10.742	**CPZ-sulfone**	2.902	2
16	10.992	Unknown	1.339	-
17	11.858	Unknown	0.437	-
18	12.058	Unknown	0.157	-
19	12.358	Unknown	0.099	-
20	12.825	**CPZ**	1.503	1
21	13.192	Unknown	0.149	-
22	13.492	Unknown	0.069	-
23	14.142	Unknown	0.140	-
24	14.758	Unknown	0.056	-

Procedure E6: 3.0 mA, 24 hrs, 8.79 F/mol, Maximum applied voltage 2.69 V

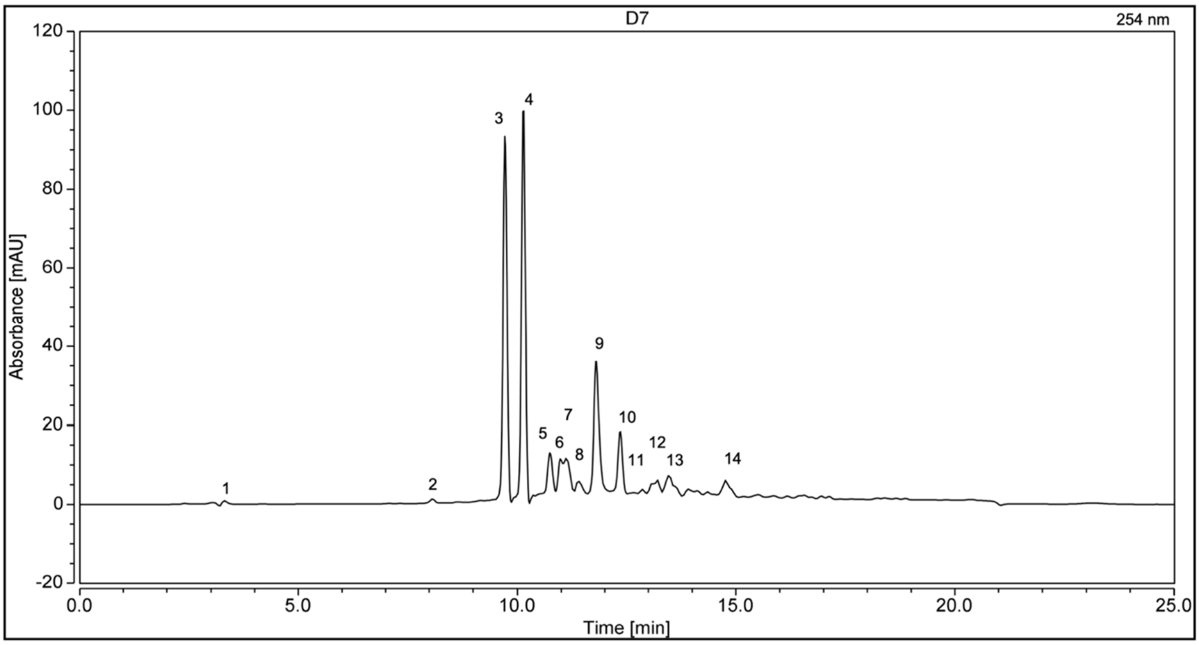

No.	Rt	Name	AUC	Ratio
1	3.308	Unknown	0.972	-
2	8.075	Unknown	0.142	-
3	9.708	**CPZ-sulfoxide**	10.255	-
4	10.142	Unknown	10.796	-
5	10.742	**CPZ-sulfone**	1.994	-
6	10.992	Unknown	1.569	-
7	11.108	Unknown	2.794	-
8	11.392	Unknown	0.435	-
9	11.792	Unknown	6.061	-
10	12.358	Unknown	2.990	-
11	12.858	**CPZ**	0.672	-
12	13.208	Unknown	0.899	-
13	13.475	Unknown	2.084	-
14	14.758	Unknown	1.283	-

6 CPZ-SO (1 mL min^{-1}, 0.05% TFA in water: acetonitrile)

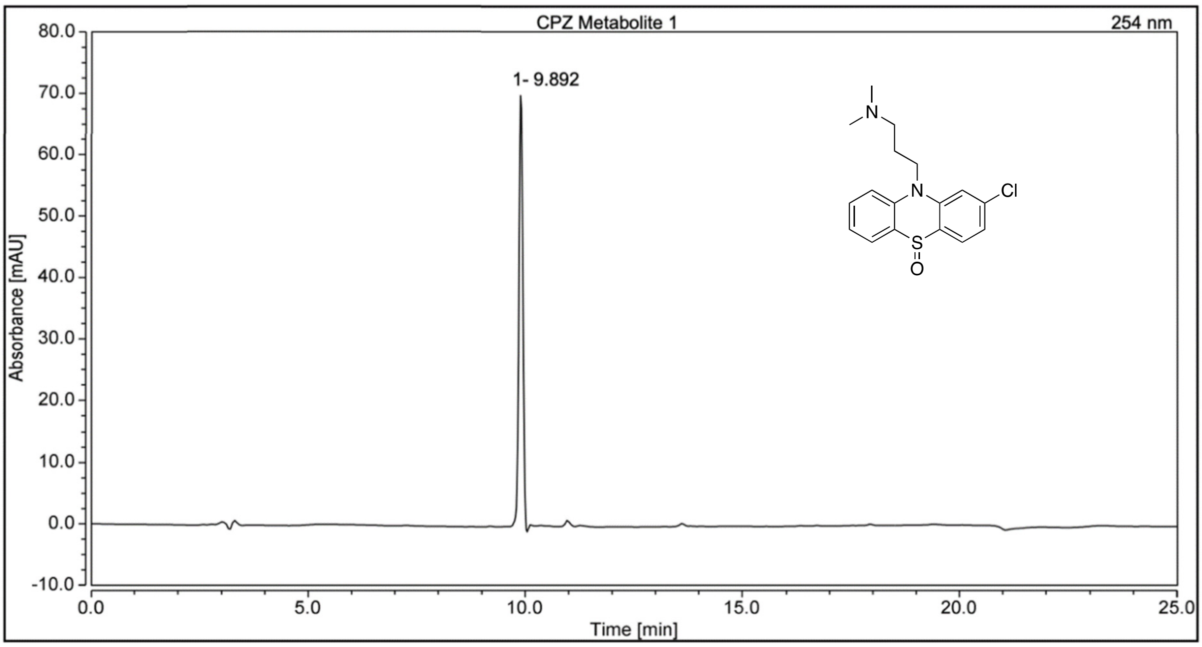

No.	Peak Name	Retention Time	Area mAU*min	Height mAU	Relative Area %	Relative Height %
1	CPZ-SO	9.892	7.747	70.943	42.17	87.65

7 CPZ-SO_2 (1 mL min^{-1}, 0.05% TFA in water: acetonitrile)

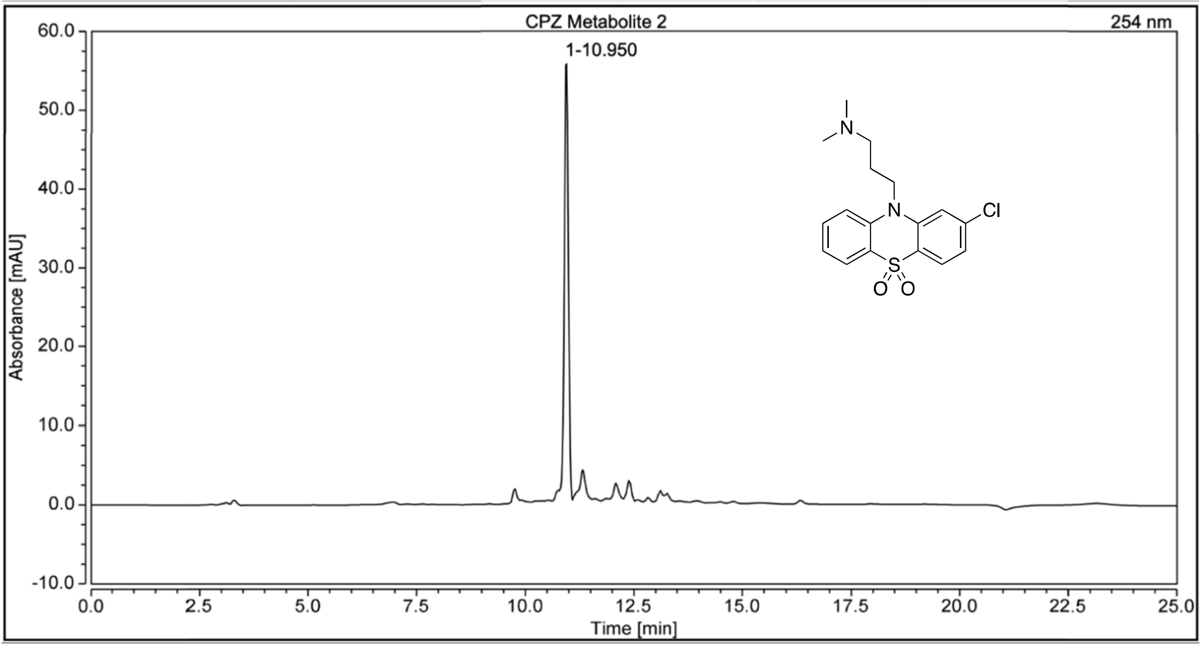

No.	Peak Name	Retention Time	Area mAU*min	Height mAU	Relative Area %	Relative Height %
1	CPZ-SO_2	10.950	6.217	55.975	38.03	60.72

LCMS Data

a. 2-Chlorophenothiazine Metabolites

2 2CPTZ-SO molecular weight 249 (2.64 min, LCMS **ES-** 247.9937, **ES+** 250.0100)

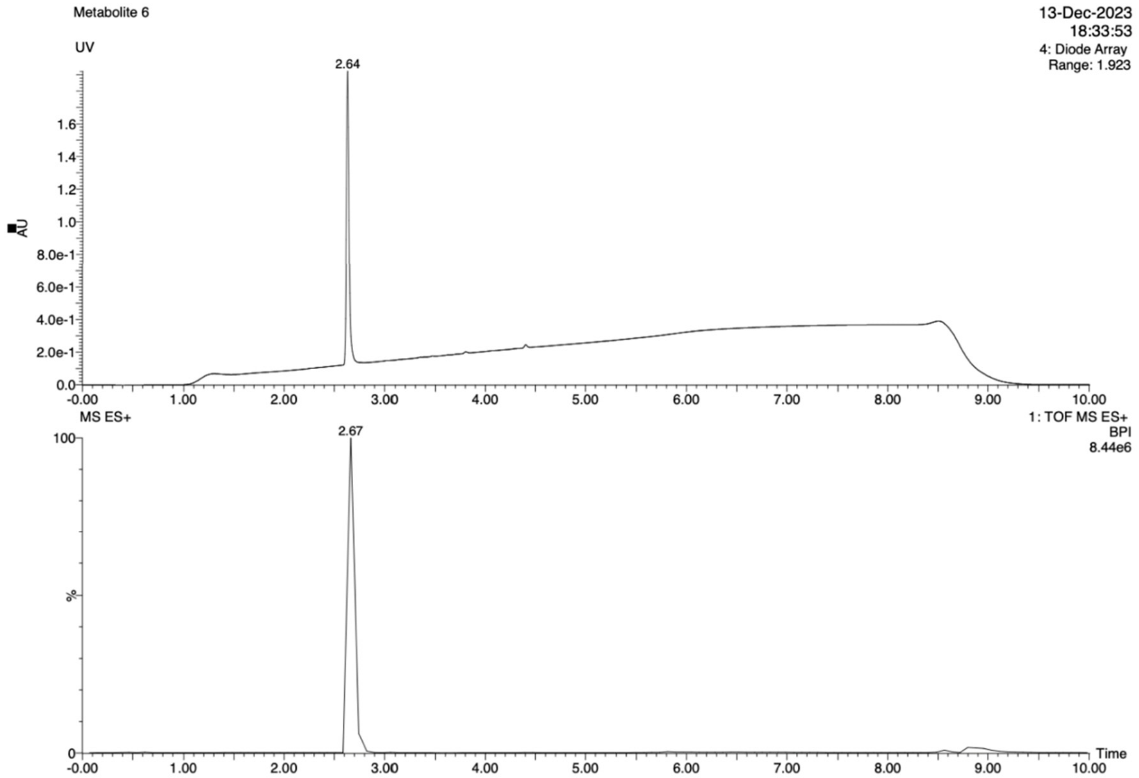

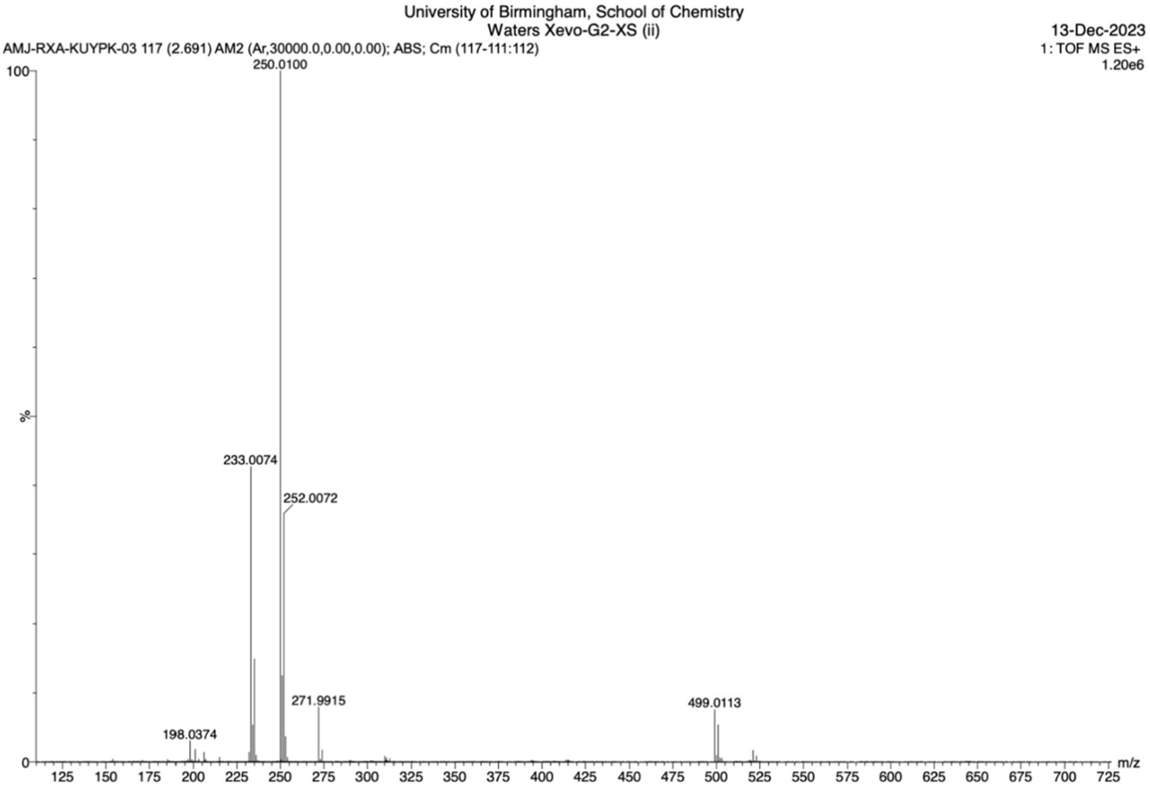
University of Birmingham, School of Chemistry
Waters Xevo-G2-XS (ii)
13-Dec-2023
AMJ-RXA-KUYPK-03 117 (2.691) AM2 (Ar,30000.0,0.00,0.00); ABS; Cm (117-111:112)
1: TOF MS ES+
1.20e6
250.0100
233.0074
252.0072
271.9915
198.0374
499.0113
%
m/z

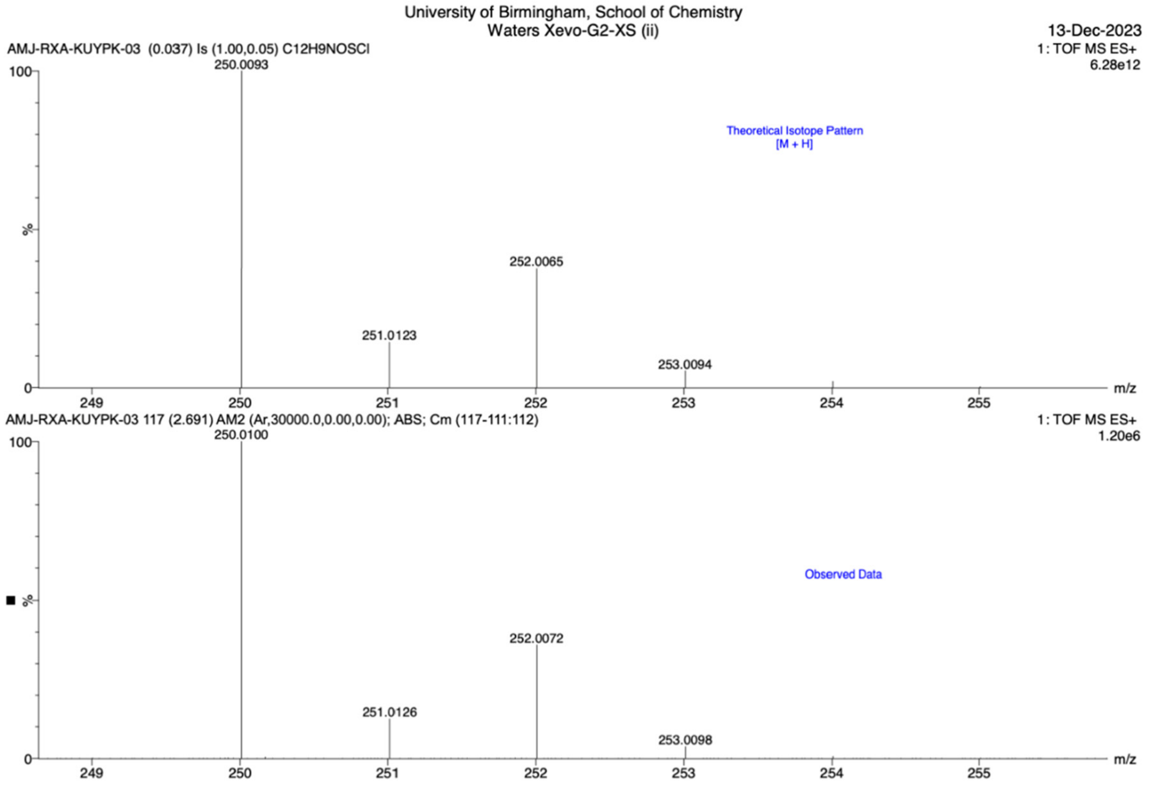
University of Birmingham, School of Chemistry
Waters Xevo-G2-XS (ii)
13-Dec-2023
AMJ-RXA-KUYPK-03 (0.037) Is (1.00,0.05) C12H9NOSCl
1: TOF MS ES+
6.28e12
Theoretical Isotope Pattern
[M + H]
250.0093
252.0065
251.0123
253.0094
AMJ-RXA-KUYPK-03 117 (2.691) AM2 (Ar,30000.0,0.00,0.00); ABS; Cm (117-111:112)
1: TOF MS ES+
1.20e6
Observed Data
250.0100
252.0072
251.0126
253.0098
%
m/z

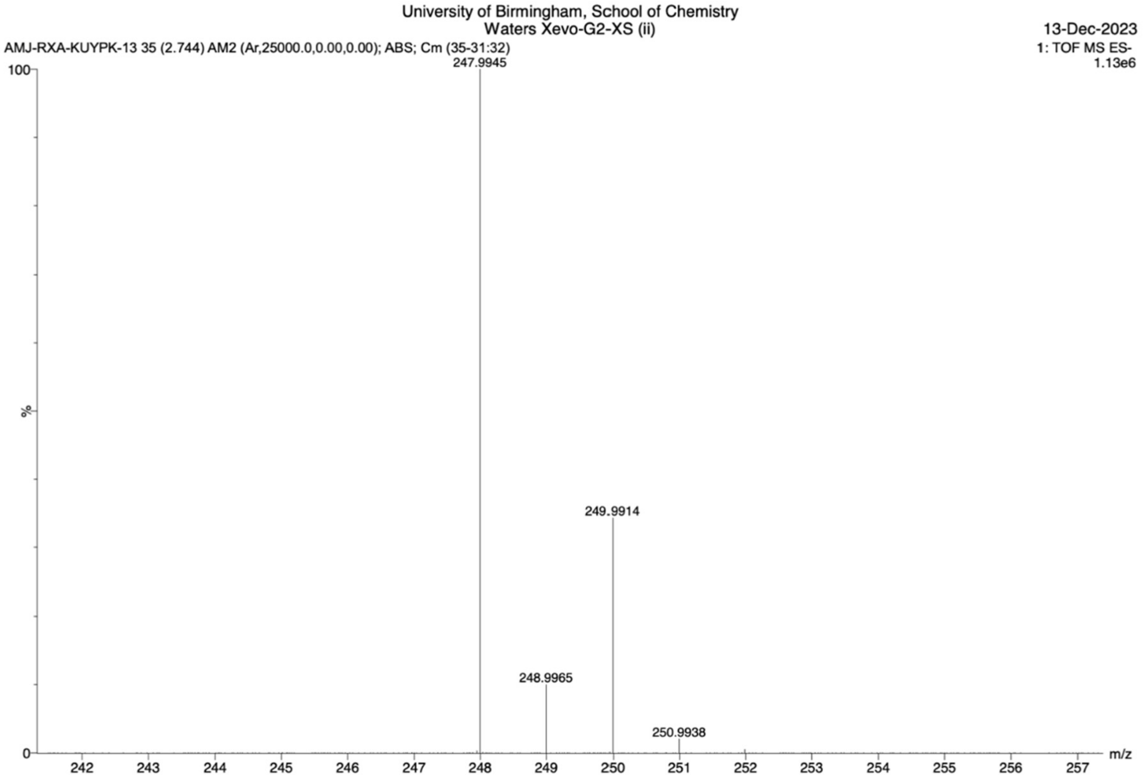
University of Birmingham, School of Chemistry
Waters Xevo-G2-XS (ii)
AMJ-RXA-KUYPK-13 35 (2.744) AM2 (Ar,25000.0,0.00,0.00); ABS; Cm (35-31:32)
13-Dec-2023
1: TOF MS ES-
1.13e6
247.9945
249.9914
248.9965
250.9938
%
m/z

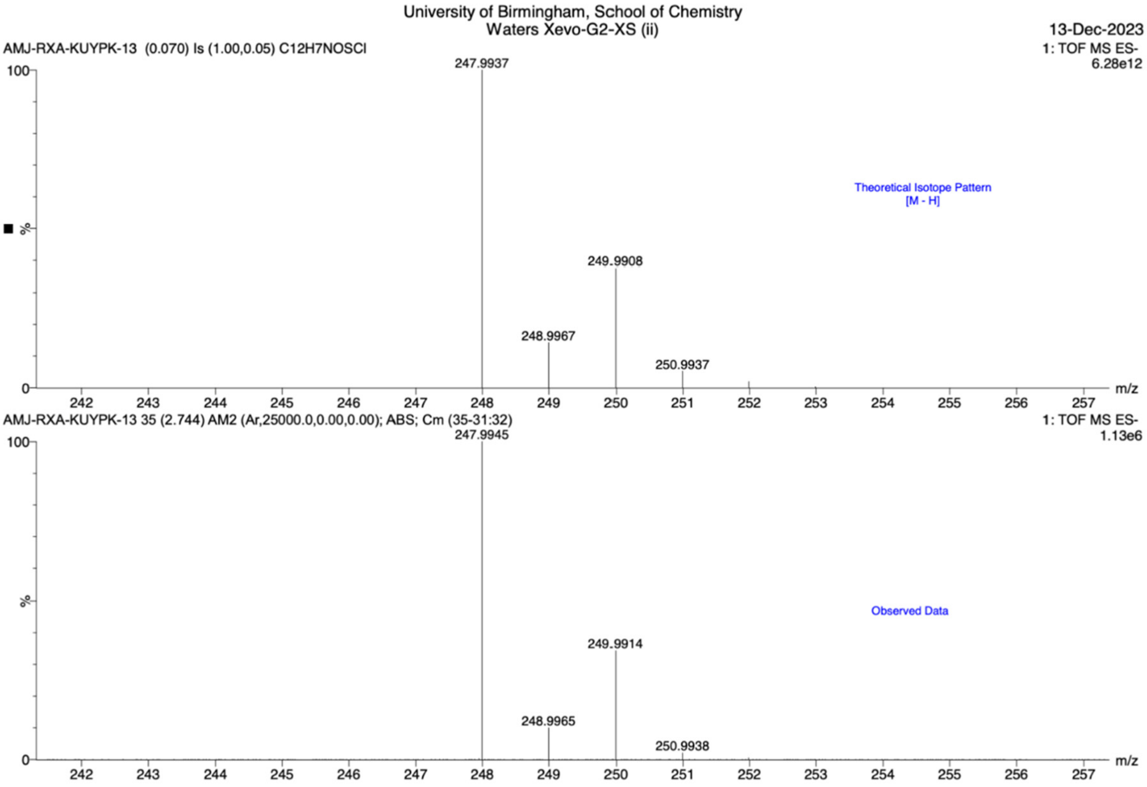
University of Birmingham, School of Chemistry
Waters Xevo-G2-XS (ii)
AMJ-RXA-KUYPK-13 (0.070) Is (1.00,0.05) C12H7NOSCl
13-Dec-2023
1: TOF MS ES-
6.28e12
247.9937
Theoretical Isotope Pattern
[M - H]
249.9908
248.9967
250.9937
AMJ-RXA-KUYPK-13 35 (2.744) AM2 (Ar,25000.0,0.00,0.00); ABS; Cm (35-31:32)
1: TOF MS ES-
1.13e6
247.9945
Observed Data
249.9914
248.9965
250.9938
%
m/z

3 2CPTZ-SO_2 molecular weight 265 (2.95 min, LCMS **ES-** 263.9906, **ES+** 266.0070)

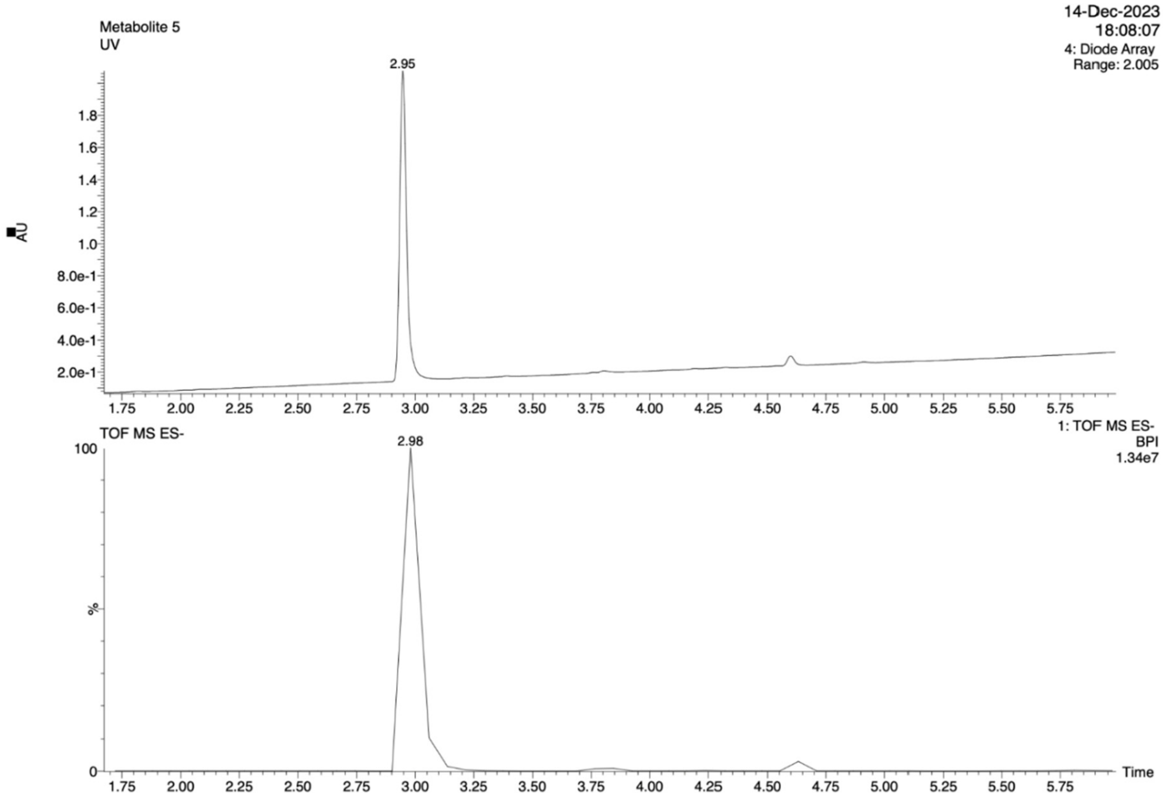

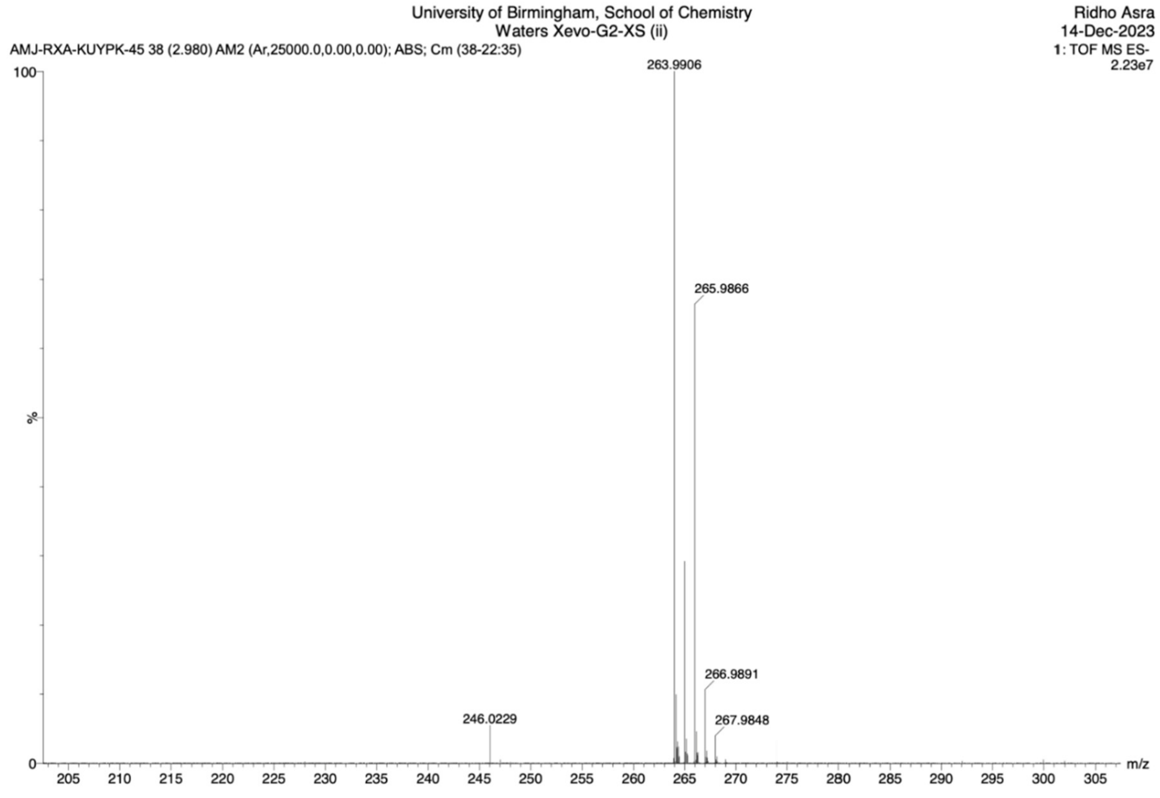

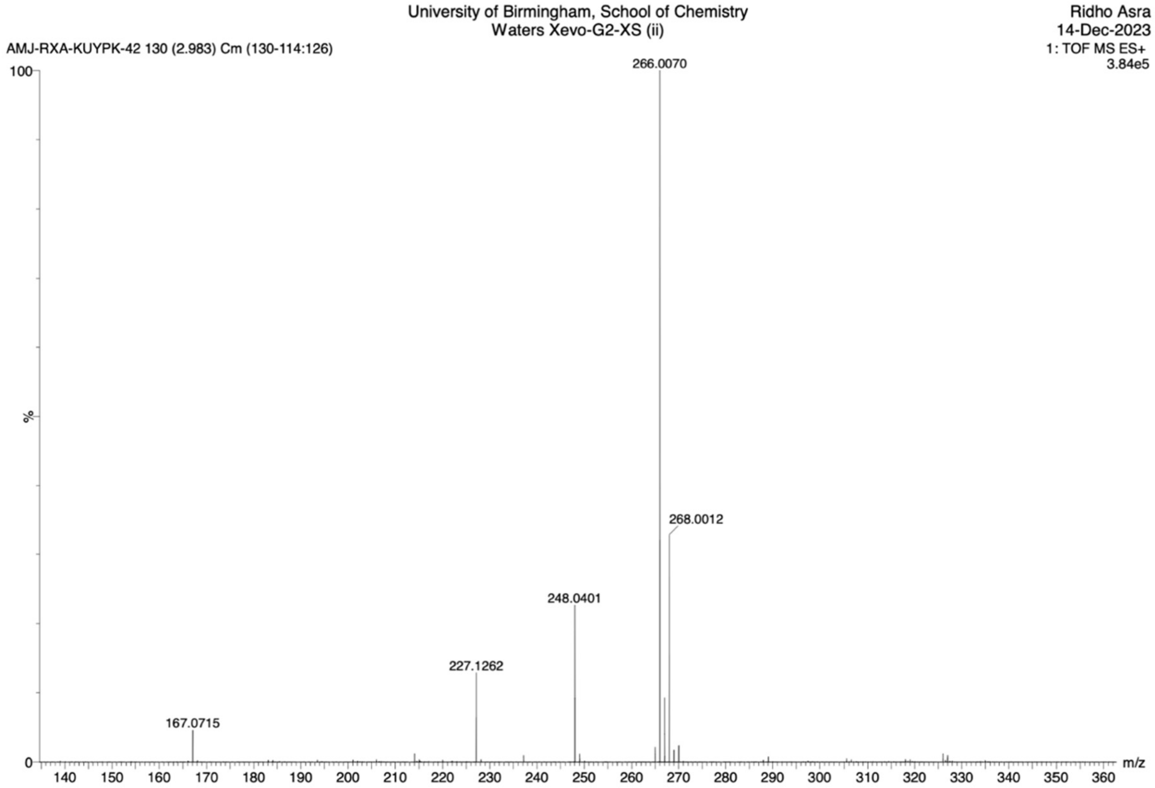

University of Birmingham, School of Chemistry
Waters Xevo-G2-XS (ii)
Ridho Asra
14-Dec-2023
AMJ-RXA-KUYPK-42 130 (2.983) Cm (130-114:126)
1: TOF MS ES+
3.84e5
266.0070
268.0012
248.0401
227.1262
167.0715
%
m/z

b. Chlorpromazine Metabolites

6 CPZ-SO molecular weight 334 (LRMS 1.885 min, LCMS ES+ 335.1009)

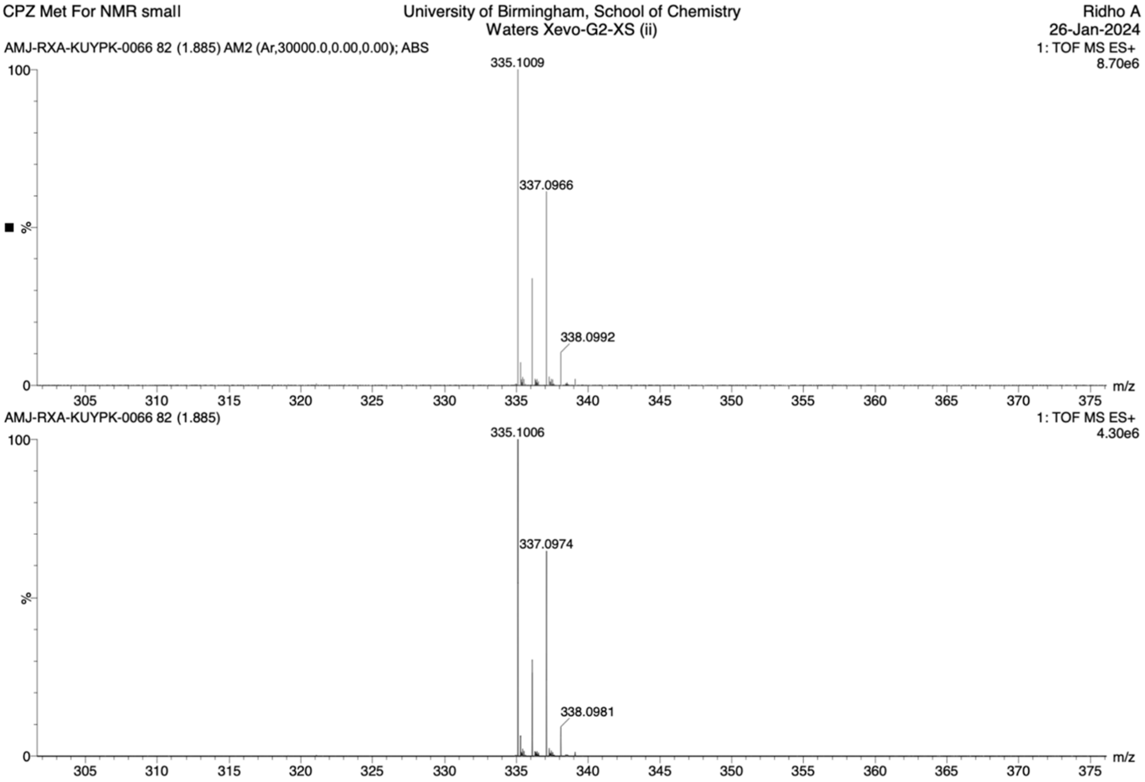

7 CPZ-SO_2 molecular weight 350 (LRMS 2.068 min, LCMS **ES+** 351.0937)

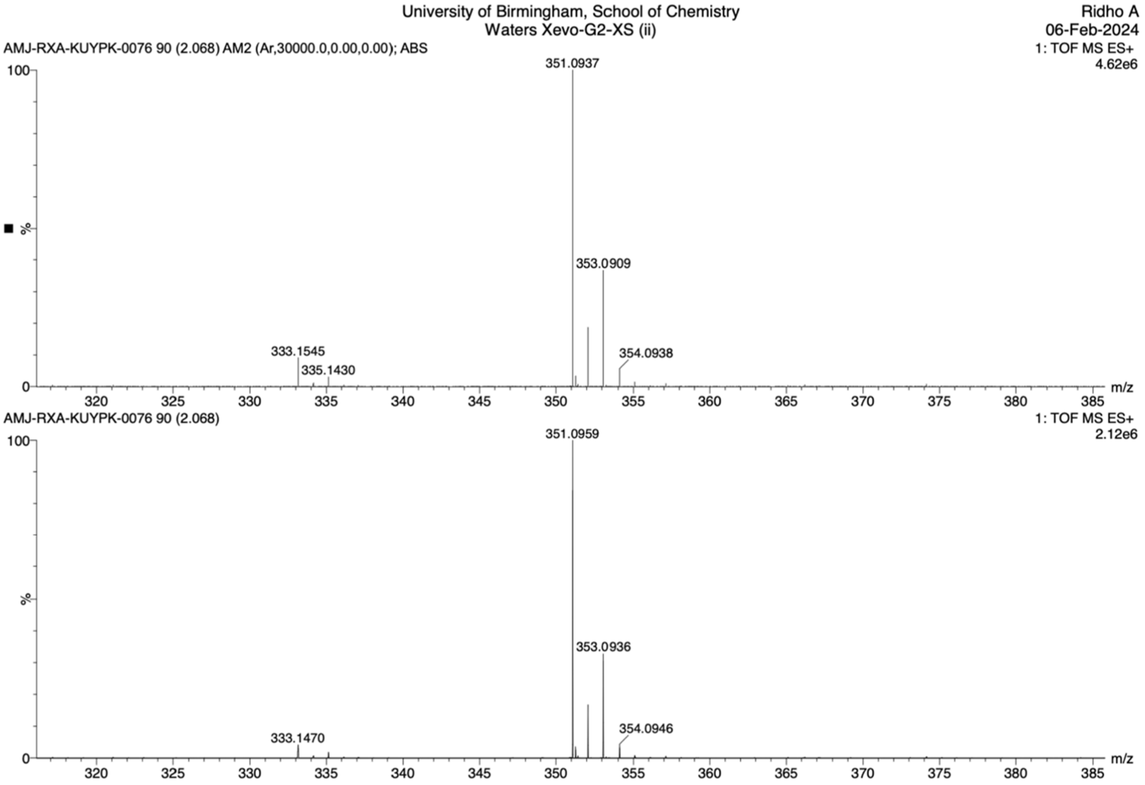

CPZ Metabolite 3 molecular weight 368 (LRMS 2.159 min, LCMS ES+ 369.0600)

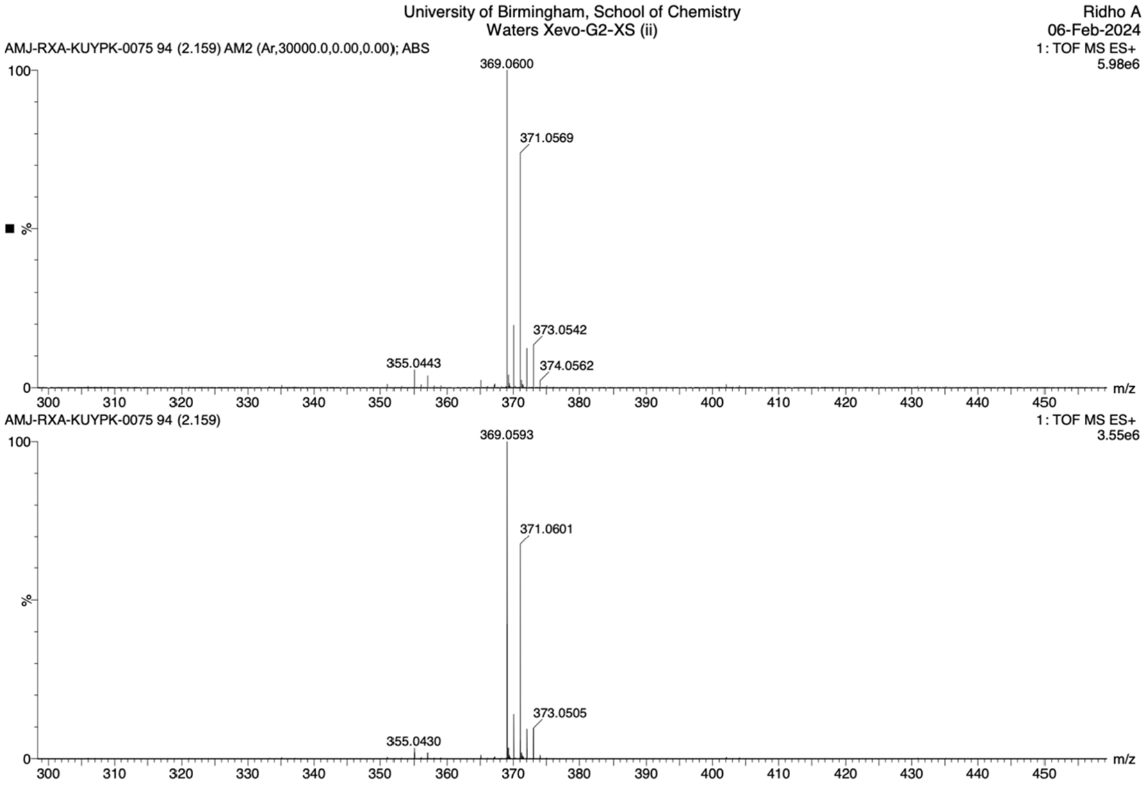

CPZ Metabolite 4 molecular weight 185 (LRMS 2.279 min, LCMS ES+ 186.2199)

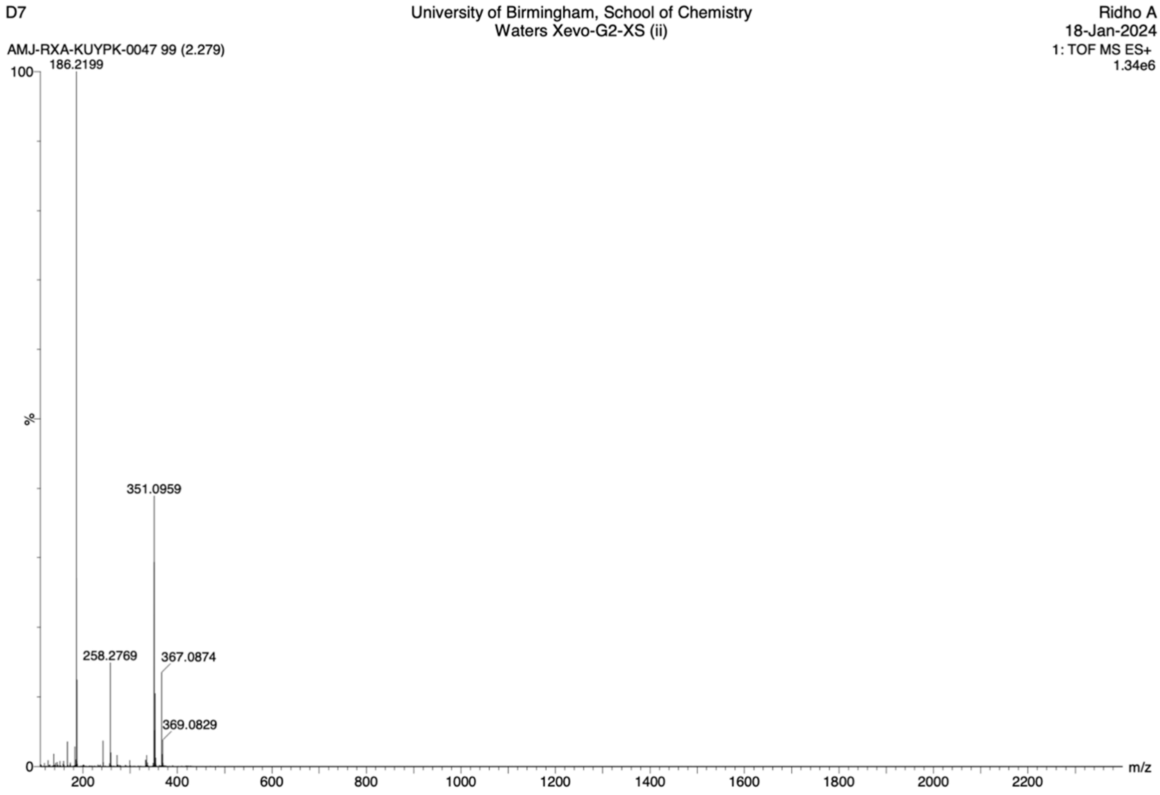

CPZ Metabolite 5 molecular weight 249 (LRMS 2.508 min, LCMS ES- 247.9941)

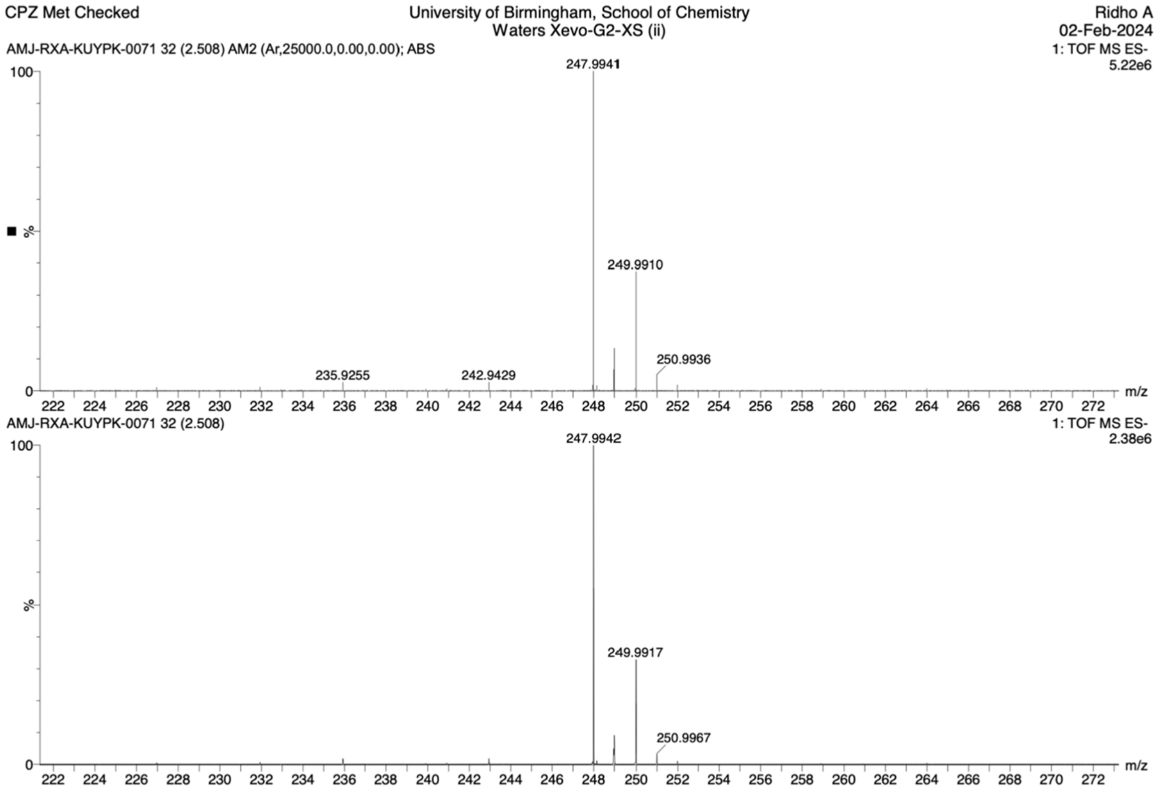

CPZ Metabolite 6 molecular weight 348 (LRMS 2.737 min, LCMS ES+ 349.0808)

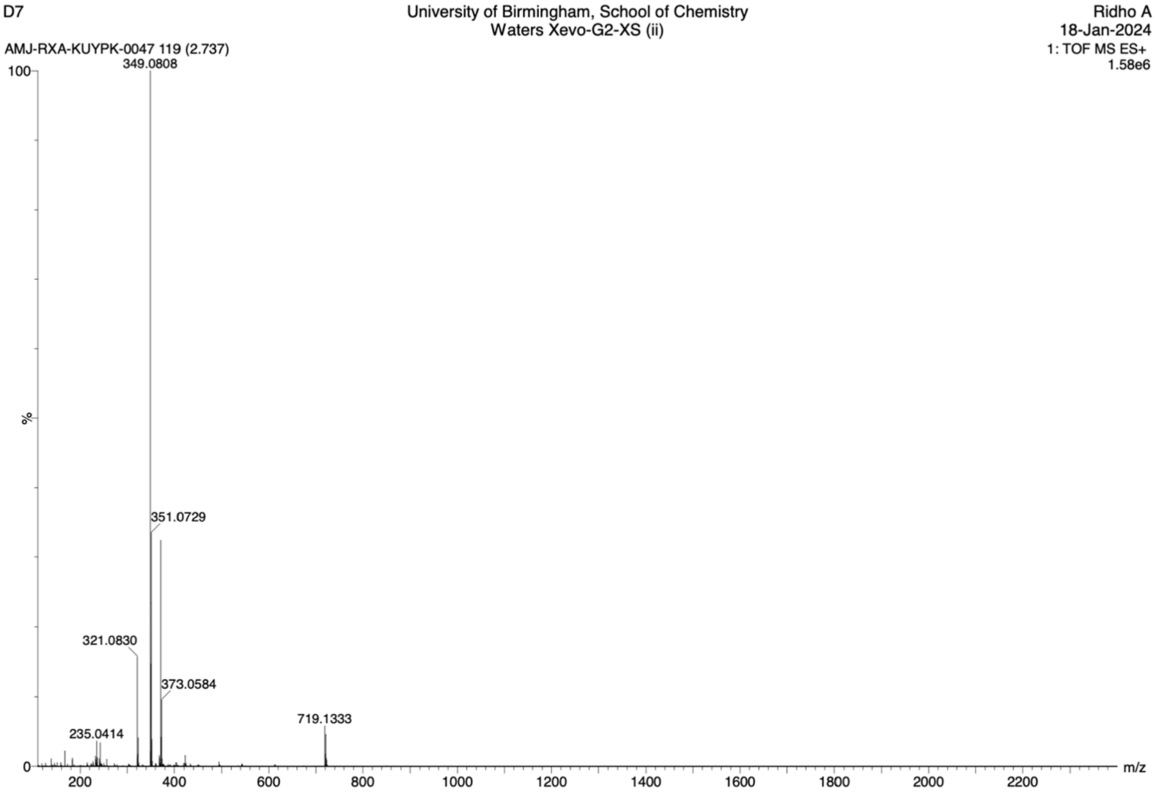

CPZ Metabolite 7 molecular weight 242 (LRMS 2.800 min, LCMS ES+ 243.2926)

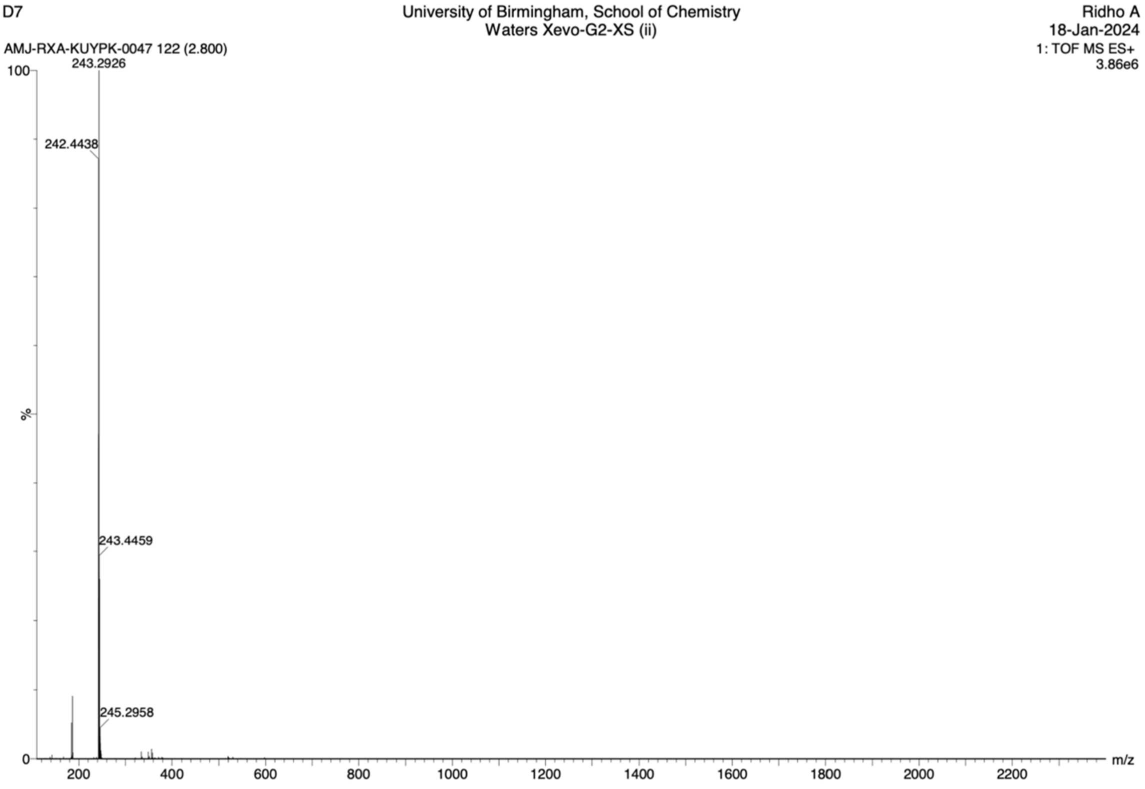

IR Data

a. 2-Chlorophenothiazine Metabolites

1 2CPTZ

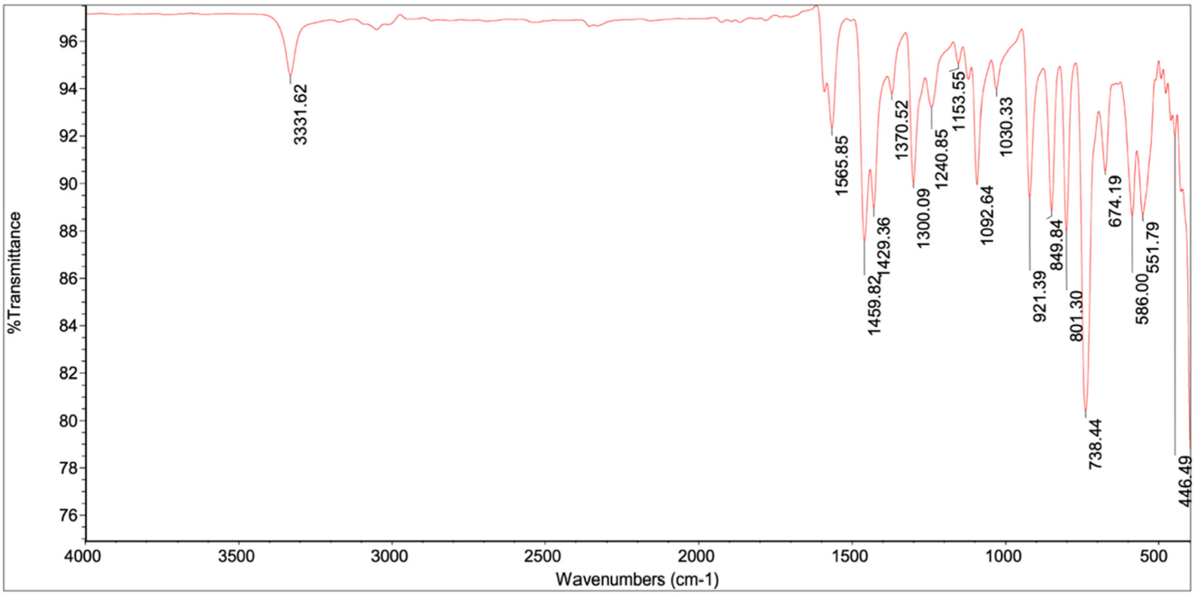

2 2CPTZ-SO

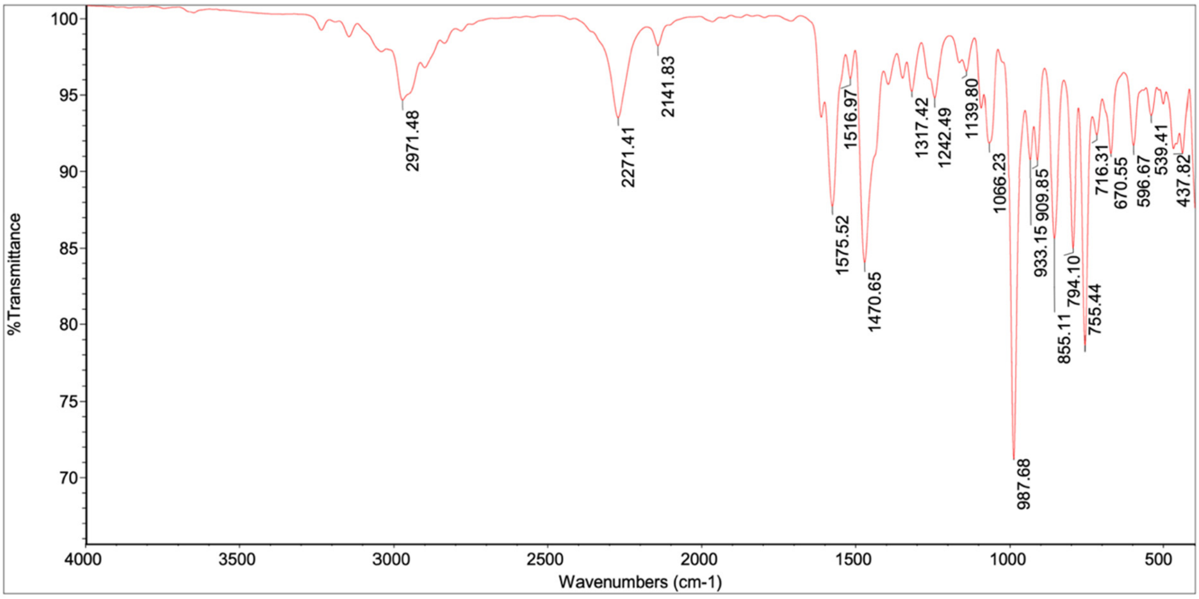

3 2CPTZ-SO_2 (in 2-propanol)

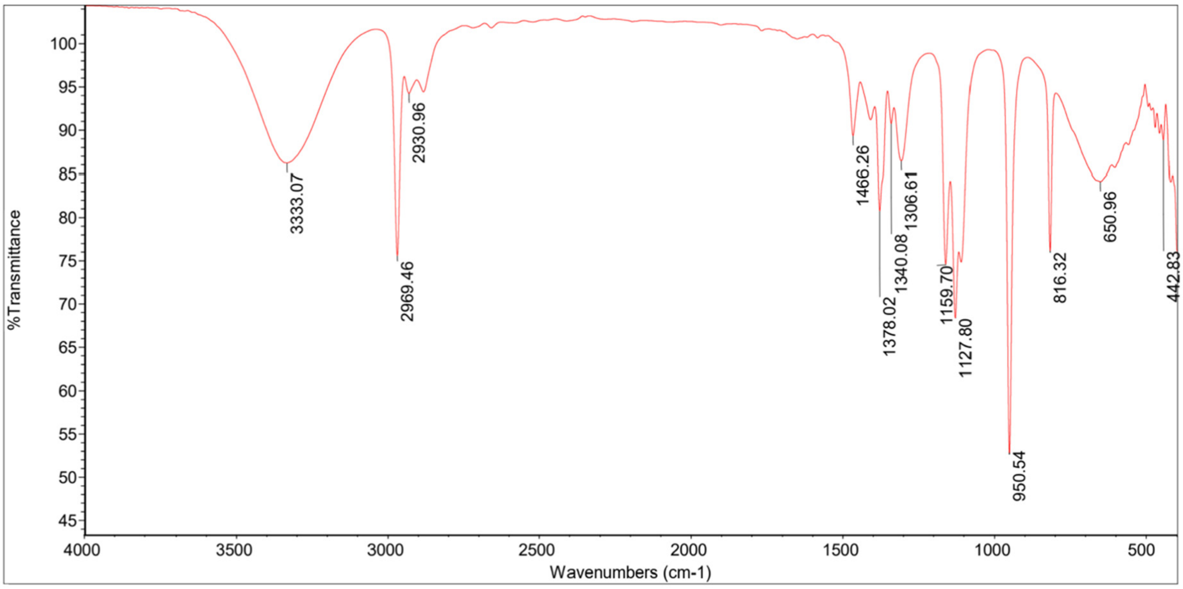

b. Chlorpromazine Metabolites

5 CPZ

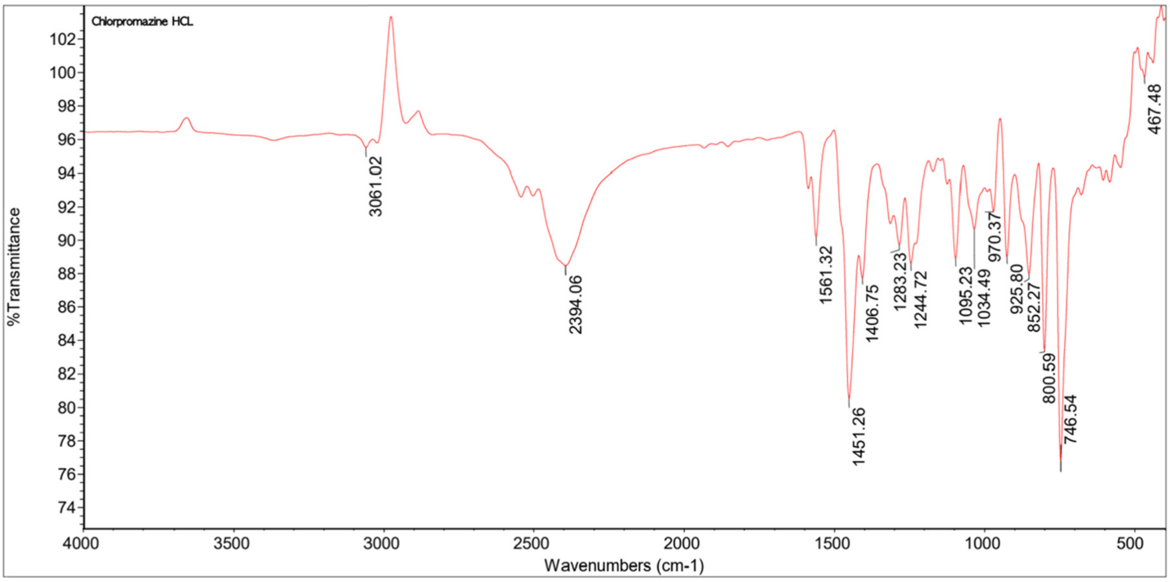

6 CPZ-SO

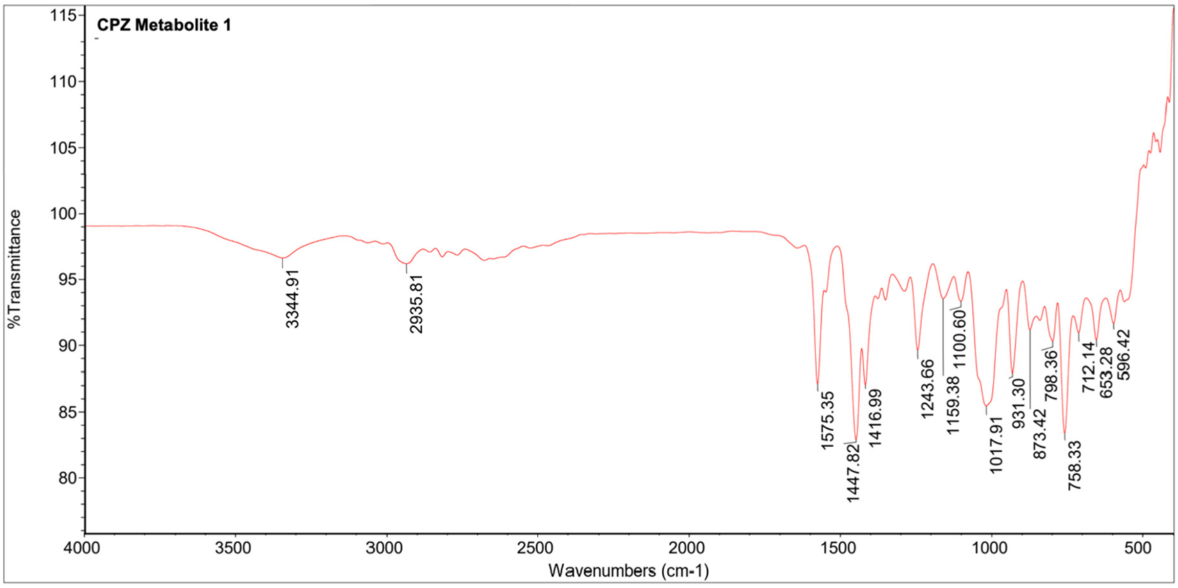

7 CPZ-SO_2

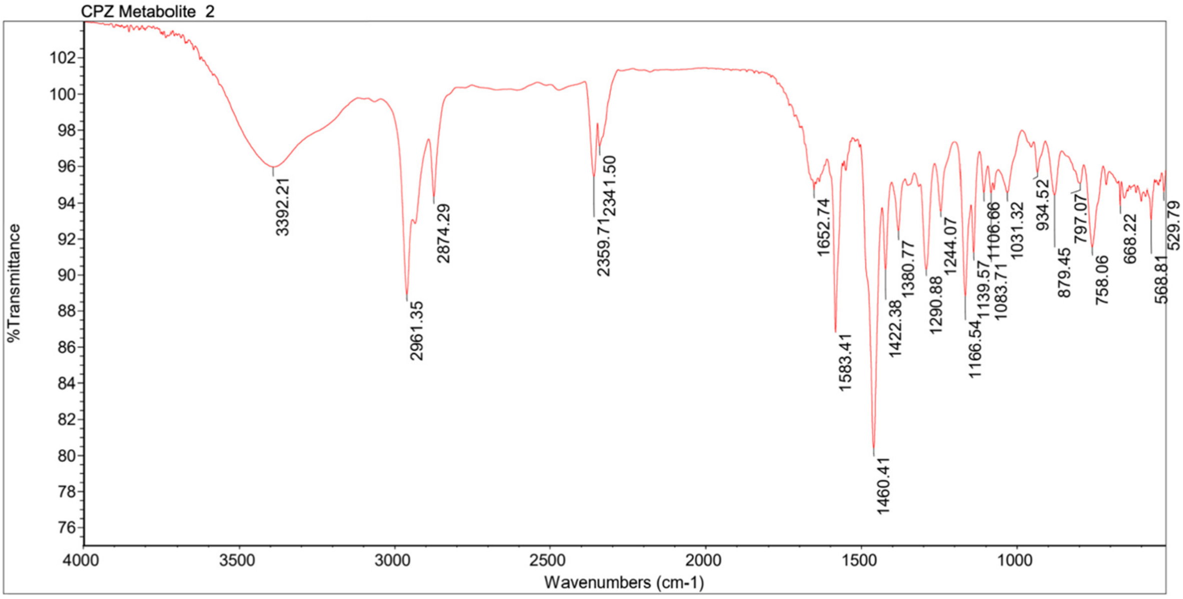

HPLC-LCMS 2-Chlorophenothiazine Fractions

Metabolite 3 (HPLC RT 12.308 min, MW: 277.9881)

HPLC retention time: 12.308, LCMS ES-: 276.9808, LCMS ES-: actual molecular weight (amu) = MW from LCMS ES-(amu) + Mass of proton (1.0073 amu) = **277.9881**

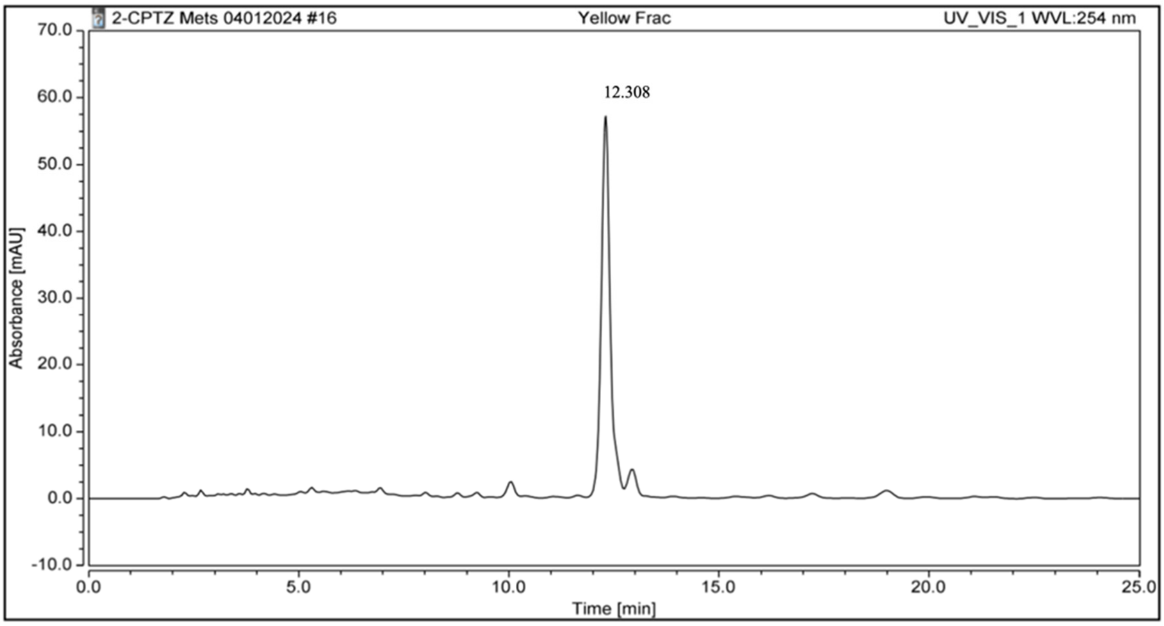

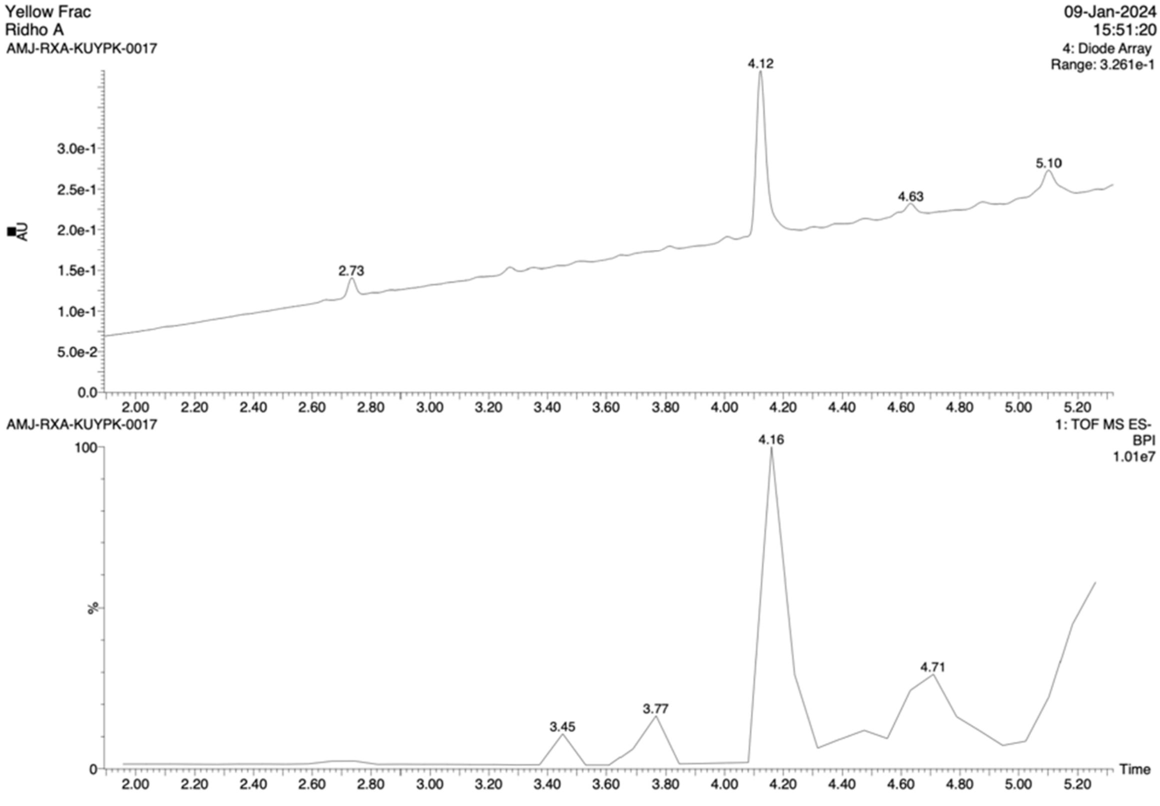

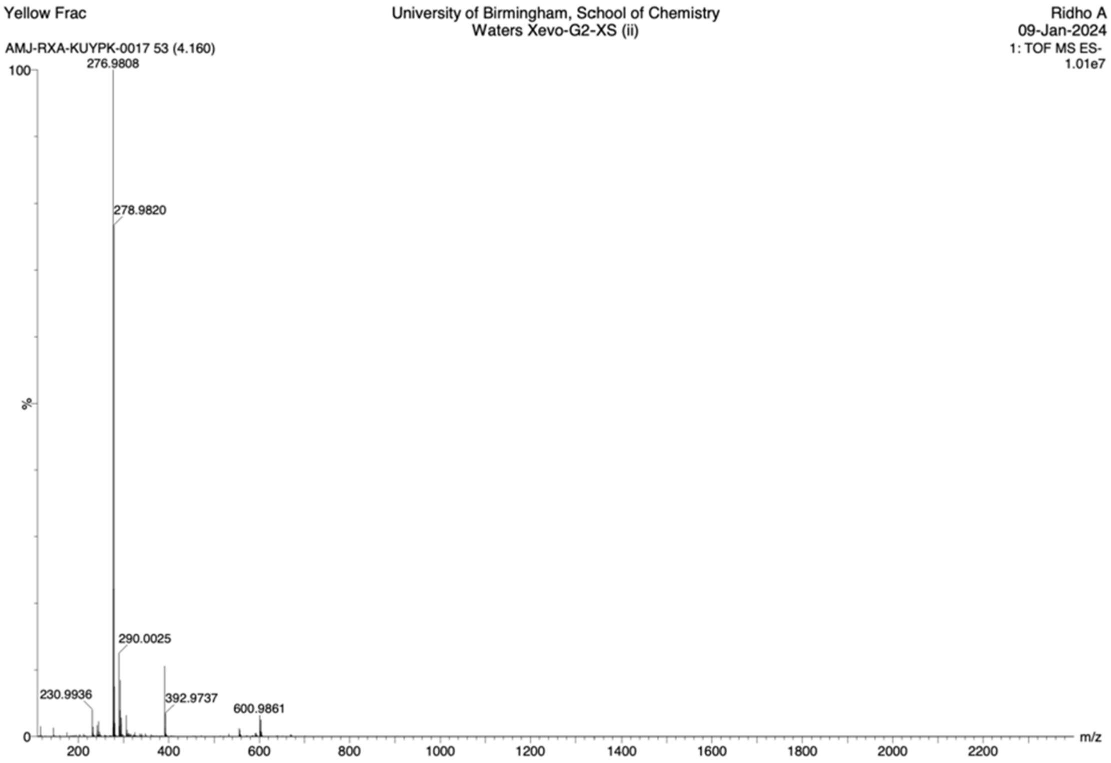
Yellow Frac
University of Birmingham, School of Chemistry
Waters Xevo-G2-XS (ii)
Ridho A
09-Jan-2024
AMJ-RXA-KUYPK-0017 53 (4.160)
1: TOF MS ES-
1.01e7
276.9808
278.9820
290.0025
230.9936
392.9737
600.9861
%
m/z

Metabolite 4 (HPLC RT 5.142 min, MW: 187.2502)

MW ES-(2.64 min): 186.2429, actual molecular weight (amu) = MW from LCMS ES-(amu) + Mass of proton (1.0073 amu) = **187.2502**

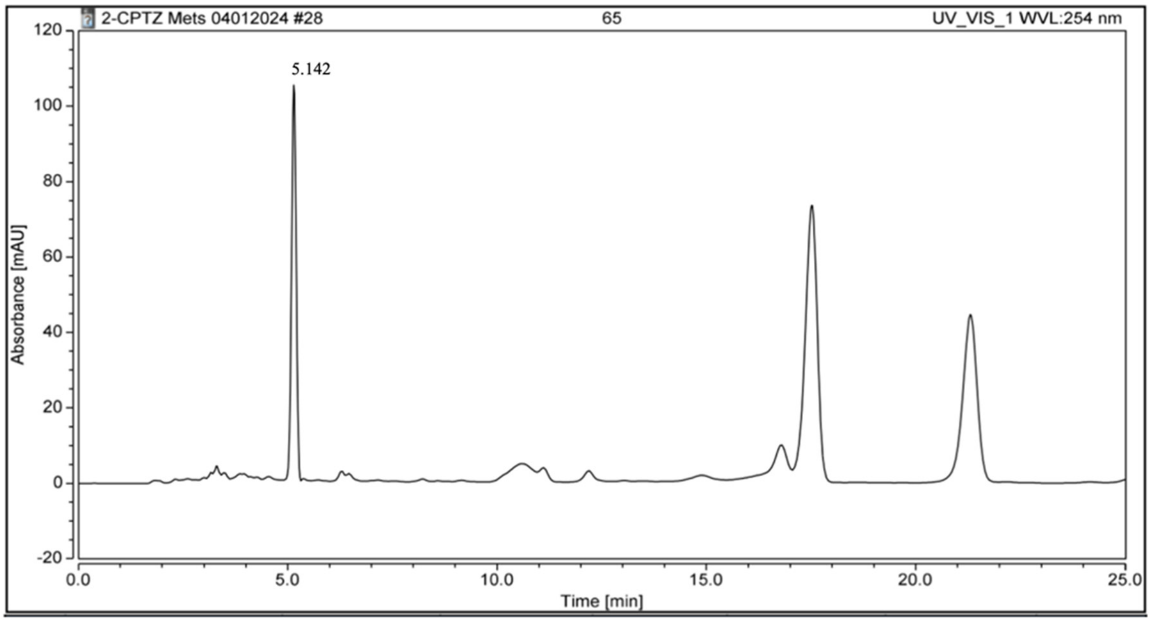

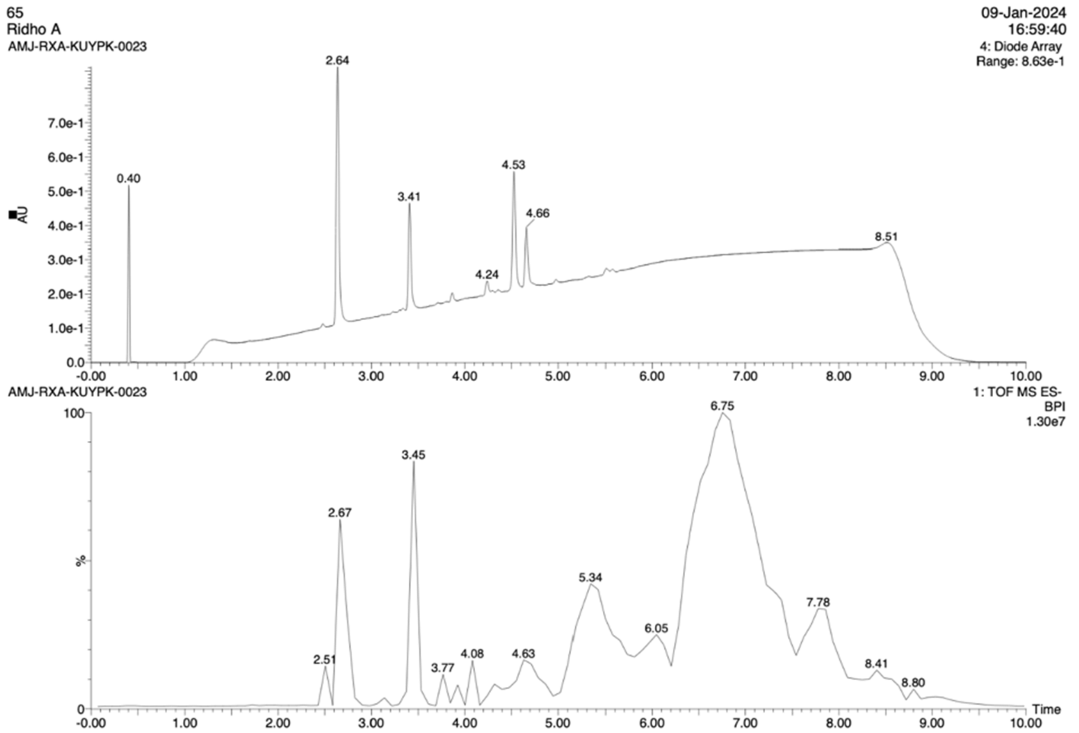

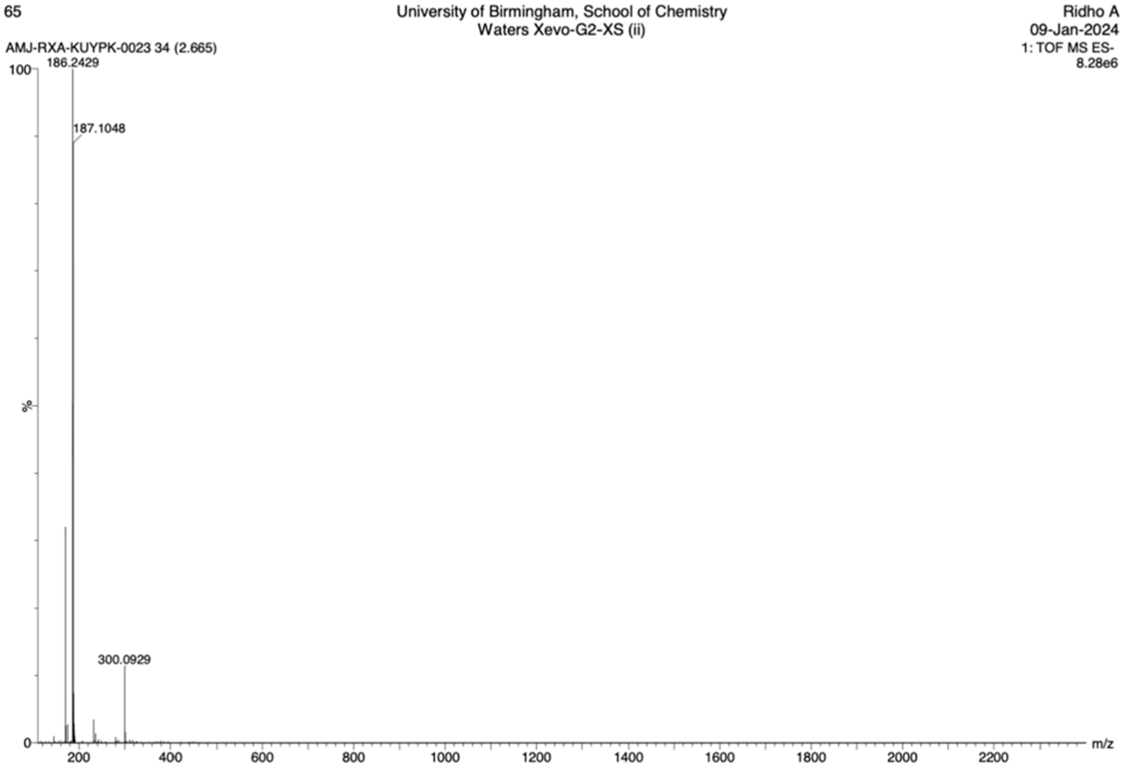
65
University of Birmingham, School of Chemistry
Waters Xevo-G2-XS (ii)
Ridho A
09-Jan-2024
AMJ-RXA-KUYPK-0023 34 (2.665)
1: TOF MS ES-
8.28e6
186.2429
187.1048
300.0929
%
m/z

Metabolite 5 (HPLC RT 10.608 min, MW: 318.9214)

MW ES-(3.41 min): 317.9141, actual molecular weight (amu) = MW from LCMS ES-(amu) + Mass of proton (1.0073 amu) = **318.9214**

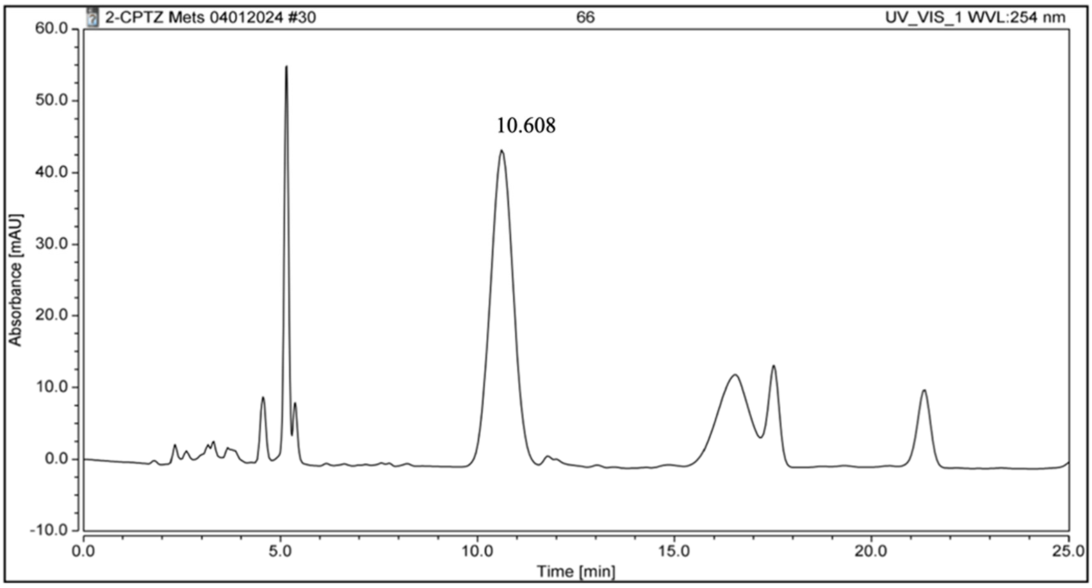

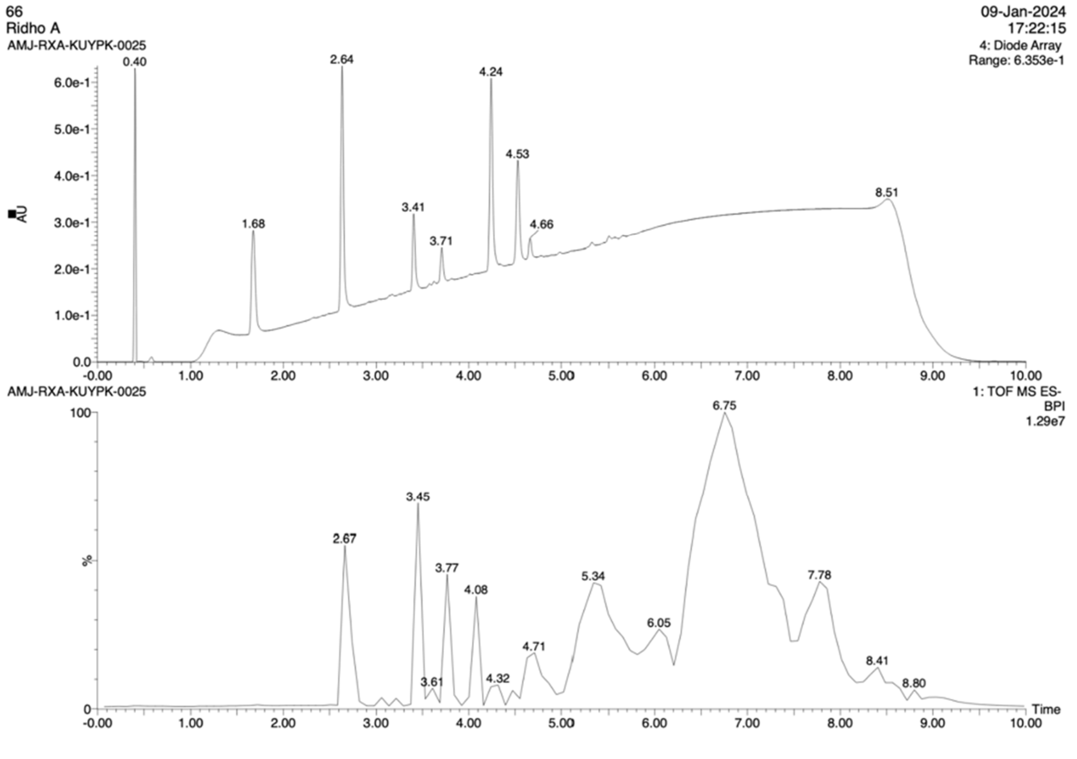

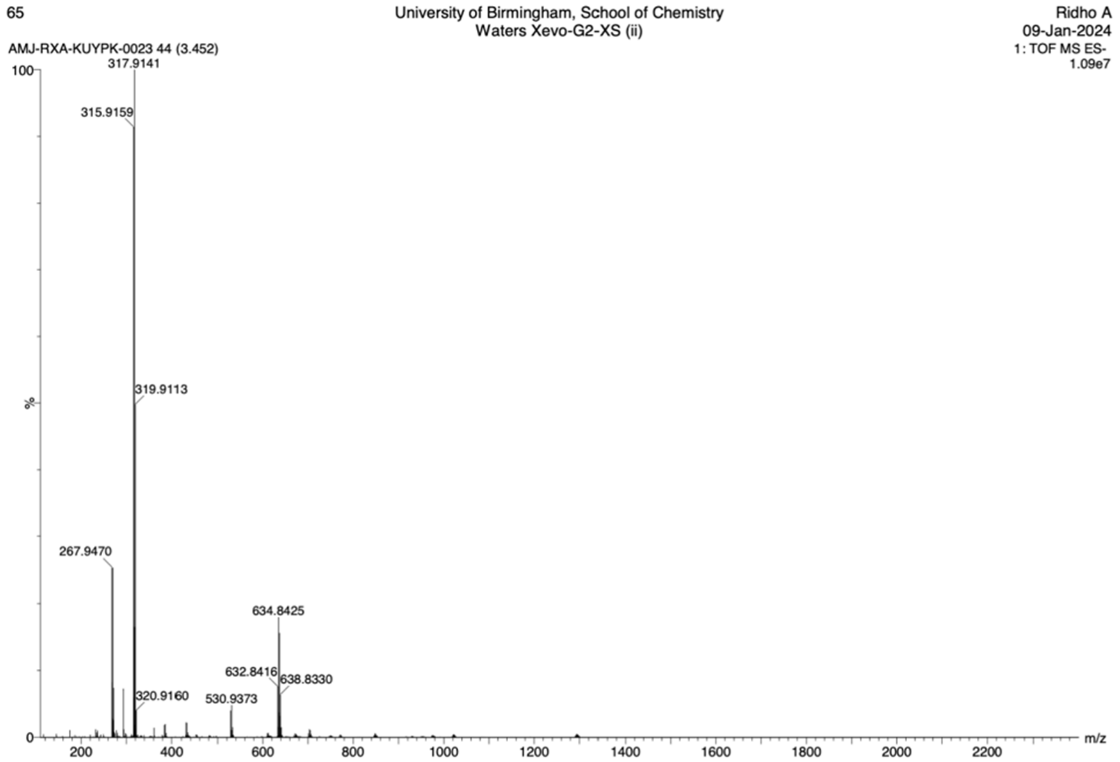

65
University of Birmingham, School of Chemistry
Waters Xevo-G2-XS (ii)
Ridho A
09-Jan-2024
AMJ-RXA-KUYPK-0023 44 (3.452)
1: TOF MS ES-
1.09e7
100
%
0
317.9141
315.9159
319.9113
267.9470
320.9160
530.9373
632.8416
634.8425
638.8330
200
400
600
800
1000
1200
1400
1600
1800
2000
2200
m/z

Cyclic voltammetry data
2-Chlorophenothiazine

Preliminary Analysis
Blank/control solution analysis
(0.1 M tetrabutylammonium hexafluorophosphate in 10 mL MeCN)

CV staircase setting:
Start potential: 0 Vreff
Upper Vertex Potential: 3.00 Vreff
Lower Vertex Potential: -0.10Vreff
Stop Potential: 0 Vreff
Number of Scans: 1
Scan Rate: 0.10 V/s
Step: 0.00244 V

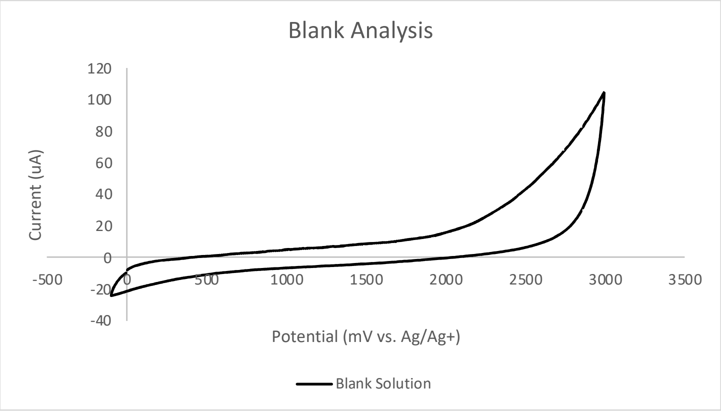

Reference solution
(1 mM ferrocene and 0.1 M tetrabutylammonium hexafluorophosphate in 10 mL MeCN)

CV staircase setting:
Start potential: 0 Vreff
Upper Vertex Potential: 0.80 Vreff
Lower Vertex Potential: -0.20Vreff
Stop Potential: 0 Vreff
Number of Scans: 1
Scan Rate: 0.10 V/s
Step: 0.00244 V

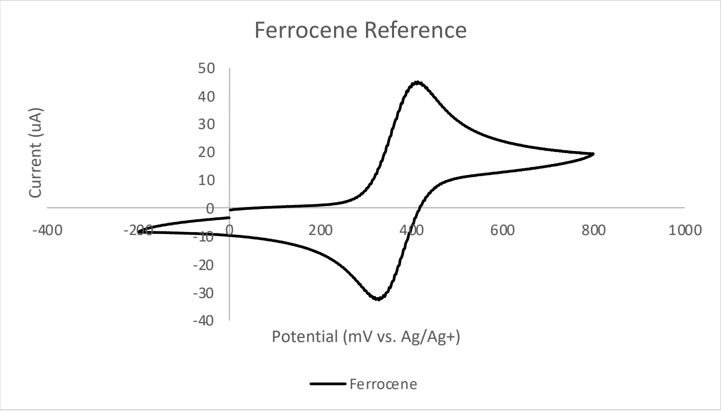

Ep 0.415 V; Ep_2 0.332 V; $E_{1/2}$ 0.37 V

Potential Range of 2-Chlorophenothiazine
CV staircase setting:
Start potential: 0 Vreff,
Upper Vertex Potential: 3.00 Vreff Lower Vertex Potential: -0.10 Vreff
Stop Potential: 0 Vreff
Number of Scans: 1
Scan Rate: 0.10 V/s
Step: 0.00244 V

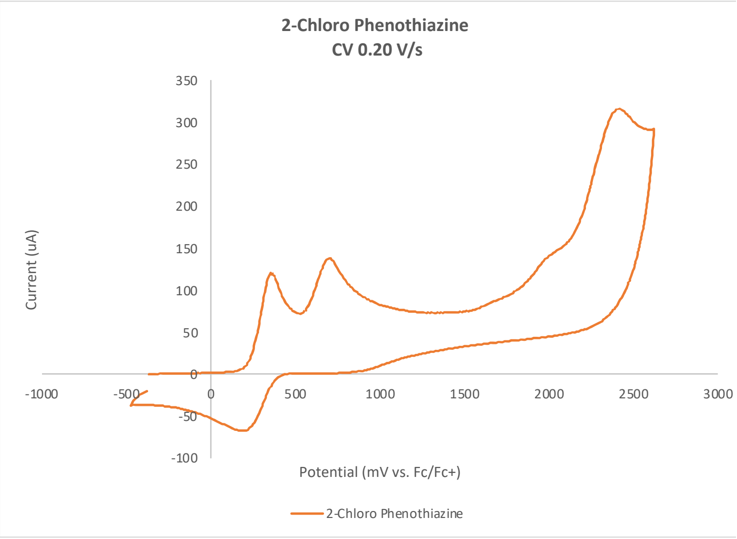

[1]Ep 0.346 V; ip 116 μA; Ep_2 0.200 V; ip -68 μA; $E_{1/2}$ 0.27 V

[2]Ep 0.690 V; ip 137 μA. [3]Ep 1.675 V; ip 87 μA. [4]Ep 2.00 V; ip 141 μA. [5]Ep 2.40 V; ip 316 μA

Cyclic Voltammetry Studies of 2-Chlorophenothiazine

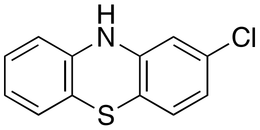

CV staircase setting:
Start potential: 0 Vreff,
Upper Vertex Potential: 1.40 Vreff Lower Vertex Potential: -0.10Vreff
Stop Potential: 0 Vreff
Number of Scans: 1
Scan Rate: 0.20 V/s
Step: 0.00244 V

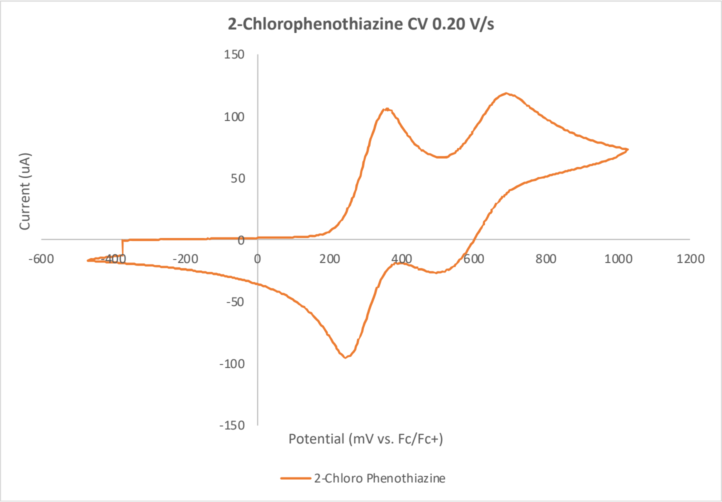

[1]Ep 0.353 V; ip 105 μA; Ep_2 0.246 V; ip -96 μA; $E_{1/2}$ 0.12 V
[2]Ep 0.702 V; ip 118 μA; Ep_2 0.514 V; ip -26 μA; $E_{1/2}$ 0.61 V

(2-(4-(6-(2-chloro-10H-phenothiazin-10-yl) hexyl) piperazin-1- yl) ethan-1-ol)

Preliminary Analysis
Blank/control solution analysis
(0.1 M tetrabutylammonium hexafluorophosphate in 10 mL MeCN)

CV staircase setting:
Start potential: 0 Vreff
Upper Vertex Potential: 3.00 Vreff
Lower Vertex Potential: -0.10 Vreff Stop Potential: 0 Vreff
Number of Scans: 1
Scan Rate: 0.20 V/s
Step: 0.00244 V

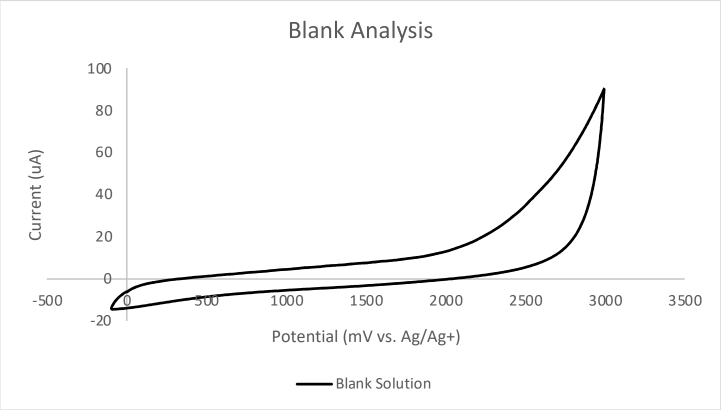

Reference solution
(1 mM ferrocene and 0.1 M tetrabutylammonium hexafluorophosphate in 10 mL MeCN)

CV staircase setting:
Start potential: 0 Vreff
Upper Vertex Potential: 0.90 Vreff
Lower Vertex Potential: -0.20Vreff
Stop Potential: 0 Vreff
Number of Scans: 1
Scan Rate: 0.10 V/s
Step: 0.00244 V

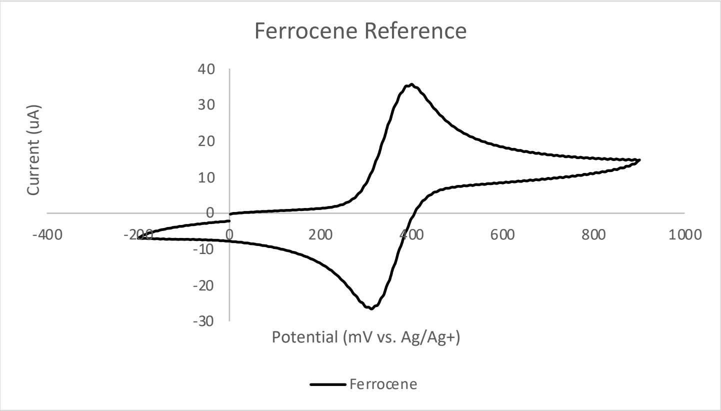

Ep 0.400 V; Ep_2 0.315 V; $E_{1/2}$ 0.36 V

Cyclic Voltammetry Studies of AiM 16

Chemical Formula: $C_{24}H_{32}ClN_3OS$
Molecular Weight: 446.05

(2 mg (2-(4-(6-(2-chloro-10H-phenothiazin-10-yl) hexyl) piperazin-1- yl) ethan-1-ol) and 0.1 M tetrabutylammonium hexafluorophosphate in 10 mL MeCN)

CV staircase setting:
Start potential: 0 Vreff
Upper Vertex Potential: 2.00 Vreff
Lower Vertex Potential: -0.10Vreff
Stop Potential: 0 Vreff

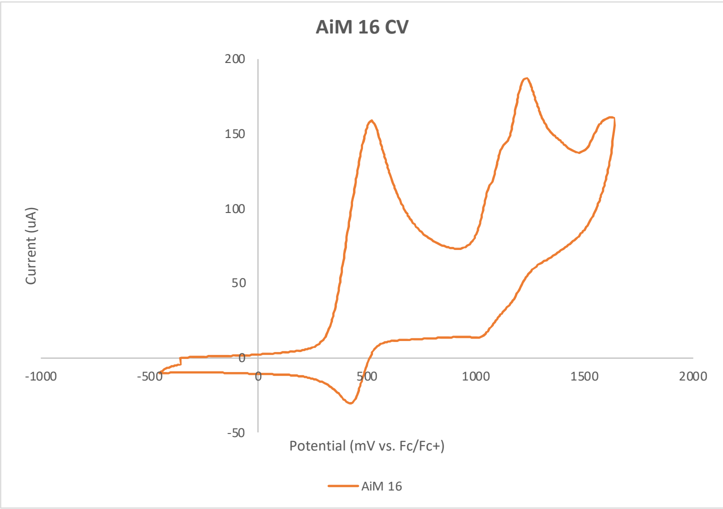

Number of Scans: 1
Scan Rate: 0.20 V/s
Step: 0.00244 V

[1]Ep 0.513 V; ip 158 μA; Ep_2 0.427 V; ip -31 μA; $E_{1/2}$ 0.48 V
[2]Ep 1.059 V; ip 114 μA. [3]Ep 1.144 V; ip 144 μA. [4]Ep 1.222 V; ip 186 μA.

(2-(4-(6-(2-chloro-10H-phenothiazin-10-yl) hexyl) piperazin-1- yl) ethan-1-ol)CV Multiple Scanning
CV staircase setting:
Start potential: 0 Vreff
Upper Vertex Potential: 2.00 Vreff
Lower Vertex Potential: -0.10Vreff
Stop Potential: 0 Vreff
Number of Scans: 2
Scan Rate: 0.20 V/s
Step: 0.00244 V

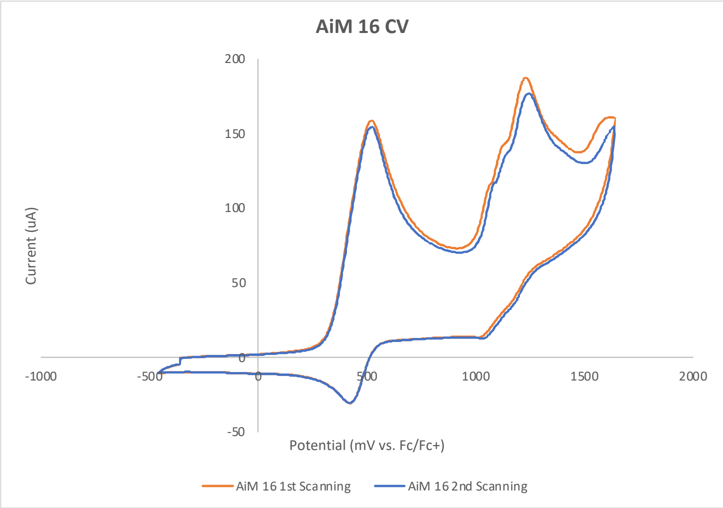

Potential Range of (2-(4-(6-(2-chloro-10H-phenothiazin-10-yl) hexyl) piperazin-1- yl) ethan-1-ol)
CV staircase setting:
Start potential: 0 Vreff
Upper Vertex Potential: 3.00 Vreff
Lower Vertex Potential: -0.10Vreff
Stop Potential: 0 Vreff
Number of Scans: 1
Scan Rate: 0.20 V/s
Step: 0.00244 V

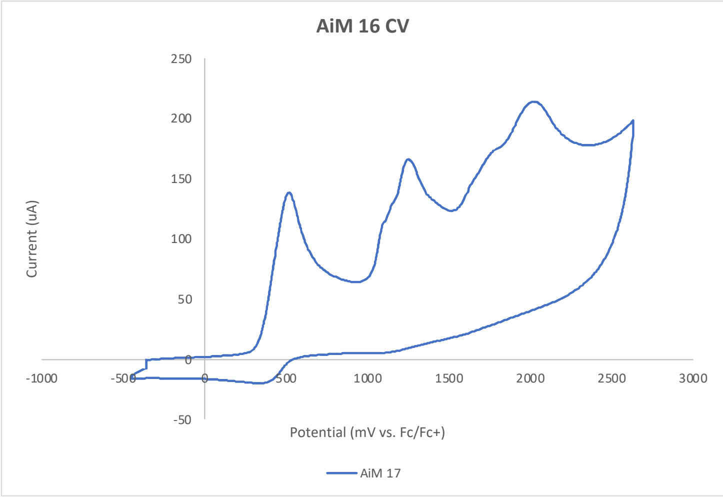

CV profile after Scanning up to 3 V/s

CV staircase setting:
Start potential: 0 Vreff
Upper Vertex Potential: 2.00 Vreff
Lower Vertex Potential: -0.10Vreff
Stop Potential: 0 Vreff
Number of Scans: 1
Scan Rate: 0.20 V/s
Step: 0.00244 V

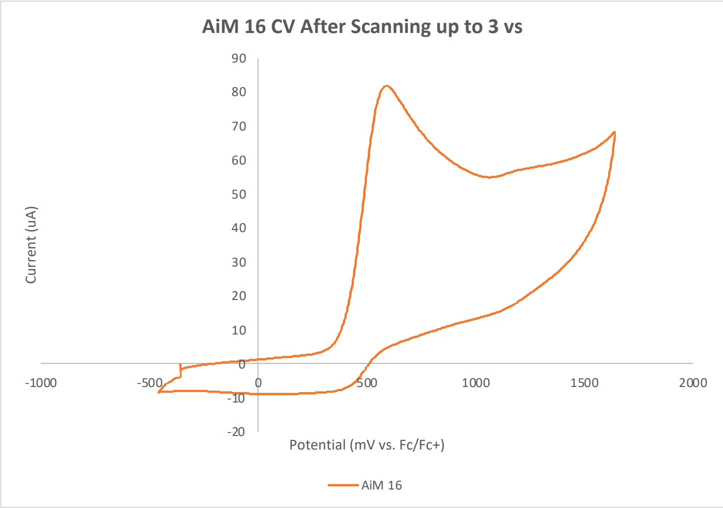

(6-(2-chloro-10H-phenothiazin-10-yl)-N,N-diethylhexan-1-amine)

Cyclic Voltammetry Studies of (6-(2-chloro-10H-phenothiazin-10-yl)-N,N-diethylhexan-1-amine)

Chemical Formula: $C_{22}H_{29}ClN_2S$
Molecular Weight: 389.00

(2 mg (6-(2-chloro-10H-phenothiazin-10-yl)-N,N-diethylhexan-1-amine)and 0.1 M tetrabutylammonium hexafluorophosphate in 10 mL MeCN)

CV staircase setting:
Start potential: 0 Vreff
Upper Vertex Potential: 2.00 Vreff
Lower Vertex Potential: -0.10Vreff
Stop Potential: 0 Vreff
Number of Scans: 1
Scan Rate: 0.20 V/s
Step: 0.00244 V

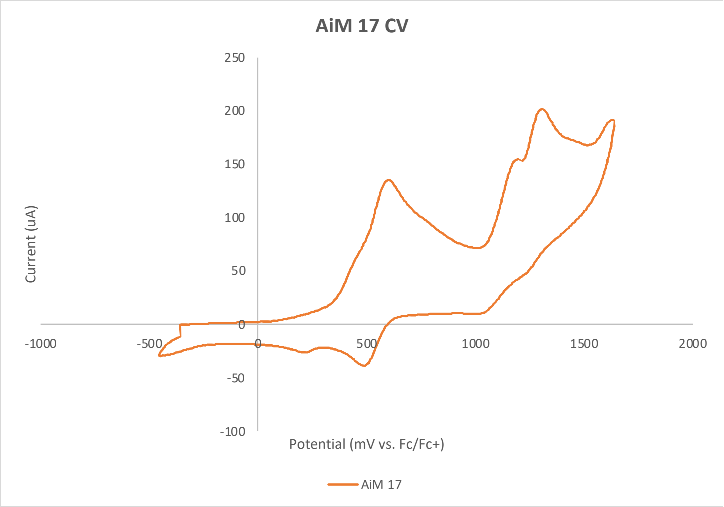

[1]Ep 0.437 V; ip 57 μA; Ep_2 0.223 V; ip -27 μA; $E_{1/2}$ 0.33 V
[2]Ep 0.591 V; ip 134 μA; Ep_2 0.491 V; ip -39 μA; $E_{1/2}$ 0.54 V
[3]Ep 1.181 V; ip 153 μA; Ep_2 1.039 V; ip 11 μA; $E_{1/2}$ 1.11 V
[4]Ep 1.298 V; ip 201 μA; Ep_2 1.237 V; ip 48 μA; $E_{1/2}$ 1.27 V

(6-(2-chloro-10H-phenothiazin-10-yl)-N,N-diethylhexan-1-amine) CV Multiple Scanning

CV staircase setting:
Start potential: 0 Vreff
Upper Vertex Potential: 2.00Vreff
Lower Vertex Potential: -0.10Vreff
Stop Potential: 0 Vreff
Number of Scans: 3
Scan Rate: 0.20 V/s
Step: 0.00244 V

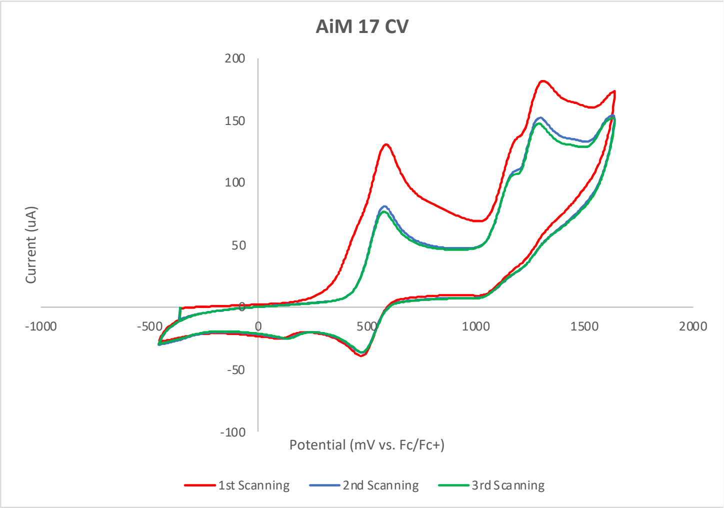

Potential Range of AiM 17

CV staircase setting:
Start potential: 0 Vreff
Upper Vertex Potential: 2.00 Vreff
Lower Vertex Potential: -0.10Vreff
Stop Potential: 0 Vreff
Number of Scans: 1
Scan Rate: 0.20 V/s
Step: 0.00244 V

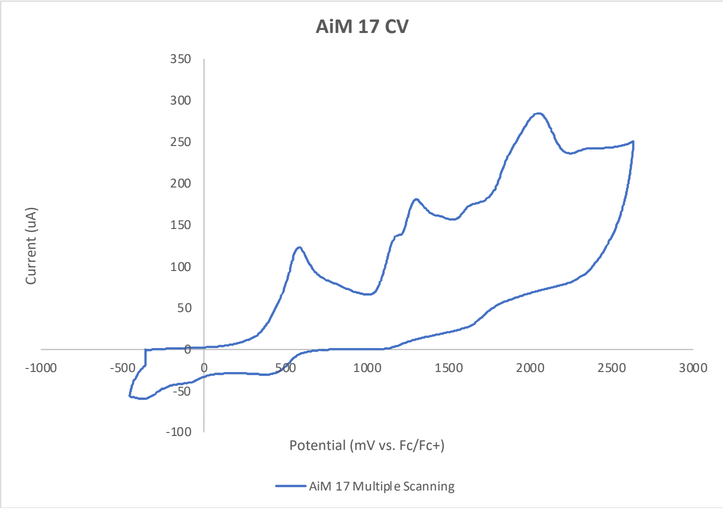

(6-(2-chloro-10H-phenothiazin-10-yl)-N,N-diethylhexan-1-amine) CV Multiple Scanning
CV staircase setting:
Start potential: 0 Vreff
Upper Vertex Potential: 2.00 Vreff
Lower Vertex Potential: -0.10Vreff
Stop Potential: 0 Vreff
Number of Scans: 3
Scan Rate: 0.20 V/s
Step: 0.00244 V

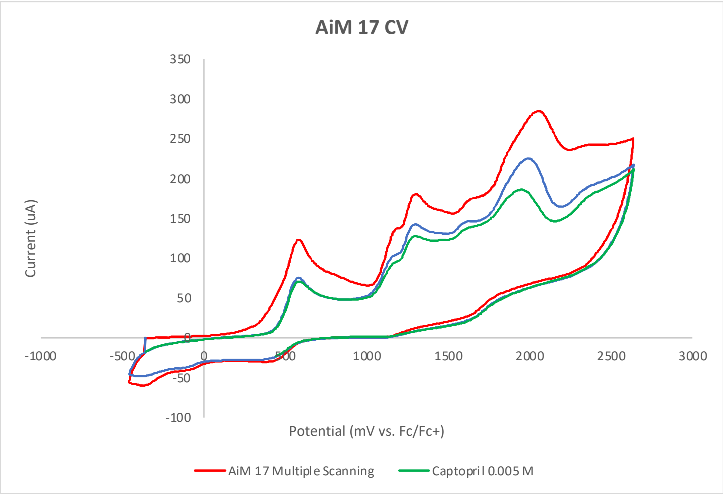

(2-chloro-10-(5-thiomorpholinopentyl)-10H-phenothiazine)

Cyclic Voltammetry Studies of (2-chloro-10-(5-thiomorpholinopentyl)-10H-phenothiazine)

$C_{21}H_{25}ClN_2S_2$, M_w= 405.02

CV staircase setting:
Start potential: 0 Vreff
Upper Vertex Potential: 2.00 Vreff Lower Vertex Potential: -0.10Vreff
Stop Potential: 0 Vreff
Number of Scans: 1
Scan Rate: 0.20 V/s
Step: 0.00244 V

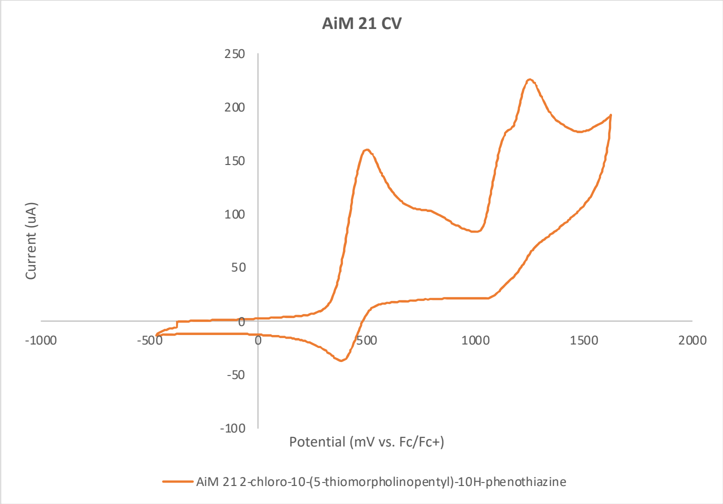

[1]Ep 0.492 V; ip 159 µA; Ep2 0.392 V; ip -37 µA; $E_{1/2}$ 0.44 V
[2]Ep 0.799 V; ip 103 µA
[3]Ep 1.160 V; ip 179 µA; Ep2 1.079 V; ip 23 µA; $E_{1/2}$ 1.12 V
[4]Ep 1.268 V; ip 224 µA; Ep2 1.184 V; ip 43 µA; $E_{1/2}$ 1.23 V

Potential Range of (2-chloro-10-(5-thiomorpholinopentyl)-10H-phenothiazine)
CV staircase setting:
Start potential: 0 Vreff
Upper Vertex Potential: 3.00 Vreff Lower Vertex Potential: -0.1Vreff
Stop Potential: 0 Vreff
Number of Scans: 1
Scan Rate: 0.20 V/s
Step: 0.00244 V

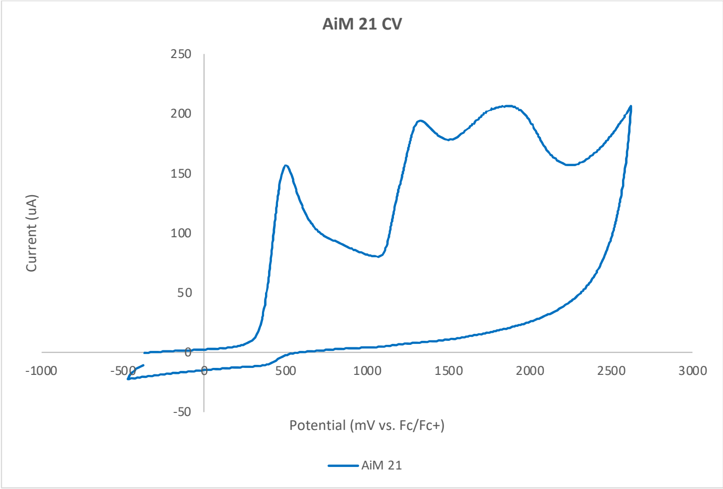

(2-chloro-10-(5-thiomorpholinopentyl)-10H-phenothiazine) CV Multiple Scanning

CV staircase setting:
Start potential: 0 Vreff
Upper Vertex Potential: 2.00
Vreff Lower Vertex Potential: -
0.10Vreff
Stop Potential: 0 Vreff
Number of Scans: 3
Scan Rate: 0.20 V/s
Step: 0.00244 V

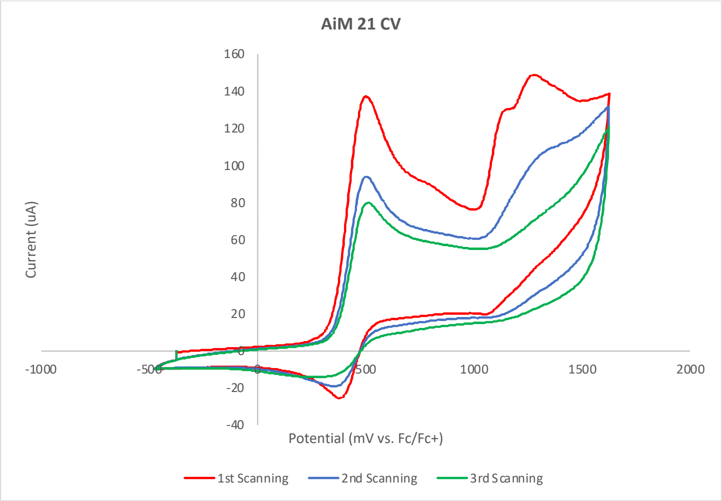

(4-(5-(2-chloro-10H-phenothiazin-10-yl)pentyl)morpholine)

Cyclic Voltammetry Studies of (4-(5-(2-chloro-10H-phenothiazin-10-yl)pentyl)morpholine)

Chemical Formula:
$C_{21}H_{25}ClN_2OS$
Molecular Weight: 388.95

(1.5 mg (4-(5-(2-chloro-10H-phenothiazin-10-yl)pentyl)morpholine)and 0.1 M tetrabutylammonium hexafluorophosphate in 10 mL MeCN)

CV staircase setting:
Start potential: 0 Vreff
Upper Vertex Potential: 2.00 Vreff
Lower Vertex Potential: -0.10Vreff
Stop Potential: 0 Vreff
Number of Scans: 1
Scan Rate: 0.20 V/s
Step: 0.00244 V

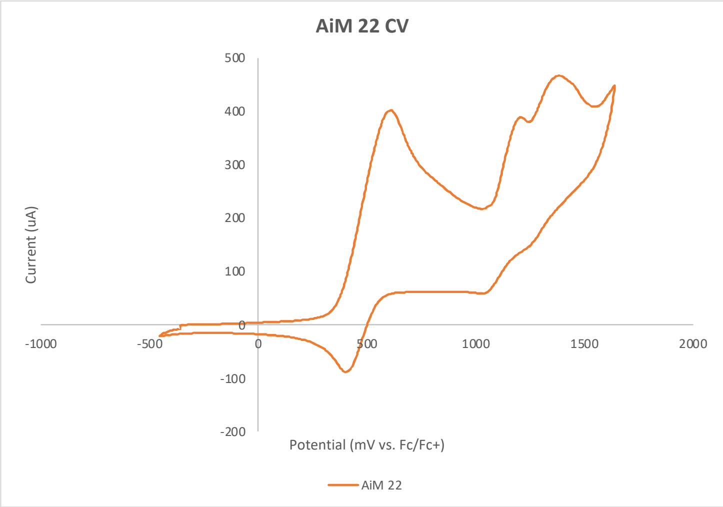

[1]Ep 0.598 V; ip 401 μA; Ep_2 0.408 V; ip -88 μA; $E_{1/2}$ 0.50 V
[2]Ep 1.200 V; ip 388 μA; Ep_2 1.049 V; ip 60μA; $E_{1/2}$ 1.13 V
[3]Ep 1.371 V; ip 466 μA; Ep_2 1.256 V; ip 151 μA; $E_{1/2}$ 1.31 V

(4-(5-(2-chloro-10H-phenothiazin-10-yl)pentyl)morpholine)CV Multiple Scanning
CV staircase setting:
Start potential: 0 Vreff
Upper Vertex Potential: 2.00 Vreff
Lower Vertex Potential: -0.10Vreff
Stop Potential: 0 Vreff
Number of Scans: 2
Scan Rate: 0.20 V/s
Step: 0.00244 V

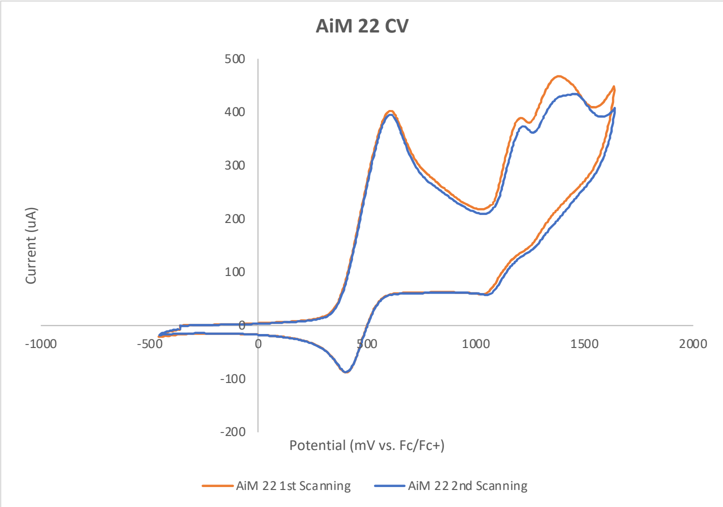

Potential Range of (4-(5-(2-chloro-10H-phenothiazin-10-yl)pentyl)morpholine)

CV staircase setting:
Start potential: 0 Vreff
Upper Vertex Potential: 3.00 Vreff
Lower Vertex Potential: -0.10Vreff
Stop Potential: 0 Vreff
Number of Scans: 1
Scan Rate: 0.20 V/s
Step: 0.00244 V

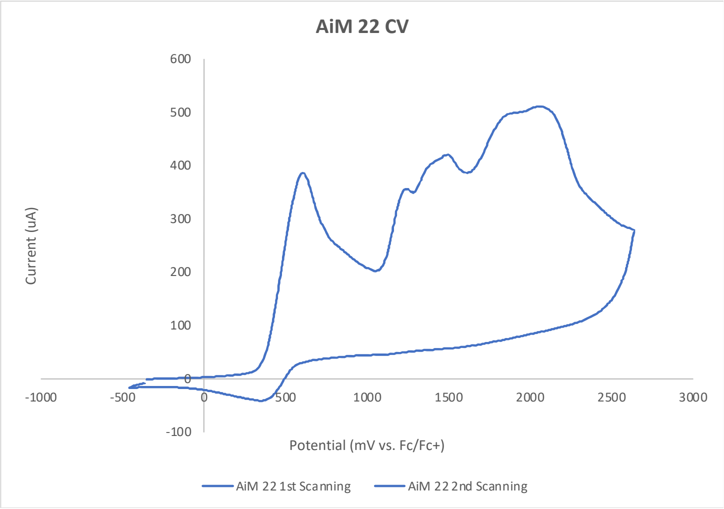

(4-(5-(2-chloro-10H-phenothiazin-10-yl)pentyl)morpholine) CV Multiple Scanning
CV staircase setting:
Start potential: 0 Vreff
Upper Vertex Potential: 3.00 Vreff
Lower Vertex Potential: -0.10Vreff
Stop Potential: 0 Vreff
Number of Scans: 4
Scan Rate: 0.20 V/s
Step: 0.00244 V

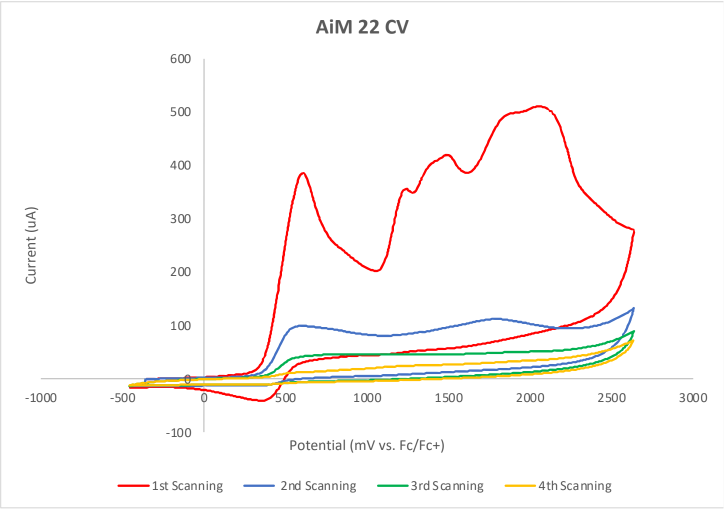

(2-(Methylthio)-10-(5-(piperazin-1-yl)pentyl)-10H-phenothiazine)

Cyclic Voltammetry Studies of (2-(Methylthio)-10-(5-(piperazin-1-yl)pentyl)-10H-phenothiazine)

Chemical Formula: $C_{22}H_{29}N_3S_2$
Molecular Weight: 399.62

(1.2 mg (2-(Methylthio)-10-(5-(piperazin-1-yl)pentyl)-10H-phenothiazine) and 0.1 M tetrabutylammonium hexafluorophosphate in 10 mL MeCN)

CV staircase setting:
Start potential: 0 Vreff
Upper Vertex Potential: 2.00 Vreff
Lower Vertex Potential: -0.10Vreff
Stop Potential: 0 Vreff
Number of Scans: 1
Scan Rate: 0.20 V/s
Step: 0.00244 V

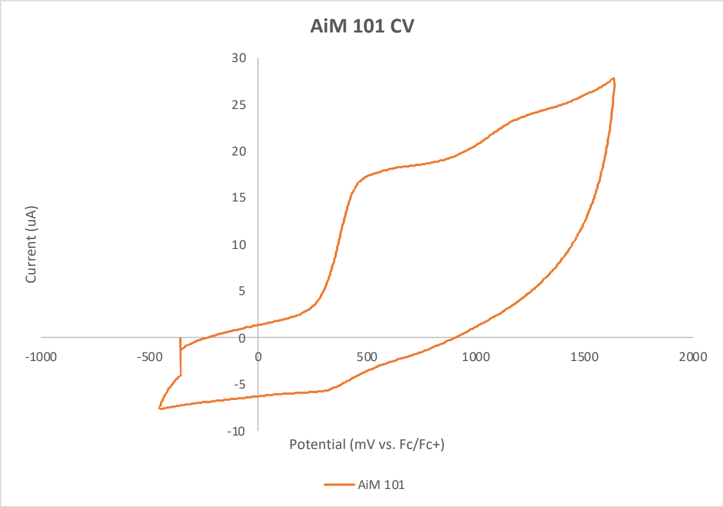

[1]Ep 0.484 V; ip 17 μA; Ep_2 0.335 V; ip -6 μA; $E_{1/2}$ 0.41 V
[2]Ep 1.186 V; ip 23 μA

(2-(methylthio)-10-(5-(piperazin-1-yl)pentyl)-10H-phenothiazine)CV Multiple Scanning
(1.2 mg AiM 101 and 0.1 M tetrabutylammonium hexafluorophosphate in 10 mL MeCN)

CV staircase setting:
Start potential: 0 Vreff
Upper Vertex Potential: 2.00 Vreff
Lower Vertex Potential: -0.1Vreff
Stop Potential: 0 Vreff
Number of Scans: 4
Scan Rate: 0.20 V/s
Step: 0.00244 V

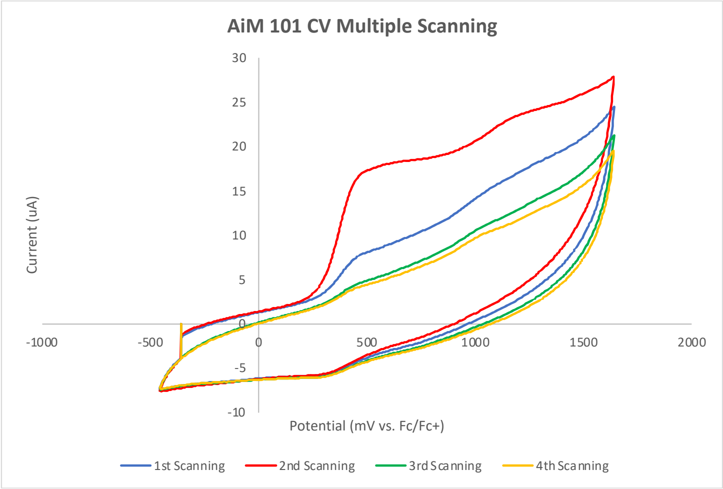

(10-(5-(4-methylpiperazin-1-yl)pentyl)-2-(methylthio)-10H-phenothiazine)

Cyclic Voltammetry Studies of (10-(5-(4-methylpiperazin-1-yl)pentyl)-2-(methylthio)-10H-phenothiazine)

Chemical Formula: $C_{23}H_{31}N_3S_2$
Molecular Weight: 413.64

(1.3 mg (10-(5-(4-methylpiperazin-1-yl)pentyl)-2-(methylthio)-10H-phenothiazine)and 0.1 M tetrabutylammonium hexafluorophosphate in 10 mL MeCN)

CV staircase setting:
Start potential: 0 Vreff
Upper Vertex Potential: 2.00 Vreff
Lower Vertex Potential: -0.10Vreff
Stop Potential: 0 Vreff
Number of Scans: 1
Scan Rate: 0.20 V/s
Step: 0.00244 V

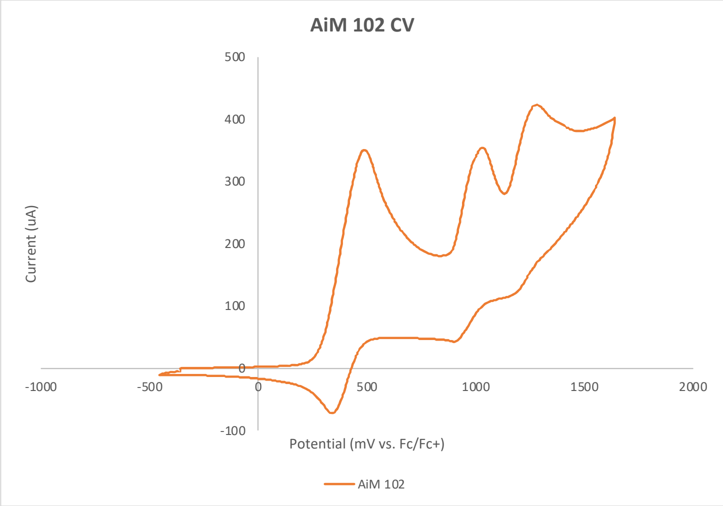

[1]Ep 0.478 V; ip 349 µA; Ep_2 0.342 V; ip -72 µA; $E_{1/2}$ 0.41 V
[2]Ep 1.022 V; ip 352 µA; Ep_2 0.910 V; ip 43 µA; $E_{1/2}$ 0.97 V
[3]Ep 1.269 V; ip 422 µA; Ep_2 1.185 V; ip 121 µA; $E_{1/2}$ 1.23 V

(10-(5-(4-methylpiperazin-1-yl)pentyl)-2-(methylthio)-10H-phenothiazine)CV Multiple Scanning
(1.3 mg AiM 102 and 0.1 M tetrabutylammonium hexafluorophosphate in 10 mL MeCN)

CV staircase setting:
Start potential: 0 Vreff
Upper Vertex Potential: 2.00 Vreff
Lower Vertex Potential: -0.10Vreff
Stop Potential: 0 Vreff
Number of Scans: 2
Scan Rate: 0.20 V/s
Step: 0.00244 V

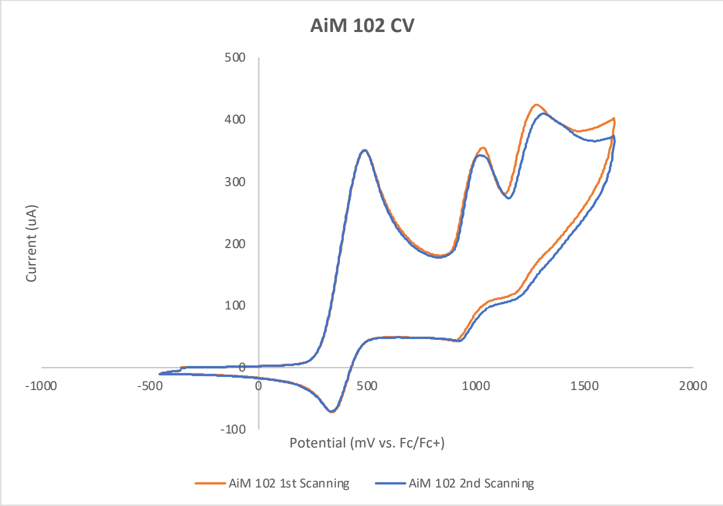

Potential Range of (10-(5-(4-methylpiperazin-1-yl)pentyl)-2-(methylthio)-10H-phenothiazine)

(10-(5-(4-methylpiperazin-1-yl)pentyl)-2-(methylthio)-10H-phenothiazine) up to 3 V/s
CV staircase setting:
Start potential: 0 Vreff
Upper Vertex Potential: 3.00 Vreff
Lower Vertex Potential: -0.10Vreff
Stop Potential: 0 Vreff
Number of Scans: 1
Scan Rate: 0.20 V/s
Step: 0.00244 V

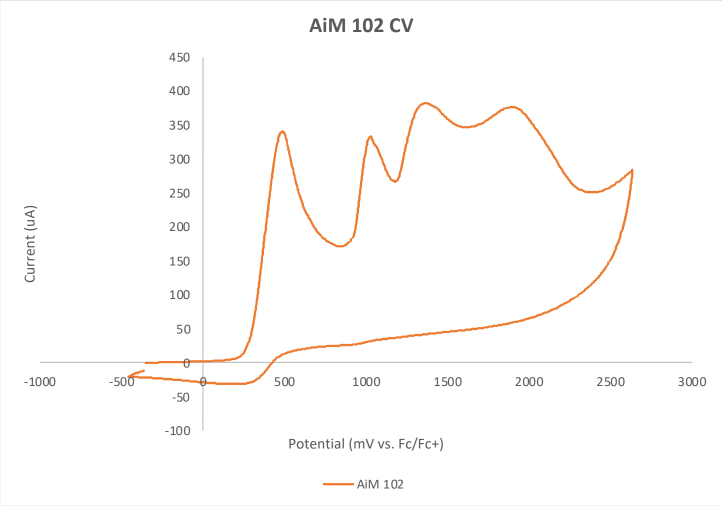

(10-(5-(4-methylpiperazin-1-yl)pentyl)-2-(methylthio)-10H-phenothiazine) after scanning up to 3 V/s
CV staircase setting:
Start potential: 0 Vreff
Upper Vertex Potential: 2.00 Vreff
Lower Vertex Potential: -0.10Vreff
Stop Potential: 0 Vreff
Number of Scans: 1
Scan Rate: 0.20 V/s
Step: 0.00244 V

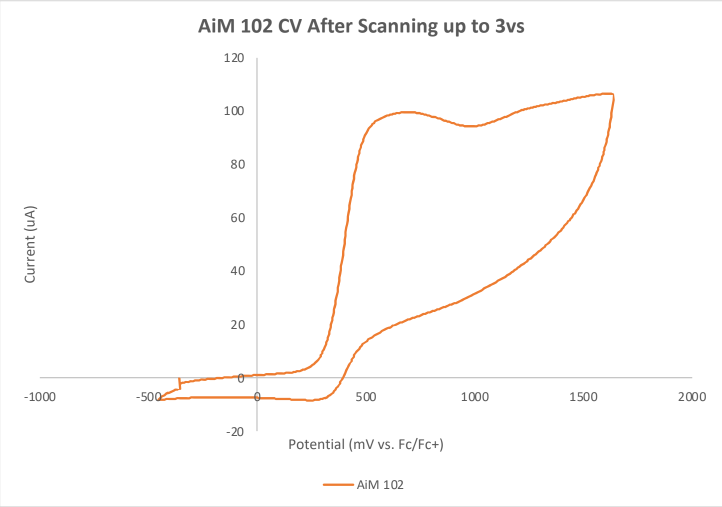

(2-(4-(5-(2-(methylthio)-10H-phenothiazin-10-yl)pentyl)piperazin-1-yl)ethan-1-ol)

Cyclic Voltammetry Studies of (2-(4-(5-(2-(methylthio)-10H-phenothiazin-10-yl)pentyl)piperazin-1-yl)ethan-1-ol)

Chemical Formula: $C_{24}H_{33}N_3OS_2$
Molecular Weight: 443.67

(2.3 mg (2-(4-(5-(2-(methylthio)-10H-phenothiazin-10-yl)pentyl)piperazin-1-yl)ethan-1-ol) and 0.1 M tetrabutylammonium hexafluorophosphate in 10 mL MeCN)

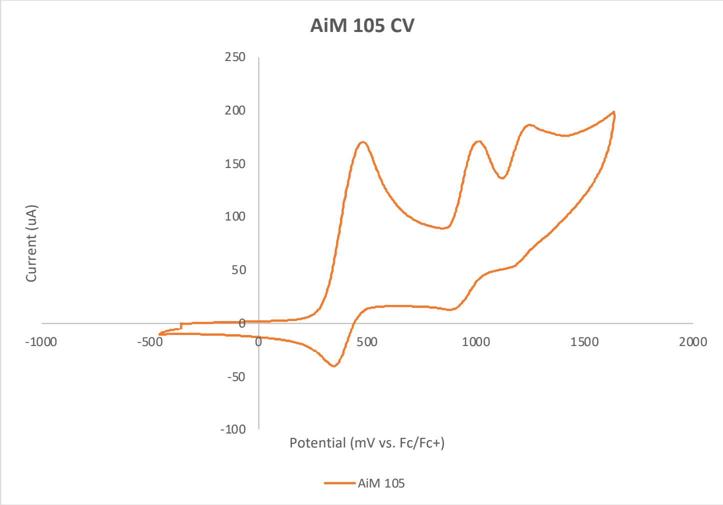

[1]Ep 0.469 V; ip 169 μA; Ep_2 0.352 V; ip-40 μA; $E_{1/2}$ 0.41 V

[2]Ep 1.003 V; ip 170 μA; Ep_2 0.895 V; ip13 μA; $E_{1/2}$ 0.95 V

[3]Ep 1.261 V; ip 186 μA; Ep_2 1.186 V; ip55μA; $E_{1/2}$ 1.22 V

CV staircase setting:
Start potential: 0 Vreff
Upper Vertex Potential: 2.0 Vreff
Lower Vertex Potential: -0.1
Stop Potential: 0 Vreff
Number of Scans: 2
Scan Rate: 0.2 V/s
Step: 0.00244 V

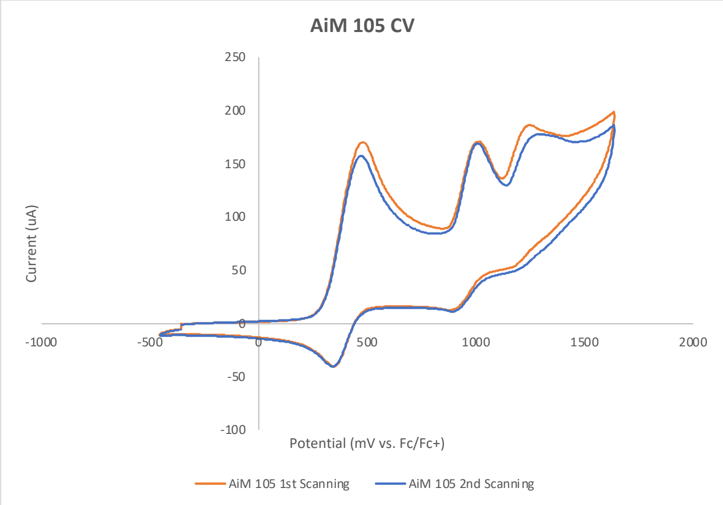

(2-(4-(5-(2-(methylthio)-10H-phenothiazin-10-yl)pentyl)piperazin-1-yl)ethan-1-ol)CV up to 3 vs
CV staircase setting:
Start potential: 0 Vreff
Upper Vertex Potential: 3.0 Vreff
Lower Vertex Potential: -0.1
Stop Potential: 0 Vreff
Number of Scans: 1
Scan Rate: 0.2 V/s
Step: 0.00244 V

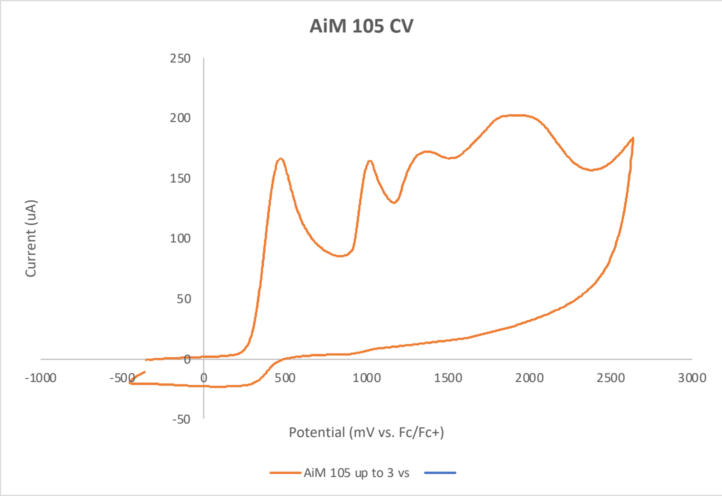

(2-(4-(5-(2-(methylthio)-10H-phenothiazin-10-yl)pentyl)piperazin-1-yl)ethan-1-ol)CV after scanning up to 3 vs
CV staircase setting:
Start potential: 0 Vreff
Upper Vertex Potential: 2.0 Vreff
Lower Vertex Potential: -0.1
Stop Potential: 0 Vreff
Number of Scans: 4
Scan Rate: 0.2 V/s
Step: 0.00244 V

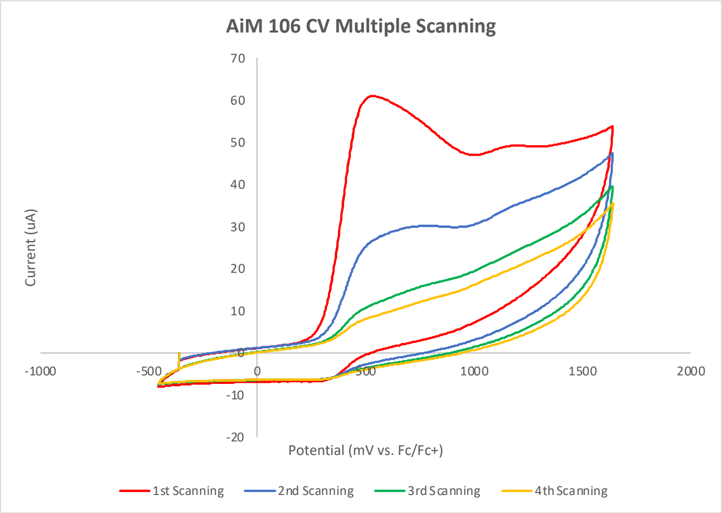

(N,N-diethyl-5-(2-(methylthio)-10H-phenothiazin-10-yl)pentan-1-amine)

Cyclic Voltammetry Studies of (N,N-diethyl-5-(2-(methylthio)-10H-phenothiazin-10-yl)pentan-1-amine)

Chemical Formula: $C_{22}H_{30}N_2S_2$,
Molecular Weight: 386.62

(3 mg (N,N-diethyl-5-(2-(methylthio)-10H-phenothiazin-10-yl)pentan-1-amine)and 0.1 M tetrabutylammonium hexafluorophosphate in 10 mL MeCN)

CV staircase setting:
Start potential: 0 Vreff
Upper Vertex Potential: 2.00 Vreff
Lower Vertex Potential: -0.10Vreff
Stop Potential: 0 Vreff
Number of Scans: 2
Scan Rate: 0.20 V/s
Step: 0.00244 V

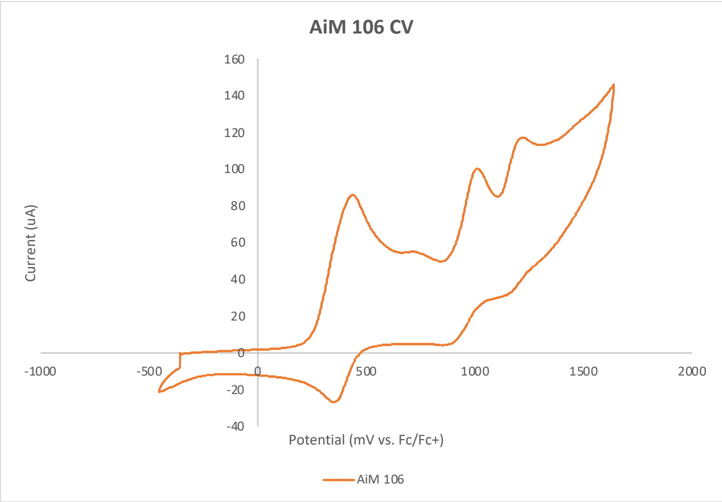

[1]Ep 0.430 V; ip 86 µA; Ep2 0.355 V; ip-27 µA; $E_{1/2}$ 0.39 V
[1]Ep 0.742 V; ip 54 µA
[3]Ep 1.006 V; ip 100 µA; Ep2 0.888 V; ip 4.80 µA; $E_{1/2}$ 0.95 V
[4]Ep 1.232 V; ip 117 µA; Ep2 1.156 V; ip 32 µA; $E_{1/2}$ 1.19 V

(N,N-diethyl-5-(2-(methylthio)-10H-phenothiazin-10-yl)pentan-1-amine)CV Multiple Scanning
CV staircase setting:
Start potential: 0 Vreff
Upper Vertex Potential: 2.00 Vreff
Lower Vertex Potential: -0.10Vreff
Stop Potential: 0 Vreff
Number of Scans: 2
Scan Rate: 0.20 V/s
Step: 0.00244 V

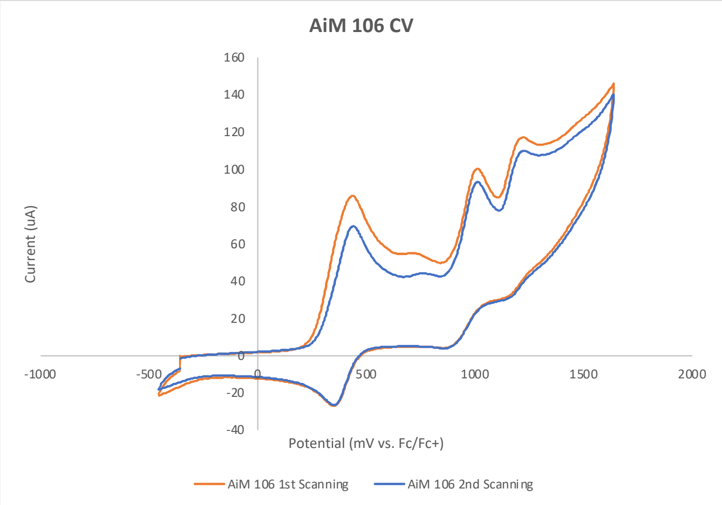

Potential Range of (N,N-diethyl-5-(2-(methylthio)-10H-

phenothiazin-10-yl)pentan-1-amine)
CV staircase setting:
Start potential: 0 Vreff
Upper Vertex Potential: 3.00 Vreff
Lower Vertex Potential: -0.10Vreff
Stop Potential: 0 Vreff
Number of Scans: 2
Scan Rate: 0.20 V/s
Step: 0.00244 V

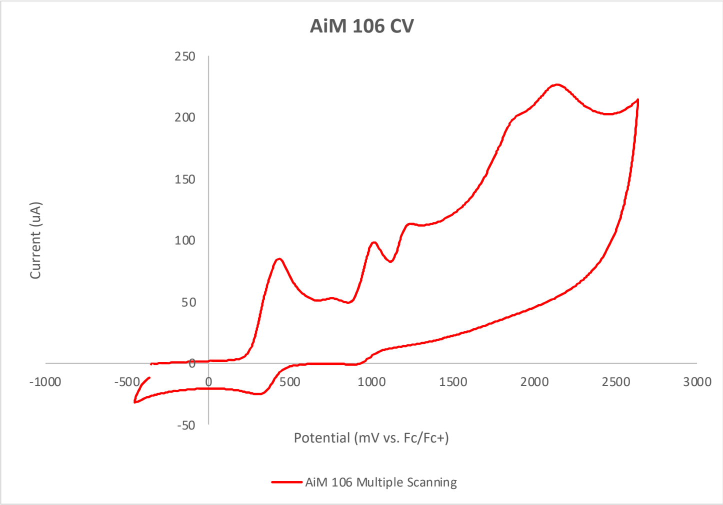

(N,N-diethyl-5-(2-(methylthio)-10H-phenothiazin-10-yl)pentan-1-amine) CV Multiple Scanning

CV staircase setting:
Start potential: 0 Vreff
Upper Vertex Potential: 3.0 0Vreff
Lower Vertex Potential: -0.1Vreff
Stop Potential: 0 Vreff
Number of Scans: 4
Scan Rate: 0.20 V/s
Step: 0.00244 V

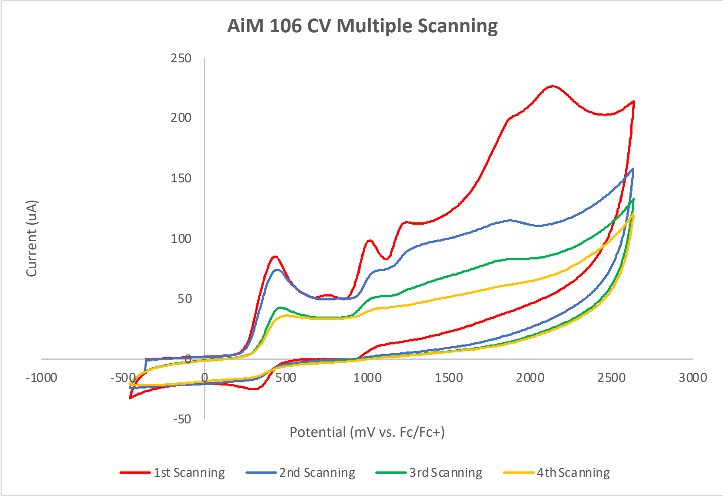

Molecular Docking Results

Molecular docking was performed with Flare^{TM} V 8.0.0 from Cresset. The protein structure was obtained from a protein data bank (PDB) and prepared according to the Flare V8.0 manual.

CYP1A2 (2HI4), CYP2B6 (4RQL), CYP3A4 (1TQN)',CYP2C9 (4NZ2), andCYP2D6 (5TFT)

The protein was prepared using the following parameters: calculation method—normal; cap chains—intelligent capping; remove waters outside active side—yes; active site size—6;00 Å; copy protein and auto-extract ligand (reference)—yes. The ligand was prepared using the following parameters: pop to 3D and minimize—yes. The docking calculation method was performed using the following parameters: method—very accurate but slow; number of runs—3; max poses to output each ligand—100. Lead Finder version 2212 build 1, 10 December 2022, was used.

Information about the Scoring function

1. Ranking scoring function, or Rank Score: this function is used for ranking ligand poses obtained during a docking run. The purpose of this function is to reproduce experimentally observed ligand poses as well as possible

2. dG-scoring function, or dG score: this scoring function has been designed to perform an accurate estimation of the free energy of protein–ligand binding for a given protein–ligand complex. The scaling coefficients for this function have been derived by fitting calculated binding energies to the experimental values for a set of 100 protein–ligand complexes with known 3D structures and experimentally measured binding constants

3. Virtual screening (VS) scoring function, or VSscore: this function has been designed to produce maximum efficiency in virtual screening experiments i.e. to assign higher scores to active ligands (true binders) and lower scores to inactive ligands.

a)

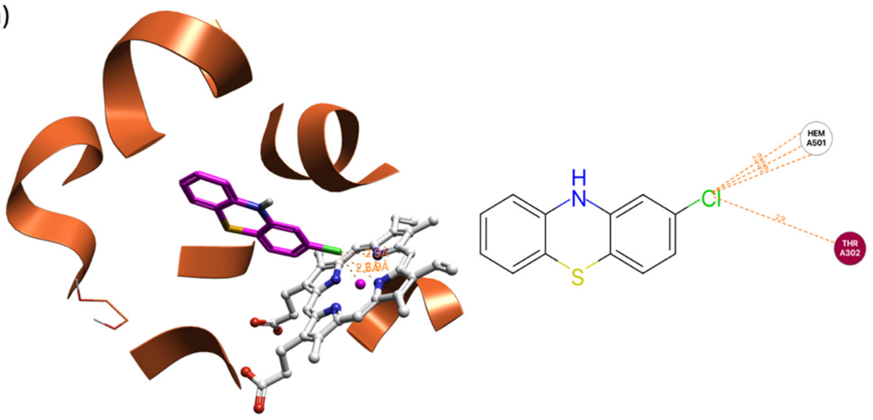

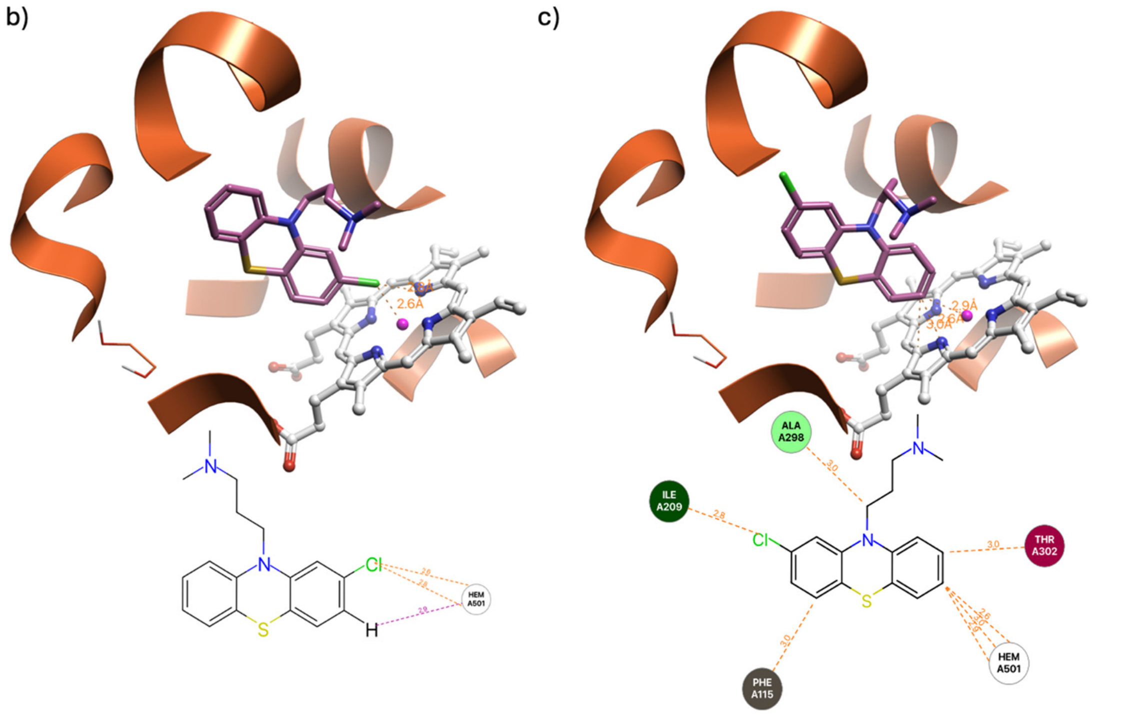

Figure S1. PTZs docked to CYP2B6 (Fe interaction)

Table S1. Binding energy score of molecular docking PTZs to CYP2B6 (Fe interaction)

Pos e	dG, kcal/mol	VS score	LE, (kcal/mol)/atom	Rank score	Interaction
a	-7.57807	-7.86413	-0.50520	-6.94236	Steric clashes
b	-9.29444	-9.49358	-0.44259	-7.02473	Steric clashes
c	-7.99483	-8.61872	-0.38071	-3.54414	Steric clashes

A steric interaction between the chlorine of 2CPTZ and iron exists on the CYP2B6 (Figure 9).

d)

e)

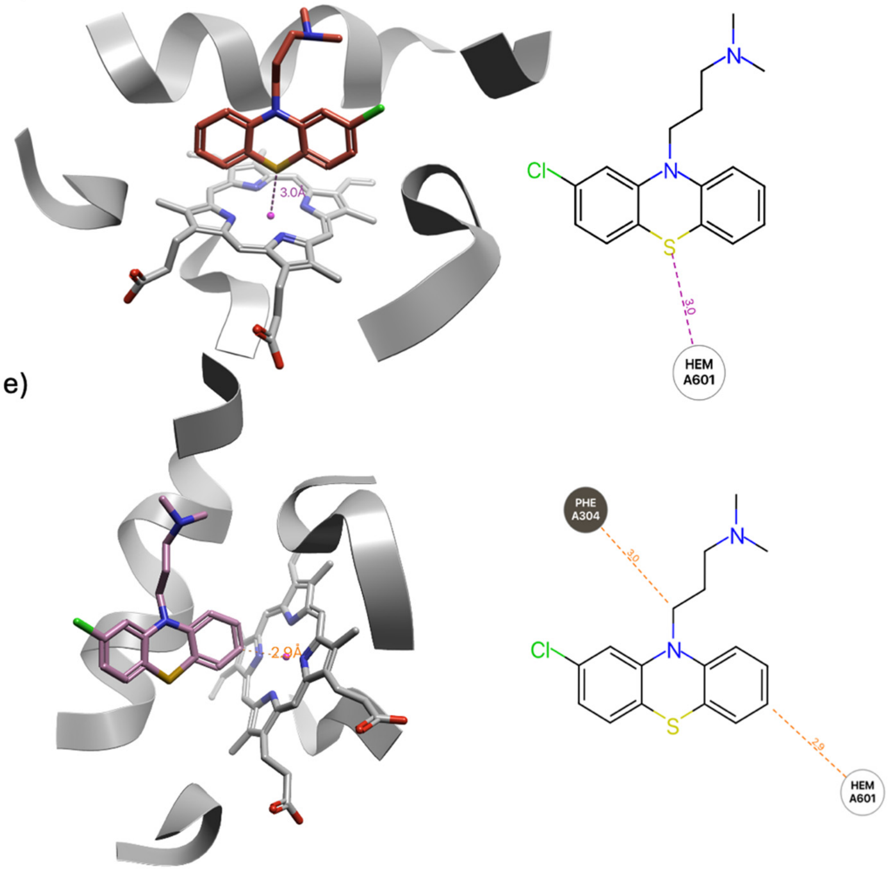

Figure S2. PTZs docked to CYP3A4 (Fe interaction)

Table S2. Binding energy score of molecular docking PTZs to CYP3A64 (Fe interaction)

Pose	dG, kcal/mol	VS score	LE, (kcal/mol)/atom	Rank score	Interaction
d	-7.45125	-7.96179	-0.35482	-6.31011	Salt bridge metal, sulfur ion pairs
e	-7.52928	-7.82384	-0.35854	-6.12549	Steric clashes

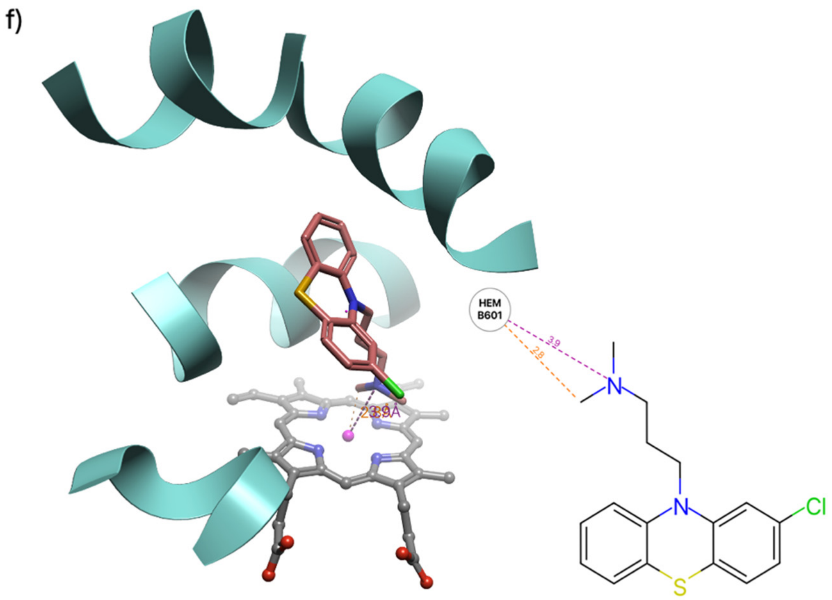

Figure S3. PTZs docked to CYP2D6 (Fe interaction)

Table S3. Binding energy score of molecular docking PTZs to CYP2D6 (Fe interaction)

Pose	dG, kcal/mol	VS score	LE, (kcal/mol)/atom	Rank score	Interaction
f	-8.45742	-8.83563	-0.40273	-7.69322	Steric clashes

MOLECULAR DOCKING DATA

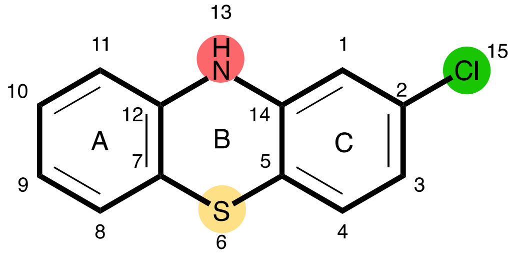

2-Chlorophenothiazine (**2CPTZ**)

Docked to **CYP1A2 (2HI4)**

Top pose dG, kcal/mol : -8.85389

Top pose VS score : -9.20253

Top pose LE, (kcal/mol)/atom : -0.59026

Pose with best free energy of binding has dG, kcal/mol : -8.85389

Pose with best free energy of binding has VS score : -9.20253

Hem A900 Interaction

Pose	dG, kcal/mol	VS score	LE, (kcal/mol)/atom	Rank score	Position/Distance
0	-8.85389	-9.20253	-0.59026	-10.50218	na
1	-8.69538	-8.99924	-0.57969	-9.89036	na
2	-8.44439	-8.80109	-0.56296	-9.88566	na
3	-8.49353	-8.84647	-0.56624	-9.66308	H10(4.4) H11(4.4)
4	-8.40089	-8.76470	-0.56006	-9.58857	na
5	-8.56194	-8.90732	-0.57080	-9.38900	H9(3.0)
6	-8.38174	-8.72481	-0.55878	-9.32323	na
7	-8.31287	-8.65923	-0.55419	-9.17449	na
8	-7.69614	-8.25981	-0.51308	-8.84924	na

Docked to **CYP2B6 (4RQL)**

Top pose dG, kcal/mol : -7.86125

Top pose VS score : -8.10683

Top pose LE, (kcal/mol)/atom : -0.52408

Pose with best free energy of binding has dG, kcal/mol : -7.86125

Pose with best free energy of binding has VS score : -8.10683

Hem A501 Interaction

Pose	dG, kcal/mol	VS score	LE, (kcal/mol)/atom	Rank score	Position/Distance
0	-7.86125	-8.10683	-0.52408	-7.19710	Cl15(3.0) C3(2.9) H3(2.3)
1	-7.70201	-7.98164	-0.51347	-7.03211	C9(3.8)C10(3.8)
2	-7.57807	-7.86413	-0.50520	-6.94236	Cl15(Fe-2.5,2.9) H3(2.5)C3(3.5)
3	-7.38598	-7.72196	-0.49240	-6.93176	H10(2.5)C10(3.6)A(4.8)
4	-7.68892	-8.03891	-0.51259	-6.88785	C3(2.8)C4(3.7)
5	-7.36812	-7.72671	-0.49121	-6.80056	C10(2.9) H10(2.5)
6	-7.39478	-7.72657	-0.49299	-6.67721	Cl15(2.9) H3(3.2)
7	-7.31381	-7.64080	-0.48759	-6.55188	H9(3.3) H10(2.9)
8	-7.71497	-8.04532	-0.51433	-6.54799	Cl15 (2.9) H1(3.6, 4.2)
9	-7.07891	-7.46527	-0.47193	-6.48255	C9(3.5)H9(2.5) C10(3.6)H10(2.6)
10	-7.44096	-7.76033	-0.49606	-6.39384	Cl15 (2.9) H1(4.5)
11	-7.09993	-7.48591	-0.47333	-6.32327	Cl15 (2.9,3.0) C3(3.4)H3(3.1)
12	-7.49119	-7.95875	-0.49941	-5.97660	C(4.6), C3(2.7,2.7)C4(3.6)
13	-7.20353	-7.67052	-0.48024	-5.09125	na
14	-7.49080	-7.92255	-0.49939	-5.04122	na
15	-5.73681	-6.44037	-0.38245	-4.93819	na
16	-6.99673	-7.39442	-0.46645	-4.68305	C9(3.2)H9(2.4) H10(2.3) C10(2.9)
17	-6.95533	-7.40922	-0.46369	-4.66040	na
18	-6.92981	-7.34637	-0.46199	-4.01301	Cl15 (2.6) H3(3.5)
19	-7.17392	-7.65001	-0.47826	-3.97563	na
20	-5.26308	-6.41192	-0.35087	-0.25260	H10(3.0) H11(3.8)

Top pose dG, kcal/mol : -7.73814

Top pose VS score : -7.98447

Top pose LE, (kcal/mol)/atom : -0.51588

Pose with best free energy of binding has dG, kcal/mol : -7.85096

Pose with best free energy of binding has VS score : -8.09419

Hem B501 Interaction

Pose	dG, kcal/mol	VS score	LE, (kcal/mol)/atom	Rank score	Position/Distance
0	-7.73814	-7.98447	-0.51588	-7.17504	Cl15(Fe-2.4, 2.9) H3(2.7)C3(3.3)
1	-7.77745	-8.04440	-0.51850	-7.13703	C9(3.8)C10(3.8)
2	-7.85096	-8.09419	-0.52340	-7.12422	Cl15(2.8) H3(2.4)C3(3.2)
3	-7.71894	-7.99055	-0.51460	-7.11545	Cl15(Fe-2.6)
4	-7.52322	-7.83685	-0.50155	-6.98627	Cl15(2.9) H3(3.1)
5	-7.38520	-7.74014	-0.49235	-6.94680	H9(3.3) H10(2.8)C10(3.7)
6	-7.42020	-7.76829	-0.49468	-6.77310	A(4.6) H10(2.2)C10(3.4)
7	-7.13280	-7.49918	-0.47552	-6.56333	H9(2.4)C9(3.6)H10(2.7)C10(3.6)
8	-6.90951	-7.35096	-0.46063	-6.26643	C9(3.0)C10(3.4)
9	-7.43176	-7.90264	-0.49545	-5.78385	C(4.6) C3(2.6,2.8,3.0)C4(3.7)
10	-7.23893	-7.61217	-0.48260	-5.36945	H9(2.3)C9(3.5)H10(2.4)C10(3.0)
11	-6.84128	-7.36453	-0.45609	-4.78046	na
12	-6.25626	-6.85498	-0.41708	-4.68111	na
13	-6.26554	-6.93014	-0.41770	-2.87603	na

Docked to **CYP3A4 (1TQN)**

Top pose dG, kcal/mol : -5.16670

Top pose VS score : -6.45144

Top pose LE, (kcal/mol)/atom : -0.34445

Pose with best free energy of binding has dG, kcal/mol : -6.75485

Pose with best free energy of binding has VS score : -7.21736

Hem A601 Interaction

Pose	dG, kcal/mol	VS score	LE, (kcal/mol)/atom	Rank score	Position/Distance
0	-5.16670	-6.45144	-0.34445	-7.80002	na
1	-6.16255	-6.85360	-0.41084	-7.66196	Cl(3.0)
2	-5.65186	-6.51387	-0.37679	-7.46291	na
3	-5.96428	-6.63136	-0.39762	-7.18594	na
4	-6.15929	-6.59505	-0.41062	-7.09432	na
5	-5.90396	-6.47258	-0.39360	-7.08922	na
6	-5.92186	-6.43364	-0.39479	-7.03845	na
7	-5.86554	-6.42195	-0.39104	-6.94624	H1(4.3)
8	-6.12668	-6.58224	-0.40845	-6.93684	na
9	-6.17583	-6.66525	-0.41172	-6.90094	A(4.9)H10(3.0)C10(3.4
10	-6.09108	-6.57406	-0.40607	-6.86618	na
11	-6.21577	-6.58301	-0.41438	-6.79172	na
12	-6.02163	-6.33344	-0.40144	-6.72702	na
13	-5.77243	-6.31938	-0.38483	-6.67230	na
14	-6.19182	-6.56378	-0.41279	-6.58941	na
15	-6.51164	-7.02209	-0.43411	-6.55952	S(3.4)C(3.6)C1,C5,C7-11
16	-5.98453	-6.50598	-0.39897	-6.52087	H3(2.6)
17	-5.68589	-6.21013	-0.37906	-6.50080	na
18	-5.58902	-6.01512	-0.37260	-6.50057	na
19	-6.02443	-6.40696	-0.40163	-6.48793	na
20	-5.72725	-6.28612	-0.38182	-6.48546	na
21	-5.59527	-6.10442	-0.37302	-6.47334	na
22	-5.49186	-6.07830	-0.36612	-6.42743	na
23	-5.32932	-5.88577	-0.35529	-6.42435	na
24	-5.57830	-6.43231	-0.37189	-6.42007	Cl(2.9)H1,C1,H13,C10,C11, H11
25	-6.75485	-7.21736	-0.45032	-6.40825	A(3.4)C(3.8)C1,C3,C4,C5,C7,C9-11
26	-5.99843	-6.46722	-0.39990	-6.40242	na
27	-5.77283	-6.38153	-0.38486	-6.40134	H8(3.8)H9(2.1)C9(3.4)C10(3.8)
28	-6.10558	-6.56056	-0.40704	-6.38583	na
29	-5.92279	-6.41363	-0.39485	-6.36308	na
30	-5.98883	-6.40265	-0.39926	-6.36253	na
31	-6.34055	-6.87045	-0.42270	-6.35199	S(3.8)C(3.9,4.1)Cl(2.9,3.0)C1,C4
32	-6.34445	-6.72020	-0.42296	-6.34952	H3(2.7)
33	-5.70200	-6.11394	-0.38013	-6.28722	na
34	-5.60226	-6.05605	-0.37348	-6.25843	na

Docked to **CYP2C9 (4NZ2)**

Top pose dG, kcal/mol : -8.04259

Top pose VS score : -8.15166

Top pose LE, (kcal/mol)/atom : -0.53617

Pose with best free energy of binding has dG, kcal/mol : -8.04955

Pose with best free energy of binding has VS score : -8.10252

Hem B501 Interaction

Pose	dG, kcal/mol	VS score	LE, (kcal/mol)/atom	Rank score	Position/Distance
0	-8.04259	-8.15166	-0.53617	-7.19152	na
1	-5.90004	-6.75310	-0.39334	-7.16239	na
2	-7.56760	-7.77446	-0.50451	-7.09317	na
3	-7.98897	-8.09721	-0.53260	-7.06569	na
4	-7.34151	-7.61599	-0.48943	-6.98529	na
5	-6.85639	-7.27430	-0.45709	-6.86447	na
6	-7.24506	-7.62933	-0.48300	-6.85705	na
7	-6.43928	-6.88927	-0.42929	-6.83311	na
8	-6.54997	-6.93367	-0.43666	-6.82953	na
9	-7.09301	-7.56526	-0.47287	-6.81144	na
10	-6.37898	-6.84312	-0.42527	-6.79391	na
11	-8.04955	-8.10252	-0.53664	-6.73626	na
12	-6.00414	-6.78513	-0.40028	-6.67343	na
13	-6.95281	-7.27480	-0.46352	-6.62441	na
14	-6.61149	-7.07404	-0.44077	-6.59464	na
15	-6.29682	-6.80459	-0.41979	-6.52578	na
16	-6.98024	-7.30877	-0.46535	-6.52230	na
17	-5.87879	-6.55209	-0.39192	-6.45121	na
18	-7.22268	-7.57142	-0.48151	-6.43063	na
19	-6.41692	-6.89009	-0.42779	-6.42900	na
20	-6.34138	-6.86309	-0.42276	-6.41616	na
21	-6.79875	-7.18333	-0.45325	-6.30619	H1(4.4)
22	-6.80124	-7.11817	-0.45342	-6.28199	na
23	-6.36484	-6.79194	-0.42432	-6.17806	na
24	-6.68873	-7.09871	-0.44592	-6.14422	na
25	-6.89430	-7.15802	-0.45962	-5.93708	na

Top pose dG, kcal/mol : -7.48003

Top pose VS score : -7.69709

Top pose LE, (kcal/mol)/atom : -0.49867

Pose with best free energy of binding has dG, kcal/mol : -7.59301

Pose with best free energy of binding has VS score : -7.81775

Hem A501 Interaction

Pose	dG, kcal/mol	VS score	LE, (kcal/mol)/atom	Rank score	Position/Distance
0	-7.48003	-7.69709	-0.49867	-7.20855	na
1	-6.90723	-7.32985	-0.46048	-7.04461	na
2	-6.78084	-7.19544	-0.45206	-6.94184	na
3	-6.61574	-7.28559	-0.44105	-6.93568	na
4	-7.59301	-7.81775	-0.50620	-6.91615	na
5	-7.28508	-7.61274	-0.48567	-6.89384	na
6	-7.36777	-7.61362	-0.49118	-6.85796	na
7	-6.57745	-7.02299	-0.43850	-6.84421	na
8	-7.46357	-7.68776	-0.49757	-6.82264	na
9	-5.45814	-6.10597	-0.36388	-6.78055	na
10	-6.47100	-6.90303	-0.43140	-6.73670	na
11	-6.64703	-7.10297	-0.44314	-6.71243	na
12	-6.71948	-7.12245	-0.44797	-6.66653	na
13	-6.59614	-6.98080	-0.43974	-6.66341	na
14	-6.60577	-7.27773	-0.44038	-6.66063	na
15	-5.87140	-6.66646	-0.39143	-6.65612	na
16	-7.20464	-7.48092	-0.48031	-6.53629	na
17	-6.15313	-6.61986	-0.41021	-6.53166	na
18	-6.27792	-6.82871	-0.41853	-6.52575	na
19	-6.79157	-7.15999	-0.45277	-6.50238	na
20	-6.51089	-6.97253	-0.43406	-6.42716	H1(4.4)Cl(2.9)
21	-6.37370	-6.87379	-0.42491	-6.42468	na
22	-6.38175	-6.82028	-0.42545	-6.36113	na
23	-6.78461	-7.14433	-0.45231	-6.36051	na
24	-6.48544	-6.87600	-0.43236	-6.24993	na
25	-6.67851	-6.97181	-0.44523	-5.94921	na

Docked to **CYP2D6 (5TFT)**

Top pose dG, kcal/mol : -6.33625

Top pose VS score : -7.22069

Top pose LE, (kcal/mol)/atom : -0.42242

Pose with best free energy of binding has dG, kcal/mol : -7.58091

Pose with best free energy of binding has VS score : -7.95144

Hem A601 Interaction

Pose	dG, kcal/mol	VS score	LE, (kcal/mol)/atom	Rank score	Position/Distance
0	-6.33625	-7.22069	-0.42242	-8.00876	na
1	-7.03847	-7.57143	-0.46923	-7.81955	na
2	-7.48935	-7.87982	-0.49929	-7.80538	na
3	-7.13493	-7.57882	-0.47566	-7.70063	na
4	-6.98081	-7.42881	-0.46539	-7.67177	na
5	-6.53742	-7.25777	-0.43583	-7.55389	na
6	-7.58091	-7.95144	-0.50539	-7.40944	C9(3.8)C10(3.7)H10(3.1)
7	-6.66239	-7.24158	-0.44416	-7.34330	na
8	-6.77003	-7.21040	-0.45134	-7.32020	na
9	-6.73575	-7.06516	-0.44905	-7.27243	na
10	-6.60781	-7.01097	-0.44052	-7.25969	na
11	-6.86488	-7.13048	-0.45766	-7.20723	na
12	-6.31090	-6.86328	-0.42073	-7.15560	na
13	-6.76964	-7.16610	-0.45131	-7.02878	na
14	-6.55978	-6.96460	-0.43732	-6.88924	na

Top pose dG, kcal/mol : -7.09223

Top pose VS score : -7.77137

Top pose LE, (kcal/mol)/atom : -0.47282

Pose with best free energy of binding has dG, kcal/mol : -7.61582

Pose with best free energy of binding has VS score : -7.97618

Hem B601 Interaction

Pose	dG, kcal/mol	VS score	LE, (kcal/mol)/atom	Rank score	Position/Distance
0	-7.09223	-7.77137	-0.47282	-8.29504	na
1	-7.20038	-7.99465	-0.48003	-8.27015	na
2	-6.17488	-7.24851	-0.41166	-8.24529	na
3	-6.46655	-7.32437	-0.43110	-8.23399	na
4	-6.86215	-7.60844	-0.45748	-8.07874	na
5	-6.77586	-7.46872	-0.45172	-7.87637	na
6	-7.52857	-7.99011	-0.50190	-7.77685	C3(3.4)H3(2.5)
7	-7.10916	-7.72417	-0.47394	-7.76054	na
8	-6.86732	-7.46049	-0.45782	-7.71952	na
9	-7.61582	-7.97618	-0.50772	-7.71840	C9(3.4)C10(3.7)H10(2.9)
10	-7.33210	-7.87542	-0.48881	-7.67108	H10(4.0)
11	-6.79755	-7.27007	-0.45317	-7.50143	na
12	-6.88108	-7.50517	-0.45874	-7.49206	na
13	-6.67544	-7.13589	-0.44503	-7.43278	na
14	-7.22936	-7.55550	-0.48196	-7.34366	na
15	-6.80178	-7.26239	-0.45345	-7.16547	na
16	-7.05894	-7.41028	-0.47060	-7.14046	na
17	-6.76205	-7.18100	-0.45080	-7.11091	na

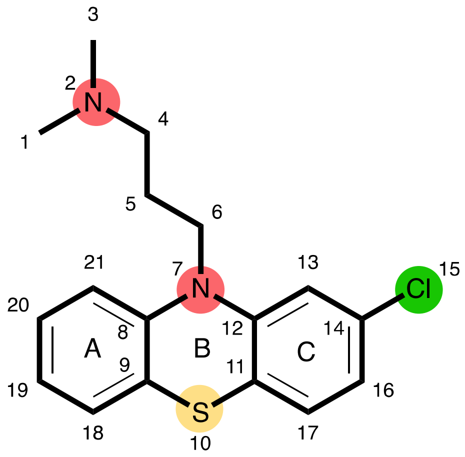

Chlorpromazine (**CPZ**)

Docked to **CYP1A2 (2HI4)**

Top pose dG, kcal/mol : -10.84496

Top pose VS score : -10.97010

Top pose LE, (kcal/mol)/atom : -0.51643

Pose with best free energy of binding has dG, kcal/mol : -11.16137

Pose with best free energy of binding has VS score : -11.19985

Hem A900 Interaction

Pose	dG, kcal/mol	VS score	LE, (kcal/mol)/atom	Rank score	Position/Distance
0	-10.84496	-10.97010	-0.51643	-10.31280	na
1	-10.96036	-11.06339	-0.52192	-10.21421	H19(3.5) H20(3.4)
2	-10.56234	-10.74557	-0.50297	-9.66390	H19(4.2)
3	-11.16137	-11.19985	-0.53149	-9.65341	na
4	-9.62332	-10.01255	-0.45825	-9.63800	na
5	-10.74615	-11.09876	-0.51172	-9.59275	na
6	-10.56555	-10.69410	-0.50312	-9.56703	H18(4.3) H19(4.4)
7	-10.50962	-10.68352	-0.50046	-9.54331	Cl15(2.9;3.0) H16(4.1)
8	-9.49531	-10.05397	-0.45216	-9.47196	H18(4.0)
9	-10.68904	-10.85091	-0.50900	-9.42783	na
10	-10.39775	-10.62740	-0.49513	-9.29987	na
11	-10.13724	-10.40786	-0.48273	-9.25223	H16(3.6) H17(3.8)
12	-10.14123	-10.43242	-0.48292	-9.01873	H19(3.8) H20(3.4)
13	-9.73731	-10.19848	-0.46368	-9.00854	H20(4.1)
14	-8.77945	-9.59338	-0.41807	-8.67896	H18(3.2) H19(3.1)
15	-10.01182	-10.40317	-0.47675	-8.53849	C18(3.8)H18(3.0) H19(3.9)
16	-10.64742	-10.81335	-0.50702	-8.40157	na
17	-9.89033	-10.13227	-0.47097	-8.25695	na
18	-9.44995	-9.94442	-0.45000	-8.19875	na
19	-9.69396	-10.06883	-0.46162	-7.88360	H20(4.4)

Docked to **CYP2B6 (4RQL)**

Top pose dG, kcal/mol : -9.29444

Top pose VS score : -9.49358

Top pose LE, (kcal/mol)/atom : -0.44259

Pose with best free energy of binding has dG, kcal/mol : -9.29444

Pose with best free energy of binding has VS score : -9.49358

Hem A501 Interaction

Pose	dG, kcal/mol	VS score	LE, (kcal/mol)/atom	Rank score	Position/Distance
0	-9.29444	-9.49358	-0.44259	-7.02473	Cl(Fe,2.6,2.8)H16(2.9)C16(3.2)
1	-8.86734	-9.15809	-0.42225	-6.47334	na
2	-8.41812	-8.79319	-0.40086	-6.46049	C16(2.7,2.9)C17(3.6)
3	-8.91369	-9.19326	-0.42446	-6.23382	Cl(Fe,2.3,2.8)C(4.8) C16(3.0)H16(2.3)
4	-8.77110	-9.12098	-0.41767	-6.14984	na
5	-7.54233	-8.13996	-0.35916	-6.07964	C(4.5)C16(2.9)C17(3.5)
6	-9.09617	-9.34077	-0.43315	-5.93818	H19(2.9)C19(3.9)H20(2.7)C20(3.8)
7	-8.95922	-9.22773	-0.42663	-5.79717	H19(3.0)C19(3.4)H20(2.4)C20(2.9)
8	-7.98327	-8.47611	-0.38016	-5.55223	H18(2.7)C18(2.9)C19(2.9)
9	-9.16148	-9.41096	-0.43626	-5.49814	Cl(2.9)C(4.7)C16(2.6,2.7)C17(3.9)
10	-8.67649	-8.97817	-0.41317	-5.49681	H19(2.3)C19(2.8)H20(2.9)C20(3.5)
11	-7.99966	-8.57980	-0.38094	-5.37852	H20(2.7)C20(3.7)C19(3.7)
12	-7.99395	-8.48497	-0.38066	-5.35623	H19(2.4)C19(3.6)H20(2.6)C20(3.4)
13	-7.20244	-7.94251	-0.34297	-5.28622	C(4.8)C17(3.0)H17(2.5)C16(2.7)
14	-7.84854	-8.35509	-0.37374	-5.13899	na
15	-6.65037	-7.83377	-0.31668	-4.90412	H20(2.5)C20(3.3)C19(3.3,3.7,3.9)
16	-8.31869	-8.82307	-0.39613	-4.89726	Cl(2,6,3.0)H16(3.1)
17	-8.22054	-8.64408	-0.39145	-4.78118	Cl(Fe, 2.7)H16(2.4)C16(3.8)
18	-8.89560	-9.17142	-0.42360	-4.76562	Cl(Fe, 2.4, 2.8)H16(2.6)C16(3.3)
19	-8.24461	-8.68303	-0.39260	-4.67171	Cl(2.6,2.7,2.8)H16(3.0)C16(3.9)
20	-6.86582	-7.70995	-0.32694	-4.59398	A(4.8)C19(2.6,2.9)H19(2.2)C20(3.9)
21	-8.11162	-8.59402	-0.38627	-4.49547	S(4.3)H18(2.6)C18(3.5)C19(3.2)
22	-8.30665	-8.69915	-0.39555	-4.37019	na
23	-6.75822	-7.54771	-0.32182	-4.28577	C19(2.7,3.0)C18(3.7)C20(3.4)
24	-7.16339	-7.95195	-0.34111	-4.22577	C18(3.8)H18(2.7)A(4.6)C19(3.0)
25	-7.25512	-7.83384	-0.34548	-3.92176	C3(2.9,3.0)
26	-7.25013	-7.90202	-0.34524	-3.79012	Cl(2.5,2.8)H16(2.6)C16(3.5)
27	-7.56719	-8.15639	-0.36034	-3.68683	C19(2.9) H19(2.1)C20(3.9)
28	-8.06906	-8.51848	-0.38424	-3.64544	H20(2.6)C20(3.4)
29	-7.27721	-7.99945	-0.34653	-3.63842	na
30	-7.99483	-8.61872	-0.38071	-3.54414	C19(Fe,2.9,3.0,3.6)A(4.3)C18(3.8)
31	-6.75545	-7.74696	-0.32169	-3.16392	na
32	-6.75147	-7.36973	-0.32150	-1.91476	Cl(2.6)H16(3.4)C16(3.6)

Hem B501 Interaction

Pose	dG, kcal/mol	VS score	LE, (kcal/mol)/atom	Rank score	Position/Distance
0	-9.372	-9.583	-0.446	-7.548	Cl (Fe,2.6)H16(2.9)C16(3.3)
1	-9.289	-9.527	-0.442	-7.083	na
2	-9.130	-9.382	-0.435	-6.360	C19(3.7)H19(2.6) C20(3.7)H20(2.4)
3	-8.930	-9.212	-0.425	-6.226	C16(2.7,3.0)C17(3.7)
4	-8.999	-9.269	-0.429	-6.221	Cl(Fe,2.5)C(4.9)H16(2.5)C16(3.5)
5	-9.361	-9.491	-0.446	-6.058	C19(3.5)H19(3.0)C20(2.9)H20(2.3)
6	-8.733	-9.059	-0.416	-6.056	C19(2.8,3.0)H19(2.2)H20(2.9)C20(3.2)
7	-9.227	-9.492	-0.439	-5.873	C(4.7)C16(2.6,2.8,3.0)C17(3.5)
8	-8.930	-9.254	-0.425	-5.807	Cl(Fe, 2.4, 2.7)H16(2.5)C16(3.8)
9	-7.451	-8.044	-0.355	-5.795	C(4.7)C16(2.7,2.9)H16(2.3)C17(3.7)
10	-8.584	-9.036	-0.409	-5.509	A(4.6)C18(3.9)C19(2.7)H19(2.0)C20(3.6)
11	-8.860	-9.223	-0.422	-5.353	C16(2.6,2.7)C17(3.8)
12	-8.414	-8.808	-0.401	-5.332	Cl(2.6,2.7,2.9)H16(2.7)C16(3.4)
13	-8.257	-8.695	-0.393	-5.318	C19(3.6,3.8)C20(3.7)H20(2.8)
14	-8.686	-9.044	-0.414	-5.286	na
15	-8.623	-8.950	-0.411	-5.189	Cl(2.6,2.9)H16(3.1)
16	-8.383	-8.825	-0.399	-4.872	C19(2.9)H19(2.2)C20(3.3)H20(2.7)
17	-8.774	-9.096	-0.418	-4.682	na
18	-7.814	-8.311	-0.372	-4.676	C3(2.8,2.9)
19	-7.721	-8.365	-0.368	-4.544	C19(2.8)C20(3.0)H20(2.7)
20	-7.114	-7.987	-0.339	-4.481	C18(3.9)C19(2.9)H19(2.5)A(4.8)C20(3.8)
21	-7.734	-8.339	-0.368	-4.233	S(4.3)C18(2.8,2.9)H18(2.9)C19(3.0)
22	-8.850	-9.224	-0.421	-4.217	na
23	-7.331	-8.000	-0.349	-4.186	C18(3.6)C19(2.8,2.9)C20(3.7)
24	-7.613	-8.374	-0.363	-4.152	Cl(2.6,2.9)H16(2.5)C16(3.6)
25	-6.504	-7.618	-0.310	-4.146	C19(3.0)H19(2.2)C20(3.6)H20(2.5)
26	-7.244	-8.198	-0.345	-3.664	C19(Fe, 2.8)C20(3.0)H20(2.9)
27	-7.533	-8.188	-0.359	-3.364	S(4.2)A(4.4)C18(2.9)H18(2.9)C19(2.9)
28	-7.009	-7.823	-0.334	-3.278	C19(3.7)H19(2.7)C20(3.4)H20(3.4)
29	-8.386	-8.771	-0.339	-3.091	H16(4.3)H17(4.0)

Docked to **CYP3A4 (1TQN)**

Top pose dG, kcal/mol : -7.81038

Top pose VS score : -8.10207

Top pose LE, (kcal/mol)/atom : -0.37192

Pose with best free energy of binding has dG, kcal/mol : -7.84707

Pose with best free energy of binding has VS score : -8.04626

Pose dG, kcal/mol VS score LE, (kcal/mol)/atom Rank score

Hem A601 Interaction

Pose	dG, kcal/mol	VS score	LE, (kcal/mol)/atom	Rank score	Position/Distance
0	-7.81038	-8.10207	-0.37192	-7.00560	na
1	-7.06348	-7.47475	-0.33636	-6.92733	na
2	-6.84026	-7.44090	-0.32573	-6.91371	C19(3.4)
3	-6.84428	-7.47907	-0.32592	-6.82195	na
4	-7.10324	-7.51377	-0.33825	-6.80674	na
5	-7.08329	-7.57267	-0.33730	-6.80399	na
6	-7.46956	-7.79566	-0.35569	-6.77882	na
7	-7.23622	-7.74073	-0.34458	-6.75491	na
8	-7.42993	-7.77112	-0.35381	-6.70366	na
9	-6.89226	-7.36424	-0.32820	-6.69647	na
10	-7.49691	-7.72081	-0.35700	-6.68076	na
11	-7.52006	-7.78117	-0.35810	-6.67831	na
12	-6.99009	-7.41891	-0.33286	-6.67763	na
13	-7.48262	-7.92489	-0.35632	-6.67739	na
14	-7.58998	-7.87447	-0.36143	-6.62783	Cl(2.7)C(4.8)C16(3.8)H16(2.4)
15	-7.27465	-7.60293	-0.34641	-6.62373	na
16	-6.71747	-7.26669	-0.31988	-6.61989	na
17	-7.38485	-7.65056	-0.35166	-6.61778	na
18	-6.97376	-7.39398	-0.33208	-6.59237	na
19	-6.91414	-7.34364	-0.32924	-6.59009	na
20	-6.94367	-7.36156	-0.33065	-6.58504	na
21	-7.02972	-7.50405	-0.33475	-6.58379	na
22	-6.48830	-7.10897	-0.30897	-6.55855	na
23	-6.90759	-7.41694	-0.32893	-6.55006	na
24	-6.82371	-7.36185	-0.32494	-6.54534	na
25	-7.22602	-7.55891	-0.34410	-6.52892	na
26	-7.30596	-7.79384	-0.34790	-6.52305	Cl(2.9)
27	-6.30799	-6.95893	-0.30038	-6.48334	na
28	-7.84707	-8.04626	-0.37367	-6.46538	Cl(3.0)H16(3.2)
29	-6.87271	-7.33962	-0.32727	-6.41382	na
30	-7.76796	-8.16062	-0.36990	-6.39423	A(4.2)C(3.8,4.7)C13,C18-21
31	-7.51778	-7.83906	-0.35799	-6.38663	H16(2.9)
32	-7.60856	-7.89401	-0.36231	-6.37050	C20(3.5)
33	-7.41031	-7.68090	-0.35287	-6.36843	na

34	-6.18850	-7.06336	-0.29469	-6.34333	C18(3.6)C19(2.8)C20(3.6)
35	-6.82062	-7.22744	-0.32479	-6.33643	na
36	-7.76710	-8.07380	-0.36986	-6.32977	S(3.7)A(3.8)C9,C11,C16,C17,C19-20
37	-7.45125	-7.96179	-0.35482	-6.31011	S(Fe-3.0,3.4,3.5) H17(3.5)H18(2.0)C18,C19
38	-7.72365	-7.95811	-0.36779	-6.29397	S(3.7)Cl(2.9)C(3.5)C6(3.0)C13,C16
39	-7.43079	-7.74851	-0.35385	-6.26922	H20(2.8)C20(3.6)C19(Fe,2.9)
40	-7.16575	-7.50280	-0.34123	-6.23824	na
41	-7.16801	-7.41539	-0.34133	-6.21935	na
42	-6.88279	-7.38169	-0.32775	-6.18493	C19(3.0)H19(2.2)C20(3.8)
43	-7.52928	-7.82384	-0.35854	-6.12549	C19(Fe-2.9)A(4.9)H18(2.8)
44	-7.03302	-7.36743	-0.33491	-6.12024	na
45	-7.46610	-7.60371	-0.35553	-6.11041	na
46	-7.34274	-7.59982	-0.34965	-6.09542	H18(4.1,4.5)H19(4.0)
47	-7.33750	-7.86651	-0.34940	-6.09086	S(Fe-3.4,3.7)C18(3.0)C19(3.2)H17(3.7)
48	-7.39754	-7.67990	-0.35226	-6.03578	A(4.9)C19(2.9)C18(3.8)
49	-7.46732	-7.78606	-0.35559	-5.93533	H16(2.6)C16(3.8)
50	-7.34454	-7.60344	-0.34974	-5.88923	C19(2.9,3.0)C18(3.6)C20(3.6)
51	-7.24074	-7.70639	-0.34480	-5.81259	na
52	-6.28655	-6.99134	-0.29936	-5.80208	na

Docked to **CYP2C9 (4NZ2)**

Top pose dG, kcal/mol : -9.24751

Top pose VS score : -9.34128

Top pose LE, (kcal/mol)/atom : -0.44036

Pose with best free energy of binding has dG, kcal/mol : -9.24751

Pose with best free energy of binding has VS score : -9.34128

Hem A501 Interaction

Pose	dG, kcal/mol	VS score	LE, (kcal/mol)/atom	Rank score	Position/Distance
0	-9.24751	-9.34128	-0.44036	-7.33715	na
1	-7.94169	-8.51899	-0.37818	-7.20793	na
2	-9.06463	-9.18561	-0.43165	-7.09900	na
3	-8.06667	-8.52607	-0.38413	-7.08360	na
4	-7.98745	-8.36627	-0.38035	-7.07649	na
5	-8.40191	-8.60727	-0.40009	-6.95441	na
6	-8.63781	-8.83165	-0.41132	-6.95392	na
7	-7.98990	-8.55052	-0.38047	-6.88293	na
8	-8.34982	-8.67887	-0.39761	-6.87580	na
9	-7.88041	-8.40717	-0.37526	-6.86082	na
10	-7.73694	-8.05953	-0.36843	-6.79651	na
11	-8.48801	-8.65598	-0.40419	-6.77853	na
12	-7.99508	-8.40621	-0.38072	-6.76264	na
13	-8.36501	-8.73152	-0.39833	-6.73216	C18(3.8)C19(3.3)H19(2.6)H20(4.2)
14	-7.38532	-7.94070	-0.35168	-6.68070	na
15	-8.24653	-8.55872	-0.39269	-6.67273	C18(3.8)C19(3.7)H19(2.6)H20(4.3)
16	-8.60966	-8.79385	-0.40998	-6.65446	na
17	-8.59359	-8.71371	-0.40922	-6.62859	na
18	-8.06861	-8.30472	-0.38422	-6.55292	na
19	-6.87020	-7.65361	-0.32715	-6.52694	na
20	-8.48130	-8.61649	-0.40387	-6.48394	na
21	-7.97273	-8.34414	-0.37965	-6.47089	na
22	-8.55898	-8.69668	-0.40757	-6.46312	na
23	-8.07777	-8.37617	-0.38466	-6.45915	C16(3.9)H16(3.7)
24	-8.36817	-8.56099	-0.39848	-6.43025	na
25	-7.76550	-8.24316	-0.36979	-6.42664	na
26	-8.60217	-8.77973	-0.40963	-6.40314	na
27	-8.40296	-8.71183	-0.40014	-6.37785	na
28	-7.57001	-8.15417	-0.36048	-6.35735	na
29	-7.80714	-8.08853	-0.37177	-6.32053	na
30	-7.87036	-8.20778	-0.37478	-6.26510	H20(4.4)
31	-8.20177	-8.33462	-0.39056	-6.26170	na
32	-8.18521	-8.49973	-0.38977	-6.24748	Cl(2.9)C16(3.5)H16(3.4)
33	-8.01261	-8.26916	-0.38155	-6.23926	na
34	-7.90170	-8.62566	-0.37627	-6.23774	na
35	-8.64684	-8.74378	-0.41175	-6.21748	na

36	-7.88662	-8.48033	-0.37555	-6.20220	na
37	-7.81088	-8.07053	-0.37195	-6.13748	na
38	-8.38320	-8.65300	-0.39920	-6.13538	na
39	-8.58142	-8.73153	-0.40864	-6.10945	na
40	-8.47472	-8.66543	-0.40356	-6.08892	na
41	-7.79082	-8.15145	-0.37099	-6.06995	na
42	-7.63943	-8.11669	-0.36378	-6.05259	na
43	-7.30895	-7.82268	-0.34805	-6.01684	na
44	-8.13188	-8.32188	-0.38723	-5.88453	na
45	-7.97002	-8.36139	-0.37952	-5.85865	H19(4.5)H20(3.7)
46	-8.14329	-8.58054	-0.38778	-5.74195	na

Top pose dG, kcal/mol : -8.74741

Top pose VS score : -8.90242

Top pose LE, (kcal/mol)/atom : -0.41654

Pose with best free energy of binding has dG, kcal/mol : -9.22006

Pose with best free energy of binding has VS score : -9.37453

Hem B501 Interaction

Pose	dG, kcal/mol	VS score	LE, (kcal/mol)/atom	Rank score	Position/Distance
0	-8.74741	-8.90242	-0.41654	-7.16622	Cl(2.9)
1	-9.22006	-9.37453	-0.43905	-7.14306	na
2	-8.57097	-8.76984	-0.40814	-7.09591	na
3	-8.49109	-8.81148	-0.40434	-7.02547	na
4	-7.94355	-8.42255	-0.37826	-7.00014	na
5	-7.62547	-8.10952	-0.36312	-6.84273	na
6	-8.01213	-8.43364	-0.38153	-6.83087	na
7	-7.76596	-8.10377	-0.36981	-6.79704	na
8	-8.66031	-8.84915	-0.41240	-6.78576	na
9	-8.78560	-8.96836	-0.41836	-6.73420	na
10	-8.49789	-8.62203	-0.40466	-6.72808	na
11	-7.95161	-8.37040	-0.37865	-6.71373	na
12	-8.77371	-8.88899	-0.41780	-6.67823	na
13	-8.31957	-8.62466	-0.39617	-6.67161	H19(4.3)H20(3.3)
14	-7.54205	-8.13506	-0.35915	-6.62588	na
15	-7.69119	-8.24186	-0.36625	-6.61097	na
16	-7.83519	-8.15247	-0.37310	-6.60912	na
17	-8.15560	-8.49160	-0.38836	-6.54380	C18(3.9)C19(3.5)H19(2.7)H20(4.3)
18	-7.51129	-8.03209	-0.35768	-6.52100	na
19	-8.04826	-8.25614	-0.38325	-6.51117	na
20	-8.70935	-8.79168	-0.41473	-6.50056	na
21	-8.45855	-8.61760	-0.40279	-6.42989	na
22	-7.72439	-8.15222	-0.36783	-6.41502	na
23	-7.82373	-8.03566	-0.37256	-6.40527	na
24	-8.46620	-8.60165	-0.40315	-6.40027	na
25	-7.38648	-8.08899	-0.35174	-6.38369	na
26	-7.94522	-8.27787	-0.37834	-6.35873	H16(3.5,4.2)
27	-7.87954	-8.28449	-0.37522	-6.33708	na
28	-7.41054	-7.93667	-0.35288	-6.33576	na
29	-7.77591	-8.11520	-0.37028	-6.32509	H16(3.5,4.1)
30	-8.25208	-8.64199	-0.39296	-6.31882	C18(3.6)C19(3.0)H19(2.5)H20(4.2)
31	-8.40136	-8.61425	-0.40006	-6.28092	na
32	-7.76936	-8.07730	-0.36997	-6.27914	na
33	-8.62135	-8.76740	-0.41054	-6.25606	na
34	-7.88701	-8.16293	-0.37557	-6.24101	na
35	-8.49707	-8.66537	-0.40462	-6.24019	C16(3.6)H16(3.4,3.6)
36	-7.84634	-8.26600	-0.37364	-6.20908	na
37	-8.00424	-8.19059	-0.38115	-6.14522	na

38	-8.52013	-8.65040	-0.40572	-6.11604	na
39	-7.80653	-8.07118	-0.37174	-6.05581	na
40	-8.13821	-8.29900	-0.38753	-6.05290	na
41	-7.09374	-7.71189	-0.33780	-6.04247	na
42	-8.42256	-8.52152	-0.40107	-6.02615	na
43	-7.76625	-7.95248	-0.36982	-5.95863	na
44	-7.21478	-7.85253	-0.34356	-5.86669	na
45	-7.87113	-8.22230	-0.37482	-5.86233	na
46	-7.88763	-8.23433	-0.37560	-5.60746	na

Docked to **CYP2D6 (5TFT)**

Top pose dG, kcal/mol : -9.46026

Top pose VS score : -9.52742

Top pose LE, (kcal/mol)/atom : -0.45049

Pose with best free energy of binding has dG, kcal/mol : -9.46026

Pose with best free energy of binding has VS score : -9.52742

Hem A601 Interaction

Pose	dG, kcal/mol	VS score	LE, (kcal/mol)/atom	Rank score	Position/Distance
0	-9.46026	-9.52742	-0.45049	-8.11904	na
1	-9.10272	-9.25088	-0.43346	-8.10066	na
2	-9.30529	-9.42911	-0.44311	-8.07423	na
3	-9.05490	-9.16398	-0.43119	-8.03177	na
4	-9.38290	-9.43813	-0.44680	-8.00158	na
5	-8.96706	-9.14920	-0.42700	-7.96742	na
6	-9.17815	-9.31150	-0.43705	-7.93097	na
7	-9.08850	-9.20945	-0.43279	-7.92667	na
8	-9.02511	-9.10726	-0.42977	-7.91066	na
9	-8.80333	-9.11304	-0.41921	-7.88388	na
10	-9.19848	-9.31825	-0.43802	-7.82462	na
11	-8.86792	-8.96549	-0.42228	-7.73950	na
12	-9.20892	-9.36112	-0.43852	-7.71486	na
13	-9.44805	-9.51735	-0.44991	-7.71128	H19(3.7)
14	-9.06112	-9.25880	-0.43148	-7.70013	na
15	-9.40829	-9.48605	-0.44801	-7.68786	H19(3.7)
16	-8.71122	-8.95905	-0.41482	-7.66645	na
17	-8.61116	-8.80233	-0.41006	-7.66592	na
18	-8.50744	-8.76857	-0.40512	-7.65530	na
19	-9.24243	-9.40715	-0.44012	-7.64209	na
20	-8.95201	-9.16980	-0.42629	-7.62003	na
21	-9.21794	-9.37159	-0.43895	-7.57456	H18(4.0)H19(3.1)C19(3.7)
22	-8.38426	-8.67644	-0.39925	-7.49136	H19(4.4)
23	-8.73381	-8.87431	-0.41590	-7.44597	na
24	-8.99376	-9.18385	-0.42827	-7.38068	na
25	-8.17123	-8.46919	-0.38911	-7.21909	na
26	-8.75408	-8.90240	-0.41686	-7.20516	na

Top pose dG, kcal/mol : -9.47824

Top pose VS score : -9.58472

Top pose LE, (kcal/mol)/atom : -0.45134

Pose with best free energy of binding has dG, kcal/mol : -9.47824

Pose with best free energy of binding has VS score : -9.58472

Hem B601 Interaction

Pose	dG, kcal/mol	VS score	LE, (kcal/mol)/atom	Rank score	Position/Distance
0	-9.47824	-9.58472	-0.45134	-8.29308	C(4.9)
1	-9.37814	-9.50284	-0.44658	-8.27057	A(4.9)
2	-9.10085	-9.29194	-0.43337	-8.07718	A(4.9)H20(4.1)
3	-9.16836	-9.37582	-0.43659	-8.07229	C19(3.7)H19(3.3)
4	-8.86783	-9.19218	-0.42228	-8.06381	C16(3.7)H16(2.8)
5	-7.79163	-8.56355	-0.37103	-7.84079	na
6	-8.46798	-8.99989	-0.40324	-7.77655	na
7	-8.77540	-9.03038	-0.41788	-7.72610	na
8	-8.45742	-8.83563	-0.40273	-7.69322	N2(Fe-3.9)C1(2.8)
9	-8.65013	-8.84800	-0.41191	-7.67245	na
10	-8.72051	-9.07438	-0.41526	-7.66446	na
11	-8.90316	-9.18150	-0.42396	-7.63929	H19(4.1)
12	-8.71715	-9.14029	-0.41510	-7.60350	na
13	-8.74139	-9.00623	-0.41626	-7.59983	na
14	-8.36052	-8.80302	-0.39812	-7.58409	na
15	-8.77831	-9.12133	-0.41801	-7.58285	na
16	-9.03545	-9.09426	-0.43026	-7.57654	na
17	-8.49961	-9.06432	-0.40474	-7.55942	na
18	-8.70178	-9.07657	-0.41437	-7.54696	na
19	-8.80824	-9.07356	-0.41944	-7.51854	na
20	-8.60060	-8.80777	-0.40955	-7.51714	na
21	-8.66356	-8.92881	-0.41255	-7.51627	na
22	-8.30949	-8.86994	-0.39569	-7.51012	na
23	-8.25412	-8.59247	-0.39305	-7.50080	na
24	-8.53832	-8.82593	-0.40659	-7.49177	N2(Fe-3.7)C1(2.5)
25	-8.44052	-8.78058	-0.40193	-7.46549	na
26	-8.58886	-9.15923	-0.40899	-7.37910	na
27	-8.66525	-8.83658	-0.41263	-7.37610	na
28	-8.19148	-8.49822	-0.39007	-7.34594	na
29	-8.45904	-9.20533	-0.40281	-7.32517	na
30	-9.32020	-9.58982	-0.44382	-7.24134	Cl(2.7)C16(3.2)C17(3.7)
31	-8.20352	-8.61259	-0.39064	-7.21025	na
32	-8.39485	-8.66925	-0.39975	-7.16218	na
33	-8.66216	-9.00302	-0.41248	-7.12088	na
34	-7.89868	-8.57693	-0.37613	-7.08480	na
35	-8.04313	-8.56806	-0.38301	-7.05978	na
36	-8.50917	-8.65989	-0.40520	-7.02300	na

Table S4. Metabolites prediction of 2-CPTZ and CPZ using BioTransformer 3.0

No.	Chemical Formula	Major Isotope Mass (Da)	Reaction Type in Human	Structure
**2-Chlorophenothiazine**				
1	$C_{12}H_8ClNOS$	249.0015	S-Oxidation of diarylthioether to sulfoxide<br>Enzyme: Cytochrome P450 1A2	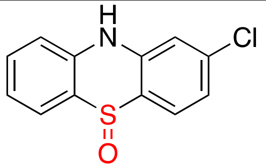
2	$C_{12}H_8ClNOS$	249.0015	Aromatic hydroxylation of fused benzene ring<br>Enzyme: Cytochrome P450 1A2	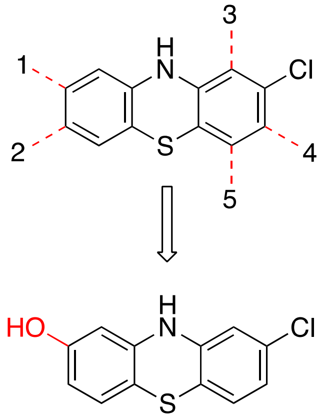<br>*(hydroxylation in 1 position)*
**Chlorpromazine**				
1	$C_5H_{11}NO$	101.084	N-Dealkylation<br>Enzyme: Cytochrome P450 1A2	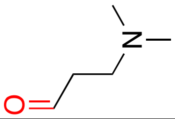
	$C_{12}H_8ClNS$	233.0065		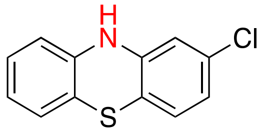
	$C_{16}H_{17}ClN_2S$	304.08		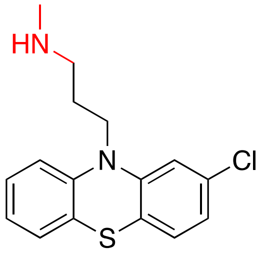
	$C_{15}H_{12}ClNOS$	289.0328		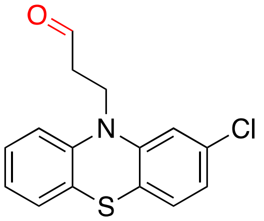

2	$C_{17}H_{19}ClN_2OS$	334.0906	Hydroxylation of acyclic aliphatic secondary carbon<br>Enzyme: Cytochrome P450 1A2	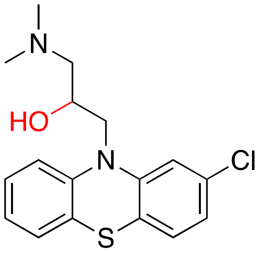
3	$C_{17}H_{19}ClN_2OS$	334.0906	S-Oxidation of diarylthioether to sulfoxide<br>Enzyme: Cytochrome P450 1A2	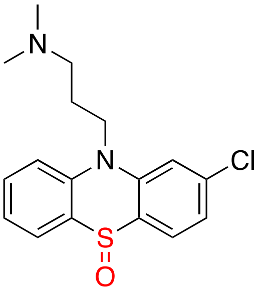
4	$C_{17}H_{19}ClN_2OS$	334.0906	Aromatic hydroxylation of fused benzene ring<br>Enzyme: Cytochrome P450 1A2	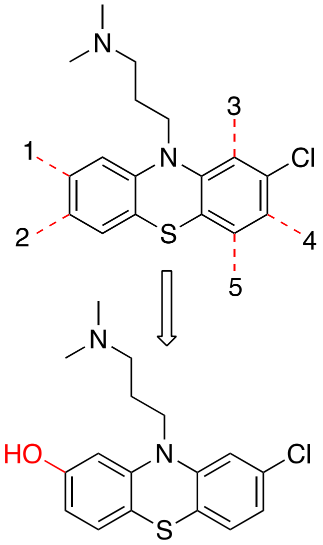<br>*(hydroxylation in 1 position)*
5	$C_{17}H_{19}ClN_2OS$	334.0906	N-Oxidation of aliphatic tertiary amine<br>Enzyme: Cytochrome P450 1A2	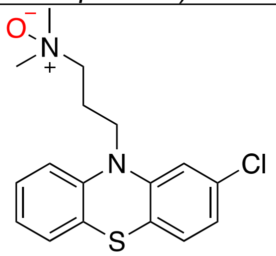
